# Institutions and the uneven geography of the first wave of the COVID‐19 pandemic

**DOI:** 10.1111/jors.12541

**Published:** 2021-06-07

**Authors:** Andrés Rodríguez‐Pose, Chiara Burlina

**Affiliations:** ^1^ Cañada Blanch Centre, Department of Geography and Environment London School of Economics London UK; ^2^ Social Sciences Gran Sasso Science Institute L'Aquila Italy

**Keywords:** COVID‐19, Europe, institutions, pandemic, regions

## Abstract

This paper examines the uneven geography of COVID‐19‐related excess mortality during the first wave of the pandemic in Europe, before assessing the factors behind the geographical differences in impact. The analysis of 206 regions across 23 European countries reveals a distinct COVID‐19 geography. Excess deaths were concentrated in a limited number of regions—expected deaths exceeded 20% in just 16 regions—with more than 40% of the regions considered experiencing no excess mortality during the first 6 months of 2020. Highly connected regions, in colder and dryer climates, with high air pollution levels, and relatively poorly endowed health systems witnessed the highest incidence of excess mortality. Institutional factors also played an important role. The first wave hit regions with a combination of weak and declining formal institutional quality and fragile informal institutions hardest. Low and declining national government effectiveness, together with a limited capacity to reach out across societal divides, and a frequent tendency to meet with friends and family were powerful drivers of regional excess mortality.

## 1 INTRODUCTION

In the first half of 2020 most of Europe became ravaged by the deadliest pandemic since the 1918 Great Flu. Just in the first 6 months of the year a total of more than 167,000 excess deaths relative to the average of the previous 5 years were computed in the 23 countries included in this analysis. The outbreak of the pandemic took the population and governments by surprise and, in some cases, led to a collapse or a near collapse of the health system and to a raft of containment and mitigation measures—including increased testing, contact tracing, lengthy lockdowns, quarantines, and restrictions to mobility—that had an uneven impact in the containment of the spread of the virus.

The pandemic, however, did not strike the whole of Europe in an even way. Some countries were hit far harder than others. In the UK, excess mortality during the first 6 months of 2020 surpassed 59,000. In Spain it topped 41,000, while in Italy and France the number of excess deaths almost reached 36,000 and 21,000, respectively. Relative to the population, the highest incidence took place in Belgium, Italy, Spain, and the UK. In contrast, many Central and Eastern European countries were spared. Deaths in the Baltic States, Bulgaria, Czechia, Hungary, and Slovakia were below the average for the preceding 5 years. Denmark and Germany also had no overall excess mortality during the first half of 2020.

Differences in excess mortality within countries were often higher than among them. Madrid, in Spain, witnessed the highest lethality in percentage terms across all the regions included in the analysis. The neighboring region of Castile‐La Mancha came third. At the opposite end, two other Spanish regions, Galicia and the Balearic Islands registered no excess deaths in the first half of 2020. Lombardy, in Italy, came second in this grim ranking and the pandemic impacted other rich Northern Italian regions, such as Trento, Bolzano, the Aosta Valley, or Emilia‐Romagna. In contrast, seven regions in the central and southern half of the country—Lazio, Molise, Sicily, Campania, Umbria, Basilicata, and Calabria—registered below average mortality levels. Some big cities, such as Paris, London, or Stockholm (on top of Madrid and Milan) were also hotspots of the illness. But other large cities remained mostly unaffected. Mortality was below trends in recent years in Norway, most of Finland, most of northern Germany, Denmark, Slovakia, Hungary, Bulgaria, and in many regions of South West France.

Since it became clear that COVID‐19 was no ordinary seasonal flu and that its effects could be devastating for, especially, certain groups of the population, scientists and decision‐makers have scrambled to try to study the factors behind the uneven geography of the COVID‐19 pandemic in Europe. At stake was far more than pure intellectual curiosity. Understanding not just the causes of the pandemic but what provoked its differential spread could lead to more adequate prevention, detection, and response measures for new waves of COVID‐19 and future epidemics. It would also help prevent situations like the collapse or near collapse of health systems that drove European societies to the brink in the months of March and April 2020.

This paper looks at these factors at the subnational level, where, despite some exceptions (e.g., Kapitsinis, 2020), the attention has been far scarcer than at national level. The regional level possibly represents a more adequate scale to evaluate the dimension and impact of the pandemic. Two objectives drive this paper. The first consists of mapping the incidence of the first wave of the COVID‐19 pandemic—over the period between January 1, 2020 and June 30, 2020—at a regional level in Europe. Second, it intends to explore which factors may be behind the uneven geography of the excess mortality detected in European regions over that period.

The reason for choosing excess mortality rather than other factors, such as number of cases or number of deaths officially attributed to COVID‐19 is related—as will be discussed in greater length in the methodological section—to the greater reliability and comparability of excess mortality data in a year in which no other epidemics have been reported.

The analysis will focus on the factors that have been highlighted by the rapidly mounting literature on the drivers of COVID‐19: from levels of agglomeration, regional wealth, density, local accessibility and connectivity to age structure, education, readiness of health systems, air pollution, and climate. Particular emphasis will be put on a dimension that, so far, has attracted less attention: the role of formal and informal institutions in explaining the uneven geography of the pandemic. Factors such as national and regional government quality and their change over time will be conflated with levels of regional autonomy and with more informal institutions, such as the frequency of personal interactions, the degree of trust, and the capacity of different groups within a society to build bridges with one another to assess what role they have played, if at all, in determining why some regions of Europe were devastated by the virus, while others relatively spared.

To achieve these objectives the paper adopts the following structure. A presentation of the uneven geography of the incidence of COVID‐19 across regions of Europe follows this introduction. The factors that have been identified as drivers of the variations in the incidence of the pandemic are analyzed in section three. This is followed by the introduction of the data and the methodology, before going over the results of the econometric analysis. The final section concludes and presents some preliminary policy implications.

## 2 THE UNEVEN GEOGRAPHY OF THE FIRST WAVE OF COVID‐19

### 2.1 Mapping excess mortality

**Figure 1:**
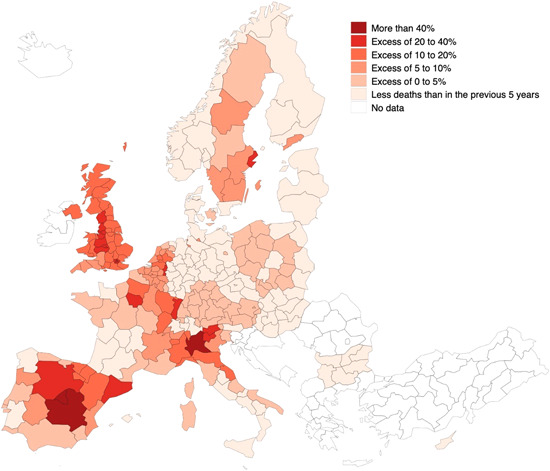
Excess death rates (as a percentage deviation from expected deaths, based on the previous 5 years) by region in the first 6 months of 2020. *Source*: Own elaboration [Color figure can be viewed at wileyonlinelibrary.com]

The European geography of COVID‐19 has been capricious. Figure 1 shows the map of excess mortality—as a percentage of deaths relative to expected deaths based on mortality in the previous 5 years—of the first wave of the pandemic across European regions. The pronounced differences in excess mortality over the first 27 weeks of 2020 are striking. Excess mortality exceeded expectations by more than 40% in Madrid, Lombardy, and Castile‐La Mancha. The excess was over 20% in 16 of the 206 European regions considered in the analysis and over 10% in 41. By contrast, in 85 of the 206 regions for which data are available, no excess mortality was recorded. In more than 41% of the regions the number of deaths in early 2020 was below the average of the previous 5 years, amid the worst pandemic to have hit Europe in a century.

The key hotspots of infection and mortality were often large cities: Madrid and Milan, but also London, Paris, Brussels, and Stockholm (Figure 1). Even in countries with a lower incidence of COVID‐19, the impact of the pandemic was more severe in some of the largest agglomerations, such as Helsinki, Budapest, Sofia, or Bremen, Hamburg, and Berlin. But this was not always the case. In Italy, two of the three largest cities—Rome and Naples—had excess mortality below the national average. The same could be said of Prague, Oslo, or of the largest urban agglomeration in Germany, the Ruhr Valley.

Finally, contrasts within countries were marked. In addition to the already mentioned differences within Italy and Spain, there was also a manifest North East/South East division in France and a South/North one in Germany. In the Netherlands, the pandemic affected the South and East to a greater extent than the more densely populated western provinces of Holland. In Poland, the division was more East/West with a lower incidence in the less developed regions of the East of the country (Figure 1).

**Figure 2:**
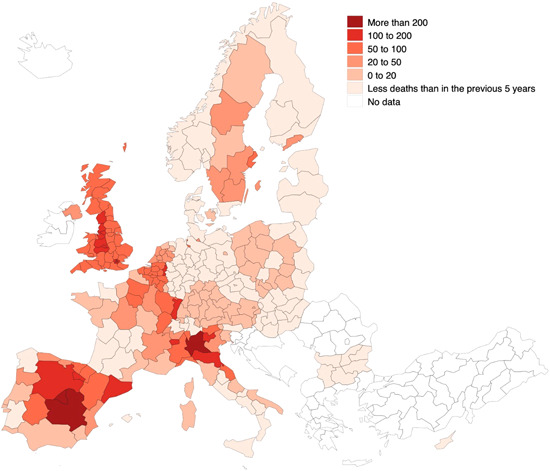
Excess death rates (per 100,000 inhabitants) by region in the first 6 months of 2020. *Source*: Own elaboration [Color figure can be viewed at wileyonlinelibrary.com]

Changing the reference from the excess percentage of expected mortality to excess mortality by population yields a similar map (Figure 2). Excess mortality remains concentrated in a limited number of countries, mostly in large, dense, and highly connected cities, but this is not the case everywhere and strong internal within‐country contrasts remain.

**Figure 3:**
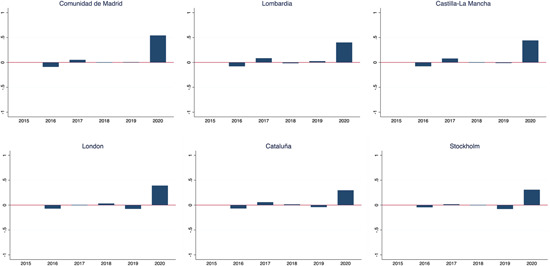
Year‐on‐year variation in mortality rates at regional level in the six hardest hit regions [Color figure can be viewed at wileyonlinelibrary.com]

The year‐on‐year variation in mortality rates affects every single region. Figure 3 reports the variation in the first half of 2020, relative to the same period in previous years, in the six most affected regions—Madrid, Lombardy, Castile‐La Mancha, London, Catalonia, and Stockholm, in that order. The mortality rates in 2020 almost doubled the values with respect to the period 2015–2019, with an increase in excess of 50% in the region of Madrid (the remaining regions are reported in the Appendix E).

To understand better if differences in pre‐ and post‐COVID‐19 excess mortality rates are significant, we conduct *t*‐tests. The results, reported in Table D1 in Appendix D, show that for 76% of the regions (160 in 211) these differences are statistically significant, clearly indicating the existence of a real COVID‐effect in the first half of 2020.

### 2.2 Change over time

**Figure 4:**
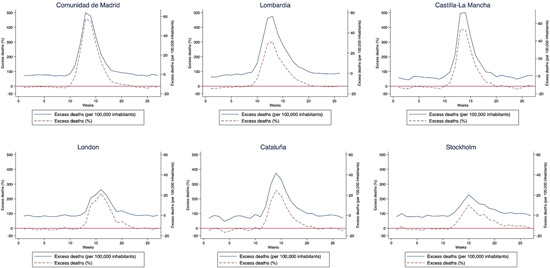
Evolution of excess mortality during the first wave of the COVID‐19 pandemic in the six hardest hit regions [Color figure can be viewed at wileyonlinelibrary.com]

Excess mortality linked to the COVID‐19 pandemic was also not evenly spread across the first half of the year. In those areas with the greatest excess deaths, it was time‐confined to a limited number of weeks, depending on when the largest outbreak of the epidemic happened in each place. Figure 4 displays the weekly evolution of excess mortality over the first 6 months of 2020 in the six hardest hit regions.

In all cases the bulk of excess deaths took place in a few weeks during the months of March and April. In Madrid excess mortality was in line with trends in the previous 5 years between Weeks 0 and 9 and from Week 20 onwards. The concentration of fatalities happened fundamentally between Weeks 12 and 15, when the excess mortality multiplied expectations by almost five times. In Lombardy mortality was below previous years in the first 7 weeks and rose steeply since then, with the biggest incidence taking place between Weeks 11 and 15 (Figure 4).

In Castile‐La Mancha and Catalonia the time patterns were like those found in Madrid, albeit delayed by one week. Overall excess mortality was also lower in Catalonia. The lethality of the outbreak in London started later—mortality only increased in late March. However, the impact was protracted in time, covering the whole of April and, with lower intensity, parts of May. In Stockholm, excess mortality also happened later (with a peak around Week 15) and was extended into May and June (Figure 4).

**Figure 5:**
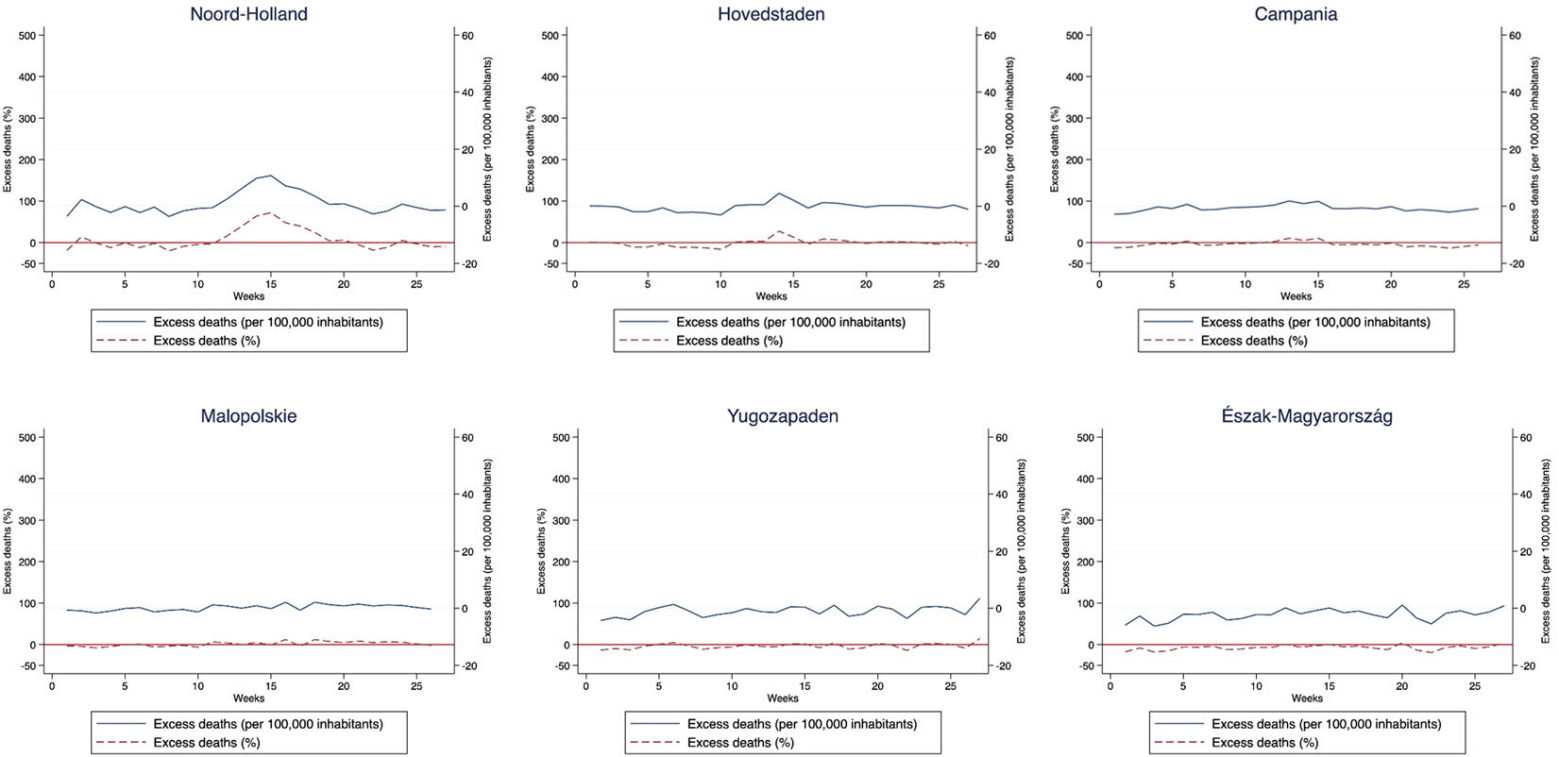
Evolution of excess mortality during the first wave of the COVID‐19 pandemic in other European regions​ [Color figure can be viewed at wileyonlinelibrary.com]

In contrast, in many other regions of Europe the incidence and lethality of COVID‐19 was far lower. In some regions, such as North Holland, excess mortality was also concentrated around Week 15 of the year, but more subdued than in regions with a similar population size and density (Figure 5). Copenhagen and Campania were also relatively spared, in spite of their large populations and high densities. And excess mortality was nonexistent in most of Central and Eastern European countries. In Lesser Poland (the region of Cracow) and the South East of Bulgaria (hosting Sofia) mortality rates throughout the period were those of a normal year. In Northern Hungary—the region with the lowest mortality rate of those considered—mortality was below that of the previous 5 years in virtually every week of the first half of 2020 (Figure 5). Excess mortality for every European region included in the analysis during the first wave of the pandemic is presented in Appendix A.

## 3 WHAT FACTORS EXPLAIN THIS UNEVEN GEOGRAPHY?

### 3.1 Size, accessibility, ageing, environment, and preparation

What explains this uneven regional geography of COVID‐19‐related excess mortality? Many different drivers of the expansion of the pandemic have been put forward. First and foremost, the spread of the pandemic has been linked with the presence of large agglomerations of people and greater connectivity (Coelho et al., 2020). The fact that large and highly connected cities were among the first affected put the focus on issues like agglomeration, density, and connectivity. Large and open cities, such as London, Paris, Madrid, or Milan are considered favorable environments for the diffusion of the disease. Large airports provided the entry points (Daon et al., 2020). The sheer concentration of people in high density environments did the rest. Large, dense, rich, and accessible European cities have therefore been regarded as a hotbed for the spread of coronavirus. But the role of these factors—and, especially, density—for the expansion and incidence of the disease has been challenged (e.g., Florida & Mellander, 2020; Hamidi et al., 2020; Nathan, 2020). Density may have influenced the timing of early outbreaks but has had less of an influence in COVID‐19‐related mortality over time (Carozzi et al., 2020). Empirical evidence shows that not every large European city fared in the same way as London, Paris, or Madrid. Regions hosting other large cities, like Rome, Oslo, Prague, or Budapest, experienced no excess mortality in the first half of 2020. That was also the case for regions with some large international airports. Hessen in Germany and Zurich in Switzerland had the 4th and 15th largest airports in Europe by passenger traffic in 2019, respectively. Yet the incidence of the pandemic in both regions was far lower than in other airport hubs. And the largest region by population in Europe, North Rhine‐Westphalia, with close to 18 million inhabitants and a high population density, also witnessed no excess mortality in the first 6 months of 2020.

Ageing, and differences in ageing trends across Europe, have also been signaled as a factor for excess mortality (e.g., Kashnitsky & Aburto, 2020). The pandemic has fundamentally targeted the elderly. Excess mortality has been particularly high among those over 65 and, in particular, those over 75. An ageing population in Northern Italy or, indeed, in regions of Northern France and Spain could be behind the particularly strong impact of the first wave of COVID‐19 in these areas. However, other areas of Europe with a severe ageing challenges, such as Bulgaria, Greece, Hungary, or the Nordic countries were, by and large, not as severely hit as Northern Italy, Madrid, Paris, or London.

Differences in levels of education are also expected to have played a role in the diffusion of the pandemic. A more educated population is more capable of shielding itself from the most devastating effects of COVID‐19 for several reasons. First, those with a higher level of education generally display a greater knowledge and awareness of the pandemic and of the measures needed to prevent infection (Zhong et al., 2020). Second, a higher average level of education in the population facilitates the possibility of conducting work from home, as well as protecting children through remote learning, preventing unnecessary outings, and covering unforeseen expenditures (Blundell et al., 2020, p. 293).

The potential role of environmental factors, ranging from climate conditions to air quality and pollution, has also been extensively discussed. The incidence of COVID‐19‐related cases has been connected with lower temperatures (Bashir et al., 2020; Gomes da Silva et al., 2020; Sajadi et al., 2020; Wang et al., 2020) and with lower (Sajadi et al., 2020; Wang et al., 2020)—and, in some cases, higher (Gomes da Silva et al., 2020)—humidity levels and precipitation. Air quality has attracted even more attention. Low air quality (Bashir et al., 2020; Filippini et al., 2020) and, in particular, the presence of high concentrations of inhalable particulate matter (PM 2.5) (Zoran et al., 2020) may have assisted in the spread of cases and, consequently, led to higher COVID‐19‐related mortality (Copat et al., 2020). The presence of pollutants in the air can enable the capacity of COVID‐19 and other respiratory viruses to spread easily, affecting lethality (Domingo et al., 2020).

Many European regions with poor air quality, both in terms of PM 2.5 and of carbon emissions, such as Lombardy, Piedmont, Veneto in Italy or Alsace in France, have had a high incidence of the virus. But this is far from the case everywhere. The worst levels of PM 2.5 in Europe are found in Central and Eastern Europe (OECD, 2020), above all in the regions of Silesia, Lesser Poland, Moravia‐Silesia, Central Moravia, and Central Slovakia. Yet the COVID‐19 related excess mortality during the first wave of the pandemic in these regions was limited.

The readiness of different health systems to withstand unusual or irregular events has also been scrutinized. The capacity to prevent and detect outbreaks early and to respond to them has been associated to a raft of different conditions within national and local health systems, among which the staffing of health personnel and the availability of hospital beds rank high (Bauer et al., 2020; Kandel et al., 2020; Liang et al., 2020). Insufficiently staffed and prepared health systems have been deemed slow to detect and respond to outbreaks, leading to a greater exposure of doctors, nurses, and other health workers to the virus (Ahmed et al., 2020), greater infection rates, and greater COVID‐19‐related mortality (Liang et al., 2020).

### 3.2 Formal and informal institutions

The impact of the pandemic may, however, also have been influenced by deep institutional variations. Institutions—or the rules of the game that shape human interaction and structure incentives for political, social, and economic exchange (North, 1990)—and their quality vary considerably across Europe. This happens both in terms of the more formal institutions, such as the rules, laws, and forms of organization in a society, and informal institutions, or the individual habits, collective routines, and social norms that determine the types and frequency of interaction (Amin, 1999).

There is considerable variation in formal institutional arrangements. In many countries of Europe government decisions are fundamentally taken in national capitals, with limited discretion to make and implement decisions at the local level. In others the authority of subnational tiers of government is far greater. Differences in regional authority are stark between, on the one hand, highly centralized countries, such as Ireland, Portugal, Finland, Denmark, or Poland, and, on the other, highly decentralized countries, such as Germany, Spain, or Belgium (Hooghe et al., 2016). Such differences are crucial in a pandemic whose incidence, as we have seen, differs greatly not only across countries, but also across regions within countries. These asymmetries in the incidence of the pandemic imply that local measures to tackle COVID‐19 can be as, if not more, effective than national ones (Buthe et al., 2020). It is often assumed that empowered subnational governments can respond faster and in a more and better targeted way than more remote national ones (Rodríguez‐Pose et al., 2009), provided they have the adequate capacity and efficiency to rise to the challenge. However, in the case of Europe, differences in quality of government are marked, with subnational governments in Northern Europe being distinctly of a better quality than in regions in the South Eastern corner of the continent (Charron et al., 2014). Despite the fact that variations in government quality persist over time, changes in subnational government efficiency have taken place, with noticeable improvements over the last decade in countries like the Baltics or Slovakia and a deterioration, at least in relative terms, in Italy, Spain, and, to a lesser extent, France (Ascani et al., 2021; Rodríguez‐Pose & Ketterer, 2020; Vollmer et al., 2020).

In any case, regional and local governments, especially in exceptional times, must operate within national legislative frameworks and, regardless of the level of decentralization, follow national policies, guidelines, and regulations. This implies a constant level of coordination during a pandemic that has tested the shared ruled capacity of national and subnational governments to find coordinated solutions (Marks et al., 2008). National governments have also taken the lead in adopting most of the tough decisions, especially during the first wave. They have been mostly responsible for the introduction and policing of often lengthy lockdowns and quarantines, the closure of public spaces, workplaces and educational institutions, restrictions to mobility, or the implementation of mandatory health rules, among others. The capacity and/or willingness of governments to, first, adopt tough decisions, and then efficiently design, coordinate, implement, and monitor them has been, however, highly variable. In some cases, European national governments have adopted highly stringent measures. According to the Oxford Coronavirus Government Response Tracker (Hale et al., 2020), the most stringent measures in Europe as of April 1, 2020 were in place in Serbia (not in the analysis), Croatia, Cyprus, and Italy. In contrast, Sweden and, especially, Belarus (not in the analysis) had notoriously lax COVID‐19 related restrictions.

But even more important than the stringency of the measures adopted has been the capacity of different governments to effectively implement such measures. This is greatly related to variations in national government effectiveness and its change over time. More effective governments are far more capable to respond rapidly to challenges and to adopt incisive measures more effectively, independently of political pressures (Kaufmann & Kraay, 2020). The level of change in government efficiency also affects the impact of the delivery of policies. Prolonged declines in government and civil service efficiency and creeping political pressures over decision‐making may have compromised the capacity and credibility of governments to commit to what are difficult to implement policies, highly restrictive of personal freedoms. Long periods of ebbing government quality may have reduced the capacity of governments to respond to deep shocks, such as the COVID‐19 pandemic, leading to difficulties in rising up to the challenge and in garnering the much‐needed political consensus in times of emergency.

While the adoption of measures to combat the pandemic fundamentally rests on formal institutions, local and regional informal institutions also play a crucial role in their success. How populations engage with one another and how frequently individuals see each other may facilitate or hinder the spread of the virus. As Richard Florida (2020) highlights, rather than density, what matters is the kind of density. In this respect crowdedness and frequent interactions within friend‐ and family‐circles are possibly more likely to have helped the spread of the virus.

Different forms of social capital, such as trust and the capacity to build consensus, are other informal institutions that may have determined variations in COVID‐19 incidence (e.g., Elgar et al., 2020). However stringent government measures to prevent and limit the spread of the virus may have been, their effectiveness may have come to nothing in environments with limited generalized trust and low trust in government capacity and in health‐related information. And the ability to build bridges across groups facilitates interaction and the generation of consensus. Bridging social capital is at the heart of civic engagement (Putnam, 1993), which is of crucial importance for the acceptance by the population of the tough responses needed during a pandemic.

However, all these institutional factors have featured far less prominently than agglomeration, density, ageing, health readiness, climate, or pollution in COVID‐19‐related research. Yet the slow reaction to the pandemic in countries with a high excess mortality during the first wave—like the UK, Spain, Belgium, and Italy—could have been related to declines in government efficiency in recent decades. Similarly, a greater habit of regularly meeting family and friends in celebrations could have driven up mortality in Mediterranean countries relative to the Nordics.

## 4 DATA AND METHOD

To test the variations in the link between different potential drivers—including institutions and other regional characteristics—and COVID‐19‐related excess mortality during the first wave of the pandemic, we rely on data stemming from several databases: EUROSTAT, the European Social Survey (ESS), the Quality of Government Institute, the Regional Authority Index (RAI), and the OECD.

### 4.1 The dependent variable

The dependent variable is excess mortality rates at a regional level in Europe during the first 6 months of 2020. The choice of excess mortality as the indicator of the incidence of the COVID‐19 pandemic in Europe is related to its far greater reliability in comparison to potential alternatives, such as the number or share of COVID‐19 cases or the official toll of COVID‐19 certified deaths. As indicated by numerous commentators (e.g., Dombey & Burn‐Murdoch, 2020), the reporting of cases and fatalities during the COVID‐19 pandemic has been besieged with problems and inaccuracies. Factors such as variations in the capacity to test at different times during the pandemic and differences and changes in the definition of what is a COVID‐19‐induced death, mean that doubts have been cast on the reliability of the reporting of new cases and of COVID‐19 deaths. In certain countries, COVID‐19 reporting may also have been massaged and subject to political vagaries, increasing its unreliability (Dombey & Burn‐Murdoch, 2020). In contrast, countries in Europe have been far better at counting deaths—a statistic they have been accurately collecting for decades—and the system has become far more sophisticated through the introduction of regular mortality monitoring (MOMO), both within countries and at the European level.

Excess mortality is thus increasingly regarded as the more adequate indicator to measure the overall incidence of COVID‐19 (Vanella et al., 2020). In general, studies that use excess mortality tend to report higher levels of lethality than simply relying on COVID‐19 officially attributed deaths, which, on the whole, underestimate the true incidence of COVID‐19 (Felix‐Cardoso et al., 2020).

We acknowledge that not all excess deaths in the first half of 2020 will be the result of COVID‐19. On the one hand, the fear of going to a hospital during an epidemic and the stress on the health system in many parts of Europe may have led to excess mortality as a consequence of the lack of immediate and/or early treatment of certain illnesses unrelated to COVID‐19. These additional deaths may, however, be considered indirect effects of COVID‐19. On the other hand, extensive lockdowns, quarantines, and the fear of going out during a pandemic are likely to have reduced other types of mortality, such as deaths provoked by road accidents or exposure to contagious diseases. This may explain in part why in some regions of Europe, and even in those with the greatest lethality of COVID‐19, excess mortality in early 2020 has been during some periods—especially in the second half of May and in June—lower than in previous years.

Data on excess mortality rates are extracted from EUROSTAT. EUROSTAT gathers these data from COVID‐19 reporting by national statistical institutes or equivalent. The data on the number of weekly deaths per region are available for a total of 23 European countries.^1^ These data are gathered at NUTS1 level for Belgium, Germany, and the UK, and at NUTS2 level for all other regions.

There are various mechanisms to calculate excess mortality. In our analysis we adopt the more common one: calculating the deviation from expected deaths in a given place in the first 27 weeks of 2020. As is commonly done, we calculate this as the ratio between the number of deaths per week in the first 6 months of 2020 (from the 1st of January until the 30th of June) and the average deaths for the same weeks in the period 2015–2019. We do this both as a percentage change as well as relative to the population (per 100,000 inhabitants) in a region.

### 4.2 Independent variables

The independent variables include population in 2019 and GDP in 2018 as a proxy for regional agglomeration (Combes et al., 2011), GDP per capita in 2018 to control for regional wealth, and population per squared kilometer in 2018 denoting density.

Connectivity is measured using accessibility by air and road. Accessibility by air is represented by the number of air passengers arriving in a region in 2018. Accessibility by road is calculated for 2014. The index includes a rather steep exponential function making accessibility negligible beyond four hours of travel.

The demographic characteristics of each region's inhabitants are measured by the median age of the population in 2019 and the share of population over 65 and over 75 in 2019. Education is calculated using the share of higher education graduates among adult population (aged 25 and over).

One of the main effects during the peak of the first wave of COVID‐19 was the saturation of hospitals and intensive care units. We control for the capacity of regional health care systems using two variables: the number of doctors per capita in 2017 and the number of hospital beds in that same year.

Environmental conditions are proxied with several measures for air pollution and climate. We introduce the exposure to air pollution by particulate matter (PM 2.5) at regional level in 2016 (using OECD data), as well as the total household carbon footprint (kgCO_2_e/cap), and the average household carbon footprint both at regional level in 2010 (Ivanova et al., 2017). We also resort to the average temperature in 2019 and the average precipitation in 2019, provided by the Agri‐4‐Cast EU‐JRC database, to control for climate.

Our main variables of interest, local institutions, are divided in the analysis into three macro blocks: level of autonomy, government quality, and social capital and trust.^2^

Regional autonomy is proxied by the RAI, compiled by Hooghe et al. (2016). This index encompasses 10 different dimensions, involving institutional depth, policy scope, fiscal autonomy, borrowing autonomy, and so forth. The RAI represents a composite measure of the relevance of subnational governments in the overall national decision process.

Formal institutions are measured through the indicator of government quality in 2017 at a regional level (Charron et al., 2016, 2019) and government effectiveness in 2018 at national level (Kaufmann & Kraay, 2020). The regional quality of government index is a composite indicator that takes into consideration not only the perception of the quality of public services, but also the level of corruption, and the impartiality of institutions. National government effectiveness captures perceptions of the quality of public services, the civil service, and the degree of its independence from political pressure (Kaufmann & Kraay, 2020). For the reasons presented in the theoretical section, we introduce these variables in levels and as changes for the longest period available—between 2010 and 2017 at regional level and 1998 and 2018 at country level—as, as indicated by Kaufmann and Kraay (2020), changes in governance quality are typically small over short time horizons and should only be considered over longer periods.^3^

Finally, we control for informal interactions by means of levels of sociability, regional trust, and social capital indices. These three indicators are computed using 2016 European Social Survey (ESS) data, a survey directed by the University of London within the European Research Infrastructure Consortium Forum. The primary aim of the ESS is to collect information at the individual level on personal and social well‐being, social capital and social trust, social exclusion, education and occupation, among other themes. This survey is conducted every 2 years and targets persons older than 15 years. Our measure of sociability and interaction is computed as the share of population in each region interacting at least once a week with members of his/her family or with friends. Generalized trust is used as our indicator of trust in the system. Social capital is proxied, following Putnam (1993, 2000), by what is known as bridging social capital. This is measured by the participation of individuals in a given region in voluntary associations aimed at forging connections among people from different cultural, social, economic, and other type of backgrounds.

The name, description, and source of each variable is reported in Appendix B, Table B1, while summary statistics appear in Table B2 in the same Appendix.

### 4.3 Method

The analysis is conducted by means of a set of ordinary least square regressions, introducing each regional (or national) characteristic alone, first, with the final model including all explanatory variables together.

The equation behind the full model adopts the following form:

$$\begin{array}{lll} {\text{Excessmortality}}_{i} & = & \beta_{0}+\beta_{1}{\mathrm{Agglomeration}}_{i}+\beta_{2}\mathrm{Regional}{\mathrm{Wealth}}_{i}+\beta_{3}{\mathrm{Density}}_{i}+\beta_{4}{\mathrm{Accessibility}\&\mathrm{Connectivity}}_{i}\\ & & +\;\beta_{5}\mathrm{Age}{\mathrm{Structure}}_{i}+\beta_{6}{\mathrm{Education}}_{i}+\beta_{7}\mathrm{Health}{\mathrm{System}}_{i}+\beta_{8}\mathrm{Air}{\mathrm{Pollution}}_{i}+\beta_{9}{\mathrm{Climate}}_{i}\\ & & +\;\beta_{10}{\mathrm{Autonomy}}_{i}+\beta_{11}\mathrm{Governemnt}{\mathrm{Quality}}_{i}+\beta_{12}{\mathrm{Social}\mathrm{Capital}\&\mathrm{Trust}}_{i}+\varepsilon_{i} \end{array}$$

where *i* are individual European regions. To mitigate for error correlation within regions, standard errors are clustered at regional (NUTS2) level. We also compute the variance inflation factor test (VIF) to control for potential multicollinearity among regressors (Miles, 2014). All regressions are run with country fixed effects, except for those that include national level government effectiveness variables.

Because not all data are available for all regions included in the analysis, the number of regions covered varies depending on the regression. The maximum is 206 regions, the minimum is limited to 122, when the number of doctors per capita is included with all the other controls. Regardless of the number of regions in each regression, the results tend to be consistent.

## 5 ANALYSIS OF RESULTS

### 5.1 Regressions considering individual factors

We first consider each group of individual factors. Table 1 assesses the link between agglomeration, regional wealth, density,^4^ and accessibility and connectivity, on the one hand, and excess mortality at a regional level during the first 6 months of 2020, on the other.

**Table 1:** Excess mortality and agglomeration, regional wealth, density, accessibility, and connectivity

Dependent variable: Excess mortality in the first 6 months of 2020	(1)	(2)	(3)	(4)	(5)	(6)	(7)
	Agglomeration		Regional wealth	Density	Accessibility and connectivity		
	OLS	OLS	OLS	OLS	OLS	OLS	OLS
Population 2019 (ln)	1.588						
	(1.186)						
GDP 2018 (ln)		2.163*					
		(1.174)					
GDP per capita 2018 (ln)			10.243**				
			(4.496)				
Population density 2018 (ln)				1.960**			
				(0.876)			
Air passengers 2018 (ln)					0.155		−0.053
					(0.212)		(0.175)
Accessibility by road 2014 (ln)						7.156***	7.218***
						(1.550)	(1.537)
Country fixed effects	Yes	Yes	Yes	Yes	Yes	Yes	Yes
No. of regions	206	192	192	206	206	201	201
*R* ^2^	0.367	0.383	0.408	0.389	0.353	0.518	0.518
Adjusted *R* ^2^	0.287	0.307	0.335	0.312	0.271	0.455	0.452
Degrees of freedom	182	170	170	182	182	177	176

*Note*: Clustered standard errors at regional (NUTS2) level in parentheses.

*** *p* < 0.01.

** *p* < 0.05.

* *p* < 0.1.

The results indicate that excess mortality was related to regional wealth, to having a larger economy, greater population density, and being more accessible and connected. However, the link between connectivity and excess mortality is stronger when accessibility by road is taken into account, rather than accessibility by air (Table 1). Regions more accessible within a 4 hour road trip witnessed a higher excess mortality than air passenger hubs. By contrast, the variable for population size in a region displays a nonsignificant coefficient (Table 1, Regression 1).

These results go mostly in line with expectations. Economically large, dense, and rich regions have been considered breeding grounds for the spread of COVID‐19. So have more connected and accessible regions, although here the expectation was that air transport would have been as relevant a determinant of the incidence of the virus as road accessibility, if not more. After all, airports have been under the spotlight as diffusers of COVID‐19 (Daon et al., 2020). The rapid decline in air transport during the first half of 2020 may have dented their association with COVID‐19 mortality. Road accessibility proved a more durable and lethal factor over time. Hence, as hinted by Florida et al. (2021), there may be a difference in the incidence of different types of connectivity between the geography of the first‐hit places and the final geography of COVID‐19 excess mortality, at least during this first wave of the pandemic.

The age structure of the population, its level of education, the preparation of the health system, and climate and air pollution as potential drivers of excess mortality are assessed in Table 2. Here, environmental factors go along with expectations and with most of the literature. The lethality attributed to COVID‐19 is greater in colder places and in regions with a higher degree of air pollution—although this seems to be only connected with the average household carbon footprint in a region. The coefficients for PM 2.5 and for the total household carbon footprint are positive and close to being significant (Table 2).

**Table 2:** Excess mortality and age structure, education, readiness of the health system, and environmental conditions

Dependent variable: Excess mortality in the first 6 months of 2020	(1)	(2)	(3)	(4)	(5)	(6)	(7)	(8)	(9)	(10)	(11)	(12)	(13)
	Age structure			Education	Health system			Air pollution			Climate		
	OLS	OLS	OLS	OLS	OLS	OLS	OLS	OLS	OLS	OLS	OLS	OLS	OLS
Median population age 2019	−0.606*												
	(0.337)												
Share of population over 65 2019		−50.631*											
		(29.443)											
Share of population over 75 2019			−63.008										
			(46.960)										
Share of adults with higher education 2017				0.236**									
				(0.075)									
Doctors per capita 2017 (ln)					−3.570		−3.286						
					(5.186)		(5.303)						
Hospital beds per capita 2017 (ln)						−2.943	−3.186						
						(5.700)	(4.195)						
Air pollution 2016 (ln)								7.993					
								(4.981)					
Total household carbon footprint 2010 (ln)									1.726				
									(1.270)				
Average household carbon footprint 2010 (ln)										21.416** (8.103)			
Average temperature 2019											−1.335**		−1.499**
											(0.344)		(0.391)
Average precipitation 2019												−0.705	−1.130
												(1.261)	(1.333)
Country fixed effects	Yes	Yes	Yes	Yes	Yes	Yes	Yes	Yes	Yes	Yes	Yes	Yes	Yes
No. of regions	206	205	205	204	174	180	162	195	172	172	197	197	197
*R* ^2^	0.386	0.382	0.370	0.400	0.295	0.287	0.301	0.380	0.378	0.413	0.430	0.385	0.433
Adjusted *R* ^2^	0.308	0.304	0.290	0.324	0.208	0.192	0.208	0.301	0.300	0.340	0.354	0.304	0.354
Degrees of freedom	182	181	181	180	154	158	142	172	152	152	173	173	172

*Note*: Clustered standard errors at regional (NUTS2) level in parentheses.

* *p* < 0.1.

** *p* < 0.01.

***p < 0.05.

However, most of the other factors considered in these fields are irrelevant or go against expectations. The coefficients for doctors and hospital beds per capita are insignificant, as is the coefficient for the share of the population over the age of 75. The two other indications of ageing societies—the share of the population over 65 and the median age of the population—have negative coefficients, significant at the 10% level. This is unexpected for an illness that has mostly targeted elderly people. *Ceteris paribus*, areas with a more educated population have also witnessed a greater number of excess deaths (Table 2).

The institutional factors considered also matter. Regions with a greater level of autonomy performed better than those subject to a more centralized regime (Table 3). So have regions in countries that have seen a long‐term improvement in their government quality and those with a greater bridging social capital, that is those where a greater share of the population is involved in activities with people of a different condition. Higher regional government quality was connected to a higher number of deaths, although the introduction of this variable suffers from multicollinearity, as revealed by the high VIF scores when it is included in the analysis (Table 3). The coefficients for the other indicators, including national government effectiveness, the change in regional government quality, the frequency of interactions, and the levels of trust are, all insignificant.

**Table 3:** Excess mortality and institutional variables

Dependent variable: Excess mortality in the first 6 months of 2020	(1)	(2)	(3)	(4)	(5)	(6)	(7)
		Government quality			Social capital and trust		
	Autonomy	Regional	National	Both	Interaction	Trust	Bridging
	OLS	OLS	OLS	OLS	OLS	OLS	OLS
Regional autonomy 2016	−0.363*						
	(0.108)						
Regional government quality 2017		4.319**		1.971			
		(2.141)		(2.141)			
Change in regional government quality 2010–2017		1.491		3.022			
		(2.643)		(2.374)			
National government effectiveness 2018			1.475 (1.141)	−1.763 (3.914)			
Change in national government effectiveness 1998–2018			−13.398*	−14.483*			
			(2.121)	(2.997)			
Frequency of meeting friends (once a week or more) 2016					−0.140		
					(0.087)		
Generalised trust 2016						2.665	
						(2.525)	
Bridging social networks 2016							−5.054*
							(1.811)
Country fixed effects	Yes	Yes	No	No	Yes	Yes	Yes
No. of regions	200	189	206	189	168	171	171
*R* ^2^	0.375	0.407	0.167	0.223	0.343	0.379	0.392
Adjusted *R* ^2^	0.297	0.329	0.159	0.206	0.268	0.310	0.325
Degrees of freedom	177	166	203	184	150	153	153

*Note*: Clustered standard errors at regional (NUTS2) level in parentheses.

* *p* < 0.01.

** *p* < 0.05.

***p < 0.1.

What happens when the independent variables are all considered together? (Table 4). Do certain factors matter more than others? Are institutional factors more connected to excess mortality in the first half of 2020 than some other factors, such as agglomeration, connectivity or age structure? Table 4 reports the results of conducting this analysis. When all the factors considered are included together, excess mortality in the first half of 2020 happened fundamentally in wealthier regions that are more accessible by road. Once again accessibility by road trumped accessibility by air in its connection with excess mortality.

**Table 4:** Full model

Dependent variable: Excess mortality in the first 6 months of 2020	(1)	(2)	(3)	(4)	(5)	(6)	(7)	(8)
	OLS	OLS	OLS	OLS	OLS	OLS	OLS	OLS
Population 2019 (ln)	0.419	0.918	1.040	−0.803	1.703	0.932	0.703	0.953
	(1.196)	(1.229)	(1.344)	(2.362)	(1.598)	(1.405)	(1.479)	(1.475)
GDP per capita 2018 (ln)	10.564***	10.220**	9.034***	7.745**	6.299**	7.824**	15.083***	6.583*
	(2.927)	(4.485)	(2.805)	(3.070)	(3.044)	(3.788)	(3.546)	(3.556)
Population density 2018 (ln)	−0.310	1.351	−2.004*	−1.426	−2.700**	−1.837*	−1.007	‐1.723
	(0.541)	(1.034)	(1.025)	(1.108)	(1.058)	(1.072)	(1.252)	(1.065)
Air passengers 2018 (ln)	−0.314	−0.198	−0.194	−0.274	−0.429	−0.172	−0.156	‐0.317
	(0.239)	(0.223)	(0.287)	(0.347)	(0.335)	(0.300)	(0.333)	(0.313)
Accessibility by road 2014 (ln)	5.635***	4.845**	6.714***	5.491***	5.880***	7.825***	6.778***	9.526***
	(1.712)	(2.071)	(1.634)	(1.816)	(1.991)	(2.686)	(2.158)	(2.766)
Share of population over 65 2019		−20.896	−26.778	−36.614	−62.329*	−29.443	9.066	‐21.209
		(24.026)	(25.100)	(26.978)	(32.399)	(25.197)	(30.945)	(26.228)
Share of adults with higher education 2017		−0.216*	0.183*	0.175*	0.160*	0.195*	0.233**	0.256**
		(0.128)	(0.095)	(0.103)	(0.093)	(0.104)	(0.115)	(0.105)
Average temperature 2019		−1.818***	‐0.557*	‐0.729*	0.133	−0.676*	−0.782*	‐1.011**
		(0.528)	(0.326)	(0.376)	(0.455)	(0.386)	(0.436)	(0.468)
Average precipitation 2019		−2.634**	−2.830**	−2.793**	−2.093*	−2.879**	−4.679***	‐3.425**
		(1.162)	(1.292)	(1.347)	(1.232)	(1.304)	(1.559)	(1.466)
Regional autonomy 2016			−0.459***	−0.373***	−0.441***	−0.406***	−0.781***	‐0.368*
			(0.107)	(0.113)	(0.104)	(0.150)	(0.278)	(0.210)
Frequency of meeting friends (once a week or more) 2016			0.210***	0.237***	0.127*	0.199***	0.201**	0.171**
			(0.064)	(0.070)	(0.069)	(0.066)	(0.081)	(0.074)
Change in regional government quality 2010‐2017			3.511*	2.654	1.576	3.253	6.354**	3.301
			(1.921)	(2.029)	(2.137)	(2.090)	(2.567)	(2.396)
National government effectiveness 2018			−9.702***	−12.553***	−9.427***	−11.199***	−8.693**	‐7.699**
			(3.112)	(3.536)	(3.327)	(3.768)	(3.473)	(3.554)
Change in national government effectiveness 1998‐2018			−17.787***	−16.865***	−12.649***	−16.032***	−32.281***	‐17.533**
			(3.001)	(2.940)	(3.005)	(3.706)	(10.513)	(6.690)
Generalised trust 2016			1.994	4.802	−0.514	1.480	−3.273	‐0.908
			(3.147)	(3.357)	(3.525)	(3.396)	(4.707)	(3.430)
Bridging social networks 2016			−3.668**	−5.232***	−5.139***	‐3.116*	−4.078*	‐2.797
			(1.692)	(1.951)	(1.864)	(1.858)	(2.242)	(1.822)
Total household carbon footprint 2010 (ln)				2.759*				
				(1.610)				
Average household carbon footprint 2010 (ln)					20.141***			
					(6.504)			
Air pollution 2016 (ln)						−4.041		
						(6.724)		
Doctors per capita 2017 (ln)							−6.063*	
							(3.131)	
Hospital beds per capita 2017 (ln)								‐6.967**
								(3.104)
Country fixed effects	Yes	Yes	No	No	No	No	No	No
No. of regions	187	182	148	128	128	148	122	124
*R* ^2^	0.565	0.629	0.491	0.516	0.540	0.493	0.477	0.491
Adjusted *R* ^2^	0.497	0.559	0.428	0.441	0.469	0.427	0.392	0.409
Degrees of freedom	161	152	131	110	110	130	104	106
*F* test			10.02	9.207	11.77	9.518	5.118	5.968

*Note*: Clustered standard errors at regional (NUTS2) level in parentheses.

*** *p* < .01.

** *p* < .05.

* *p* < .1.

Areas of Europe with a higher level of education and located in cooler and dryer environments also experienced a higher excess death toll (Table 4), as pointed out by previous research (e.g., Sajadi et al., 2020; Wang et al., 2020). The coefficients for pollution also go along with most of previous research (Bashir et al., 2020; Copat et al., 2020; Domingo et al., 2020; Filippini et al., 2020). Regions with a greater overall and with a higher than average household carbon footprints were hit harder by the first wave of the pandemic (Table 4, Regressions 4 and 5). So was the case of areas with a weaker degree of preparation of health systems. The coefficients for the number of doctors and hospital beds per capita—which were insignificant when both factors were considered in isolation (Table 3)—become negative and significant when analyzed in combination with other potential drivers of the deadliness of the pandemic (Table 3, Regressions 7 and 8). This is in line with the mounting amount of research pointing to the quality and preparedness of local health systems as a fundamental factor in lessening the impact of the pandemic (Ahmed et al., 2020; Kandel et al., 2020; Liang et al., 2020).

In contrast, most agglomeration factors appear as irrelevant for excess mortality. This is the case of the size of the population of the region and of its density. The size of the population displays an insignificant coefficient in every regression, whereas density, in line with the work of Carozzi et al. (2020), is either insignificant or marginally negative. This result contrasts with that of the degree of sociability of the population of a region. Regions where its citizens tend to meet more frequently with friends and family suffered more during the pandemic than densely populated regions. As Florida (2020) indicates, the impact of COVID‐19 may be unrelated to density and far more to how people interact. Excess mortality seems to have been more about habits of engaging with others and, perhaps, with crowdedness. Density counts for little if people do not meet and hug each other, if they avoid long and frequent interactions with friends and family, which have been an important source of many COVID‐19 outbreaks.

The remaining results emphasize the importance of our main variables of interest, formal and informal institutions, in the diffusion and impact of COVID‐19 across the regions of Europe. On the more informal side, it is not just that different modes and traditions of social engagement contribute to the lethality of the pandemic, but that a greater capacity in the population to engage with people from different origins and conditions seems to have acted as a barrier to the lethality of the virus. The coefficients for bridging social network are always negative and mostly highly significant (Table 4). Variations in the levels of trust, by contrast, seem to have played a more limited role in the differences in excess mortality observed across regions in the first half of 2020 (see also Elgar et al., 2020).

Formal institutions also matter. The model is run without the degree of regional government quality, as the introduction of that variable was associated with multicollinearity. The results indicate that, in the case of fighting the pandemic, government effectiveness at the national level mattered more than that at regional level. Countries with a better government effectiveness and those that had a greater improvement over time in their government effectiveness are far more likely to have had lower levels of excess mortality in the first 6 months of 2020. Regions with a greater level of autonomy also fared far better and, although the coefficient for the change in regional government quality is always positive and at times significant, it appears that national government effectiveness was far more important than regional government effectiveness in taming the virus, at least during the first wave (Table 4).

The ageing variable is always insignificant when included with other controls, whereas the levels of education in a region tend to be positively connected to higher excess mortality.

These general considerations are reinforced by analyzing the connection between individual variables and excess mortality in the first 6 months of 2020 in our base regression: Table 4, Regression 3. This regression includes all the variables of agglomeration, density, accessibility, ageing, education, environmental conditions, and the formal and informal institutions. The strongest association with the excess mortality as a percentage of expected deaths is related to the two national government effectiveness variables. A one standard deviation (*SD)* increase in government effectiveness in 2018 or in improvements in government effectiveness between 1998 and 2018 was connected to a reduction in excess mortality during the first half of 2020 of 0.44 *SD*s in both cases (Table 4, Regression 3). Greater road accessibility had the same effect, albeit with a positive sign. In places more accessible by road, a 1 *SD* rise in accessibility increased excess mortality by 0.44 *SD*s. Other relationships that stand out are linked to regional wealth (a 0.38 *SD* increase in excess mortality), regional autonomy (0.30 reduction) and sociability, proxied by the frequency of meeting with friends and family (0.28 reduction) (Table 4, Regression 3).

To better understand the mechanisms behind the institutional factors, we also interact the role of trust and the RAI index with the regional quality of government. The results of the full model are reported in Tables D3 and D4 (in the Appendix D): the signs and magnitude of the coefficients are almost identical to those reported in Table 4. However, the interaction terms never become significant, revealing a limited role for the interaction between trust, on the one hand, and levels of autonomy and government quality, on the other.

As an additional robustness check, we rerun the models changing the missing values in the variables by zeroes, to make the data more balanced. We also include a set of relative dummies to indicate those “artificial” zeroes. The results for the first three tables (Table D5–D7 in the Appendix D) show no differences between using artificial zeroes and the findings reported in Tables 1, 2, 3. When the full model is considered (Table D8), we obtain in some variables the same signs of the coefficients of Table 4, but with a lower (or in some cases no) significance, as for the number of doctors or carbon emissions. The only exception is represented by bridging social capital, which is higher in magnitude and more significant, expressing a higher connection of informal institutions when all the regions in the sample are included.

Changing the dependent variable from excess mortality as a percentage of expected deaths to a share of the population—which we do as a robustness test in Tables C1–C4 in Appendix C—yields, in general, marginally higher explanatory capacities (as indicated by the higher *R* ^2^ in the regressions), but hardly changes the significance and association of the different variables to excess mortality during the first half of 2020. This is particularly the case for the full model (Table C4).

## 6 CONCLUSIONS

Since the arrival of the COVID‐19 pandemic to Europe there has been no shortage of research looking into why it spread as fast as it did and why it affected some people and places far more than others. Most of the research has tended to point to large, dense, highly connected, and polluted cities in countries with weak or weakened health systems as the main hotspots for the entry and the diffusion of the virus.

However, the analysis at subnational level has been far less prominent and the salience of other factors and, in particular, of the institutional dimension in the spread of COVID‐19 has attracted far less attention.

In this paper, we have aimed to address these shortcomings by examining the uneven regional geography of COVID‐19 excess mortality in Europe and connecting it to the distinct variations in formal and informal institutions in evidence across the continent.

The analysis has revealed both the large cross‐ and within‐country inequality in the impact of the virus. During the first 6 months of the year, excess mortality exceeded expectations by more than 40% in only three regions. 16 regions had excess mortality over 20% and 41 over 10%. This latter number represented half of the regions that witnessed mortality rates that were below those expected based on average mortality during the same period in the previous 5 years. Preventive measures and lockdowns may have led to a reduction of other types of mortality (e.g., road accidents, non‐COVID‐19‐related infections diseases) and help explain this below average mortality in many parts of Central and Eastern Europe, but also in Northern Germany, South East France, and in the Nordic countries, outside Sweden.

The main message coming from the analysis is that the early incidence of COVID‐19, in terms of excess mortality, has been related to a rather complex set of factors, some of which—such as accessibility, climate, pollution, or the preparedness of the local health system—had already been pointed out by the literature, whereas the role of others may have been somewhat overstated. A whole raft of institutional factors, which, until recently, have attracted limited attention, may also be at the forefront of differences in the incidence of the virus. Overall, the most affected European regions during the first wave of the pandemic have been relatively large and connected regions, in colder and dryer climates, with high air pollution levels and relatively poorly endowed health systems. Most agglomeration factors and, in particular, population density have been relatively marginal to the impact of the disease, while excess mortality seems much more related to advantages in accessibility by road rather than by air.

While these results add interesting nuance to existing knowledge, the biggest novelty lies in how institutional factors have shaped the impact of the virus. The most ravaged regions of Europe by COVID‐19 were those with a combination of weak and declining formal institutional quality and brittle informal institutions. Low and declining government effectiveness at the national level—much more than at the regional one—emerges as one of the key factors behind excess mortality in the first half of 2020. Regions in countries with weak and declining government effectiveness were less capable of adopting, implementing, and monitoring the often‐tough decisions that have been proven to be more effective in the fight against the pandemic. Citizens in these countries consequently endured the lower capacity of their governments to prepare and rapidly respond to the challenges of COVID‐19 and to garner the necessary political consensus to take tough decisions. Weak and weakening national government effectiveness also restricted their capacity to react early to what was taking place elsewhere and to learn from the successes and mistakes of measures adopted in other places. Weaker and weakening government and bureaucratic efficiency may have also led to frequent changes of direction in the adoption and implementation of policies and may have been at the source of chronic failures in protecting key health and other types of essential workers from infection. They may have produced an inability to coordinate public intervention effectively in all types of measures, including in areas such as the procurement of medical and protective equipment for key workers. A better local performance in those regions with more regional autonomy did not compensate for the failures in national government effectiveness.

Informal institutions also played their part in the striking differences in excess mortality. COVID‐19‐related excess mortality was far higher in regions where people meet more often, but where the capacity to build bridges across different groups to create consensus is weak.

Weak government effectiveness, weak consensus building capacity, and frequent within group meetings have led to a fragile response to the COVID‐19 pandemic. These conditions limited the capacity to find consensual solutions understood, respected, and followed by the population. Hence “every man/woman for himself/herself” type of strategies often prevailed in these regions. Even in the case of widely accepted measures, their implementation over time may have left a lot to be desired, relative to other areas of Europe. Overall, this type of regions struggled to put in place effective and co‐ordinated responses when the virus struck and failed to instill the necessary trust to bring the population on board over the long periods required to combat the pandemic. Unfortunately, this possibly resulted in higher death rates linked to the pandemic.

Confronting the challenges of COVID‐19 and future health or natural shocks requires effectively tackling institutional bottlenecks. While little can be done in terms of addressing agglomeration and density and climate is an exogenous factor, improving national government effectiveness and redressing government quality decline should be priorities to respond to this pandemic and to prepare societies for future social, economic, and political challenges. Addressing profound social cleavages and finding the mechanisms to reach greater consensus across divides is also essential. Any delays or failure to improve in this respect have, as we have seen during the first wave of the COVID‐19 pandemic, an important cost in lives.

## Supporting information

Supporting information.

## Data Availability

The data that support the findings of this study are available in LSE Research Online at https://urldefense.com/v3/__http://eprints.lse.ac.uk/__;!!N11eV2iwtfs!9py‐1sjkvGqdh_EF1t9wzhvkGeaExaKvu2XUrswe‐WwtnmqCAMb‐8OSOEbw9vnyvHg$. These data were derived from the following resources available in the public domain:—EUROSTAT, https://urldefense.com/v3/__https://ec.europa.eu/eurostat__;!!N11eV2iwtfs!9py‐1sjkvGqdh_EF1t9wzhvkGeaExaKvu2XUrswe‐WwtnmqCAMb‐8OSOEbwRm8VLng$.

## Footnotes

1 These are Austria, Belgium, Bulgaria, Czechia, Denmark, Estonia, Finland, France, Germany, Hungary, Italy, Latvia, Lithuania, Luxembourg, Netherlands, Norway, Poland, Portugal, Slovakia, Spain, Sweden, Switzerland, and the UK.

2 Other recent papers, such as Oksanen et al. (2020) and Charron et al. (2020), have focused on the role of institutions on the incidence of the COVID‐19 pandemic. Oksanen et al. (2020) put the emphasis on country differences in measures taken to stop the spread of the virus. Charron et al. (2020) put the stress fundamentally on political polarization.

3 The timing of the impact of the pandemic is another important factor to consider, as it approximates the way in which governments reacted to COVID‐19 threats. Italy was hit first by the pandemic and had limited time to respond. Countries and regions that were hit later were afforded some additional time to learn from the Italian experience. This implies that while some regions had to deal not only with an intrinsic deterioration in the quality of government, but also with a “surprise‐effect,” like many Italian regions, others had a short window of opportunity to adapt and prepare. However, if we run the correlation between the stringency index (which measures the variation in governments' responses: https://ourworldindata.org/grapher/covid-stringency-index) and the quality of government, there is a negligible correlation between these two variables (see Table D2 in Appendix D). This is, however, a factor that would deserve greater attention than what can be afforded in this type of article.

4 We use density at NUTS2 level rather than NUTS3 to have the same unit of analysis as the dependent variables. We recognize that density by municipalities or provinces may better approximate the effect of highly concentrated cities or provinces within the same region. However, we prefer to maintain the consistency of our estimates with all the same geographical level.
