# Supplementary material for: Institutions and the uneven geography of the first wave of the COVID‐19 pandemic
Source: J Reg Sci. 2021 Jun 7;61(4):728–52. doi: 10.1111/jors.12541 (PMC8242880; doi:10.1111/jors.12541)
Supplement: Supplementary file 1 — Supporting information. [file JORS-61-728-s001.pdf]

Appendix A

Figure A1. Evolution of excess mortality during the first wave of the COVID-19 pandemic by Country.

AUSTRIA

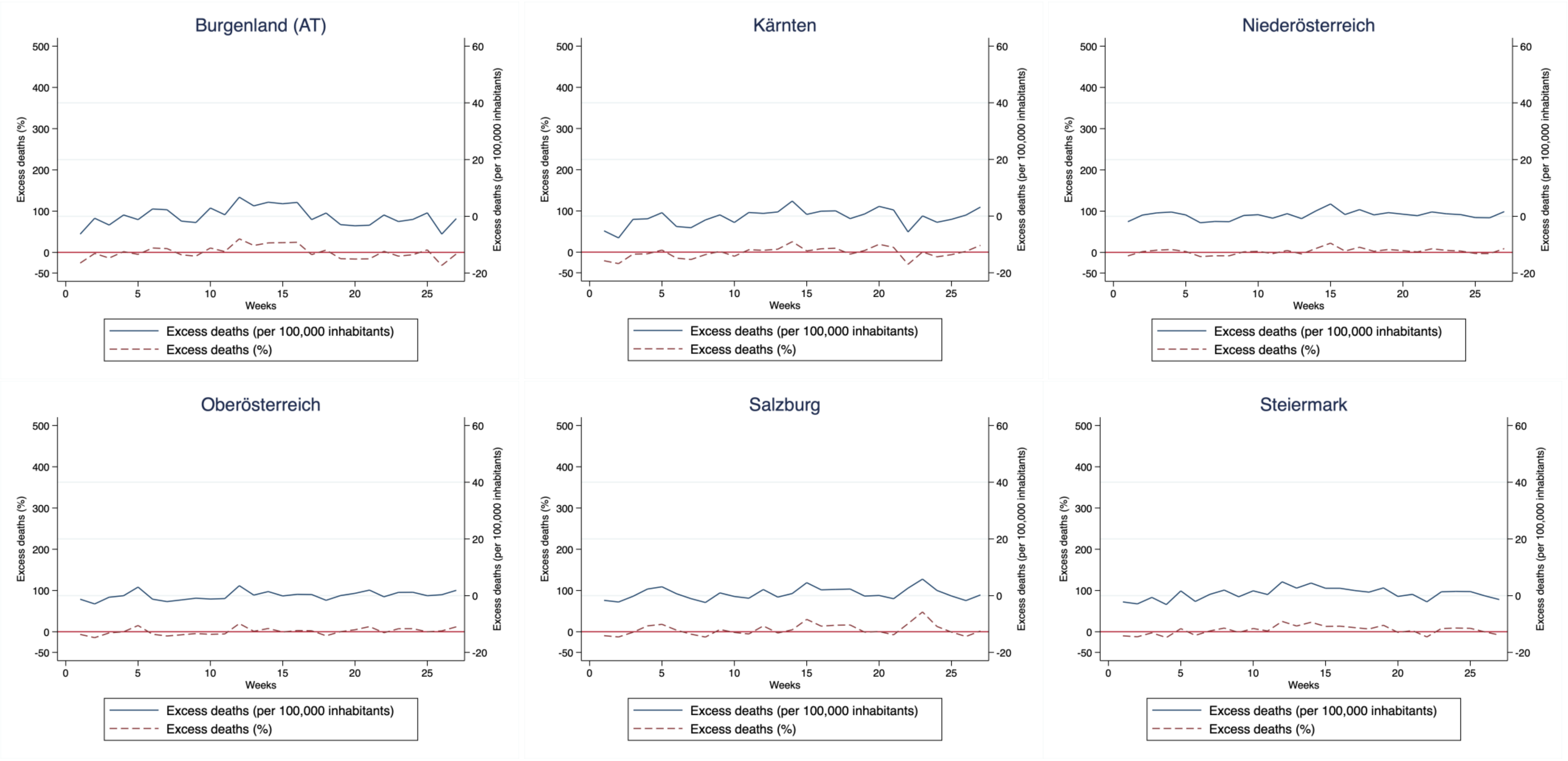

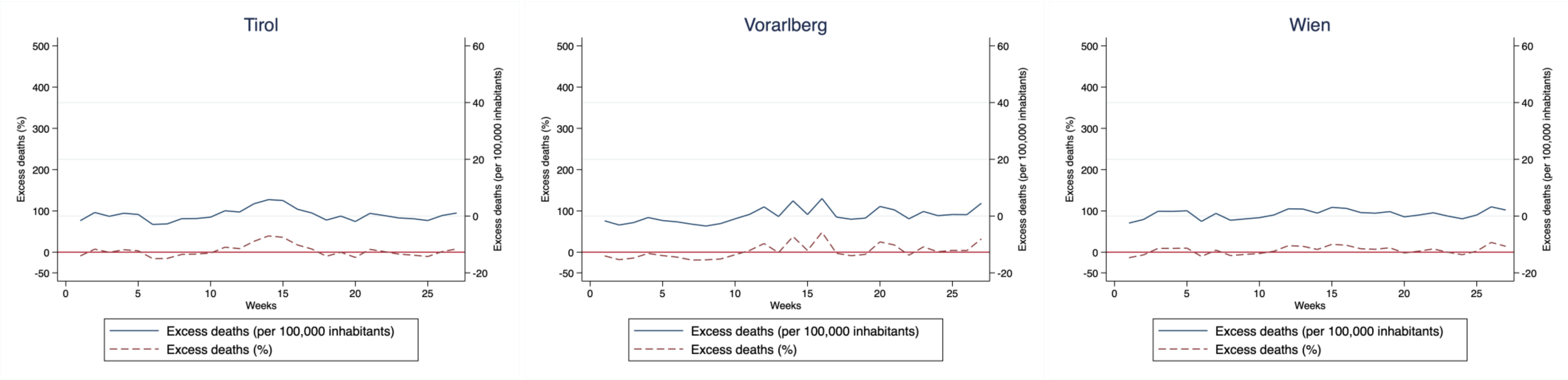
Tirol
Vorarlberg
Wien
Excess deaths (%)
Excess deaths (per 100,000 inhabitants)
Weeks
Excess deaths (per 100,000 inhabitants)
Excess deaths (%)

BELGIUM

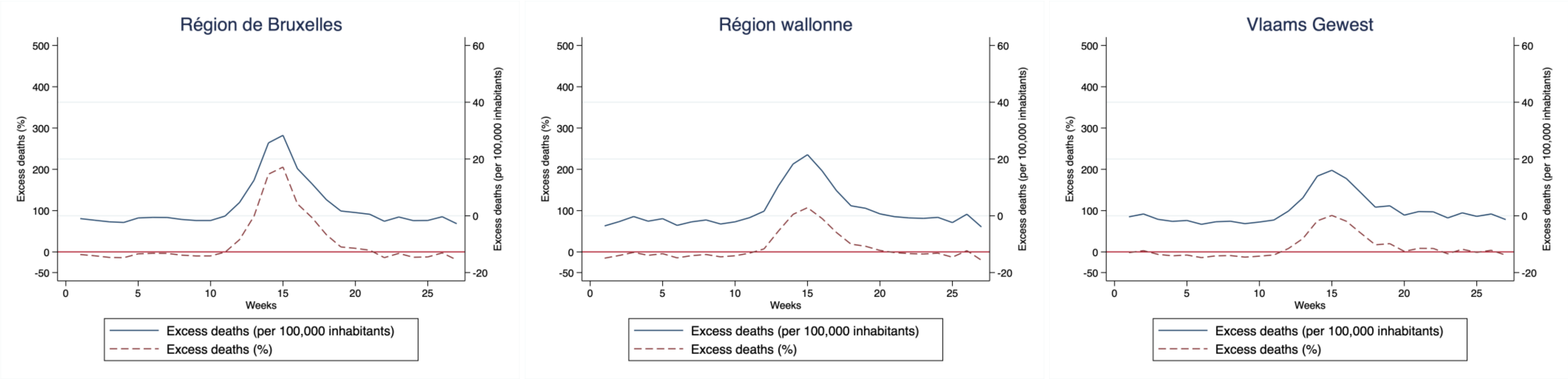
Région de Bruxelles
Région wallonne
Vlaams Gewest
Excess deaths (%)
Excess deaths (per 100,000 inhabitants)
Weeks
Excess deaths (per 100,000 inhabitants)
Excess deaths (%)

BULGARIA

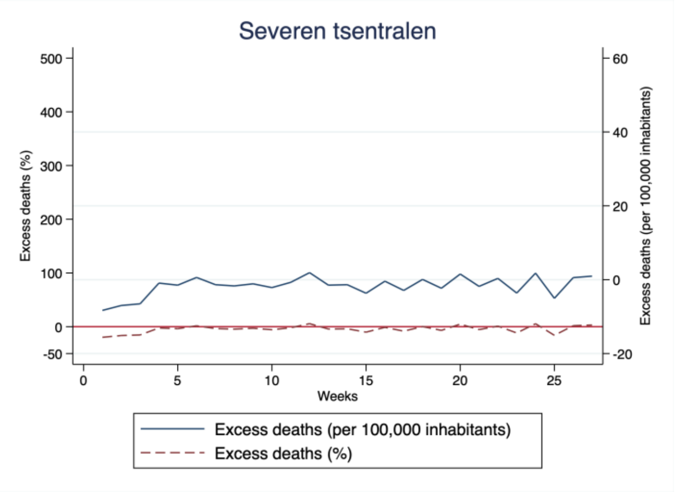
Severen tsentralen
Excess deaths (%)
500
400
300
200
100
0
-50
Excess deaths (per 100,000 inhabitants)
60
40
20
0
-20
0
5
10
15
20
25
Weeks
Excess deaths (per 100,000 inhabitants)
Excess deaths (%)

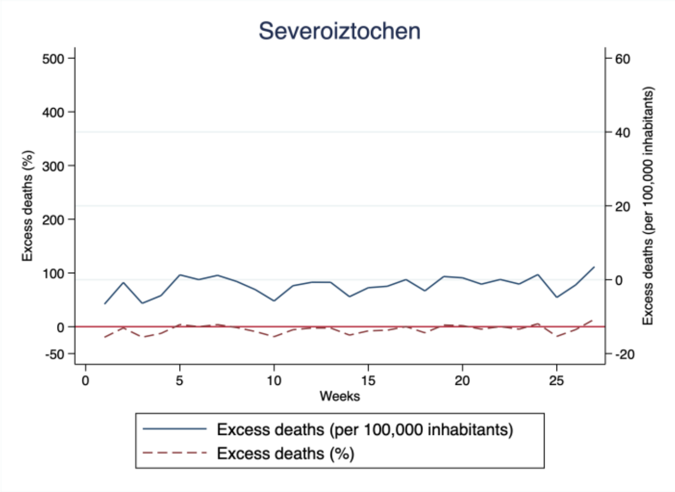
Severoiztochen
Excess deaths (%)
500
400
300
200
100
0
-50
Excess deaths (per 100,000 inhabitants)
60
40
20
0
-20
0
5
10
15
20
25
Weeks
Excess deaths (per 100,000 inhabitants)
Excess deaths (%)

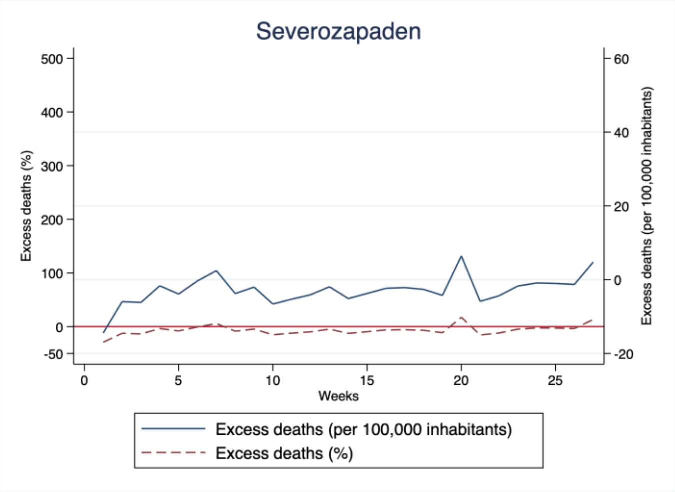
Severozapaden
Excess deaths (%)
500
400
300
200
100
0
-50
Excess deaths (per 100,000 inhabitants)
60
40
20
0
-20
0
5
10
15
20
25
Weeks
Excess deaths (per 100,000 inhabitants)
Excess deaths (%)

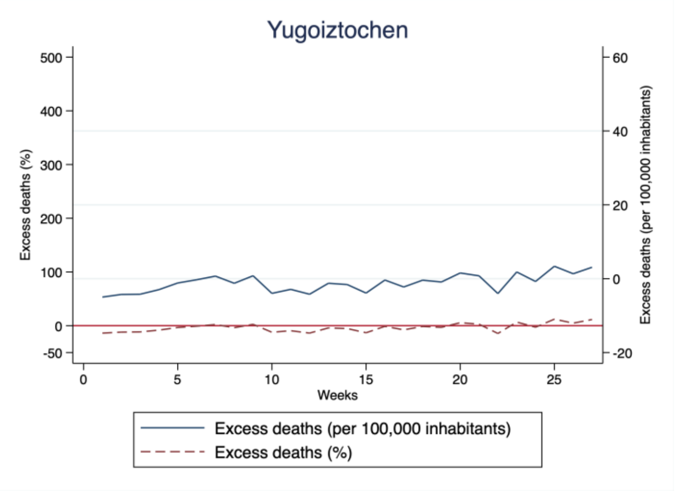
Yugoiztochen
Excess deaths (%)
500
400
300
200
100
0
-50
Excess deaths (per 100,000 inhabitants)
60
40
20
0
-20
0
5
10
15
20
25
Weeks
Excess deaths (per 100,000 inhabitants)
Excess deaths (%)

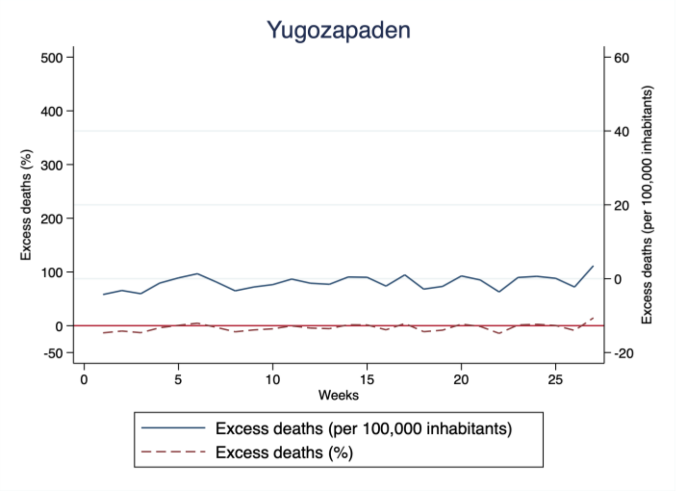
Yugozapaden
Excess deaths (%)
500
400
300
200
100
0
-50
Excess deaths (per 100,000 inhabitants)
60
40
20
0
-20
0
5
10
15
20
25
Weeks
Excess deaths (per 100,000 inhabitants)
Excess deaths (%)

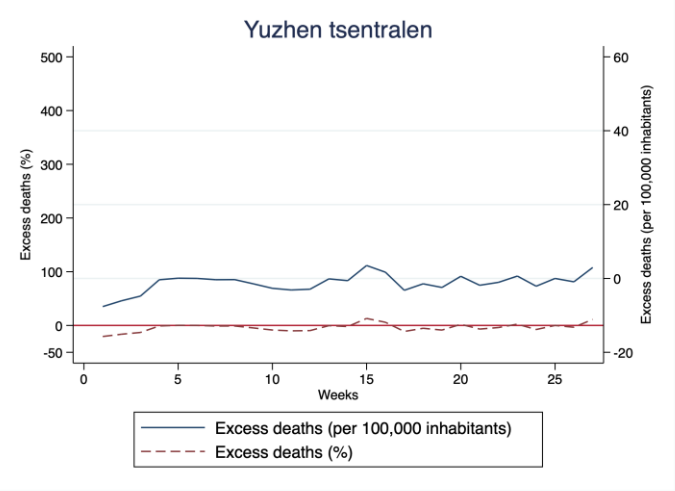
Yuzhen tsentralen
Excess deaths (%)
500
400
300
200
100
0
-50
Excess deaths (per 100,000 inhabitants)
60
40
20
0
-20
0
5
10
15
20
25
Weeks
Excess deaths (per 100,000 inhabitants)
Excess deaths (%)

CZECHIA

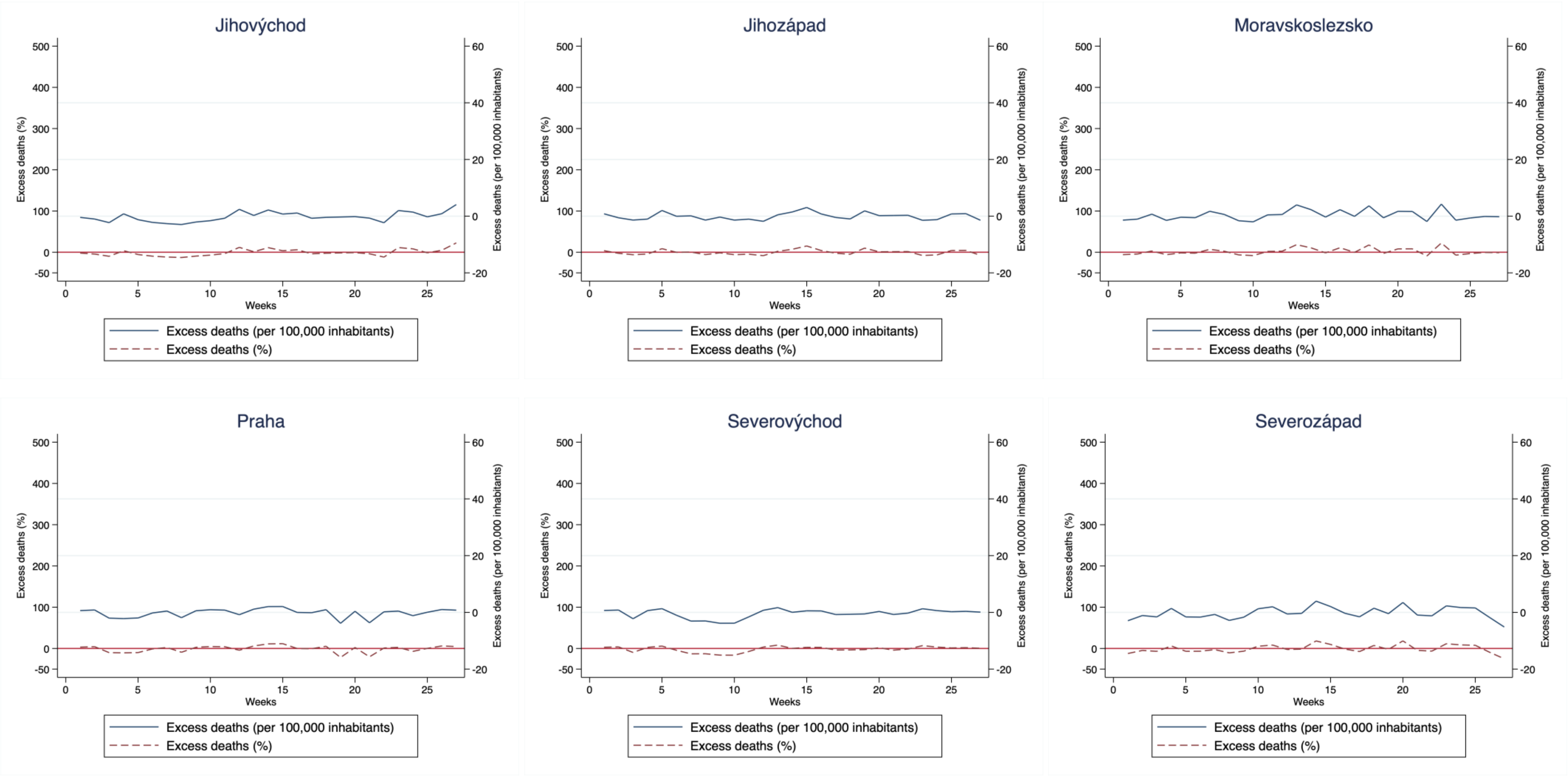
Jihovýchod
Jihozápad
Moravskoslezsko
Praha
Severovýchod
Severozápad
Excess deaths (%)
Excess deaths (per 100,000 inhabitants)
Weeks
500
400
300
200
100
0
-50
60
40
20
0
-20
0
5
10
15
20
25
Excess deaths (per 100,000 inhabitants)
Excess deaths (%)

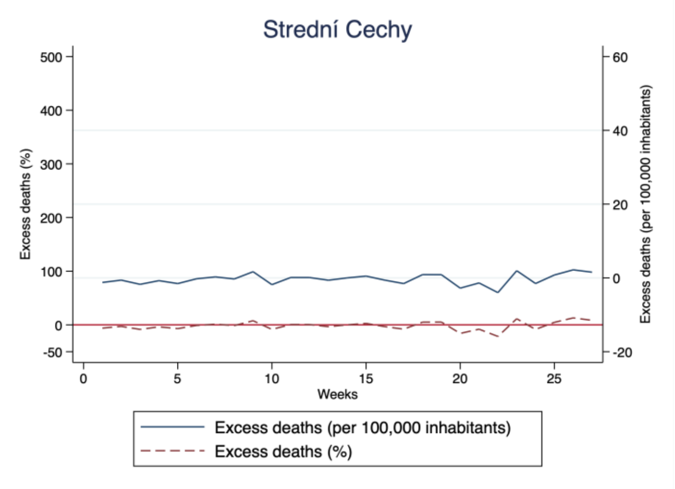

Strední Cechy
Excess deaths (%)
Excess deaths (per 100,000 inhabitants)
Weeks
Excess deaths (per 100,000 inhabitants)
Excess deaths (%)

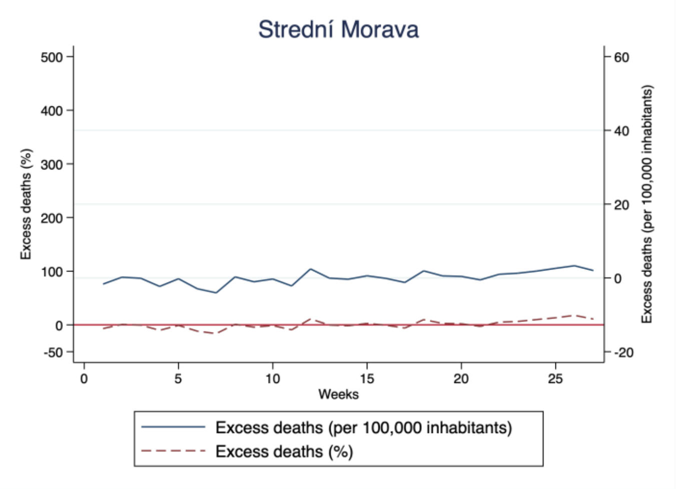

Strední Morava
Excess deaths (%)
Excess deaths (per 100,000 inhabitants)
Weeks
Excess deaths (per 100,000 inhabitants)
Excess deaths (%)

GERMANY

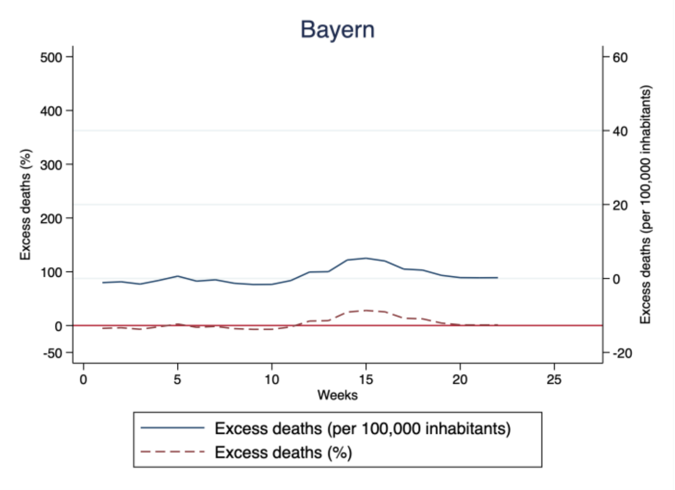

Bayern
Excess deaths (%)
Excess deaths (per 100,000 inhabitants)
Weeks
Excess deaths (per 100,000 inhabitants)
Excess deaths (%)

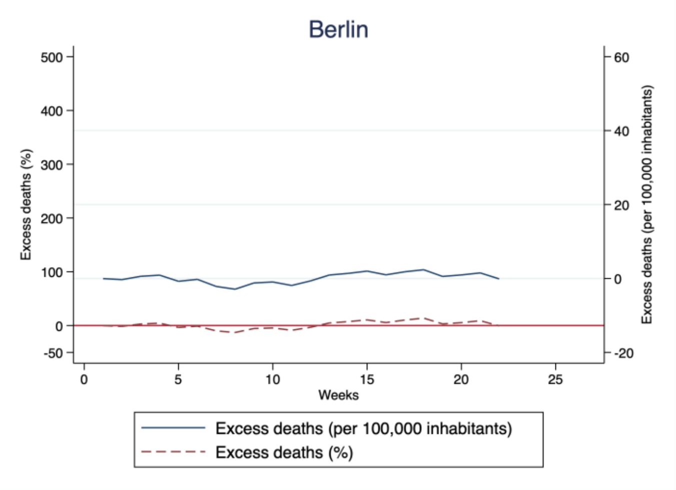

Berlin
Excess deaths (%)
Excess deaths (per 100,000 inhabitants)
Weeks
Excess deaths (per 100,000 inhabitants)
Excess deaths (%)

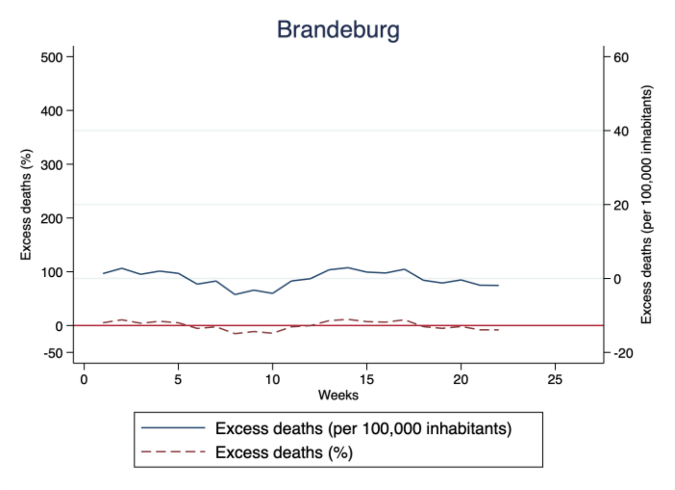

Brandeburg
Excess deaths (%)
Excess deaths (per 100,000 inhabitants)
Weeks
Excess deaths (per 100,000 inhabitants)
Excess deaths (%)

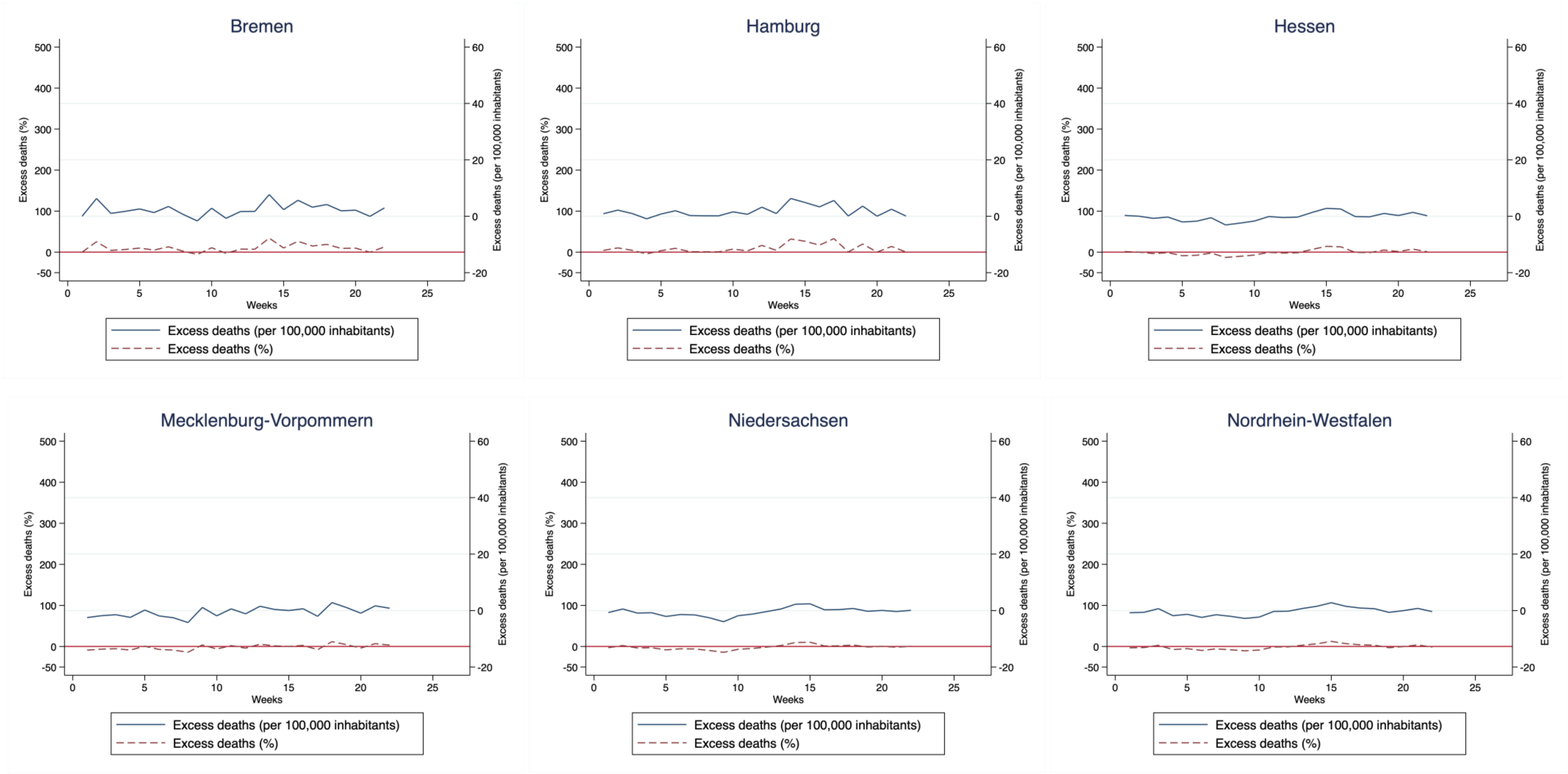
Bremen
Hamburg
Hessen
Mecklenburg-Vorpommern
Niedersachsen
Nordrhein-Westfalen
Excess deaths (%)
Excess deaths (per 100,000 inhabitants)
Weeks
Excess deaths (per 100,000 inhabitants)
Excess deaths (%)

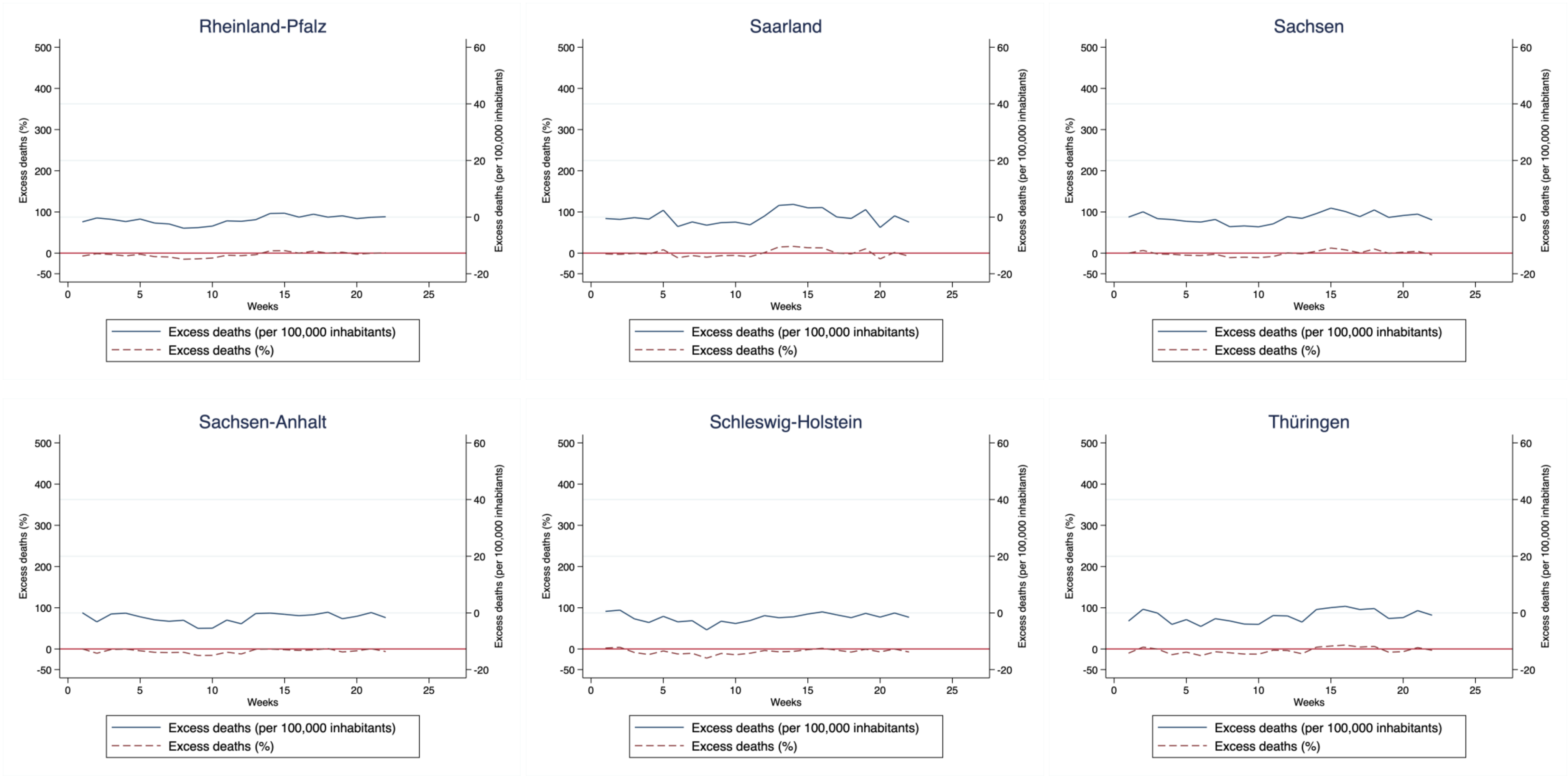
Rheinland-Pfalz
Saarland
Sachsen
Sachsen-Anhalt
Schleswig-Holstein
Thüringen
Excess deaths (%)
Excess deaths (per 100,000 inhabitants)
Weeks
500
400
300
200
100
0
-50
60
40
20
0
-20
0
5
10
15
20
25
Excess deaths (per 100,000 inhabitants)
Excess deaths (%)

DENMARK

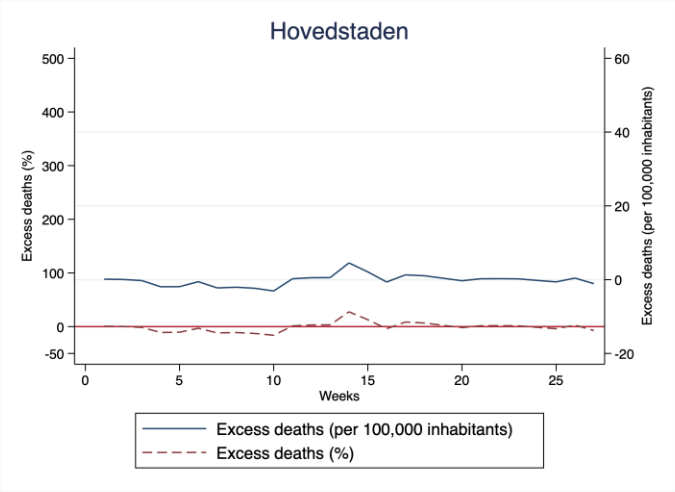
Hovedstaden
Excess deaths (%)
Excess deaths (per 100,000 inhabitants)
Weeks
500
400
300
200
100
0
-50
60
40
20
0
-20
0 5 10 15 20 25
Excess deaths (per 100,000 inhabitants)
Excess deaths (%)

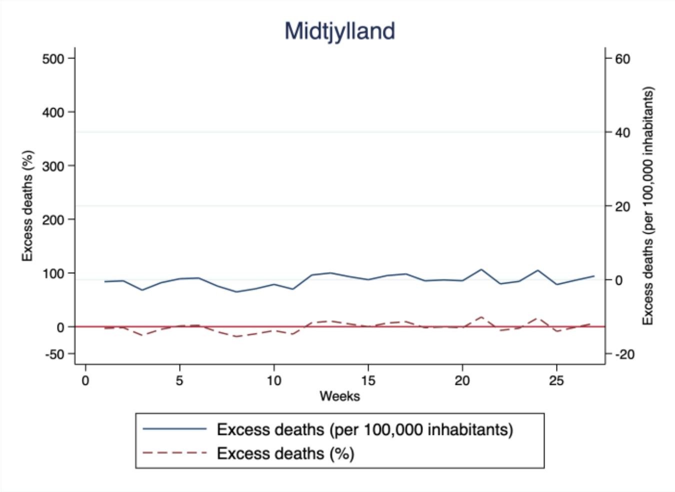
Midtjylland
Excess deaths (%)
Excess deaths (per 100,000 inhabitants)
Weeks
500
400
300
200
100
0
-50
60
40
20
0
-20
0 5 10 15 20 25
Excess deaths (per 100,000 inhabitants)
Excess deaths (%)

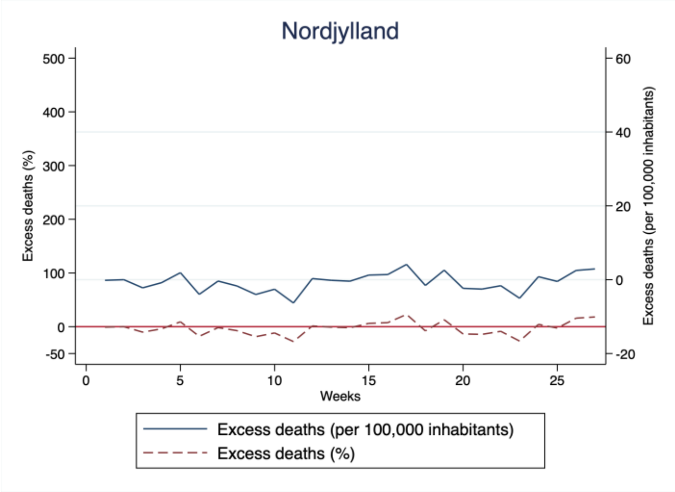
Nordjylland
Excess deaths (%)
Excess deaths (per 100,000 inhabitants)
Weeks
500
400
300
200
100
0
-50
60
40
20
0
-20
0 5 10 15 20 25
Excess deaths (per 100,000 inhabitants)
Excess deaths (%)

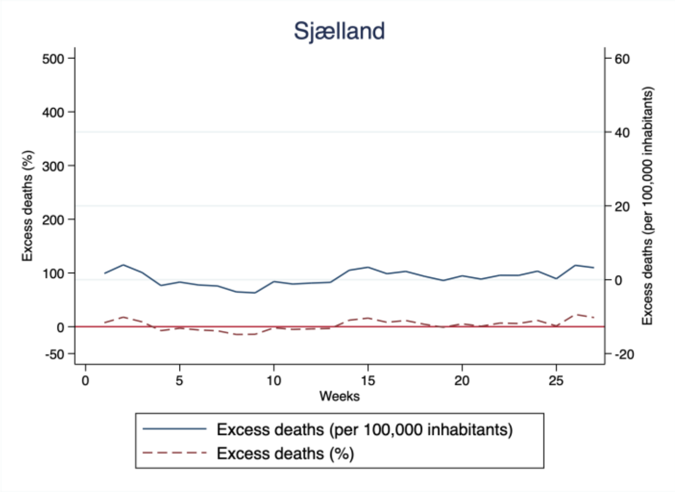
Sjælland
Excess deaths (%)
Excess deaths (per 100,000 inhabitants)
Weeks
500
400
300
200
100
0
-50
60
40
20
0
-20
0 5 10 15 20 25
Excess deaths (per 100,000 inhabitants)
Excess deaths (%)

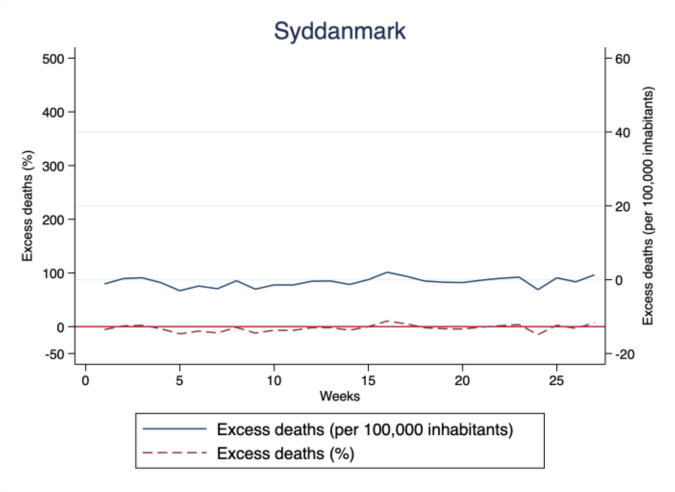
Syddanmark
Excess deaths (%)
Excess deaths (per 100,000 inhabitants)
Weeks
500
400
300
200
100
0
-50
60
40
20
0
-20
0 5 10 15 20 25
Excess deaths (per 100,000 inhabitants)
Excess deaths (%)

ESTONIA

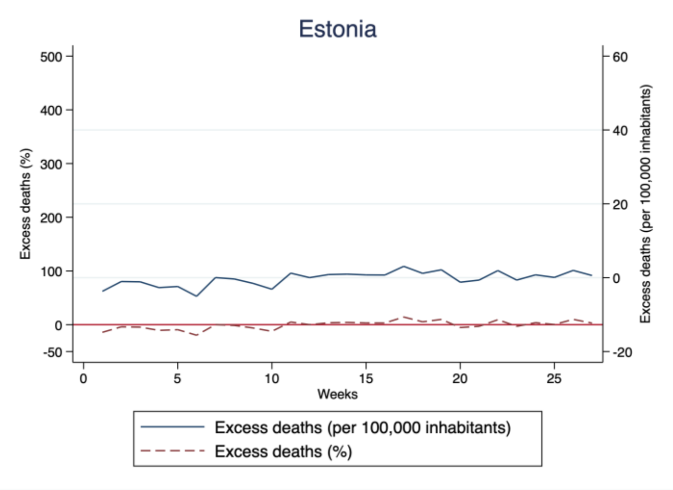

FINLAND

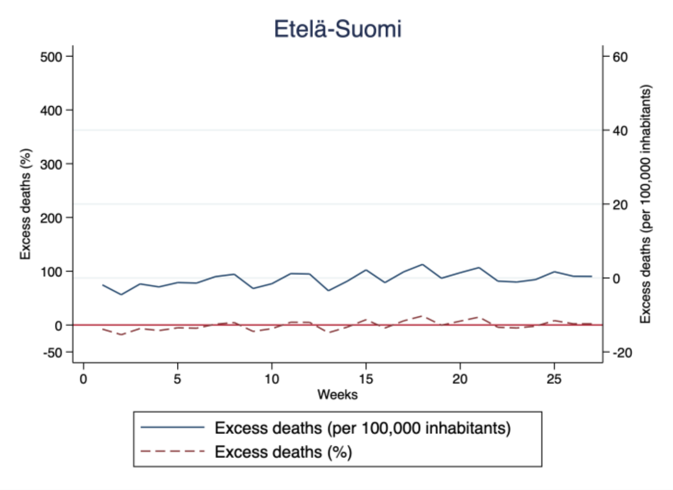

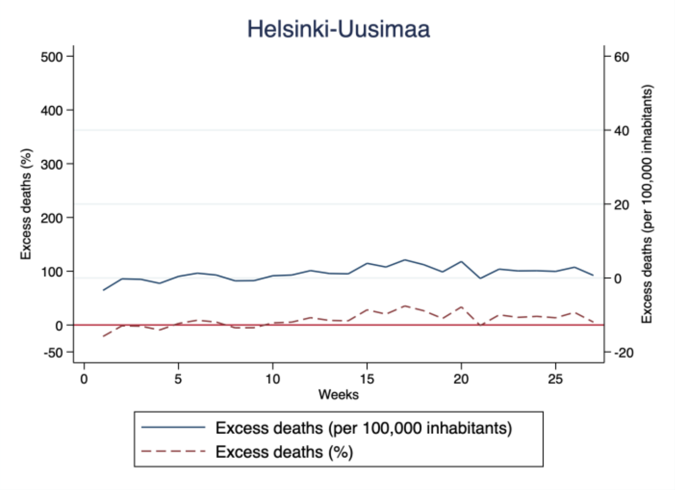

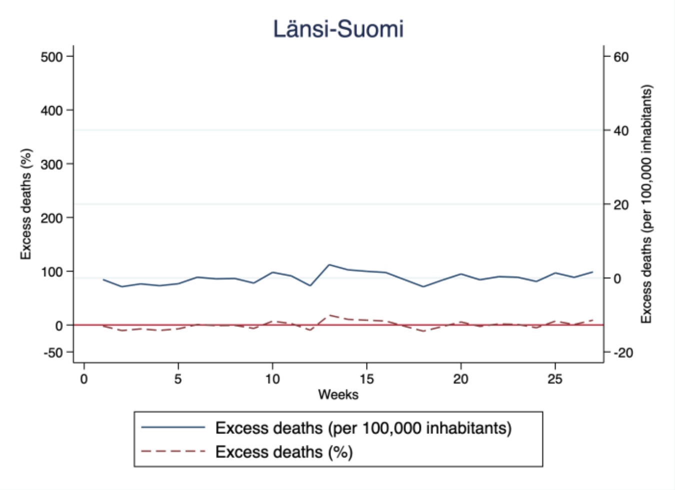

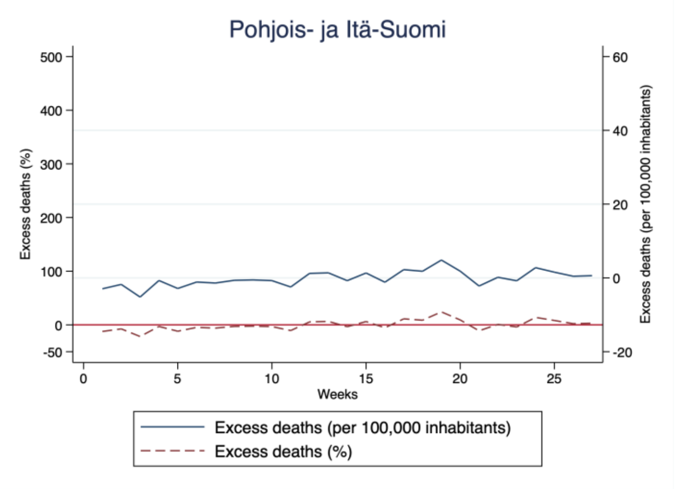
Pohjois- ja Itä-Suomi
Excess deaths (%)
Excess deaths (per 100,000 inhabitants)
Weeks
Excess deaths (per 100,000 inhabitants)
Excess deaths (%)

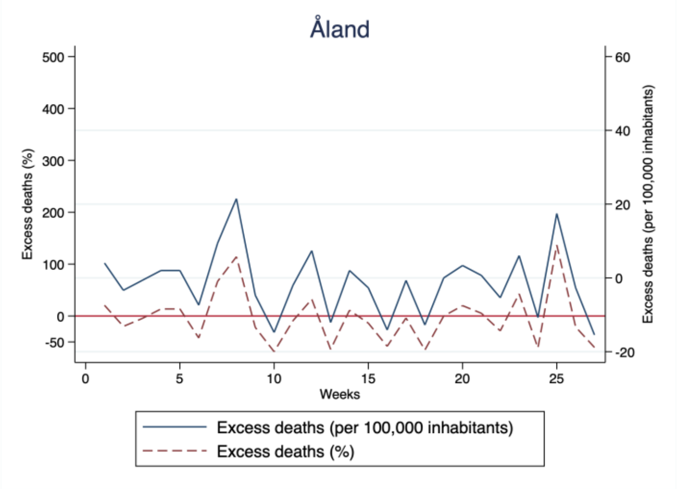
Åland
Excess deaths (%)
Excess deaths (per 100,000 inhabitants)
Weeks
Excess deaths (per 100,000 inhabitants)
Excess deaths (%)

FRANCE

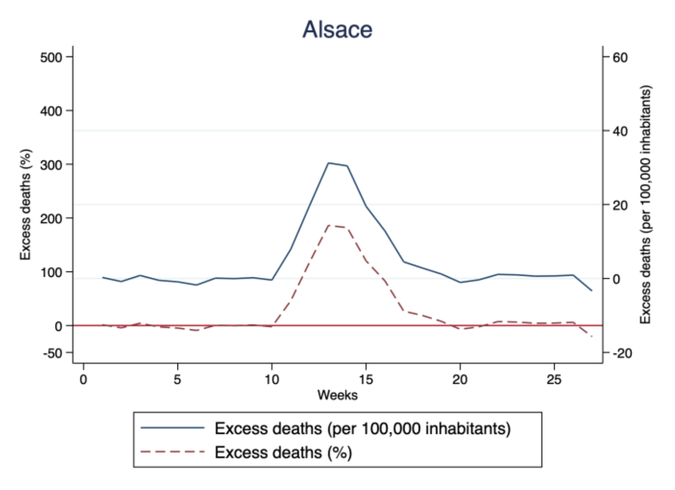
Alsace
Excess deaths (%)
Excess deaths (per 100,000 inhabitants)
Weeks
Excess deaths (per 100,000 inhabitants)
Excess deaths (%)

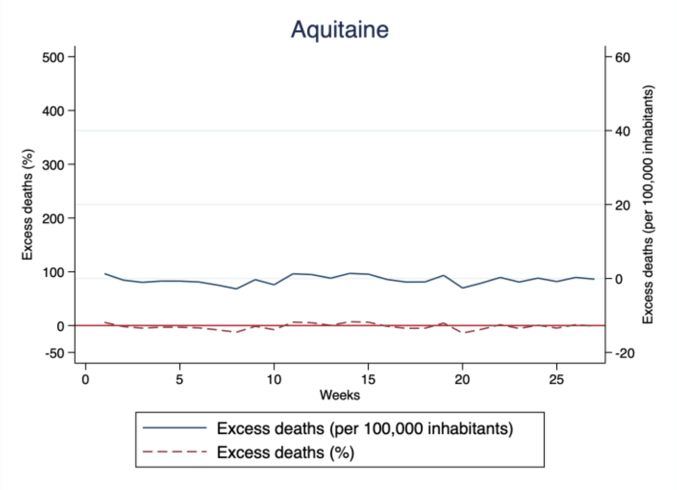
Aquitaine
Excess deaths (%)
Excess deaths (per 100,000 inhabitants)
Weeks
Excess deaths (per 100,000 inhabitants)
Excess deaths (%)

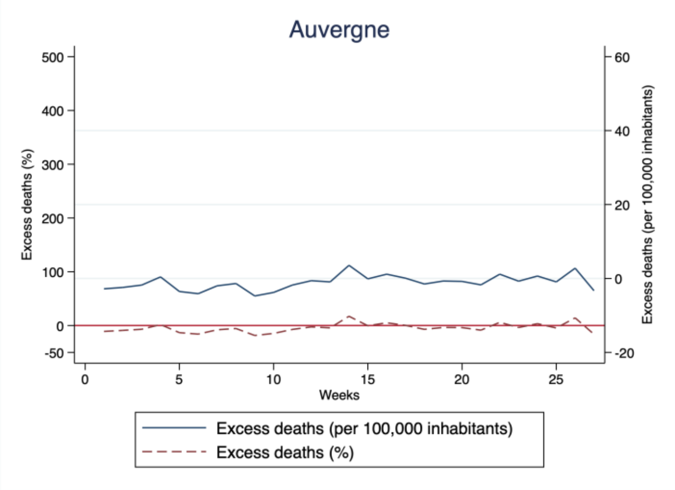
Auvergne
Excess deaths (%)
Excess deaths (per 100,000 inhabitants)
Weeks
Excess deaths (per 100,000 inhabitants)
Excess deaths (%)

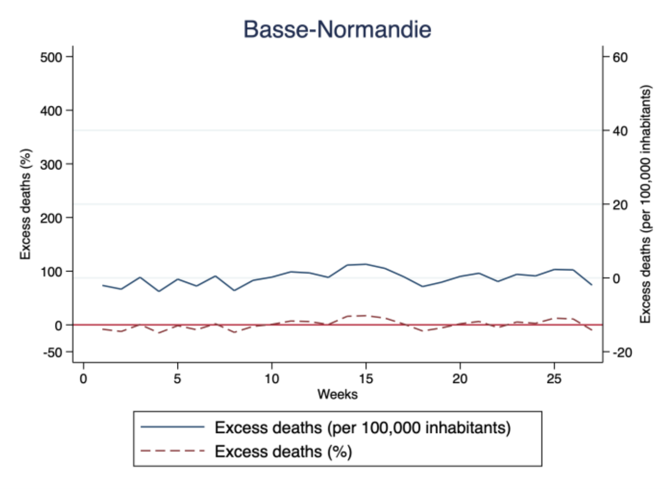
Basse-Normandie
Excess deaths (%)
Excess deaths (per 100,000 inhabitants)
Weeks
Excess deaths (per 100,000 inhabitants)
Excess deaths (%)

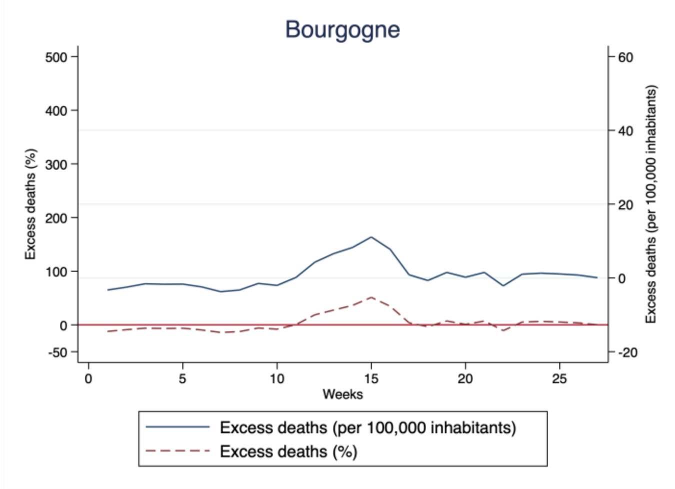
Bourgogne
Excess deaths (%)
Excess deaths (per 100,000 inhabitants)
Weeks
Excess deaths (per 100,000 inhabitants)
Excess deaths (%)

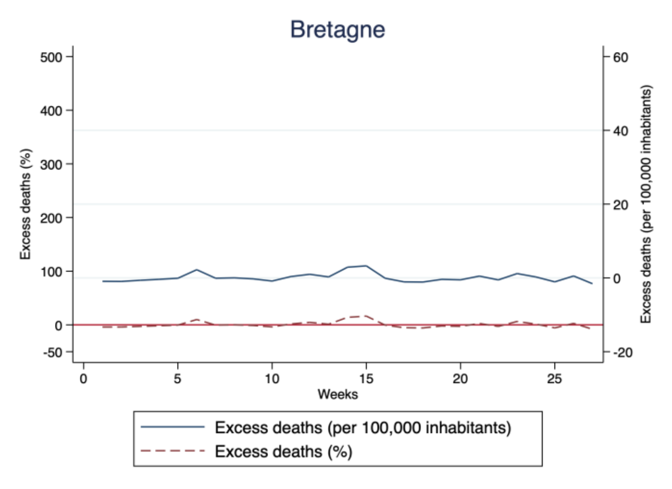
Bretagne
Excess deaths (%)
Excess deaths (per 100,000 inhabitants)
Weeks
Excess deaths (per 100,000 inhabitants)
Excess deaths (%)

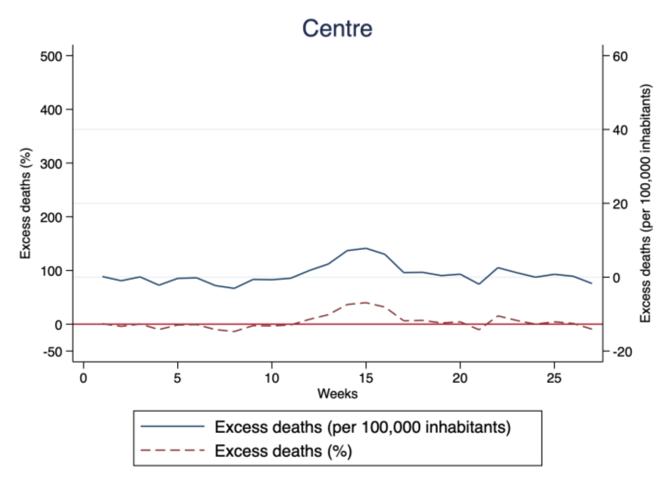
Centre
Excess deaths (%)
Excess deaths (per 100,000 inhabitants)
Weeks
Excess deaths (per 100,000 inhabitants)
Excess deaths (%)

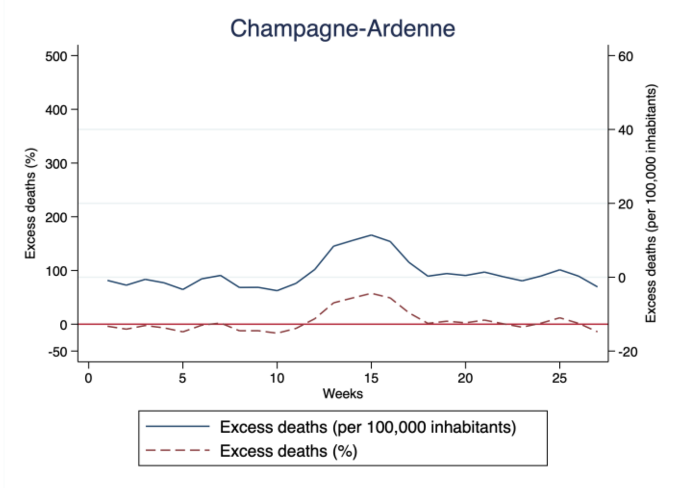
Champagne-Ardenne
Excess deaths (%)
Excess deaths (per 100,000 inhabitants)
Weeks
Excess deaths (per 100,000 inhabitants)
Excess deaths (%)

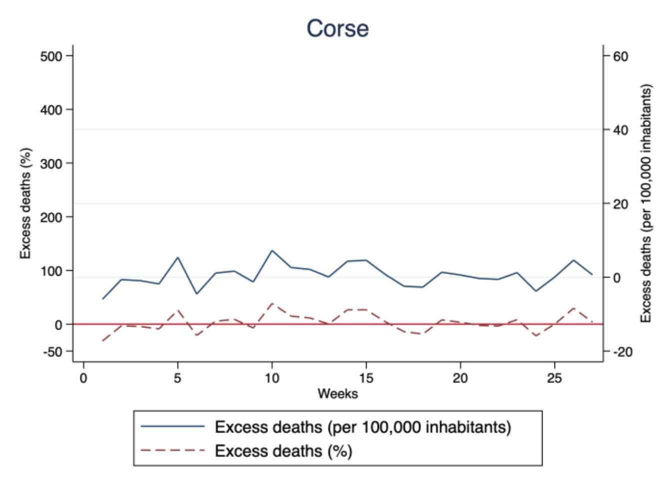
Corse
Excess deaths (%)
Excess deaths (per 100,000 inhabitants)
Weeks
Excess deaths (per 100,000 inhabitants)
Excess deaths (%)

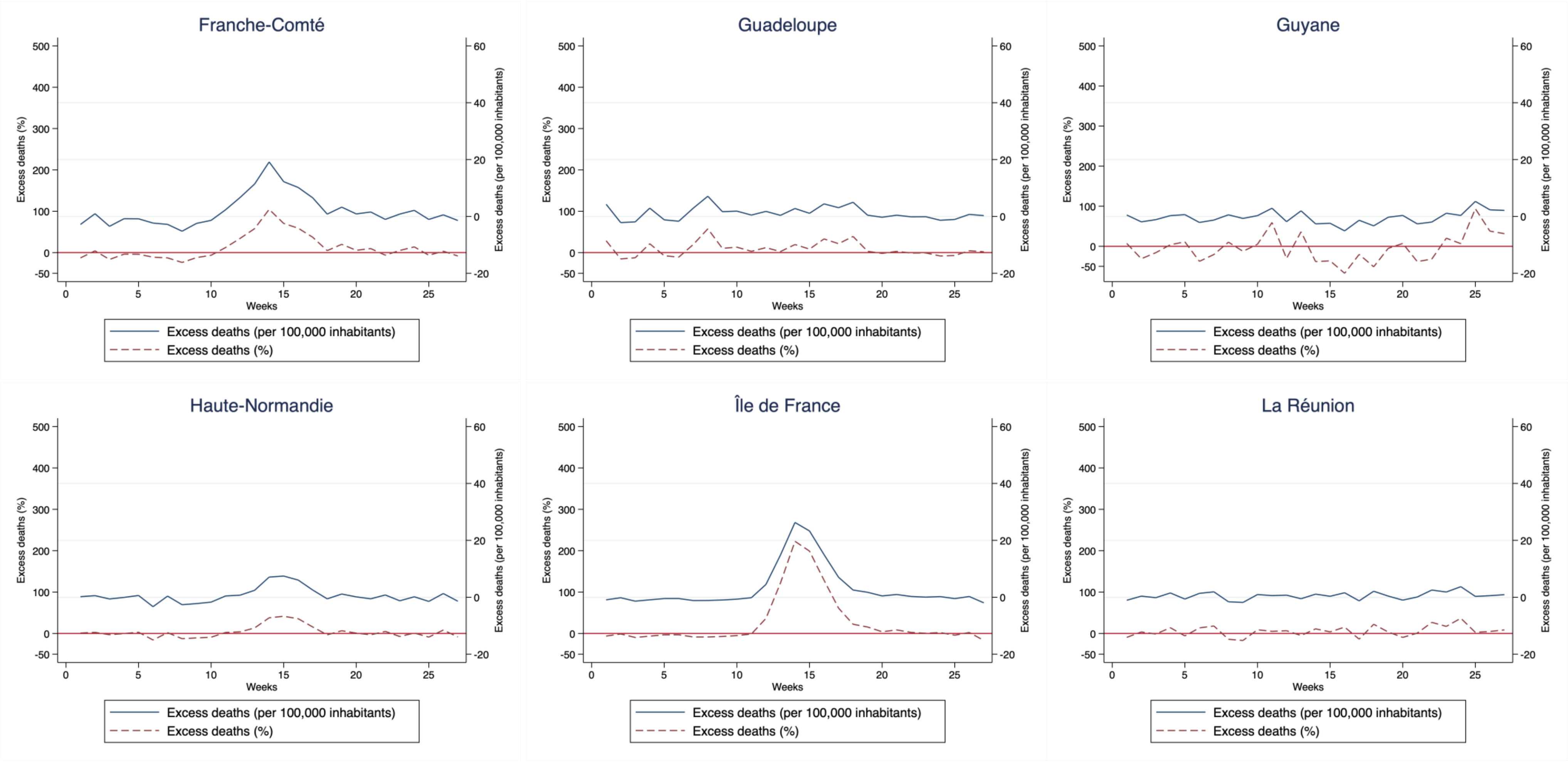
Franche-Comté
Guadeloupe
Guyane
Haute-Normandie
Île de France
La Réunion
Excess deaths (%)
Excess deaths (per 100,000 inhabitants)
Weeks
500
400
300
200
100
0
-50
60
40
20
0
-20
0
5
10
15
20
25
Excess deaths (per 100,000 inhabitants)
Excess deaths (%)

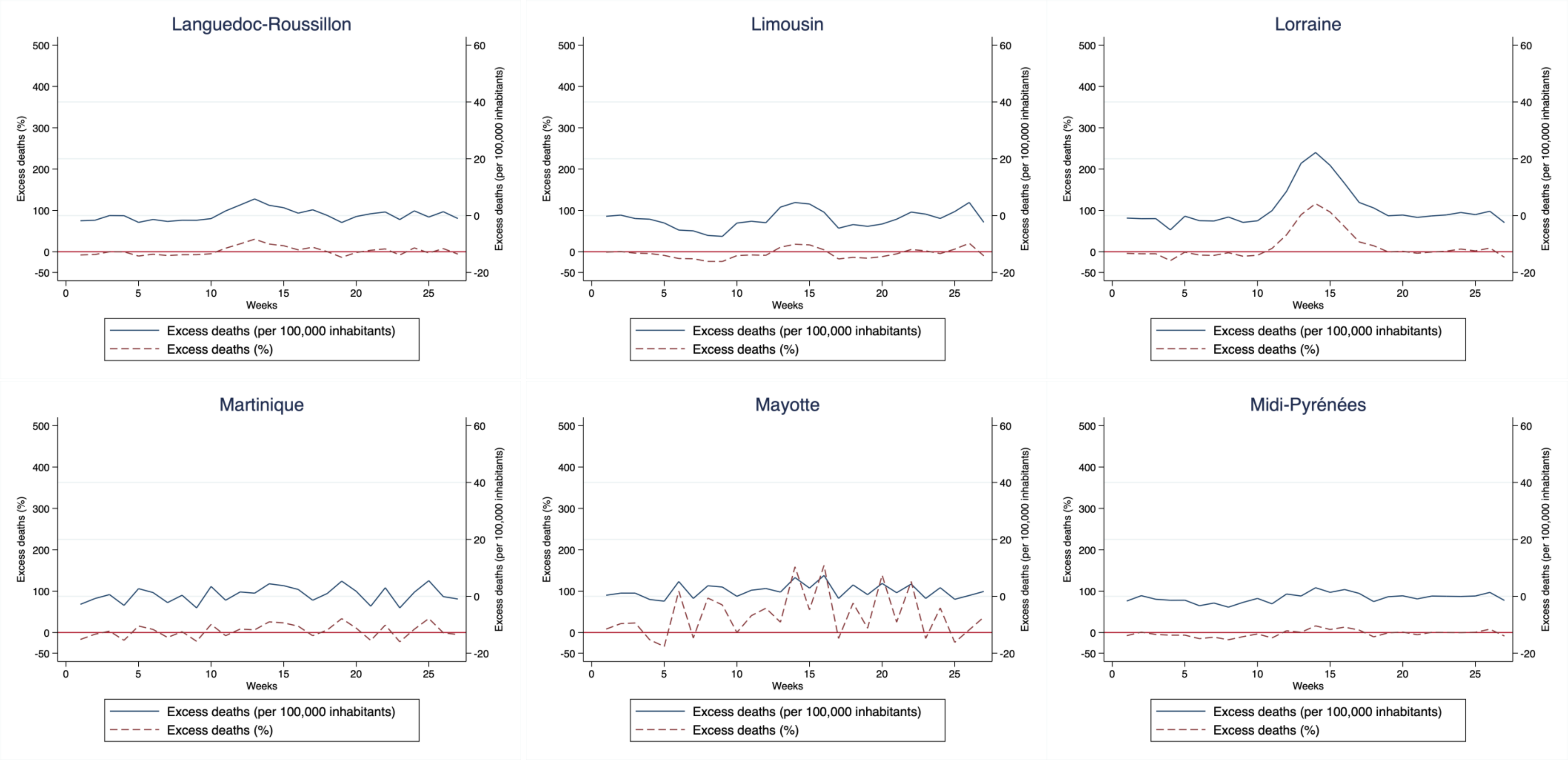
Languedoc-Roussillon
Limousin
Lorraine
Martinique
Mayotte
Midi-Pyrénées
Excess deaths (%)
Excess deaths (per 100,000 inhabitants)
Weeks
Excess deaths (per 100,000 inhabitants)
Excess deaths (%)

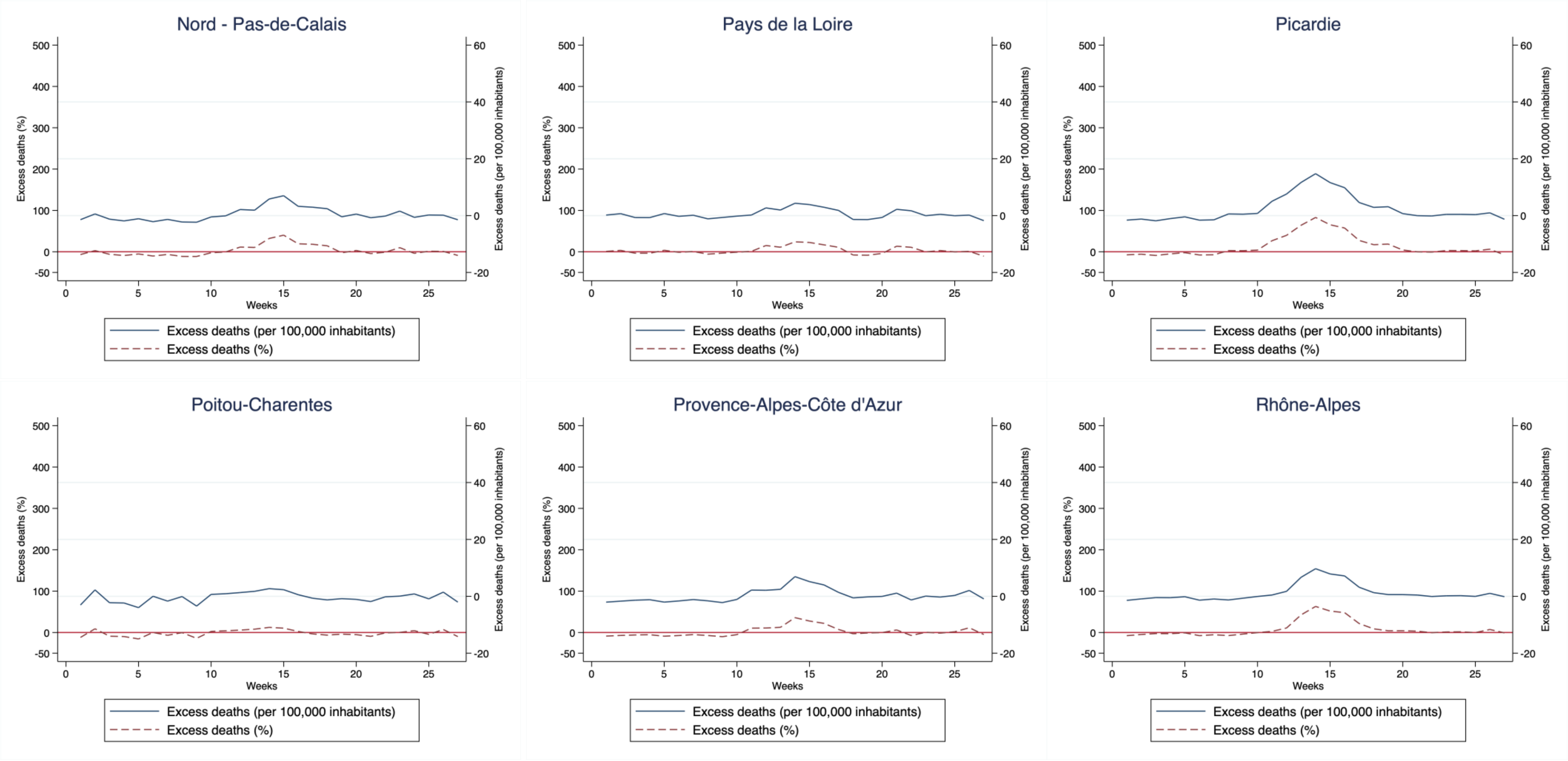
Nord - Pas-de-Calais
Pays de la Loire
Picardie
Poitou-Charentes
Provence-Alpes-Côte d'Azur
Rhône-Alpes
Excess deaths (%)
Excess deaths (per 100,000 inhabitants)
Weeks
Excess deaths (per 100,000 inhabitants)
Excess deaths (%)

HUNGARY

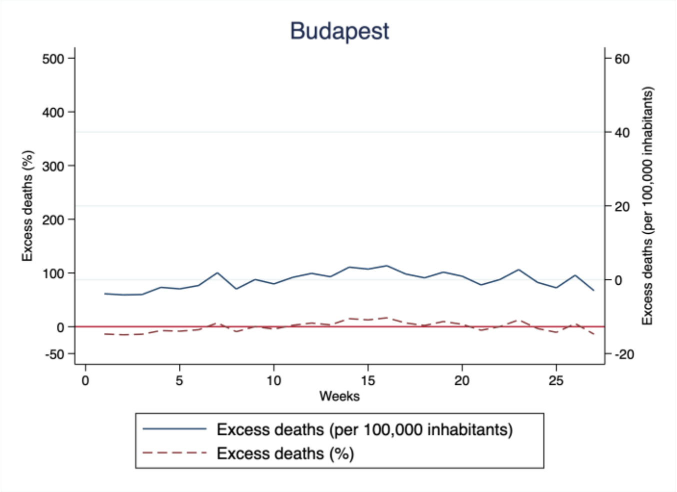

Budapest
Excess deaths (%)
Excess deaths (per 100,000 inhabitants)
Weeks
Excess deaths (per 100,000 inhabitants)
Excess deaths (%)

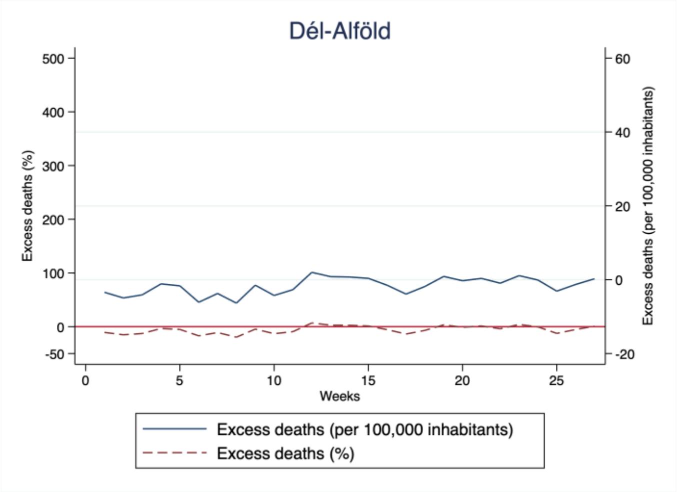

Dél-Alföld
Excess deaths (%)
Excess deaths (per 100,000 inhabitants)
Weeks
Excess deaths (per 100,000 inhabitants)
Excess deaths (%)

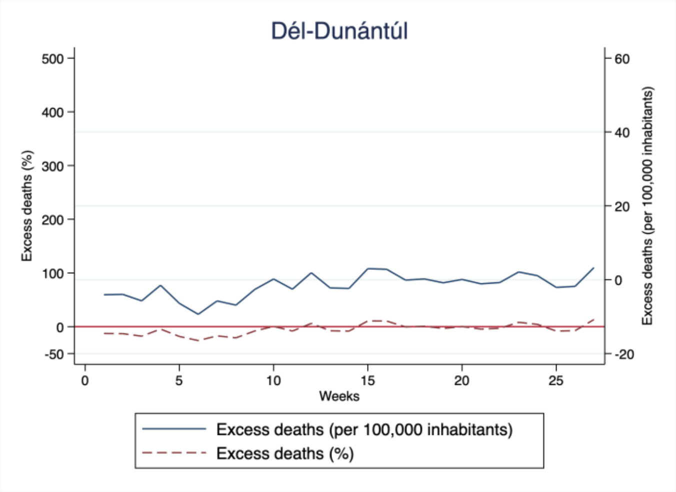

Dél-Dunántúl
Excess deaths (%)
Excess deaths (per 100,000 inhabitants)
Weeks
Excess deaths (per 100,000 inhabitants)
Excess deaths (%)

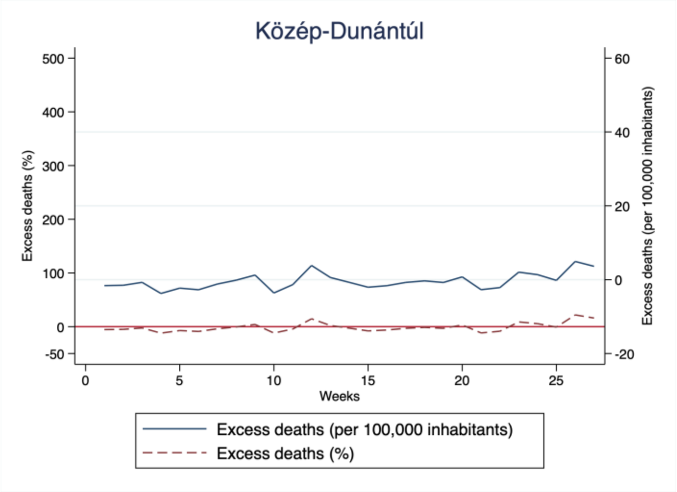

Közép-Dunántúl
Excess deaths (%)
Excess deaths (per 100,000 inhabitants)
Weeks
Excess deaths (per 100,000 inhabitants)
Excess deaths (%)

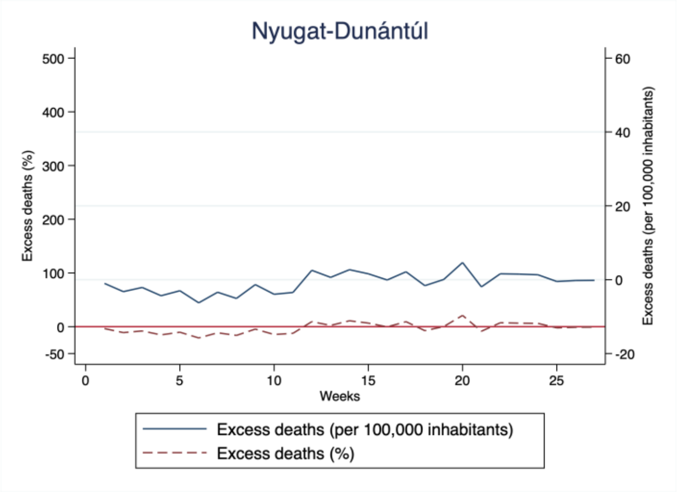

Nyugat-Dunántúl
Excess deaths (%)
Excess deaths (per 100,000 inhabitants)
Weeks
Excess deaths (per 100,000 inhabitants)
Excess deaths (%)

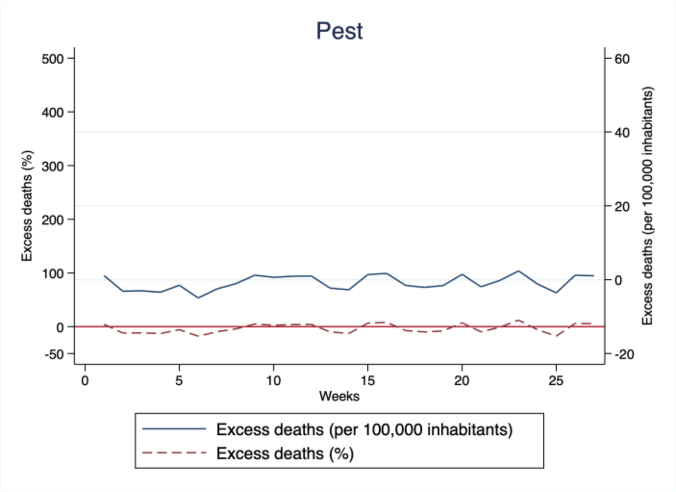

Pest
Excess deaths (%)
Excess deaths (per 100,000 inhabitants)
Weeks
Excess deaths (per 100,000 inhabitants)
Excess deaths (%)

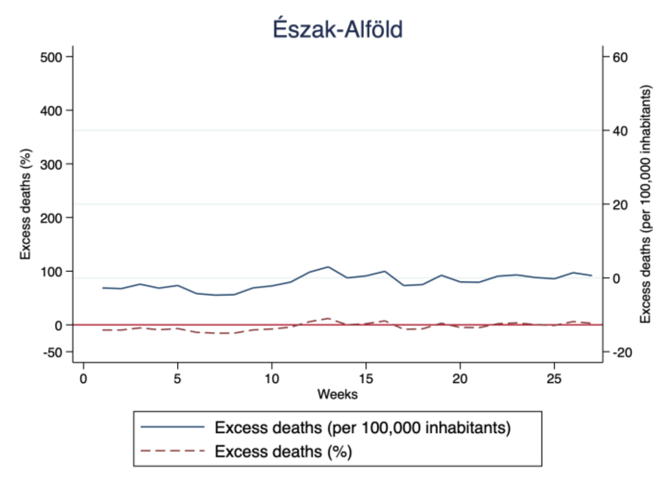
Észak-Alföld
Excess deaths (%)
Excess deaths (per 100,000 inhabitants)
Weeks
Excess deaths (per 100,000 inhabitants)
Excess deaths (%)

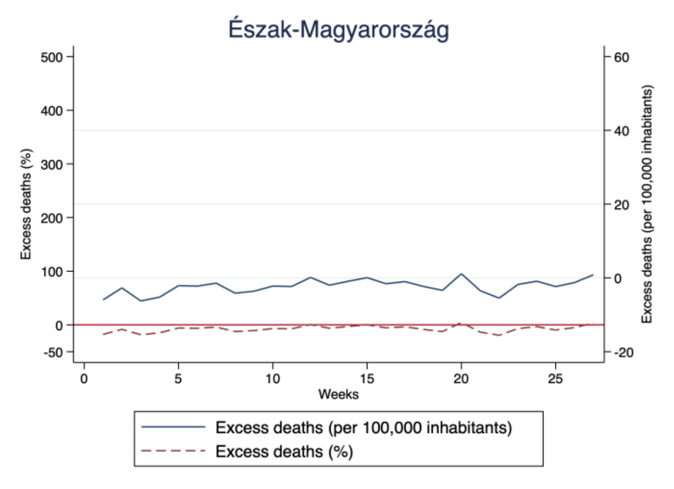
Észak-Magyarország
Excess deaths (%)
Excess deaths (per 100,000 inhabitants)
Weeks
Excess deaths (per 100,000 inhabitants)
Excess deaths (%)

ITALY

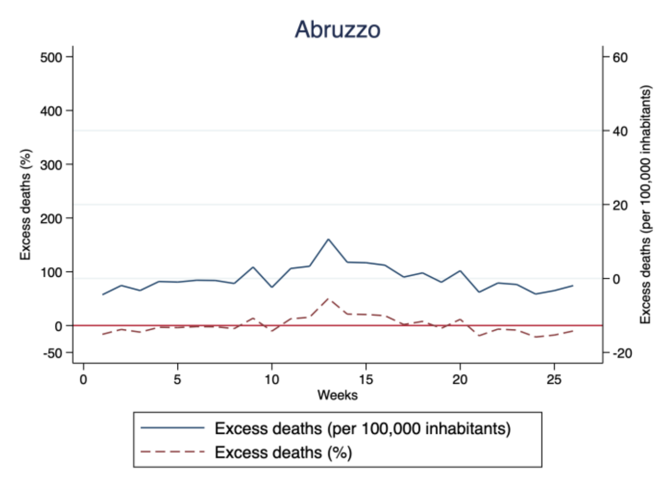
Abruzzo
Excess deaths (%)
Excess deaths (per 100,000 inhabitants)
Weeks
Excess deaths (per 100,000 inhabitants)
Excess deaths (%)

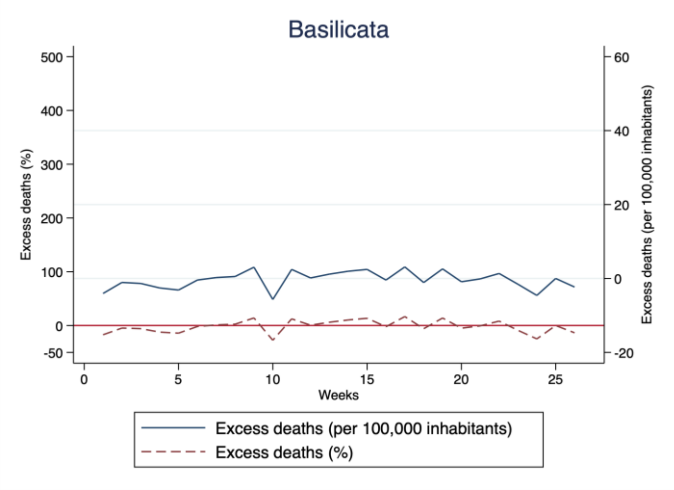
Basilicata
Excess deaths (%)
Excess deaths (per 100,000 inhabitants)
Weeks
Excess deaths (per 100,000 inhabitants)
Excess deaths (%)

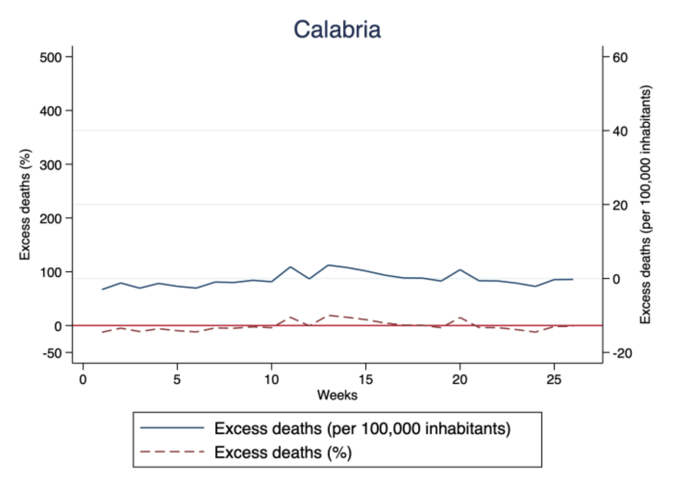
Calabria
Excess deaths (%)
Excess deaths (per 100,000 inhabitants)
Weeks
Excess deaths (per 100,000 inhabitants)
Excess deaths (%)

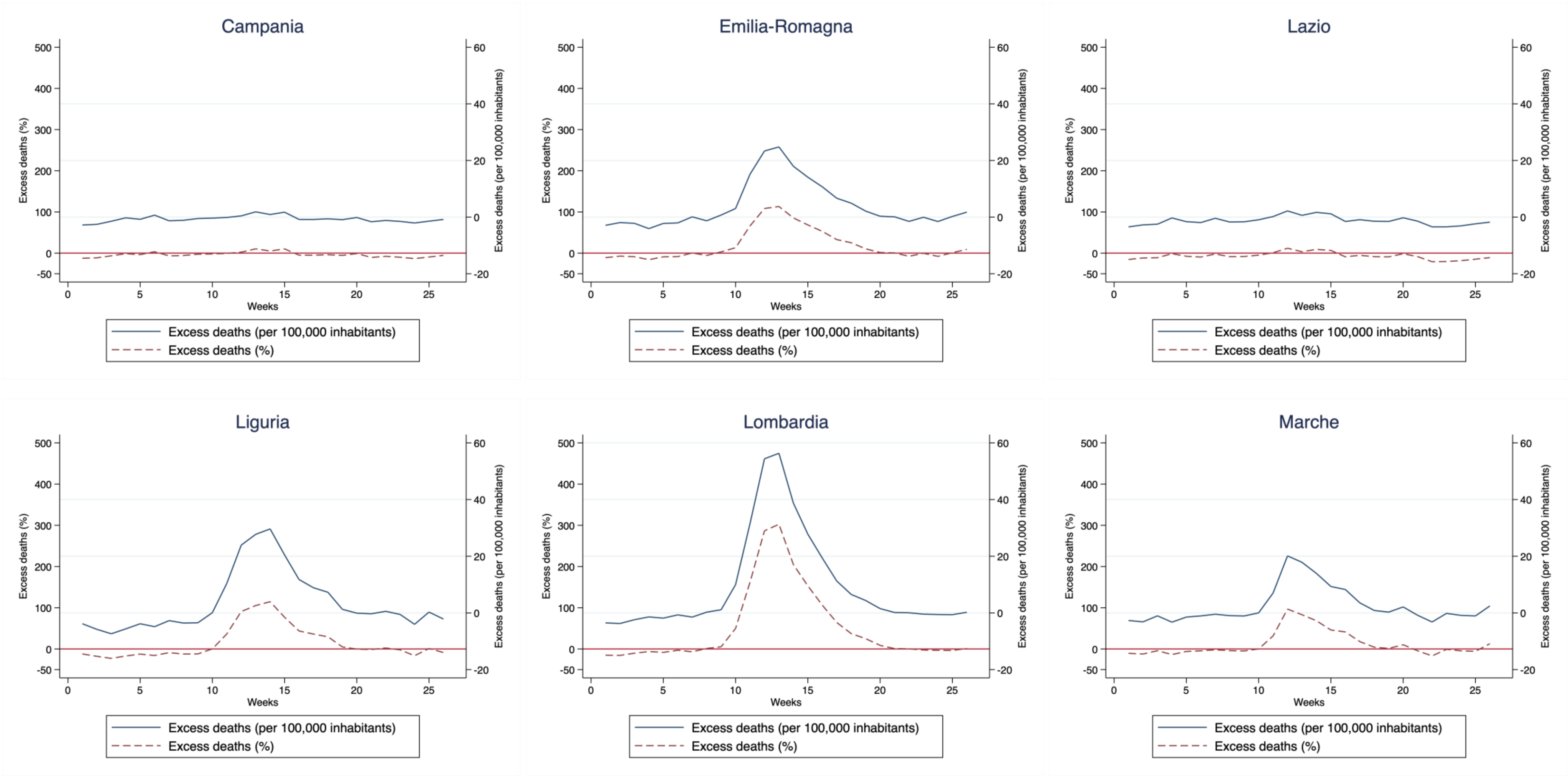

Campania
Emilia-Romagna
Lazio
Liguria
Lombardia
Marche
Excess deaths (%)
Excess deaths (per 100,000 inhabitants)
Weeks
Excess deaths (per 100,000 inhabitants)
Excess deaths (%)

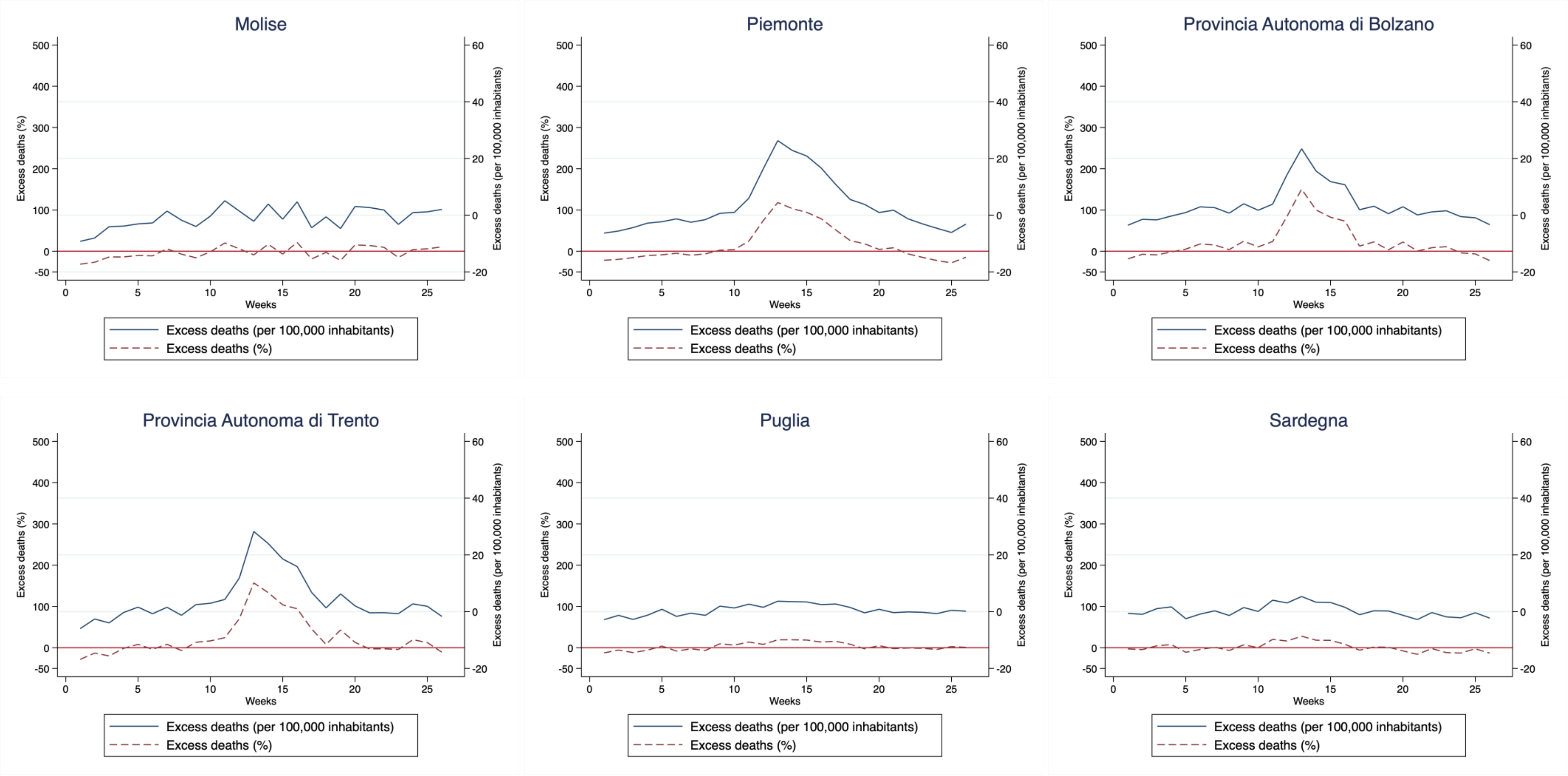
Molise
Piemonte
Provincia Autonoma di Bolzano
Provincia Autonoma di Trento
Puglia
Sardegna
Excess deaths (%)
Excess deaths (per 100,000 inhabitants)
Weeks
Excess deaths (per 100,000 inhabitants)
Excess deaths (%)

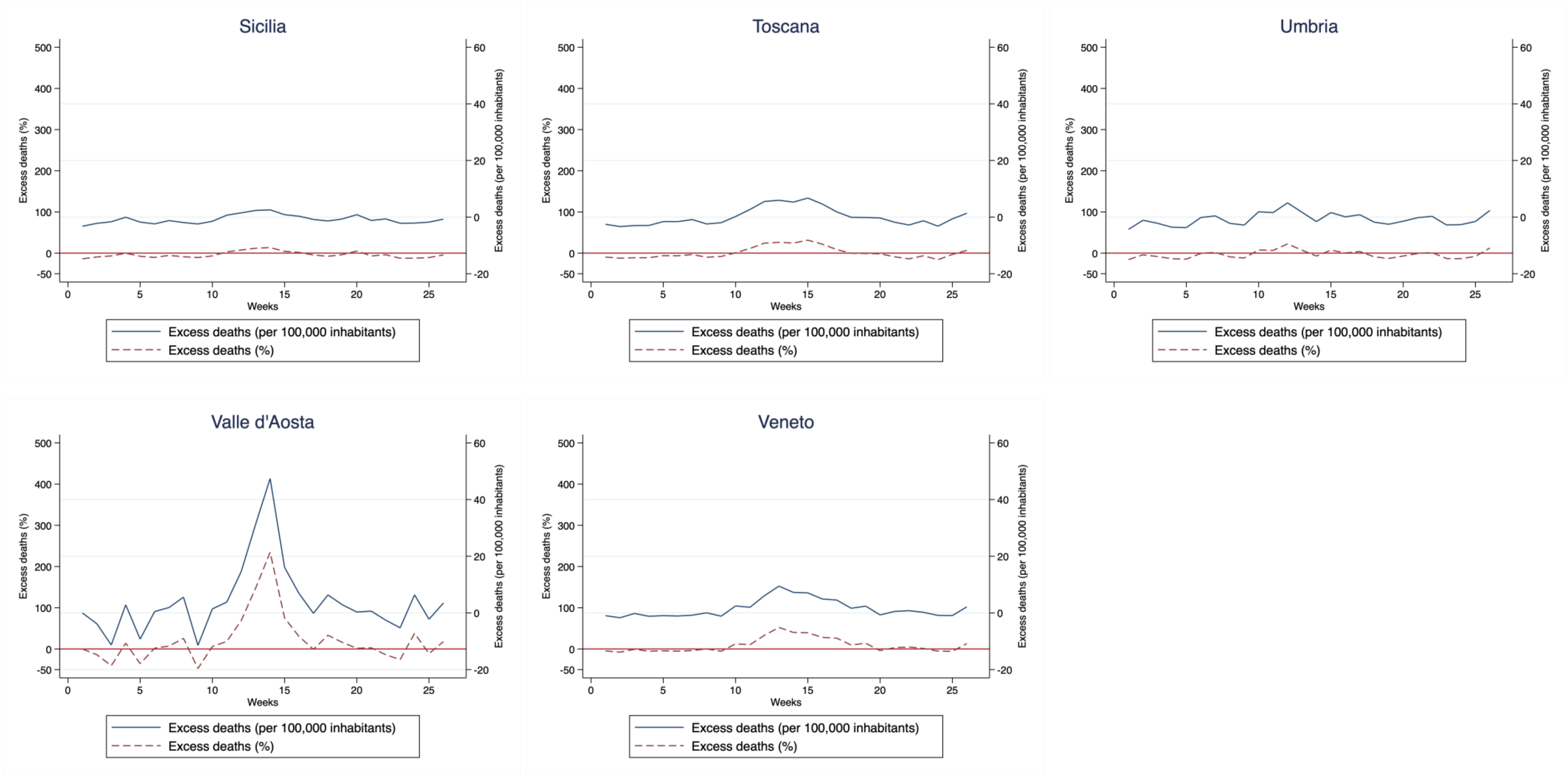
Sicilia
Toscana
Umbria
Valle d'Aosta
Veneto
Excess deaths (%)
Excess deaths (per 100,000 inhabitants)
Weeks
Excess deaths (per 100,000 inhabitants)
Excess deaths (%)

LITHUANIA

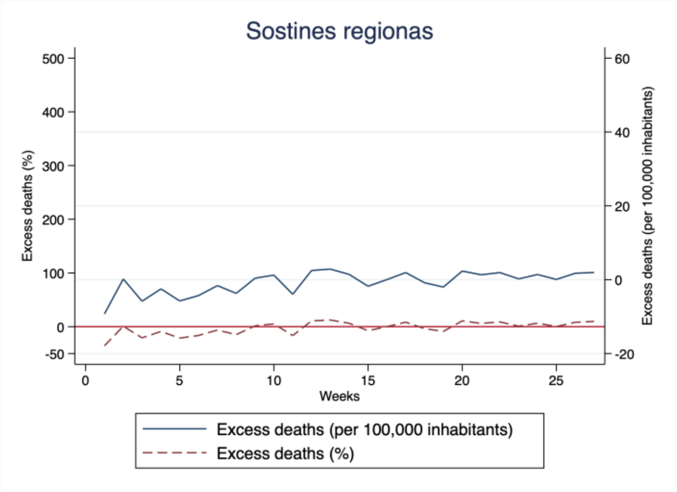

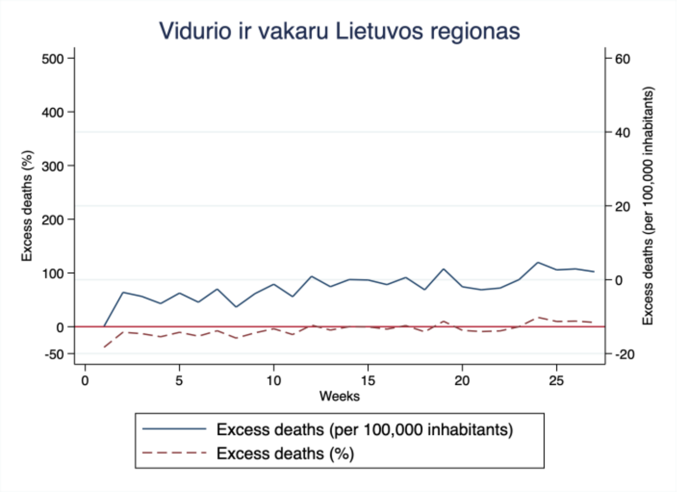

LUXEMBOURG

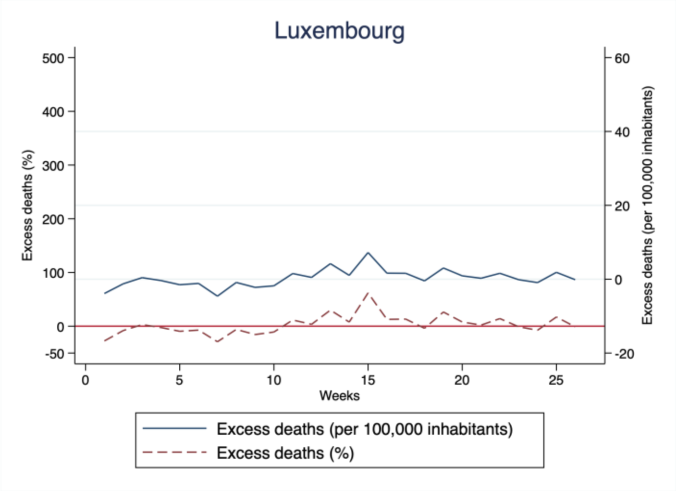

LATVIA

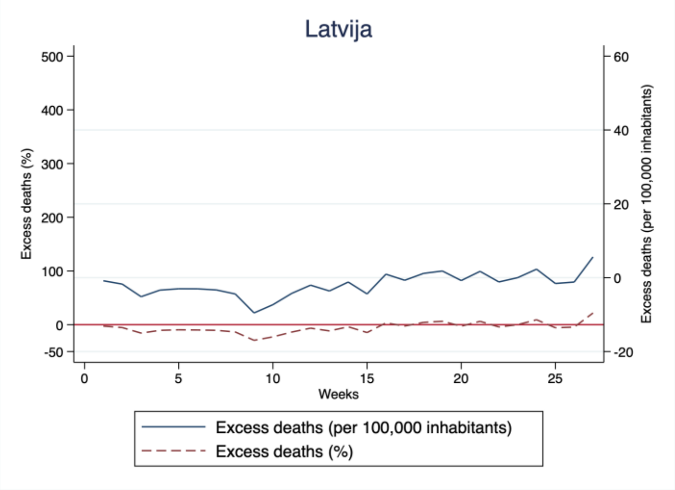

NETHERLANDS

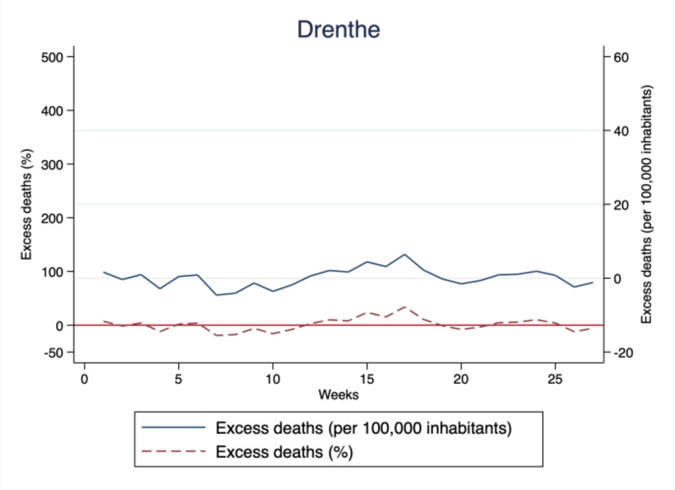

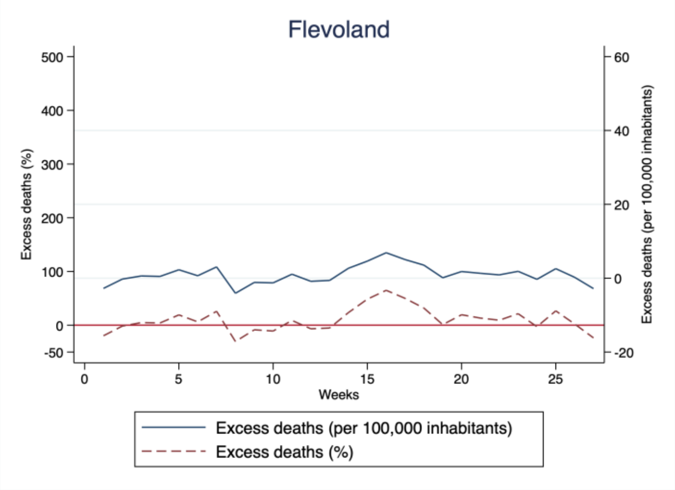

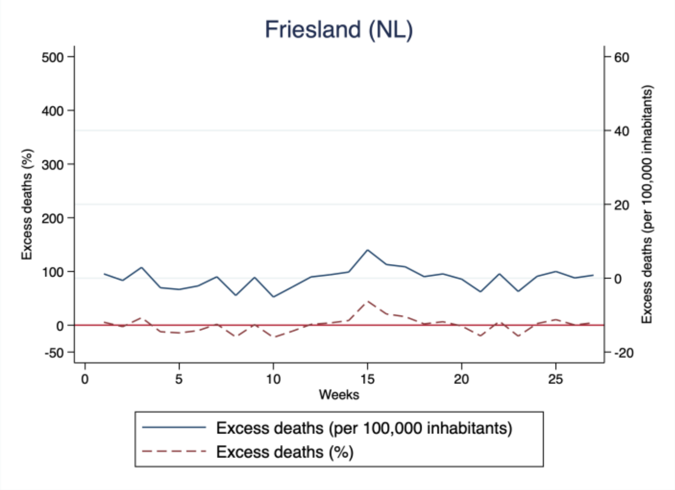

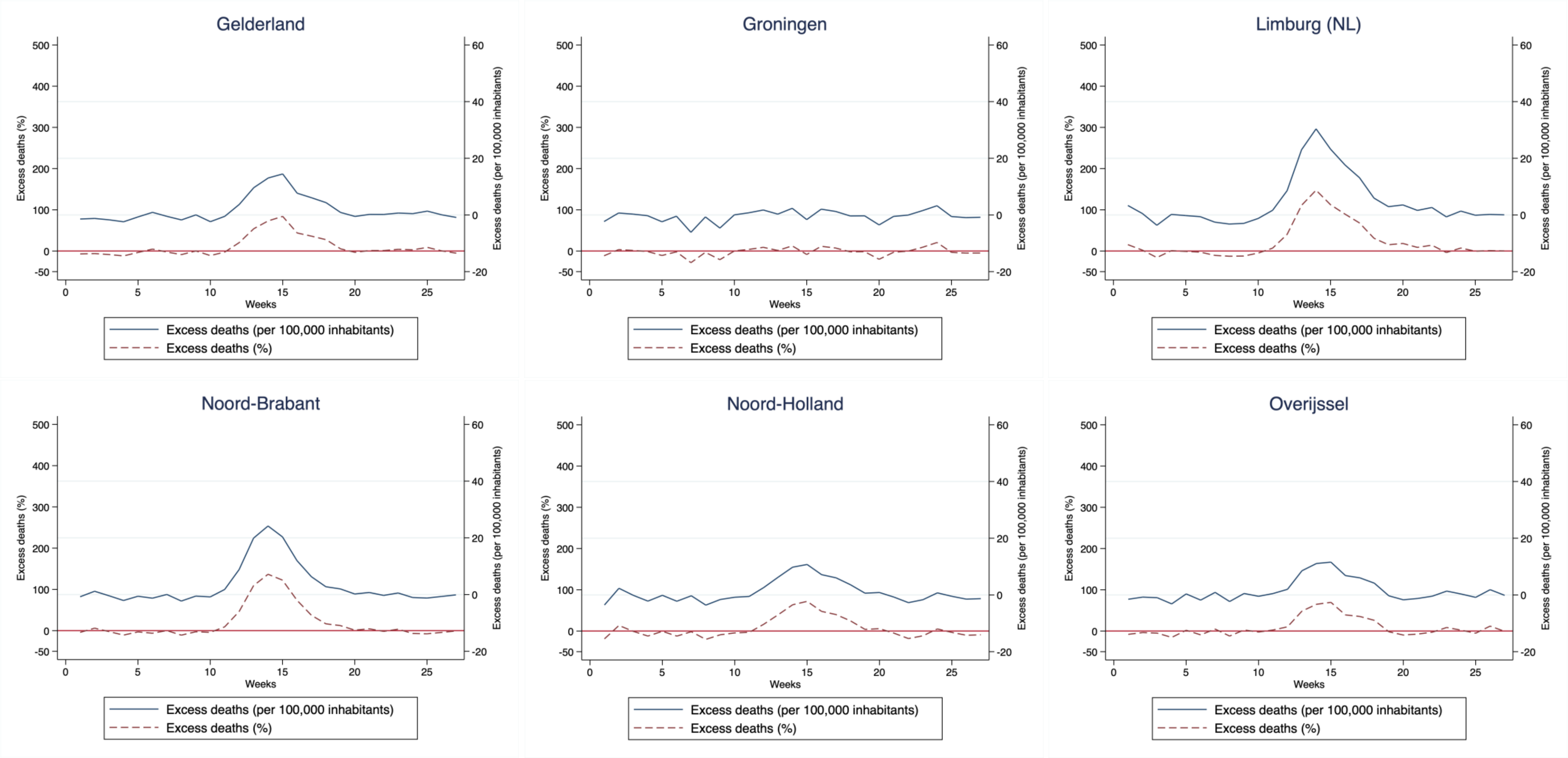
Gelderland
Groningen
Limburg (NL)
Noord-Brabant
Noord-Holland
Overijssel
Excess deaths (%)
Excess deaths (per 100,000 inhabitants)
Weeks
Excess deaths (per 100,000 inhabitants)
Excess deaths (%)

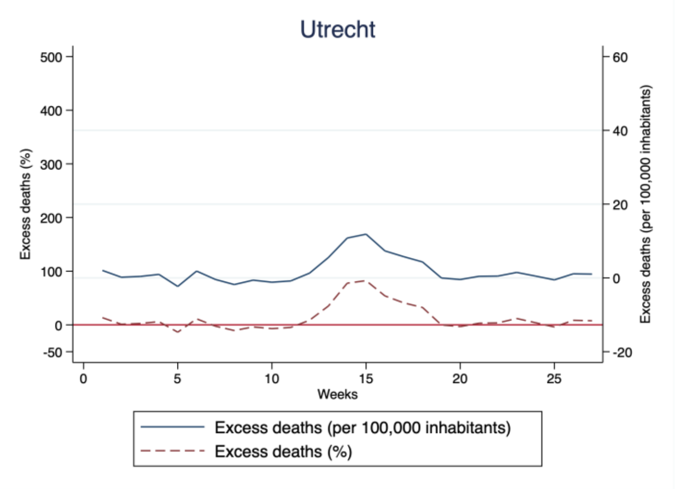
Utrecht
Excess deaths (%)
Excess deaths (per 100,000 inhabitants)
Weeks
Excess deaths (per 100,000 inhabitants)
Excess deaths (%)

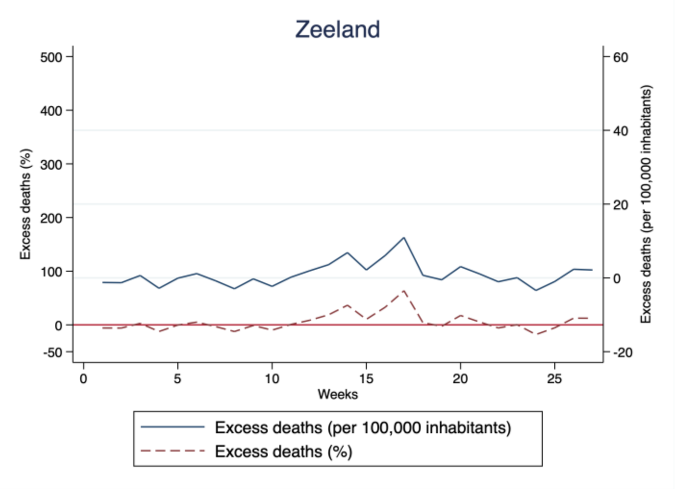
Zeeland
Excess deaths (%)
Excess deaths (per 100,000 inhabitants)
Weeks
Excess deaths (per 100,000 inhabitants)
Excess deaths (%)

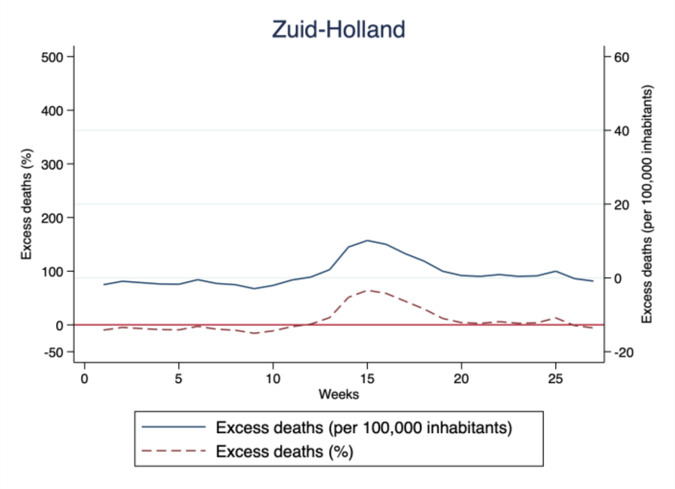
Zuid-Holland
Excess deaths (%)
Excess deaths (per 100,000 inhabitants)
Weeks
Excess deaths (per 100,000 inhabitants)
Excess deaths (%)

NORWAY

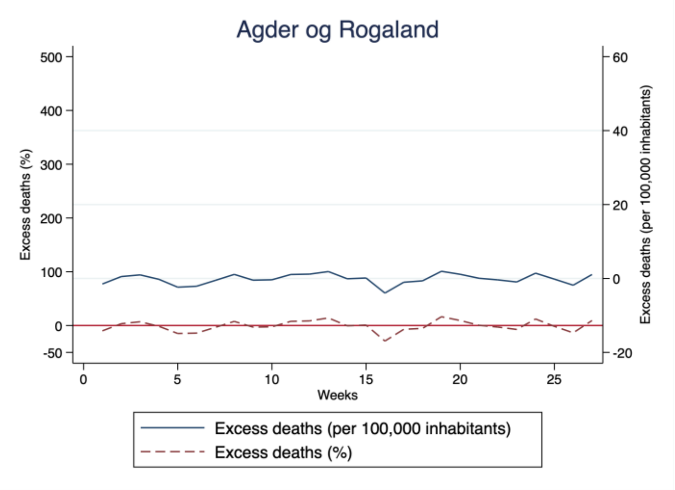
Agder og Rogaland
Excess deaths (%)
Excess deaths (per 100,000 inhabitants)
Weeks
Excess deaths (per 100,000 inhabitants)
Excess deaths (%)

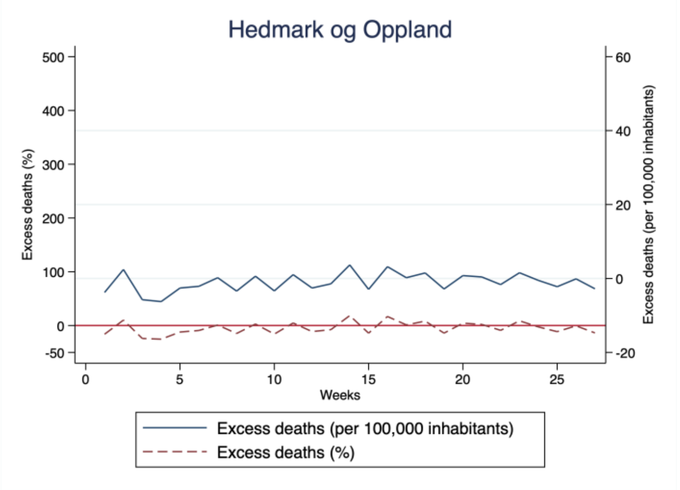
Hedmark og Oppland
Excess deaths (%)
Excess deaths (per 100,000 inhabitants)
Weeks
Excess deaths (per 100,000 inhabitants)
Excess deaths (%)

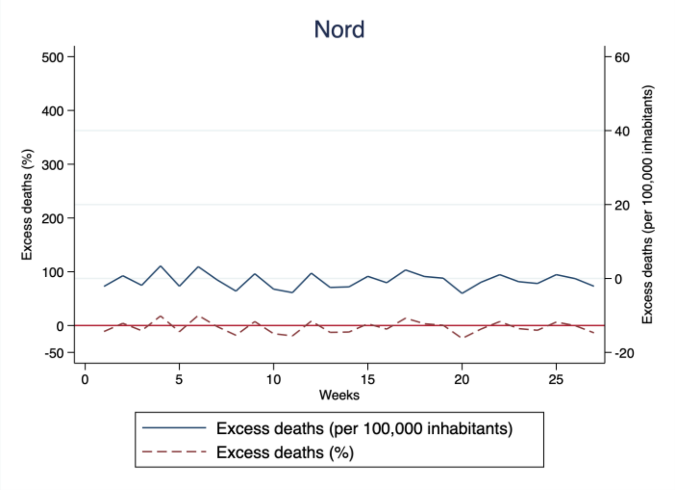
Nord
Excess deaths (%)
Excess deaths (per 100,000 inhabitants)
Weeks
Excess deaths (per 100,000 inhabitants)
Excess deaths (%)

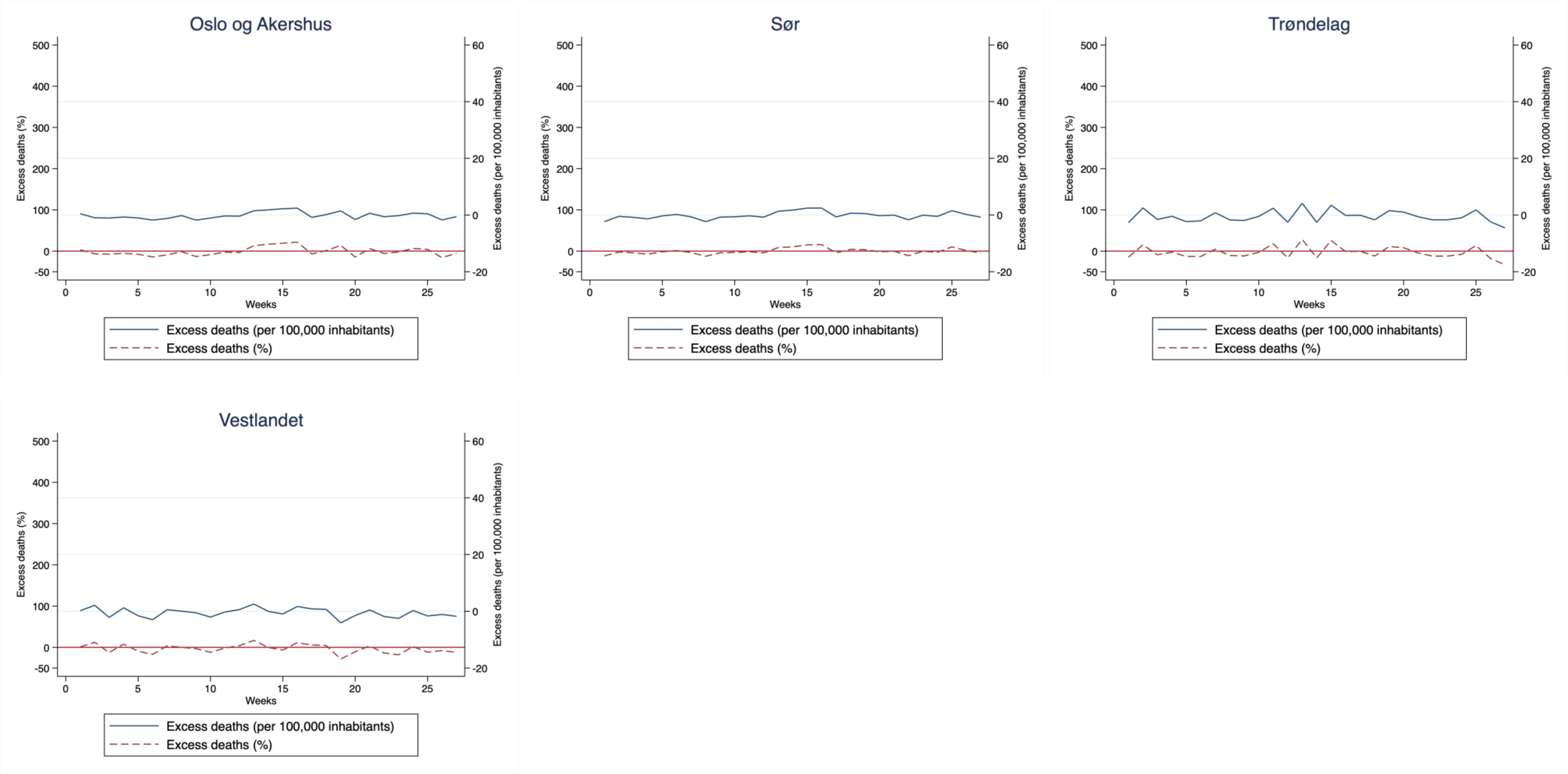

Oslo og Akershus
Sør
Trøndelag
Vestlandet
Excess deaths (%)
Excess deaths (per 100,000 inhabitants)
Weeks
500
400
300
200
100
0
-50
60
40
20
0
-20
0
5
10
15
20
25
Excess deaths (per 100,000 inhabitants)
Excess deaths (%)

POLAND

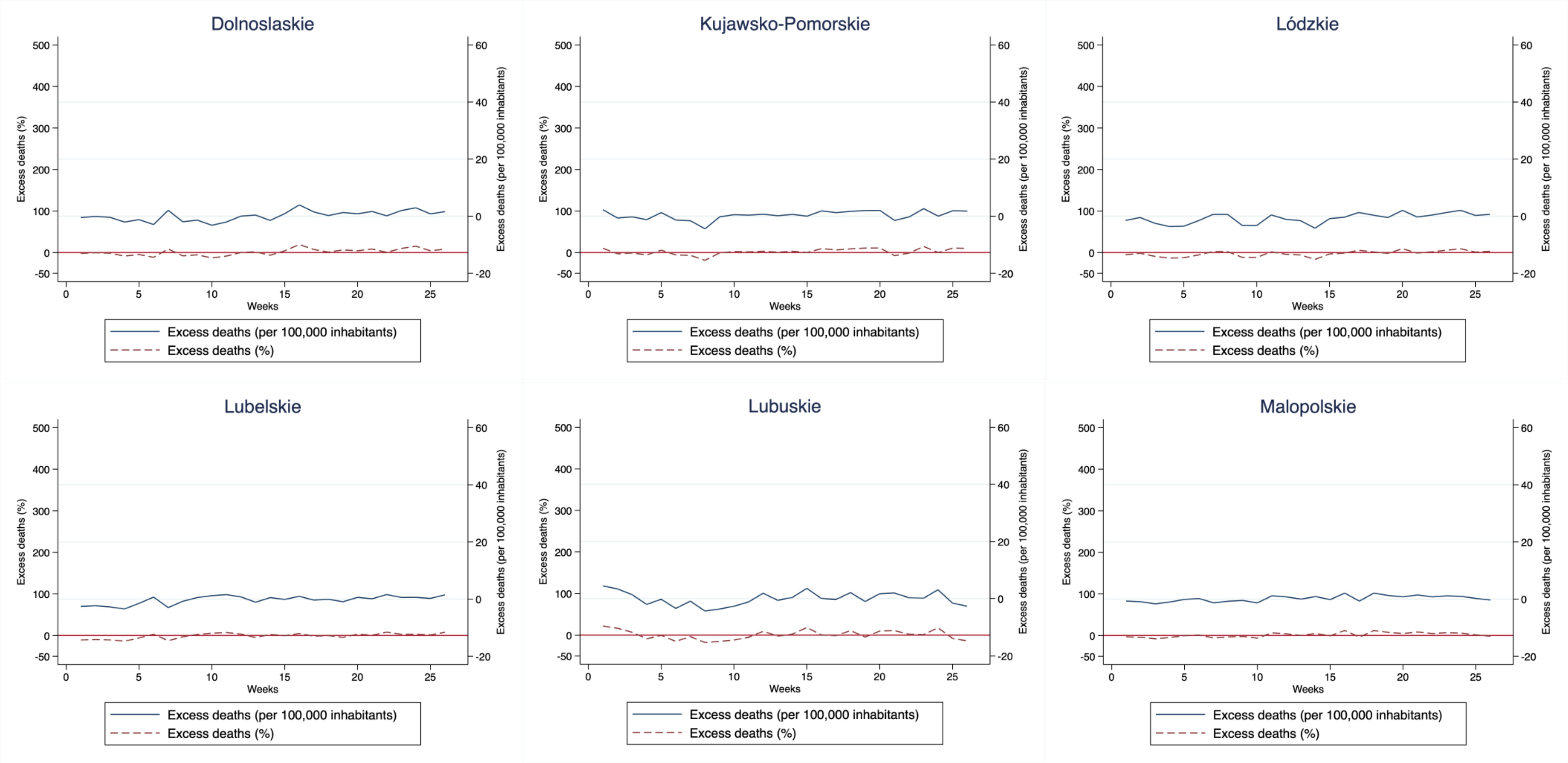
Dolnoslaskie
Kujawsko-Pomorskie
Lódzkie
Lubelskie
Lubuskie
Malopolskie
Excess deaths (%)
Excess deaths (per 100,000 inhabitants)
Weeks
Excess deaths (per 100,000 inhabitants)
Excess deaths (%)

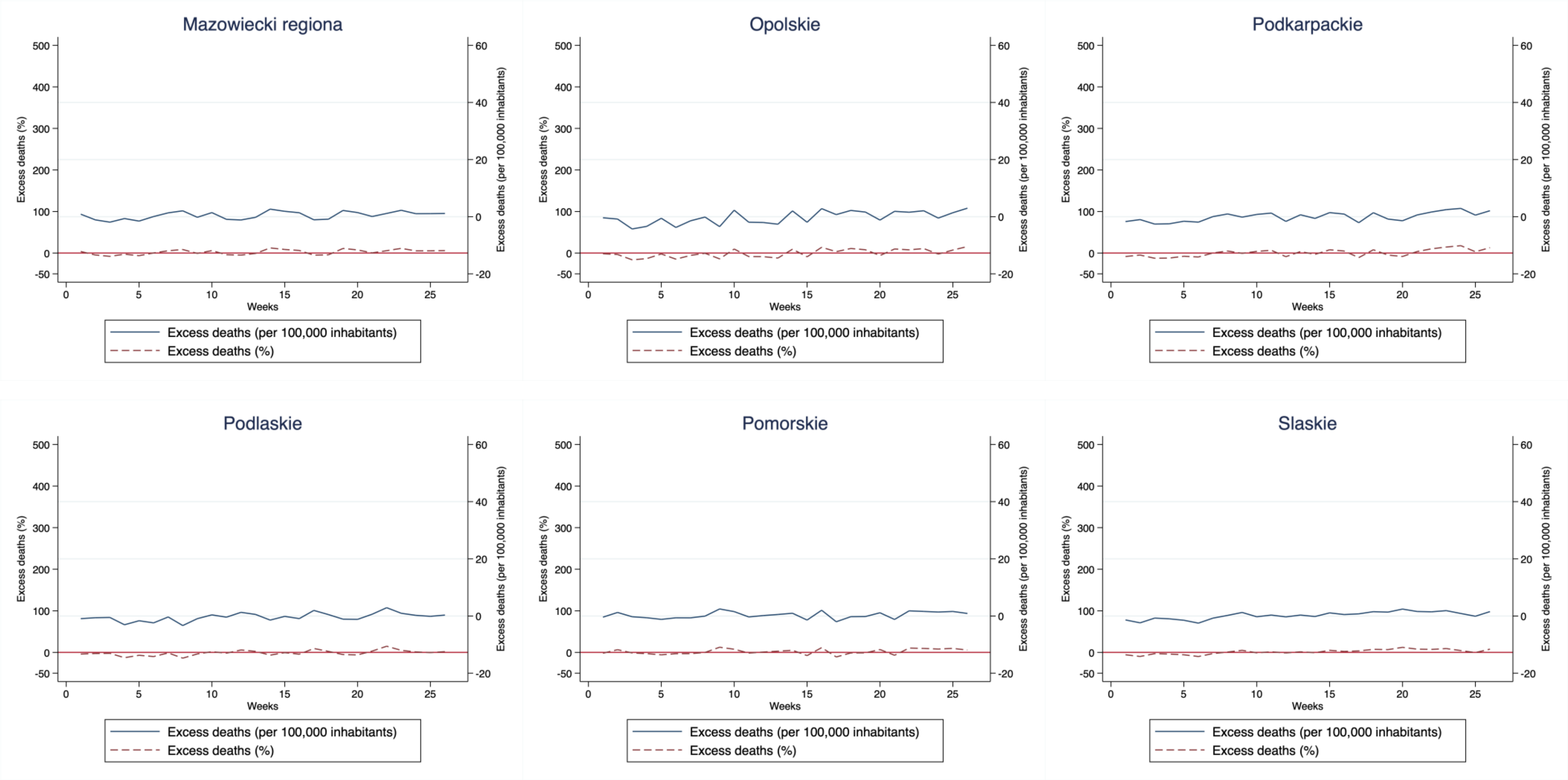
Mazowiecki regiona
Opolskie
Podkarpackie
Podlaskie
Pomorskie
Slaskie
Excess deaths (%)
Excess deaths (per 100,000 inhabitants)
Weeks
500
400
300
200
100
0
-50
60
40
20
0
-20
0
5
10
15
20
25
Excess deaths (per 100,000 inhabitants)
Excess deaths (%)

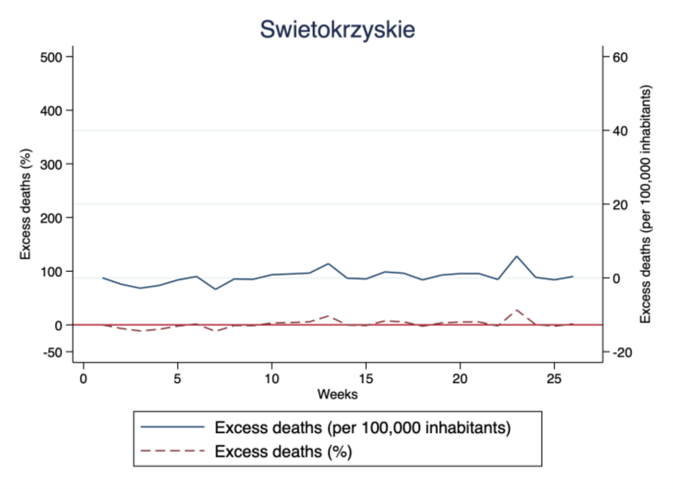
Swietokrzyskie
Excess deaths (%)
Excess deaths (per 100,000 inhabitants)
500
400
300
200
100
0
-50
60
40
20
0
-20
0
5
10
15
20
25
Weeks
Excess deaths (per 100,000 inhabitants)
Excess deaths (%)

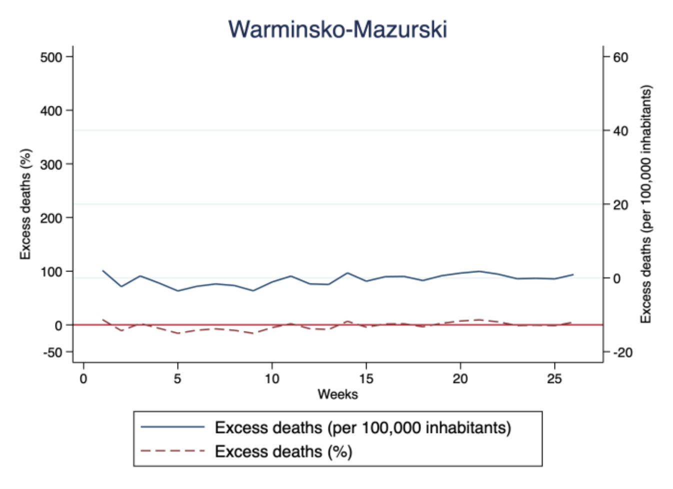
Warminsko-Mazurski
Excess deaths (%)
Excess deaths (per 100,000 inhabitants)
500
400
300
200
100
0
-50
60
40
20
0
-20
0
5
10
15
20
25
Weeks
Excess deaths (per 100,000 inhabitants)
Excess deaths (%)

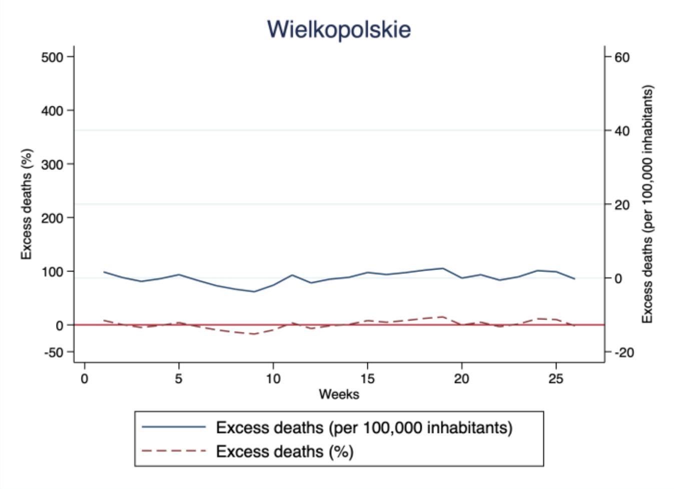
Wielkopolskie
Excess deaths (%)
Excess deaths (per 100,000 inhabitants)
500
400
300
200
100
0
-50
60
40
20
0
-20
0
5
10
15
20
25
Weeks
Excess deaths (per 100,000 inhabitants)
Excess deaths (%)

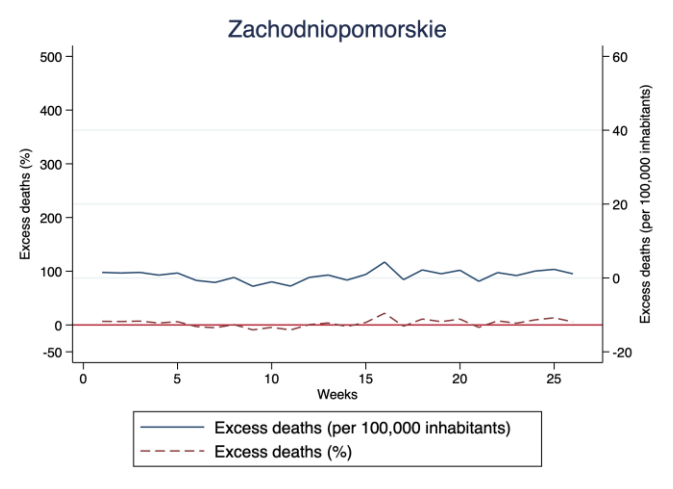
Zachodniopomorskie
Excess deaths (%)
Excess deaths (per 100,000 inhabitants)
500
400
300
200
100
0
-50
60
40
20
0
-20
0
5
10
15
20
25
Weeks
Excess deaths (per 100,000 inhabitants)
Excess deaths (%)

PORTUGAL

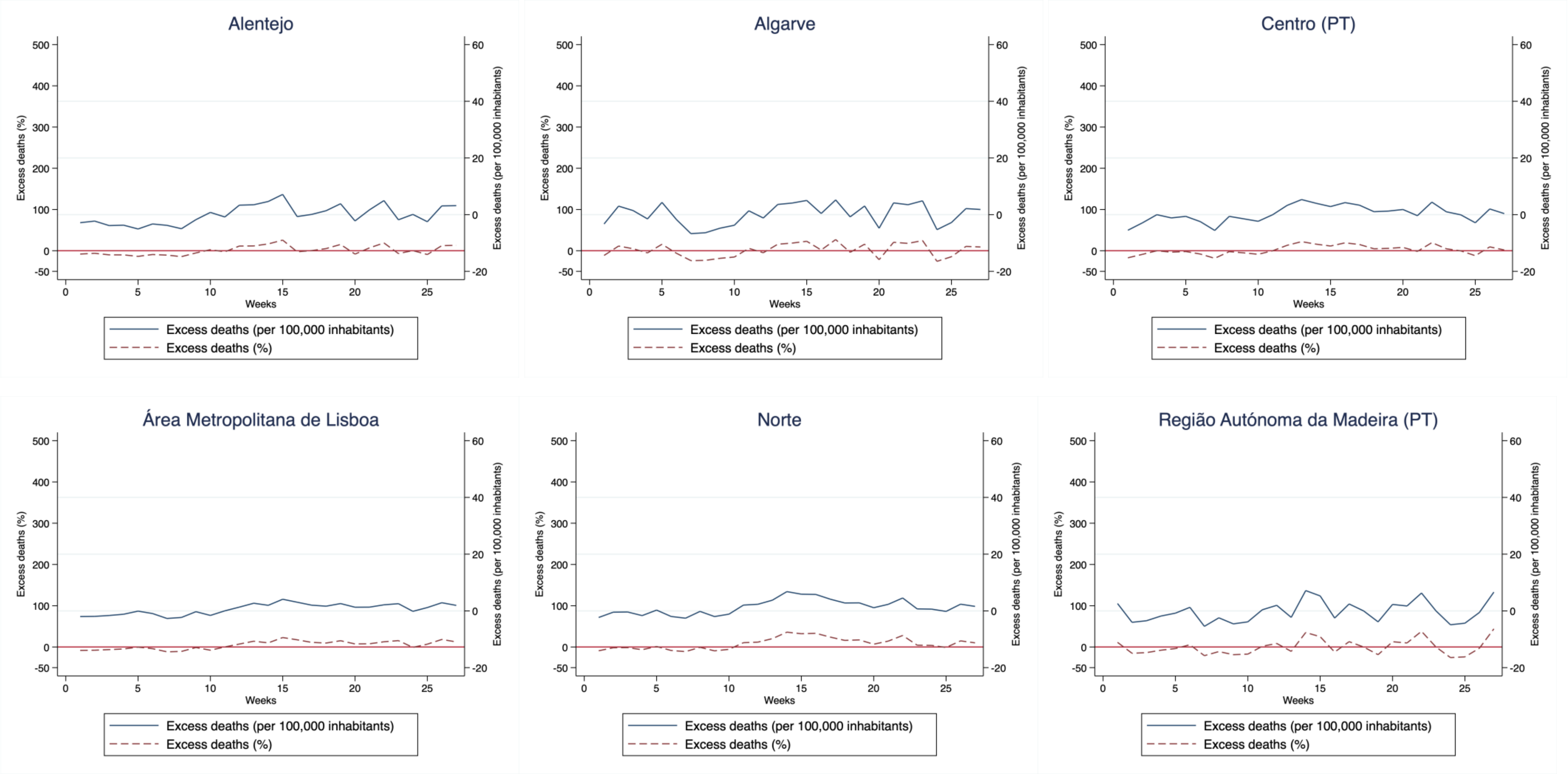

Alentejo
Algarve
Centro (PT)
Área Metropolitana de Lisboa
Norte
Região Autónoma da Madeira (PT)
Excess deaths (%)
Excess deaths (per 100,000 inhabitants)
Weeks
Excess deaths (per 100,000 inhabitants)
Excess deaths (%)

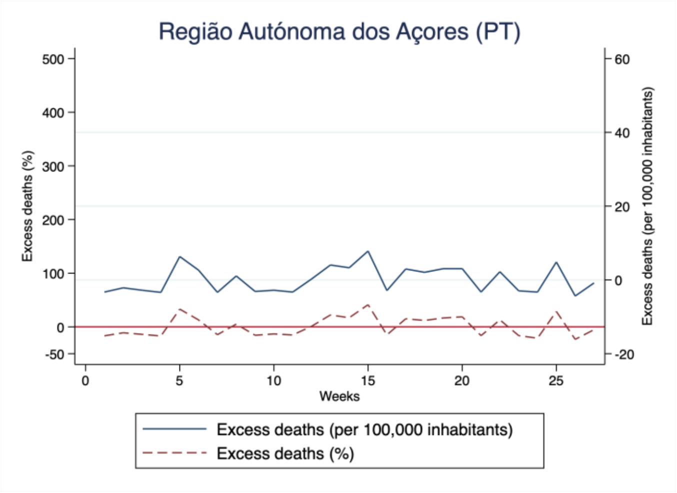
Região Autónoma dos Açores (PT)
Excess deaths (%)
Excess deaths (per 100,000 inhabitants)
Weeks
Excess deaths (per 100,000 inhabitants)
Excess deaths (%)

SLOVAKIA

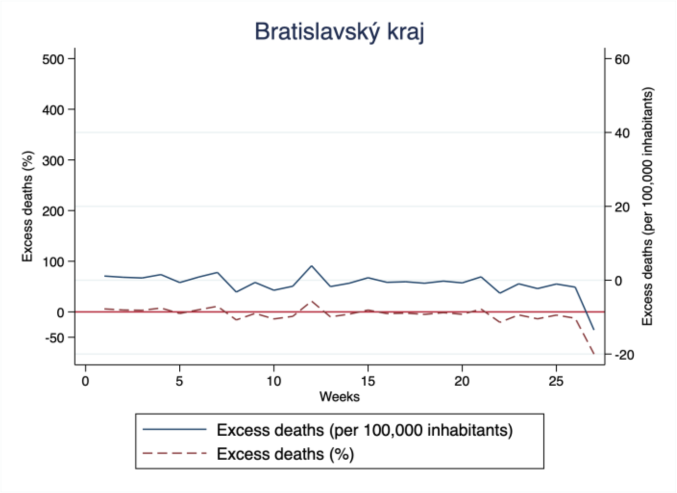
Bratislavský kraj
Excess deaths (%)
Excess deaths (per 100,000 inhabitants)
Weeks
Excess deaths (per 100,000 inhabitants)
Excess deaths (%)

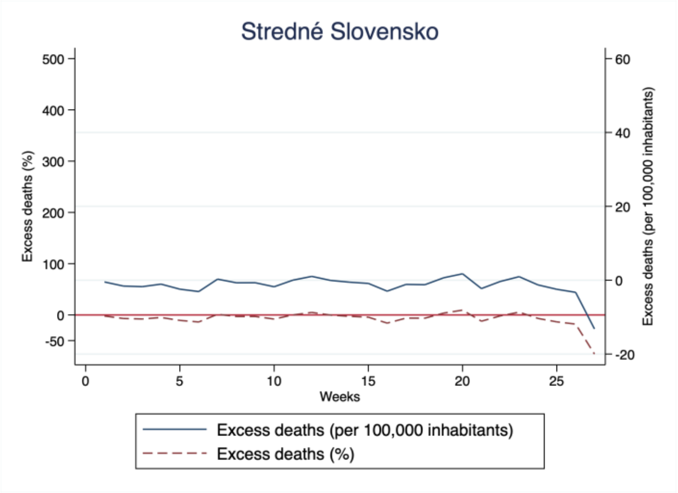
Stredné Slovensko
Excess deaths (%)
Excess deaths (per 100,000 inhabitants)
Weeks
Excess deaths (per 100,000 inhabitants)
Excess deaths (%)

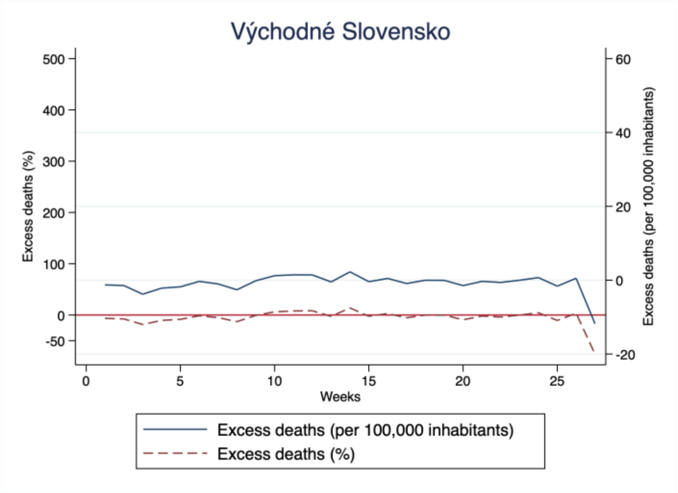
Východné Slovensko
Excess deaths (%)
Excess deaths (per 100,000 inhabitants)
Weeks
Excess deaths (per 100,000 inhabitants)
Excess deaths (%)

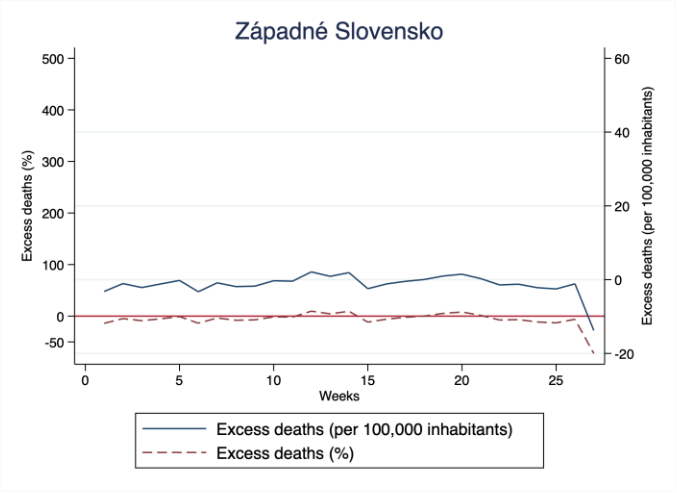
Západné Slovensko
Excess deaths (%)
Excess deaths (per 100,000 inhabitants)
Weeks
Excess deaths (per 100,000 inhabitants)
Excess deaths (%)

SPAIN

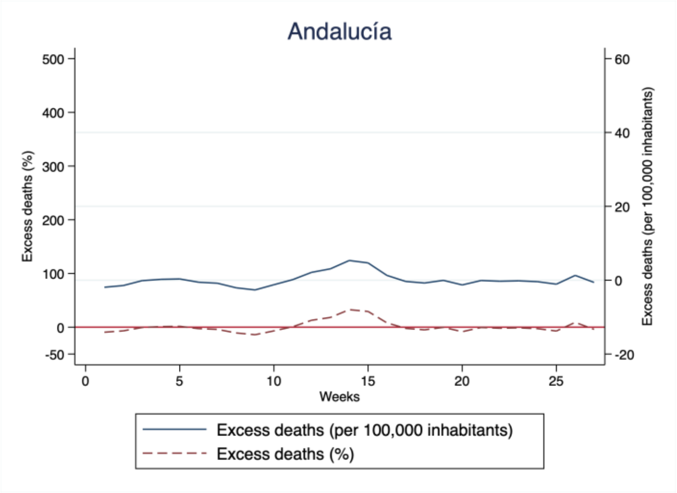
Andalucía
Excess deaths (%)
Excess deaths (per 100,000 inhabitants)
Weeks
Excess deaths (per 100,000 inhabitants)
Excess deaths (%)

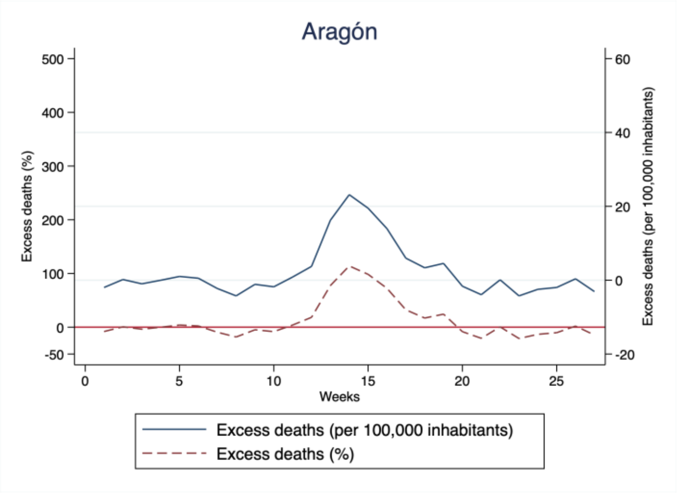
Aragón
Excess deaths (%)
Excess deaths (per 100,000 inhabitants)
Weeks
Excess deaths (per 100,000 inhabitants)
Excess deaths (%)

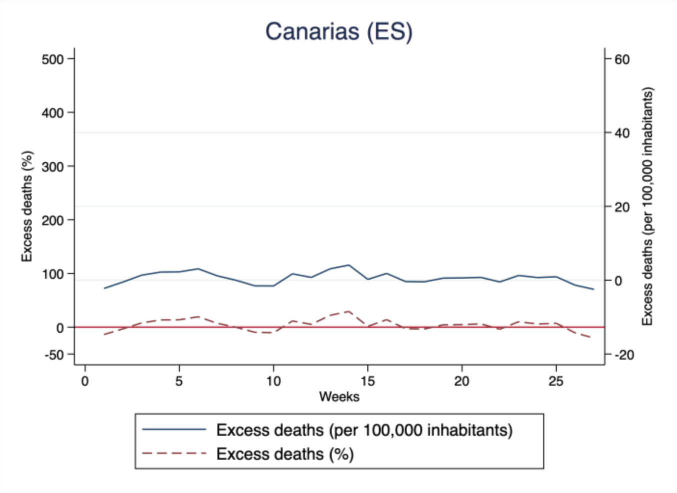
Canarias (ES)
Excess deaths (%)
Excess deaths (per 100,000 inhabitants)
Weeks
Excess deaths (per 100,000 inhabitants)
Excess deaths (%)

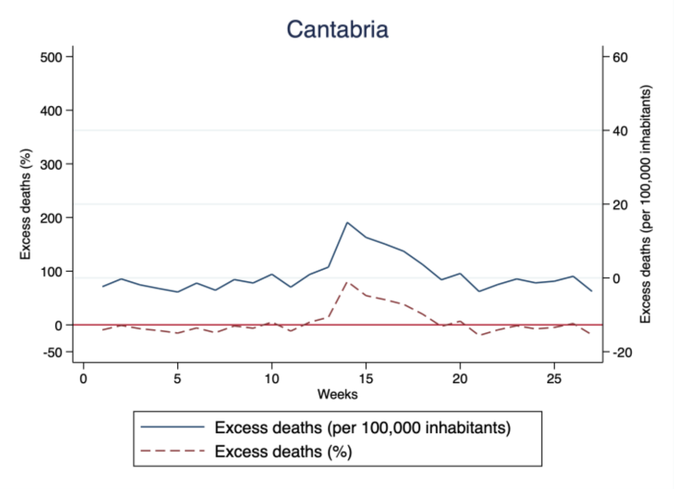
Cantabria
Excess deaths (%)
Excess deaths (per 100,000 inhabitants)
Weeks
Excess deaths (per 100,000 inhabitants)
Excess deaths (%)

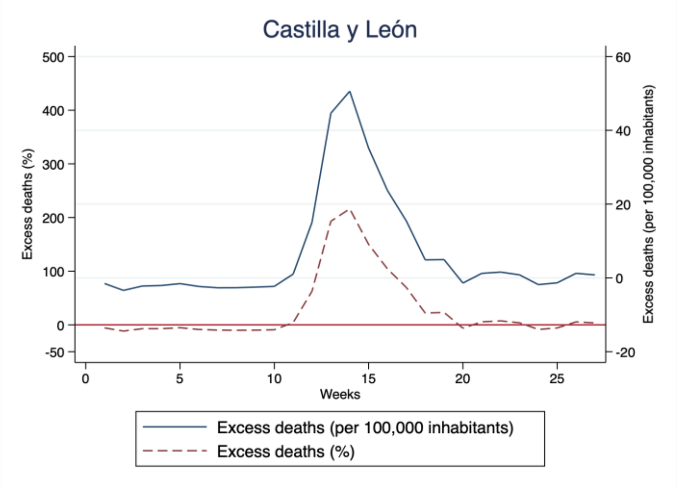
Castilla y León
Excess deaths (%)
Excess deaths (per 100,000 inhabitants)
Weeks
Excess deaths (per 100,000 inhabitants)
Excess deaths (%)

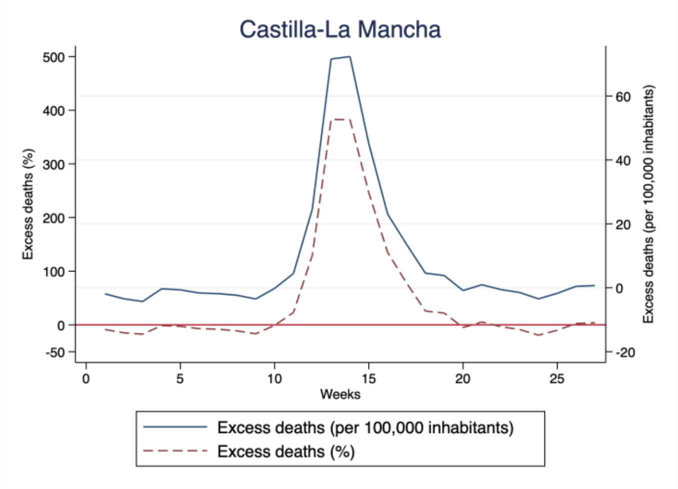
Castilla-La Mancha
Excess deaths (%)
Excess deaths (per 100,000 inhabitants)
Weeks
Excess deaths (per 100,000 inhabitants)
Excess deaths (%)

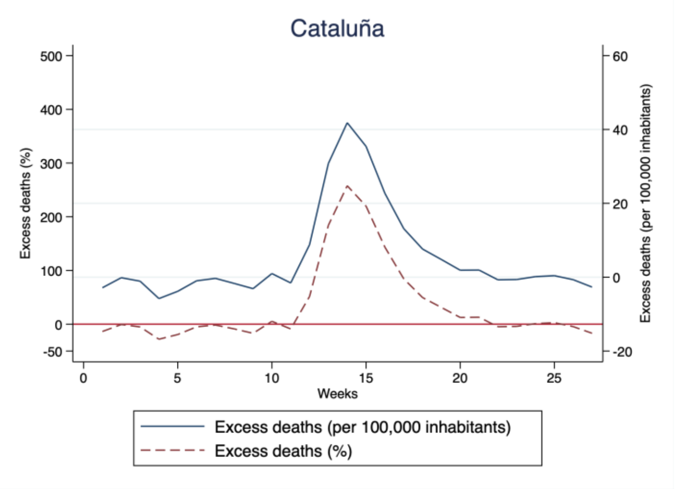
Cataluña
Excess deaths (%)
Excess deaths (per 100,000 inhabitants)
Weeks
Excess deaths (per 100,000 inhabitants)
Excess deaths (%)

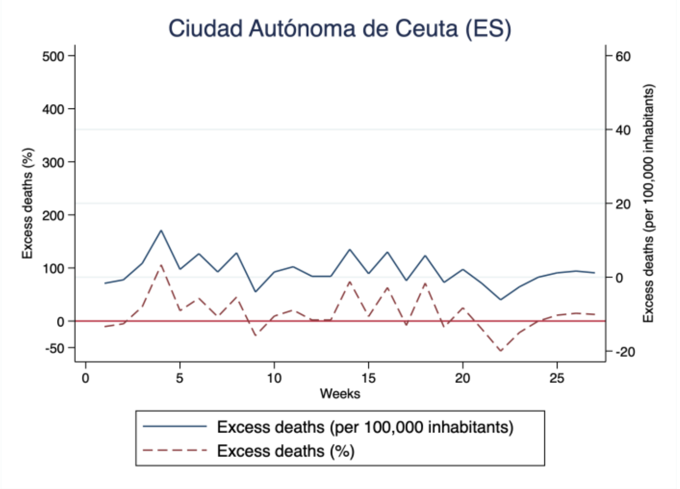
Ciudad Autónoma de Ceuta (ES)
Excess deaths (%)
Excess deaths (per 100,000 inhabitants)
Weeks
Excess deaths (per 100,000 inhabitants)
Excess deaths (%)

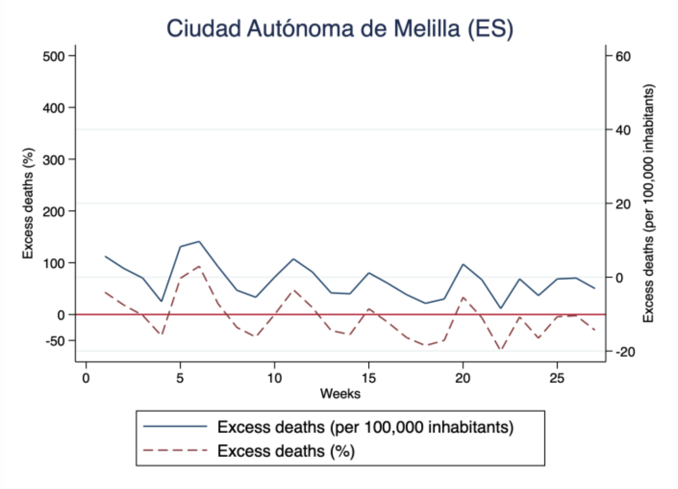
Ciudad Autónoma de Melilla (ES)
Excess deaths (%)
Excess deaths (per 100,000 inhabitants)
Weeks
Excess deaths (per 100,000 inhabitants)
Excess deaths (%)

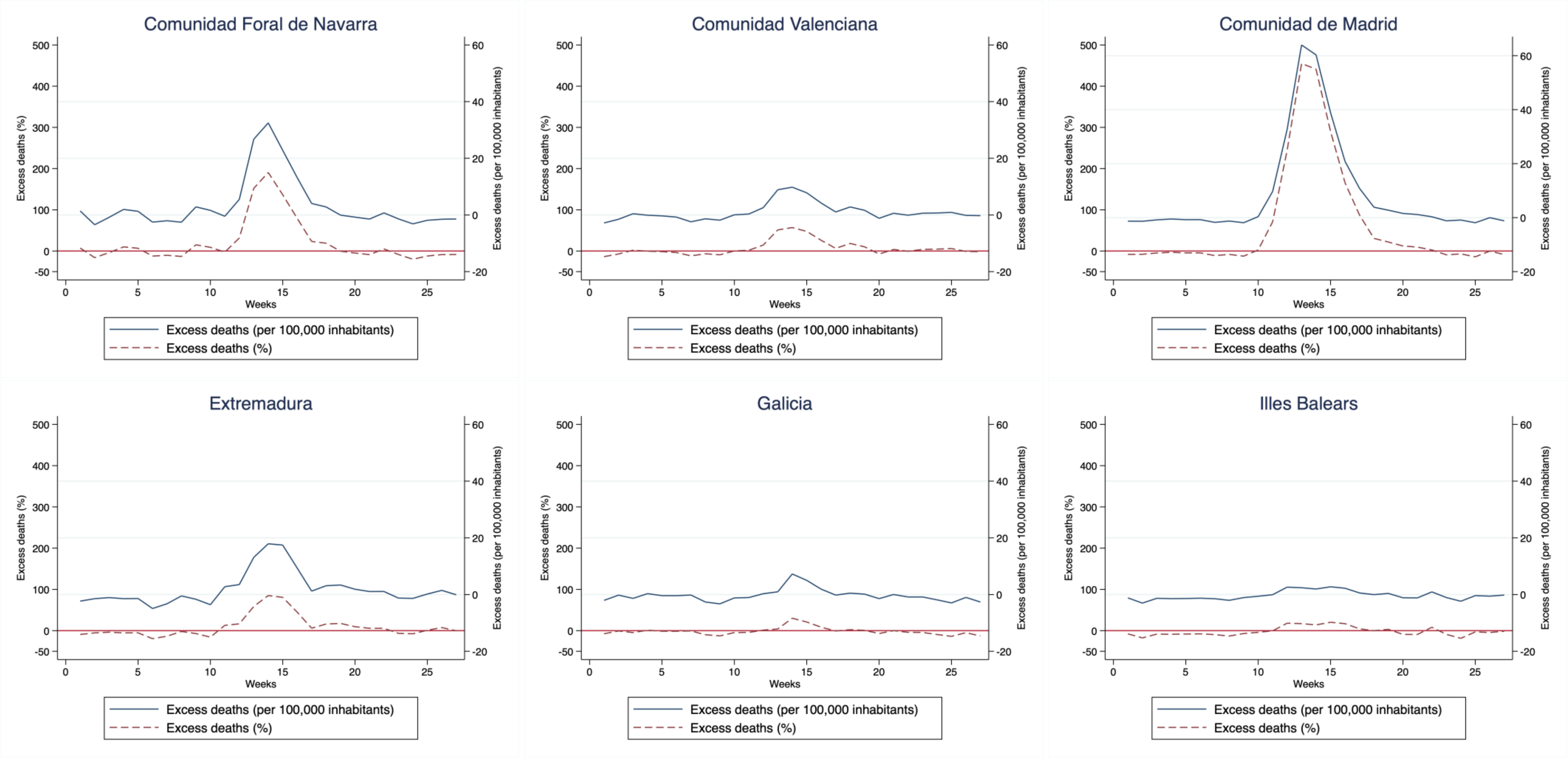
Comunidad Foral de Navarra
Comunidad Valenciana
Comunidad de Madrid
Extremadura
Galicia
Illes Balears
Excess deaths (%)
Excess deaths (per 100,000 inhabitants)
Weeks
Excess deaths (per 100,000 inhabitants)
Excess deaths (%)

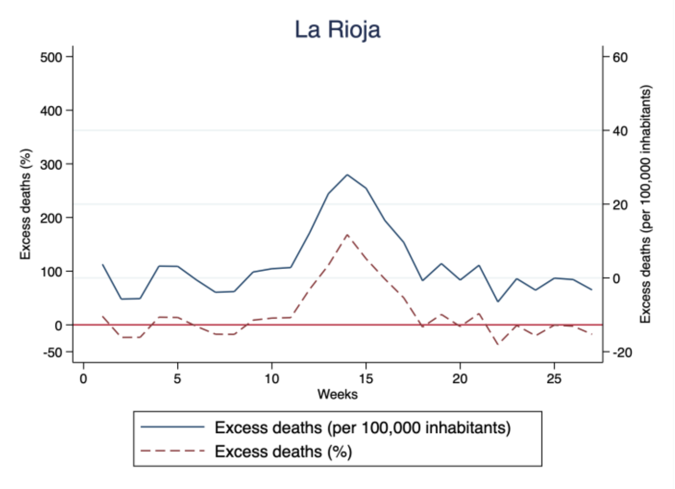
La Rioja
Excess deaths (%)
Excess deaths (per 100,000 inhabitants)
Weeks
Excess deaths (per 100,000 inhabitants)
Excess deaths (%)

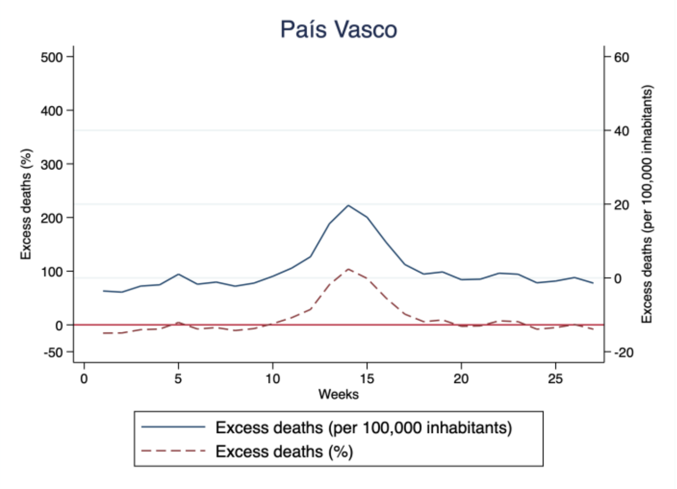
País Vasco
Excess deaths (%)
Excess deaths (per 100,000 inhabitants)
Weeks
Excess deaths (per 100,000 inhabitants)
Excess deaths (%)

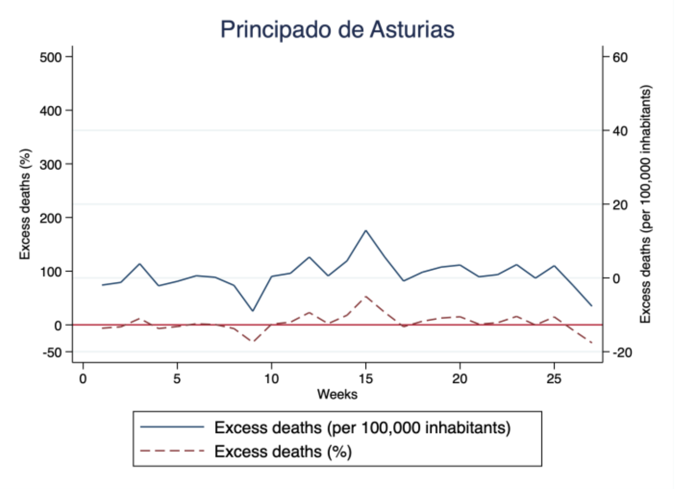
Principado de Asturias
Excess deaths (%)
Excess deaths (per 100,000 inhabitants)
Weeks
Excess deaths (per 100,000 inhabitants)
Excess deaths (%)

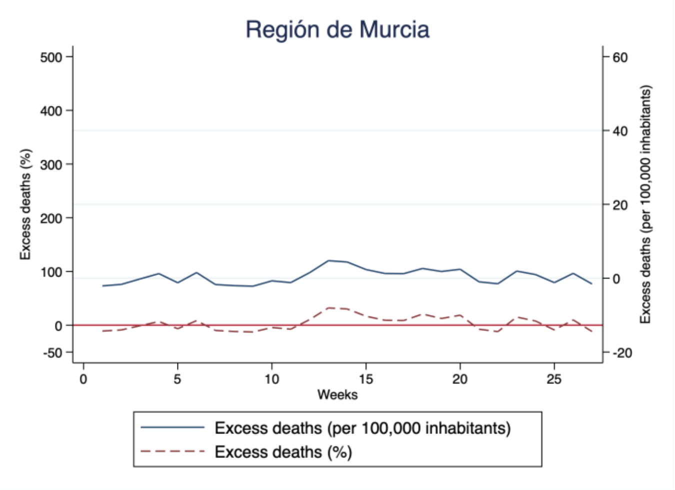
Región de Murcia
Excess deaths (%)
Excess deaths (per 100,000 inhabitants)
Weeks
Excess deaths (per 100,000 inhabitants)
Excess deaths (%)

SWEDEN

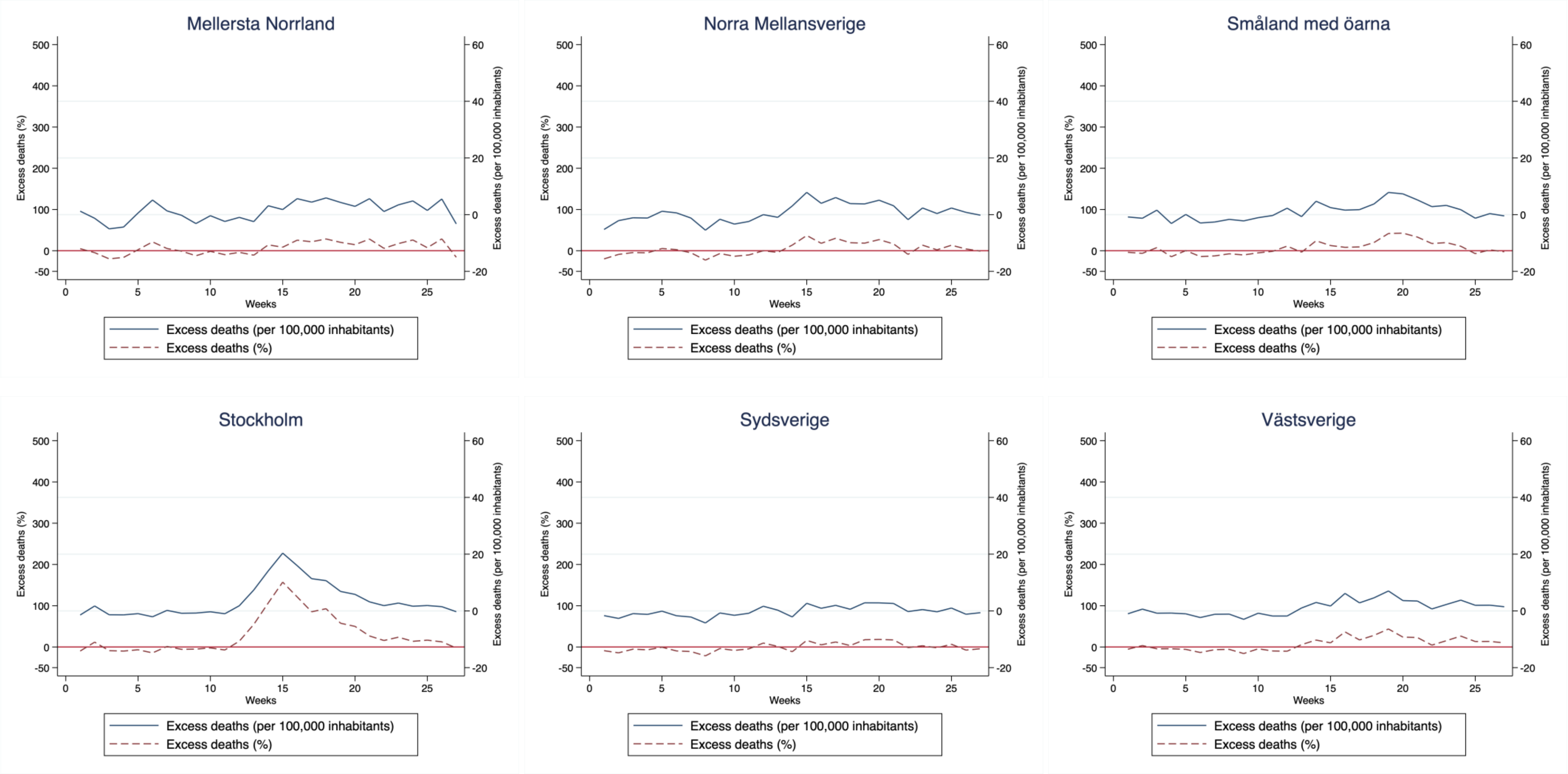
Mellersta Norrland
Norra Mellansverige
Småland med öarna
Stockholm
Sydsverige
Västsverige
Excess deaths (%)
Excess deaths (per 100,000 inhabitants)
Weeks
Excess deaths (per 100,000 inhabitants)
Excess deaths (%)

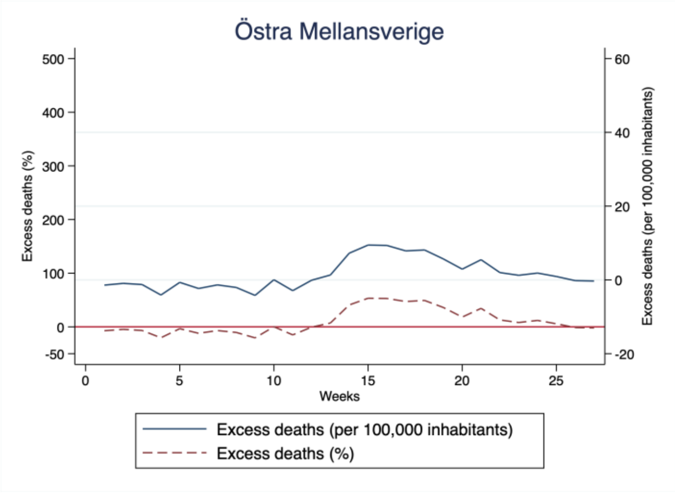

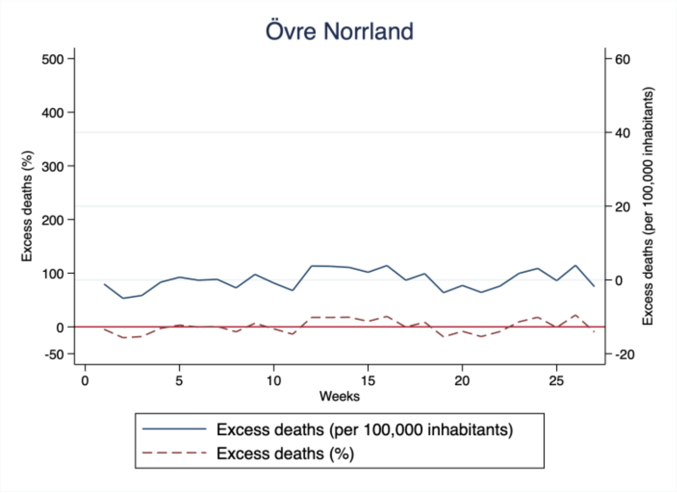

SWITZERLAND

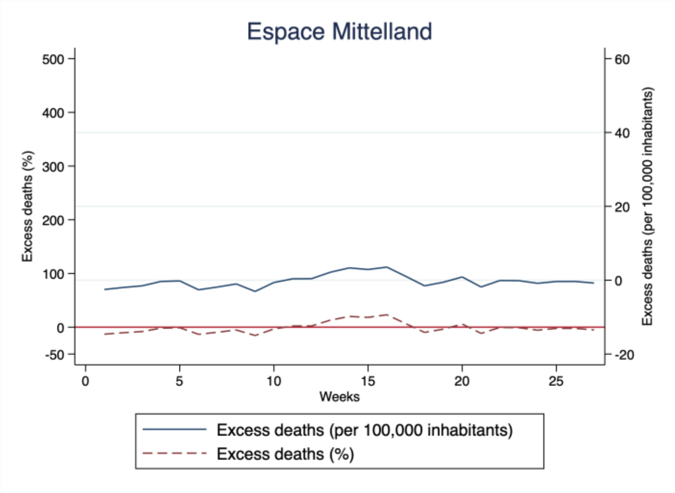

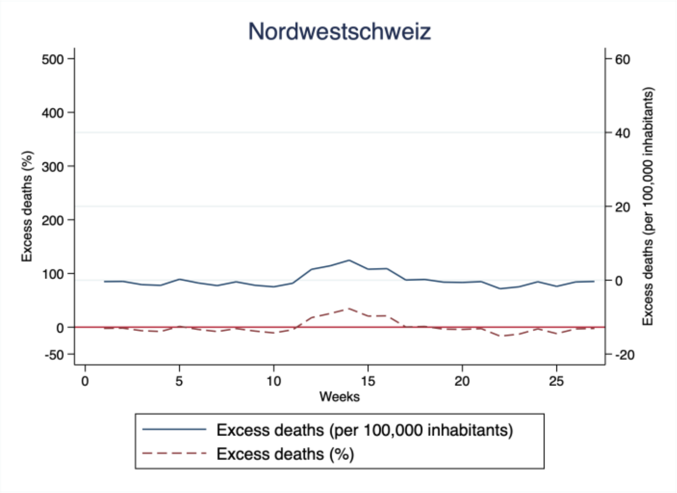

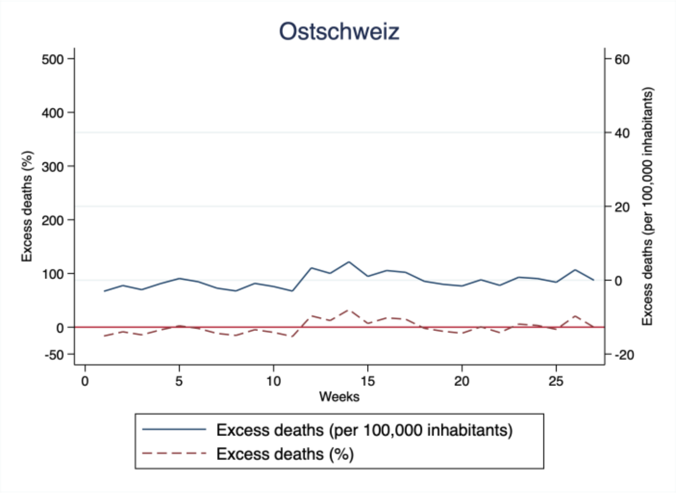

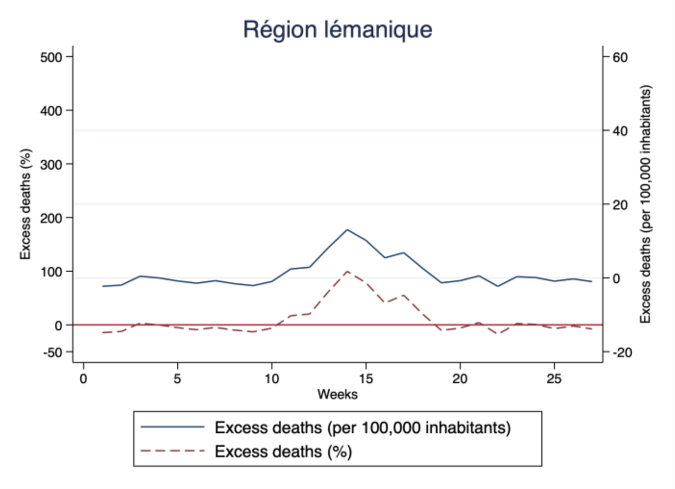
Région lémanique
Excess deaths (%)
Excess deaths (per 100,000 inhabitants)
Weeks
Excess deaths (per 100,000 inhabitants)
Excess deaths (%)

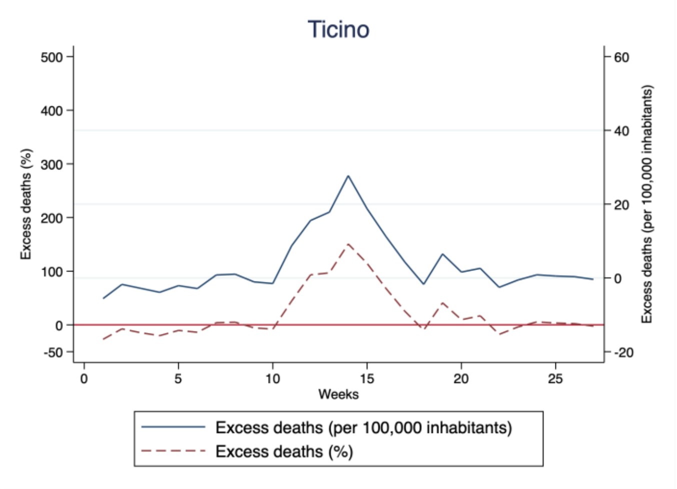
Ticino
Excess deaths (%)
Excess deaths (per 100,000 inhabitants)
Weeks
Excess deaths (per 100,000 inhabitants)
Excess deaths (%)

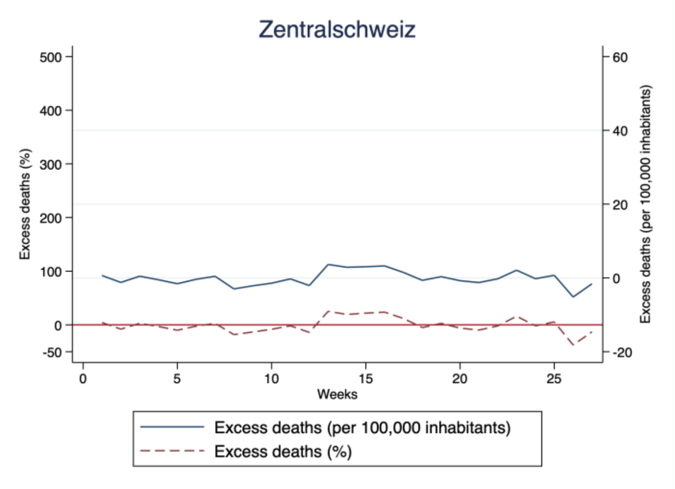
Zentralschweiz
Excess deaths (%)
Excess deaths (per 100,000 inhabitants)
Weeks
Excess deaths (per 100,000 inhabitants)
Excess deaths (%)

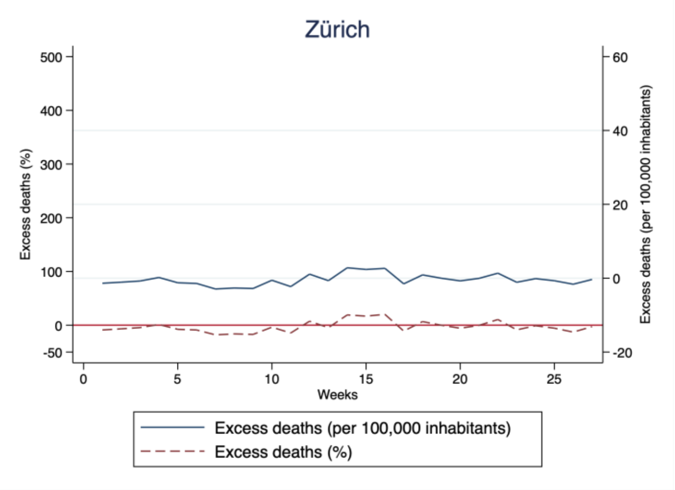
Zürich
Excess deaths (%)
Excess deaths (per 100,000 inhabitants)
Weeks
Excess deaths (per 100,000 inhabitants)
Excess deaths (%)

UNITED KINGDOM

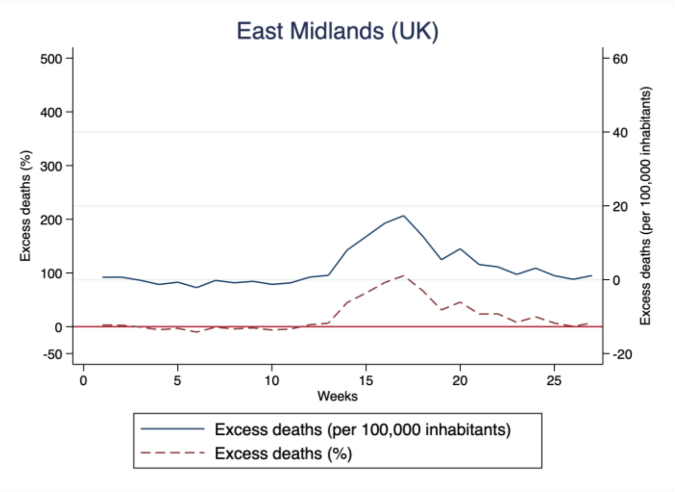

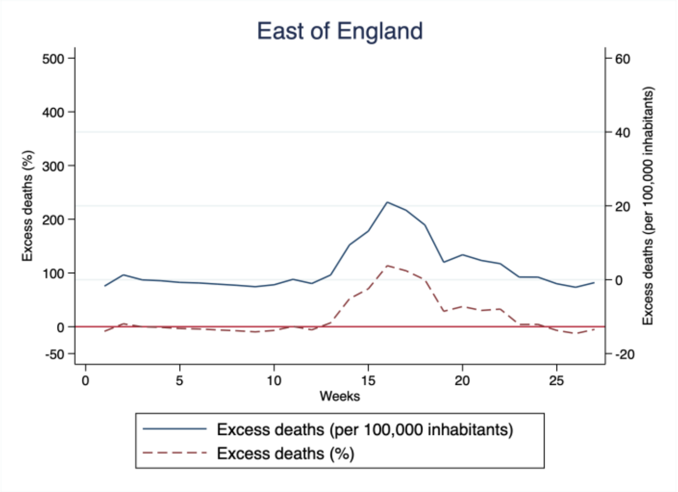

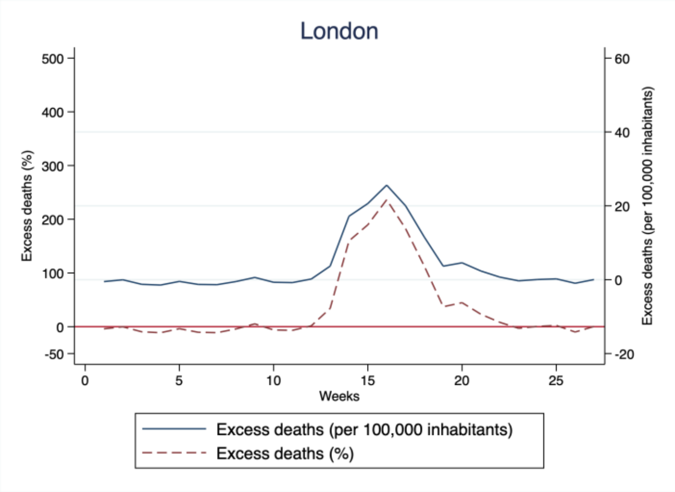

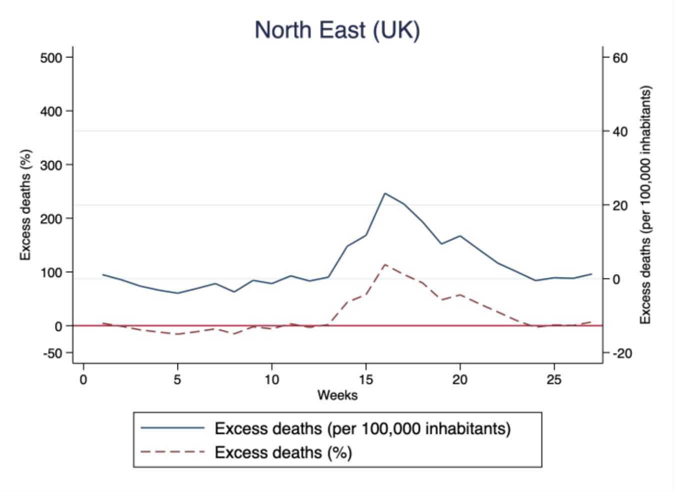

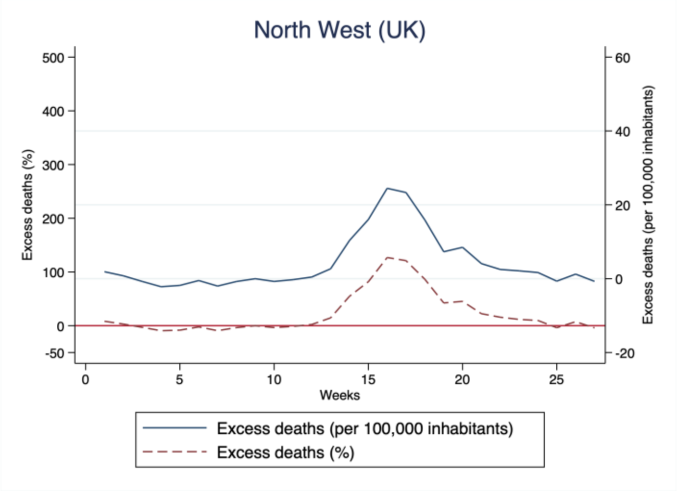

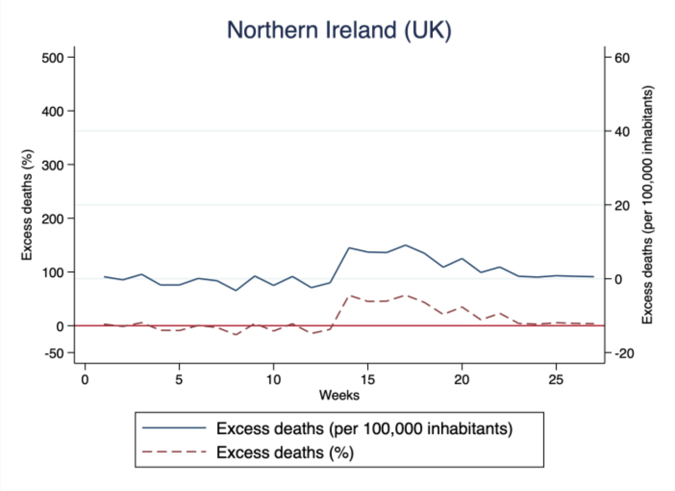

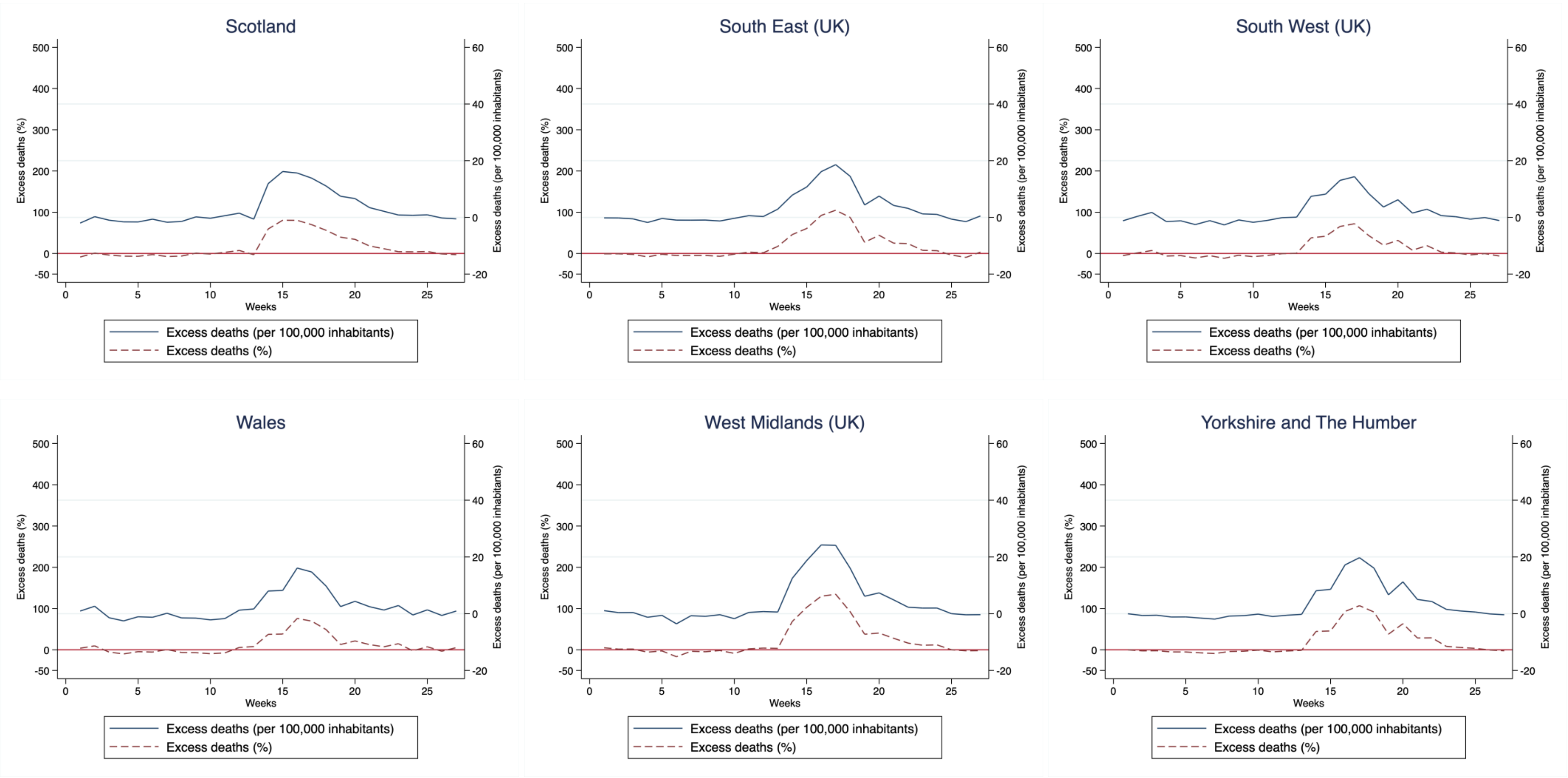

Scotland
South East (UK)
South West (UK)
Wales
West Midlands (UK)
Yorkshire and The Humber
Excess deaths (%)
Excess deaths (per 100,000 inhabitants)
Weeks
500
400
300
200
100
0
-50
60
40
20
0
-20
0
5
10
15
20
25
Excess deaths (per 100,000 inhabitants)
Excess deaths (%)

APPENDIX B

Table B1. Variable description

Macro-Area	Variable	Year	Description	Level	Source
Agglomeration	Population	2019	Number of inhabitants	NUTS2	EUROSTAT
	GDP	2018	Gross Domestic Product	NUTS2	EUROSTAT
Regional Wealth	GDP per capita	2018	Share of GDP per 100,000 inhabitants	NUTS2	EUROSTAT
Density	Population density	2018	Population per km^2	NUTS2	EUROSTAT
Accessibility and connectivity	Air passengers	2018	Air transport of passengers	NUTS2	EUROSTAT
	Accessibility by road	2014	Road accessibility measured as the inverse time-distance weighted population	NUTS2	Raw data gathered by Klaus Spiekermann EU Commission
Age structure	Median population age	2019	Median age of the population	NUTS2	EUROSTAT
	Share of population over 65	2019	Number of people over 65 years old over the total population	NUTS2	EUROSTAT
	Share of population over 75	2019	Number of people over 75 years old over the total population	NUTS2	EUROSTAT
Education	Share of adults with higher education	2017	Adult population (aged 25 to 64 years old) with tertiary education (5-8 ISCED level)	NUTS2	EUROSTAT
Health system	Doctors per capita	2017	Number of doctors over population	NUTS2	EUROSTAT
	Hospital beds per capita	2017	Number of hospital beds over population	NUTS2	EUROSTAT
Air pollution	Air pollution	2016	Exposure to air pollution by particulate matter (PM 2.5)	NUTS 0	OECD
	Total household carbon footprint	2010	Total household carbon footprint (kgCO2e/cap)	NUTS2	Ivanova et al. (2017)

	Average household carbon footprint	2010	Total household carbon footprint (kgCO2e/cap) per family	NUTS2	Ivanova et al. (2017)
Climate	Average temperature	2019	Average temperature in C°	NUTS2	Agri-4-Cast EU-JRC
	Average precipitation	2019	Average precipitation (mm)	NUTS2	Agri-4-Cast EU-JRC
Autonomy	Regional autonomy	2016	Regional Authority Index (RAI)	NUTS2	Hooghe et al. (2016)
Government Quality	Regional government quality	2017	European Quality of Government Index (EQI)	NUTS2	Charron et al. (2016)<br>University of Gothemburg
	Change in regional government quality	2017-2010	European Quality of Government Index (EQI)	NUTS2	Charron et al. (2016)<br>University of Gothenburg
	National government effectiveness	2018	Worldwide Governance Indicators	NUTS0	Kaufmann and Kraay (2020)<br>World Bank
	Change in national government effectiveness	2018-1998	Worldwide Governance Indicators	NUTS0	Kaufmann and Kraay (2020)<br>World Bank
Social capital & trust	Frequency of meeting friends (once a week or more)	2016	Share of the population meeting friends & family socially at least once a week	NUTS2	European Social Survey
	Generalised trust	2016	Level of trust in others	NUTS2	European Social Survey
	Bridging networks	2016	Participation in voluntary association that encourage relationships between dissimilar or unfamiliar people	NUTS2	European Social Survey

Table B2. Summary statistics

VARIABLES	(1) N	(2) Mean	(3) St. Dev.	(4) Min.	(5) Max
Excess mortality (%)	206	4.354	10.35	-7.712	60.86
Excess mortality (x 100,00 inhab)	206	19.16	49.91	-77.95	247.0
Median population age 2019	211	43.27	3.790	17.70	51
Share of adults with higher education 2017	209	38.86	10.82	18.90	74
Regional government quality 2017	190	0.125	0.963	-2.264	2.323
Change in regional government quality 2010-2017	190	0.00241	0.403	-1.127	1.203
Regional autonomy 2016	200	14.71	7.569	1	27
Average temperature 2019	202	11.38	3.242	0.538	20.01
Average precipitation 2019	202	2.010	0.777	0.223	4.933
Generalised trust 2016	171	-0.0352	0.381	-1.507	0.635
Bridging networks 2016	171	0.0230	0.369	-0.885	1.843
National government effectiveness 2018	211	1.199	0.544	0.112	2.040
Change in national government effectiveness 1998-2018	211	-0.173	0.352	-0.670	1
Share of population over 65 2019	210	0.238	0.042	0.031	0.343
Share of population over 75 2019	210	0.131	0.030	0.014	0.216
Population 2019	211	14.24	0.961	10.30	16.70
Population density 2018	211	4.935	1.277	1.224	8.919
Air passengers 2018	211	5.814	3.928	0	11.56
GDP 2018	197	10.57	1.182	7.219	13.51
GDP per capita 2018	197	10.11	0.586	8.294	11.50
Accessibility by road 2014	202	14.50	0.905	11.53	15.97
Total household carbon footprint 2010 (ln)	173	23.79	1.123	19.91	26.21
Average household carbon footprint 2010 (ln)	173	9.249	0.267	8.605	9.993
Doctors per capita 2017	178	-5.627	0.309	-7.211	-4.846
Hospital beds per capita 2017	184	-5.360	0.449	-6.613	-4.353
Air pollution 2016	195	2.400	0.431	0.788	3.411
Frequency of meeting friends 2016	168	63.35	14.21	16	91.54

Appendix C

Table C1. Excess mortality (per 100,000 inhabitants) and agglomeration, regional wealth, density, accessibility and connectivity

Dependent variable: Excess mortality in the first six months of 2020	(1)	(2)	(3)	(4)	(5)	(6)	(7)
	Agglomeration		***Regional wealth***	***Density***	***Accessibility & connectivity***		
	OLS	OLS	OLS	OLS	OLS	OLS	OLS
Population 2019 (ln)	8.374*						
	(4.917)						
GDP 2018 (ln)		11.325***					
		(4.257)					
GDP per capita 2018 (ln)			53.214***				
			(13.161)				
Population density 2018 (ln)				5.198			
				(3.577)			
Air passengers 2018 (ln)					0.675		-0.271
					(1.004)		(0.868)
Accessibility by road 2014 (ln)						31.740***	32.056***
						(6.279)	(6.271)
Country fixed effects	Yes	Yes	Yes	Yes	Yes	Yes	Yes
No. of regions	206	192	192	206	206	201	201
R^2	0.435	0.459	0.487	0.427	0.418	0.536	0.536
Adjusted R^2	0.364	0.392	0.423	0.355	0.344	0.476	0.473

Robust standard errors in parentheses
*** p<0.01, ** p<0.05, * p<0.1

Table C2. Excess mortality (per 100,000 inhabitants) and age structure, education, readiness of the health system, and environmental conditions

Dependent variable:	(1)	(2)	(3)	(4)	(5)	(6)	(7)	(8)	(9)	(10)	(11)	(12)	(13)
Excess mortality in the first six		***Age structure***		***Education***		***Health system***			***Air pollution***			***Climate***	
months of 2020	OLS	OLS	OLS	OLS	OLS	OLS	OLS	OLS	OLS	OLS	OLS	OLS	OLS
Median population age 2019	-0.841												
	(0.888)												
Share of population over 65 2019		-67.948											
		(87.268)											
Share of population over 75 2019			-32.756										
			(146.703)										
Share of adults with higher education 2017				0.949***									
				(0.300)									
Doctors per capita 2017 (ln)					-0.191		-4.609						
					(12.512)		(17.958)						
Hospital beds per capita 2017 (ln)						10.279	6.108						
						(13.877)	(14.253)						
Air pollution 2016 (ln)								34.298					
								(23.723)					
Total household carbon footprint 2010 (ln)									9.762*				
									(5.402)				
Average household carbon footprint 2010 (ln)										120.039***			
										(30.520)			
Average temperature 2019											-7.205***		-7.712***
											(1.692)		(1.965)
Average Precipitation 2019												5.940	-3.502
												(6.240)	(6.766)
Country fixed effects	Yes	Yes	Yes	Yes	Yes	Yes	Yes	Yes	Yes	Yes	Yes	Yes	Yes
No. of regions	206	205	205	204	174	180	162	195	172	172	197	197	197
R^2	0.419	0.421	0.418	0.438	0.351	0.354	0.355	0.410	0.454	0.501	0.487	0.437	0.488
Adjusted R^2	0.345	0.347	0.345	0.366	0.271	0.268	0.269	0.335	0.386	0.439	0.418	0.362	0.416

Robust standard errors in parentheses
*** p<0.01, ** p<0.05, * p<0.1

Table C3. Excess mortality (per 100,000 inhabitants) and institutional variables

Dependent variable:	(1)	(2)	(3)	(4)	(5)	(6)	(7)
Excess mortality in the first six months of 2020	***Autonomy***	***Government quality***			***Social capital & trust***		
		Regional	*National*	*Both*	*Interaction*	*Trust*	*Bridging*
	OLS	OLS	OLS	OLS	OLS	OLS	OLS
Regional autonomy 2016	-1.602***						
	(0.472)						
Regional government quality 2017		23.135**		12.302			
		(10.008)		(10.095)			
Change in regional government quality 2010-2017		2.219		11.086			
		(12.788)		(11.129)			
National government effectiveness 2018			7.853	-12.143			
			(5.884)	(19.033)			
Change in national government effectiveness 1998-2018			-73.403***	-75.624***			
			(10.022)	(13.286)			
Frequency of meeting friends (once a week or more) 2016					-0.608		
					(0.428)		
Generalised trust 2016						9.693	
						(13.067)	
Bridging social networks 2016							-23.555***
							(8.281)
Country fixed effects	Yes	Yes	No	No	Yes	Yes	Yes
No. of regions	200	189	206	189	168	171	171
R^2	0.421	0.458	0.215	0.257	0.374	0.430	0.443
Adjusted R^2	0.349	0.386	0.208	0.241	0.303	0.366	0.381

Robust standard errors in parentheses
*** p<0.01, ** p<0.05, * p<0.1

Table C4. Full model (excess mortality per 100,000 inhabitants)

Dependent variable: Excess mortality in the first six months of 2020	(1) OLS	(2) OLS	(3) OLS	(4) OLS	(5) OLS	(6) OLS	(7) OLS	(8) OLS
Population 2019 (ln)	1.342	4.646	4.905	-4.848	8.528	4.692	2.983	5.362
	(6.343)	(6.623)	(7.174)	(12.643)	(8.446)	(7.457)	(7.936)	(7.820)
GDP per capita 2018 (ln)	50.398***	51.367**	34.266**	29.579*	21.089	31.880*	68.920***	29.494*
	(13.832)	(22.135)	(14.720)	(15.712)	(14.943)	(19.231)	(17.827)	(17.060)
Population density 2018 (ln)	-4.953*	4.439	-10.523**	-7.550	-14.599***	-10.193*	-5.566	-10.176*
	(2.648)	(4.918)	(5.249)	(5.547)	(5.366)	(5.549)	(6.386)	(5.336)
Air passengers 2018 (ln)	-1.144	-0.685	-0.797	-1.173	-2.038	-0.754	-0.807	-1.649
	(1.224)	(1.171)	(1.480)	(1.779)	(1.715)	(1.534)	(1.690)	(1.627)
Accessibility by road 2014 (ln)	26.720***	23.103**	33.017***	25.290***	27.242***	35.208***	31.916***	43.868***
	(7.584)	(9.292)	(7.708)	(8.310)	(8.945)	(11.785)	(9.676)	(11.644)
Share of population over 65 2019		12.673	-24.944	-90.140	-238.215	-30.201	136.903	-30.822
		(130.269)	(138.335)	(148.315)	(175.587)	(137.344)	(172.889)	(146.759)
Share of adults with higher education 2017		-1.135*	0.790*	0.716	0.619	0.813	1.080*	1.237**
		(0.610)	(0.475)	(0.508)	(0.448)	(0.513)	(0.586)	(0.522)
Average temperature 2019		-8.879***	-3.181*	-4.364**	0.478	-3.415*	-3.651	-4.536*
		(2.704)	(1.753)	(2.074)	(2.464)	(1.952)	(2.319)	(2.328)
Average Precipitation 2019		-11.420*	-11.374*	-11.692*	-7.795	-11.471*	-20.685***	-13.893*
		(6.056)	(6.316)	(6.807)	(6.477)	(6.360)	(7.467)	(7.053)
Regional autonomy 2016			-2.030***	-1.583***	-1.950***	-1.925**	-3.306**	-1.302
			(0.581)	(0.590)	(0.555)	(0.743)	(1.372)	(1.105)
Frequency of meeting friends (once a week or more) 2016			1.288***	1.452***	0.832**	1.266***	1.209***	0.952**
			(0.338)	(0.375)	(0.381)	(0.358)	(0.414)	(0.397)
Change in regional government quality 2010-2017			22.870**	18.135*	12.011	22.361**	35.942***	20.650
			(10.044)	(10.325)	(10.588)	(10.654)	(13.620)	(12.569)
National government effectiveness 2018			-50.860***	-67.302***	-50.122***	-53.813***	-49.293***	-43.006**
			(15.867)	(17.747)	(16.886)	(17.066)	(17.328)	(17.662)
Change in national government effectiveness 1998-2018			-89.800***	-84.726***	-60.969***	-86.337***	-148.191***	-75.650**
			(15.631)	(15.409)	(16.630)	(19.322)	(49.623)	(33.344)
Generalised trust 2016			13.448	27.418	-2.378	12.434	-10.802	-2.221

			(17.574)	(18.660)	(19.275)	(18.616)	(25.142)	(18.823)
Bridging social networks 2016			-16.990**	-25.465***	-24.951***	-15.903*	-20.552*	-14.842*
			(8.077)	(8.909)	(8.418)	(9.120)	(10.617)	(8.254)
Total household carbon footprint 2010 (ln)				14.799*				
				(8.347)				
Average household carbon footprint 2010 (ln)					114.223***			
					(31.295)			
Air pollution 2016 (ln)						-7.970		
						(30.594)		
Doctors per capita 2017 (ln)							-34.584**	
							(15.458)	
Hospital beds per capita 2017 (ln)								-33.916**
								(14.127)
Country fixed effects	Yes	Yes	No	No	No	No	No	No
No. of regions	187	182	148	128	128	148	122	124
R^2	0.578	0.635	0.459	0.492	0.525	0.460	0.457	0.472
Adjusted R^2	0.513	0.565	0.393	0.414	0.451	0.389	0.368	0.387
F test			8.897	7.801	10.19	8.504	5.385	6.762

Robust standard errors in parentheses
*** p<0.01, ** p<0.05, * p<0.1

Appendix D

Table D1. t-test on Pre-and Covid mortality rates at regional level (H_0: pre_covid_pop=covid_pop)

	obs	Mean1	Mean2	dif	St_Err	t_value	p_value
Abruzzo	26	0	0	0	0	-0.15	0.873
Agder og Rogaland	27	0	0	0	0	0.55	0.581
Alentejo	27	0.001	0.001	0	0	0.1	0.921
Algarve	27	0	0	0	0	-0.25	0.8
Alsace	27	0	0	0	0	-2.55	0.017
Andalucia	27	0	0	0	0	-0.3	0.782
Aquitaine	27	0	0	0	0	1.85	0.077
Aragon	27	0	0	0	0	-1.7	0.105
Auvergne	27	0	0	0	0	2.9	0.008
Baden-Wuttemberg	22	0	0	0	0	-1.05	0.313
Basilicata	26	0	0	0	0	0.85	0.408
Basse-Normandie	27	0	0	0	0	0	0.996
Bayern	22	0	0	0	0	-1.5	0.142
Berlin	22	0	0	0	0	-0.4	0.679
Bourgogne	27	0	0	0	0	-1	0.328
Brandeburg	22	0.001	0.001	0	0	0.05	0.943
Bratislavský kraj	27	0	0	0	0	1.6	0.123
Bremen	22	0	0.001	0	0	-4.9	0
Bretagne	27	0	0	0	0	-0.3	0.762
Budapest	27	0	0	0	0	0.4	0.688
Burgenland (AT)	27	0	0	0	0	-0.05	0.963
Calabria	26	0	0	0	0	0.75	0.447
Campania	26	0	0	0	0	3	0.005
Canarias (ES)	27	0	0	0	0	-1.85	0.076
Cantabria	27	0	0	0	0	-0.8	0.42
Castilla y Leon	27	0	0.001	0	0	-2.25	0.035
Castilla-La Mancha	27	0	0.001	0	0	-2.15	0.042
Cataluna	27	0	0	0	0	-2.2	0.036
Centre	27	0	0	0	0	-1.5	0.14
Centro (PT)	27	0.001	0.001	0	0	-0.6	0.545
Champagne-Ardenne	27	0	0	0	0	-1.3	0.199
Ciudad Autonoma de Ceuta	27	0	0	0	0	-2.2	0.036
Ciudad Autonoma de Melilla	27	0	0	0	0	0.9	0.365
Comunidad Foral de Navarra	27	0	0	0	0	-1.95	0.063
Comunidad Valenciana	27	0	0	0	0	-1.8	0.085
Comunidad de Madrid	27	0	0	0	0	-2.4	0.024
Corse	27	0	0	0	0	-0.85	0.416
Dolnoslaskie	26	0	0	0	0	-0.35	0.717
Drenthe	27	0	0	0	0	-0.35	0.728
De-Alfold	27	0.001	0.001	0	0	3.9	0.001
Dél-Dunántúl	27	0.001	0.001	0	0	2.7	0.011
East Midlands (UK)	27	0	0	0	0	-3.15	0.004
East of England	27	0	0	0	0	-2.55	0.017
Emilia-Romagna	26	0	0.001	0	0	-2.5	0.019
Espace Mittelland	27	0	0	0	0	0.85	0.415
Estonia	27	0	0	0	0	0.7	0.477
Etela-Suomi	27	0	0	0	0	0.75	0.468
Extremadura	27	0	0	0	0	-1.75	0.092
Flevoland	27	0	0	0	0	-2	0.056
Franche-Comtè	27	0	0	0	0	-1.85	0.077
Friesland (NL)	27	0	0	0	0	0	0.998
Friuli-Venezia Giulia	26	0	0	0	0	-0.6	0.549
Galicia	27	0	0	0	0	0.7	0.504
Gelderland	27	0	0	0	0	-2.1	0.044
Groningen	27	0	0	0	0	1.05	0.309
Guadeloupe	27	0	0	0	0	-2.65	0.013
Guyane	27	0	0	0	0	0.85	0.396
Hamburg	22	0	0	0	0	-4.3	0.001

Haute-Normandie	27	0	0	0	0	-1.2	0.24
Hedmark og Oppland	27	0	0	0	0	2.2	0.038
Helsinki-Uusimaa	27	0	0	0	0	-3.55	0.002
Hessen	22	0	0	0	0	0.55	0.593
Hovedstaden	27	0	0	0	0	0.65	0.532
Illes Balears	27	0	0	0	0	0.95	0.351
Jihovýchod	27	0	0	0	0	0.65	0.51
Jihozapad	27	0	0	0	0	0.2	0.827
Kujawsko-Pomorskie	26	0	0	0	0	-1.2	0.234
Karnten	27	0	0	0	0	0.75	0.467
Közép-Dunántúl	27	0.001	0.001	0	0	1	0.316
La Rioja	27	0	0	0	0	-2.1	0.043
La Reunion	27	0	0	0	0	-2.2	0.037
Languedoc-Roussillon	27	0	0	0	0	-0.6	0.567
Latvija	27	0.001	0.001	0	0	3	0.005
Lazio	26	0	0	0	0	4.15	0.001
Liguria	26	0.001	0.001	0	0	-1.7	0.099
Limburg (NL)	27	0	0.001	0	0	-2.7	0.013
Limousin	27	0.001	0.001	0	0	2.1	0.046
Lombardia	26	0	0.001	0	0	-2.75	0.011
London	27	0	0	0	0	-2.5	0.019
Lorraine	27	0	0	0	0	-1.95	0.06
Lubelskie	26	0	0	0	0	0.95	0.344
Lubuskie	26	0	0	0	0	-0.1	0.94
Luxembourg	26	0	0	0	0	-0.55	0.597
Lansi-Suomi	27	0	0	0	0	0.05	0.941
Lodzkie	26	0.001	0	0	0	2	0.058
Malopolskie	26	0	0	0	0	-0.8	0.42
Marche	26	0	0	0	0	-1.95	0.06
Martinique	27	0	0	0	0	-1.05	0.3
Mayotte	27	0	0	0	0	-4.1	0.001
Mazowiecki regiona	26	0	0	0	0	-1.85	0.077
Mecklenburg-Vorpom~n	22	0.001	0.001	0	0	1.45	0.163
Mellersta Norrland	27	0	0	0	0	-2	0.054
Midi-Pyreneees	27	0	0	0	0	1.65	0.113
Midtjylland	27	0	0	0	0	0.75	0.471
Molise	26	0	0	0	0	1.35	0.193
Moravskoslezsko	27	0	0	0	0	-1.1	0.276
Niedersachsen	22	0	0	0	0	1.55	0.136
Niederosterreich	27	0	0	0	0	-1.6	0.128
Noord-Brabant	27	0	0	0	0	-2.35	0.028
Noord-Holland	27	0	0	0	0	-1.35	0.185
Nord	27	0	0	0	0	1.55	0.139
Nord - Pas-de-Calais	27	0	0	0	0	-0.9	0.382
Nordjylland	27	0	0	0	0	1.4	0.169
Nordrhein-Westfalen	22	0	0	0	0	1.1	0.279
Nordwestschweiz	27	0	0	0	0	-0.05	0.972
Norra Mellansverige	27	0	0	0	0	-1.05	0.297
Norte	27	0	0	0	0	-2.7	0.012
North East (UK)	27	0	0	0	0	-2.6	0.016
North West (UK)	27	0	0	0	0	-2.9	0.007
Northern Ireland	27	0	0	0	0	-2.55	0.018
Nyugat-Dunantul	27	0.001	0	0	0	1.65	0.112
Oberosterreich	27	0	0	0	0	-0.2	0.836
Opolskie	26	0	0	0	0	0.6	0.556
Oslo og Akershus	27	0	0	0	0	0.65	0.519
Ostschweiz	27	0	0	0	0	0.3	0.757
Overijssel	27	0	0	0	0	-1.95	0.06
Pays de la Loire	27	0	0	0	0	-1.8	0.082
Pais Vasco	27	0	0	0	0	-1.8	0.085
Pest	27	0	0	0	0	2.15	0.041
Picardie	27	0	0	0	0	-2.7	0.013
Piemonte	26	0	0.001	0	0	-1.8	0.082
Podkarpackie	26	0	0	0	0	0	0.984

Podlaskie	26	0	0	0	0	1.25	0.216
Pohjois- ja Itä-Suomi	27	0	0	0	0	0.45	0.649
Poitou-Charentes	27	0	0	0	0	1.15	0.268
Pomorskie	26	0	0	0	0	-1.4	0.18
Praha	27	0	0	0	0	0.55	0.575
Principado de Asturias	27	0.001	0.001	0	0	-1.1	0.282
Provence-Alpes-Côte d'Azur	27	0	0	0	0	-0.7	0.482
Provincia Autonoma di Bolazano	26	0	0	0	0	-2.8	0.009
Provincia Autonoma di Trento	26	0	0	0	0	-2.65	0.014
Puglia	26	0	0	0	0	-1.45	0.164
Region Juzne i Istocne Srbije	27	0	0	0	0	0.35	0.729
Region Sumadije i Zapadne Srbije	27	0	0	0	0	-0.1	0.923
Region de Murcia	27	0	0	0	0	-1.05	0.296
Rheinland-Pfalz	22	0	0	0	0	2.9	0.009
Rhone-Alpes	27	0	0	0	0	-2.15	0.043
Region de Bruxelles	27	0	0	0	0	-1.95	0.061
Region lemanique	27	0	0	0	0	-1.65	0.111
Region wallonne	27	0	0	0	0	-1.5	0.144
Saarland	22	0.001	0.001	0	0	0.1	0.917
Sachsen	22	0.001	0.001	0	0	0.75	0.458
Sachsen-Anhalt	22	0.001	0.001	0	0	4.6	0
Salzburg	27	0	0	0	0	-1.85	0.076
Sardegna	26	0	0	0	0	-0.65	0.512
Schleswig-Holstein	22	0	0	0	0	4.7	0
Scotland	27	0	0	0	0	-2.75	0.011
Severen tsentralen	27	0.001	0.001	0	0	3.35	0.003
Severoiztochen	27	0.001	0.001	0	0	3.1	0.004
Severovýchod	27	0	0	0	0	1.45	0.161
Severozapaden	27	0.001	0.001	0	0	3.85	0.001
Severozapad	27	0	0	0	0	0.5	0.629
Sicilia	26	0	0	0	0	2.65	0.013
Sjælland	27	0	0	0	0	-1.5	0.146
Slaskie	26	0	0	0	0	-0.95	0.354
Smaland med oarna	27	0	0	0	0	-1.85	0.075
Sostines regionas	27	0	0	0	0	1.25	0.223
South East (UK)	27	0	0	0	0	-2.8	0.009
South West (UK)	27	0	0	0	0	-2.2	0.037
Steiermark	27	0	0	0	0	-1.75	0.095
Stockholm	27	0	0	0	0	-3.15	0.004
Stredne Slovensko	27	0	0	0	0	2.75	0.011
Stredne Cechy	27	0	0	0	0	1.3	0.212
Stredne Morava	27	0	0	0	0	-0.15	0.893
Swietokrzyskie	26	0	0	0	0	-0.75	0.447
Syddanmark	27	0	0	0	0	2.4	0.024
Sydsverige	27	0	0	0	0	0.45	0.641
Sor	27	0	0	0	0	0.5	0.606
Thuringen	22	0.001	0.001	0	0	2.55	0.019
Ticino	27	0	0	0	0	-2.15	0.041
Tirol	27	0	0	0	0	-1.05	0.314
Toscana	26	0	0	0	0	-0.25	0.816
Trondelag	27	0	0	0	0	1.3	0.205
Umbria	26	0	0	0	0	1.65	0.114
Utrecht	27	0	0	0	0	-2.65	0.013
Valle d'Aosta	26	0	0.001	0	0	-1.7	0.106
Veneto	26	0	0	0	0	-2.55	0.017
Vestlandet	27	0	0	0	0	1.65	0.114
Vidurio ir vakaru Lietuvos regionas	27	0.001	0.001	0	0	2.7	0.012
Vlaams Gewest	27	0	0	0	0	-1.85	0.075
Vorarlberg	27	0	0	0	0	-0.35	0.742
Vastsverige	27	0	0	0	0	-2.25	0.033
Vychodné Slovensko	27	0	0	0	0	1.8	0.081
Wales	27	0	0	0	0	-2.55	0.017
Warminsko-Mazurski	26	0	0	0	0	1.6	0.119
Warszawski stolecz	26	0	0	0	0	-0.9	0.374

West Midlands (UK)	27	0	0	0	0	-2.8	0.009
Wielkopolskie	26	0	0	0	0	-0.15	0.866
Wien	27	0	0	0	0	-2.45	0.022
Yorkshire and The Humber	27	0	0	0	0	-2.75	0.011
Yugoiztochen	27	0.001	0.001	0	0	2.6	0.015
Yugozapaden	27	0.001	0.001	0	0	2.85	0.008
Yuzhen tsentralen	27	0.001	0.001	0	0	2.65	0.013
Zachodniopomorskie	26	0	0	0	0	-2.1	0.043
Zeeland	27	0	0	0	0	-1.55	0.137
Zentralschweiz	27	0	0	0	0	0.35	0.739
Zuid-Holland	27	0	0	0	0	-1.6	0.117
Zapadné Slovensko	27	0	0	0	0	2.35	0.028
Zùrich	27	0	0	0	0	1.55	0.133
Årea Metropolitana de Lisboa	27	0	0	0	0	-1.8	0.083
Aland	27	0	0	0	0	0.7	0.484
Eszak-Alfold	27	0.001	0.001	0	0	2.55	0.017
Eszak-Magyarorszag	27	0.001	0.001	0	0	6.2	0
Ile de France	27	0	0	0	0	-2.2	0.036
Ostra Mellansverige	27	0	0	0	0	-1.95	0.06
Ovre Norrland	27	0	0	0	0	-0.05	0.958

Table D2. Correlation among the stringency index and the quality of government.

Variables	(1)	(2)	(3)	(4)
(1) Stringency Index	1.000			
(2) Regional government quality 2017	-0.362	1.000		
(3) National government effectiveness 2018	-0.349	0.893	1.000	
(4) Change in national government effectiveness 1998-2018	-0.313	-0.101	-0.055	1.000

Table D3. Interactions with regional government and RAI

Dependent variable: Excess mortality in the first six months of 2020	(1) OLS	(2) OLS	(3) OLS	(4) OLS	(5) OLS
Population 2019 (ln)	-0.762	1.378	0.926	0.691	0.953
	(2.362)	(1.614)	(1.412)	(1.488)	(1.486)
GDP per capita 2018 (ln)	8.048**	4.443	8.162**	15.971***	6.707*
	(3.083)	(3.042)	(3.815)	(3.655)	(3.604)
Population density 2018 (ln)	-1.542	-2.444**	-1.945*	-1.115	-1.729
	(1.152)	(1.086)	(1.099)	(1.289)	(1.078)
Air passengers 2018 (ln)	-0.286	-0.409	-0.182	-0.145	-0.318
	(0.349)	(0.346)	(0.300)	(0.334)	(0.315)
Accessibility by road 2014 (ln)	5.677***	5.308**	8.011***	7.205***	9.568***
	(1.802)	(2.157)	(2.663)	(2.225)	(2.780)
Share of population over 65 2019	-36.409	-69.195*	-29.576	13.668	-21.018
	(27.105)	(35.172)	(25.394)	(30.280)	(26.244)
Share of adults with higher education 2017	0.152	0.098	0.168	0.184	0.246**
	(0.108)	(0.101)	(0.107)	(0.119)	(0.109)
Average temperature 2019	-0.637	0.711	-0.593	-0.713	-0.998**
	(0.408)	(0.475)	(0.409)	(0.448)	(0.478)
Average precipitation 2019	-2.746**	-0.829	-2.855**	-4.786***	-3.442**
	(1.353)	(1.212)	(1.313)	(1.562)	(1.480)
Change in regional government quality 2010-2017	6.944*	3.964	8.211**	15.143**	4.641
	(3.988)	(4.268)	(3.787)	(5.812)	(4.392)
Regional autonomy 2016	-0.396***	-0.491***	-0.429***	-0.934***	-0.390*
	(0.112)	(0.112)	(0.149)	(0.296)	(0.215)
Change in regional government quality 2010-2017*Regional autonomy 2016	-0.256	-0.148	-0.299	-0.474	-0.075
	(0.252)	(0.239)	(0.242)	(0.288)	(0.243)
Frequency of meeting friends (once a week or more) 2016	0.225***	0.016	0.187***	0.175**	0.172**
	(0.069)	(0.069)	(0.064)	(0.079)	(0.075)
National government effectiveness 2018	-11.984***		-10.710***	-7.172**	-7.609**
	(3.645)		(3.856)	(3.488)	(3.562)
Change in national government effectiveness 1998-2018	-17.349***	-15.399***	-16.534***	-36.745***	-18.153***
	(2.907)	(3.199)	(3.729)	(11.158)	(6.860)
Generalised trust 2016	5.294	-5.321	2.039	-2.928	-0.676
	(3.397)	(4.194)	(3.410)	(4.672)	(3.557)
Bridging social networks 2016	-5.373***	-6.506***	-3.275*	-4.100*	-2.811
	(1.955)	(2.285)	(1.859)	(2.241)	(1.832)
Total household carbon footprint 2010 (ln)	2.692*				
	(1.616)				
Average household carbon footprint 2010 (ln)		22.306***			
		(7.206)			
Air pollution 2016 (ln)			-4.149		
			(6.741)		
Doctors per capita 2017 (ln)				-5.536*	
				(3.094)	
Hospital beds per capita 2017 (ln)					-6.809**
					(3.113)
Observations	128	128	148	122	124
R-squared	0.519	0.503	0.498	0.486	0.491
Adjusted R-squared	0.440	0.426	0.427	0.397	0.404

Clustered standard errors at country level in parentheses
*** p<0.01, ** p<0.05, * p<0.1

Table D4. Interactions with regional government and trust

Dependent variable:<br>Excess mortality in the first six months of 2020	(1)<br>OLS	(2)<br>OLS	(3)<br>OLS	(4)<br>OLS	(5)<br>OLS
Population 2019 (ln)	-0.792	1.346	0.920	0.741	0.918
	(2.365)	(1.609)	(1.411)	(1.485)	(1.479)
GDP per capita 2018 (ln)	8.095**	4.764	7.983**	17.450***	7.345**
	(3.287)	(3.104)	(3.887)	(4.193)	(3.637)
Population density 2018 (ln)	-1.451	-2.506**	-1.847*	-0.928	-1.743
	(1.122)	(1.102)	(1.079)	(1.293)	(1.094)
Air passengers 2018 (ln)	-0.275	-0.415	-0.172	-0.161	-0.325
	(0.351)	(0.354)	(0.302)	(0.340)	(0.316)
Accessibility by road 2014 (ln)	5.540***	5.254**	7.916***	6.667***	9.638***
	(1.837)	(2.221)	(2.747)	(2.156)	(2.805)
Share of population over 65 2019	-34.958	-69.352*	-28.820	20.271	-17.581
	(27.497)	(35.419)	(25.553)	(33.103)	(26.822)
Share of adults with higher education 2017	0.171	0.097	0.193*	0.227*	0.248**
	(0.106)	(0.097)	(0.105)	(0.120)	(0.109)
Average temperature 2019	-0.714*	0.776	-0.677*	-0.767*	-1.008**
	(0.379)	(0.493)	(0.387)	(0.428)	(0.467)
Average precipitation 2019	-2.817**	-0.844	-2.906**	-4.931***	-3.506**
	(1.349)	(1.197)	(1.318)	(1.565)	(1.464)
Change in regional government quality 2010-2017	2.345	0.662	3.069	5.562**	2.495
	(2.054)	(2.247)	(2.133)	(2.464)	(2.393)
Generalised trust 2016	4.779	-6.181	1.464	-4.546	-1.197
	(3.343)	(3.892)	(3.397)	(4.523)	(3.355)
Change in regional government quality 2010-2017*<br>Generalised trust 2016	-3.627	-8.175	-2.200	-13.194	-8.465
	(6.873)	(7.531)	(6.635)	(8.629)	(7.140)
Regional autonomy 2016	-0.386***	-0.508***	-0.410***	-0.896***	-0.387*
	(0.121)	(0.122)	(0.152)	(0.310)	(0.213)
Frequency of meeting friends (once a week or more) 2016	0.234***	0.002	0.196***	0.181**	0.163**
	(0.071)	(0.074)	(0.066)	(0.083)	(0.076)
National government effectiveness 2018	-12.521***		-11.288***	-7.611**	-7.518**
	(3.506)		(3.880)	(3.341)	(3.500)
Change in national government effectiveness 1998-2018	-17.251***	-15.608***	-16.172***	-37.248***	-18.214***
	(3.115)	(3.383)	(3.782)	(11.957)	(6.848)
Bridging social networks 2016	-5.251***	-6.477***	-3.070	-4.094*	-2.895
	(1.971)	(2.308)	(1.867)	(2.254)	(1.851)
Total household carbon footprint 2010 (ln)	2.739*				
	(1.603)				
Average household carbon footprint 2010 (ln)		24.574***			
		(7.010)			
Air pollution 2016 (ln)			-4.275		
			(6.829)		
Doctors per capita 2017 (ln)				-7.167**	
				(3.347)	
Hospital beds per capita 2017 (ln)					-7.265**
					(3.151)
Observations	128	128	148	122	124
R-squared	0.517	0.506	0.494	0.487	0.495
Adjusted R-squared	0.437	0.429	0.423	0.398	0.409

Clustered standard errors at country level in parentheses
*** p<0.01, ** p<0.05, * p<0.1

Table D5. Excess mortality and agglomeration, regional wealth, density, accessibility and connectivity

Dependent variable: Excess mortality in the first six months of 2020	(1)	(2)	(3)	(4)	(5)	(6)	(7)
	Agglomeration		***Regional wealth***	***Density***	***Accessibility & connectivity***		
	OLS	OLS	OLS	OLS	OLS	OLS	OLS
GDP 2018 (ln)		2.163*					
		(1.175)					
GDP 2018 (ln) - dummy		18.046					
		(12.314)					
Population 2019 (ln)	1.588						
	(1.186)						
GDP per capita 2018 (ln)			10.243**				
			(4.501)				
GDP per capita 2018 (ln) - dummy			104.581**				
			(47.973)				
Population density 2018 (ln)				1.960**			
				(0.876)			
Air passengers 2018 (ln)					0.155		-0.066
					(0.212)		(0.176)
Accessibility by road 2014 (ln)						7.156***	7.233***
						(1.552)	(1.541)
Accessibility by road 2014 (ln) - dummy						110.800***	111.981***
						(24.249)	(24.227)
Observations	206	206	206	206	206	206	206
R-squared	0.367	0.387	0.411	0.389	0.353	0.494	0.495
Adjusted R-squared	0.287	0.309	0.336	0.312	0.271	0.427	0.425

Clustered standard errors at regional (NUTS2) level in parentheses.
*** p<0.01, ** p<0.05, * p<0.1

Table D6. Excess mortality and age structure, education, readiness of the health system, and environmental conditions

Dependent variable:	(1)	(2)	(3)	(4)	(5)	(6)	(7)	(8)	(9)	(10)	(11)	(12)	(13)
Excess mortality in the first six months of 2020	***Age structure***			***Education***	***Health system***			***Air pollution***			***Climate***		
	OLS	OLS	OLS	OLS	OLS	OLS	OLS	OLS	OLS	OLS	OLS	OLS	OLS
Median population age 2019	-0.606*												
	(0.337)												
Share of population over 65 2019		-50.631*											
		(29.443)											
Share of population over 75 2019			-63.008										
			(46.960)										
Share of adults with higher education 2017				0.236***									
				(0.075)									
Doctors per capita 2017 (ln)					-2.521		-1.549						
					(5.288)		(3.952)						
Doctors per capita 2017 (ln) - dummy					8.902		-2.800						
					(32.235)		(23.641)						
Hospital beds per capita 2017 (ln)						-2.373	-3.407						
						(5.542)	(4.424)						
Hospital beds per capita 2017 (ln) - dummy						17.604	28.177						
						(29.857)	(25.096)						
Air pollution 2016 (ln)								7.993					
								(4.990)					
Air pollution 2016 (ln) - dummy								23.600*					
								(13.570)					
Total household carbon footprint 2010 (ln)									1.726				
									(1.271)				
Total household carbon footprint 2010 (ln) - dummy									44.794				
									(29.328)				
Average household carbon footprint 2010 (ln)										21.416***			
										(8.104)			
Average household carbon footprint 2010 (ln) - dummy										206.099***			
										(76.096)			
Average temperature 2019											-1.164***		-1.336***
											(0.342)		(0.388)
Average temperature 2019 - dummy											-16.856**		-21.543**
											(7.733)		(9.365)
Average precipitation 2019												0.515	-1.182
												(1.225)	(1.295)
Average precipitation 2019 - dummy												0.415	
												(5.749)	
Observations	206	205	205	204	206	206	206	206	206	206	206	206	206
R-squared	0.386	0.382	0.370	0.400	0.359	0.357	0.375	0.379	0.367	0.400	0.385	0.351	0.388
Adjusted R-squared	0.308	0.304	0.290	0.324	0.274	0.272	0.284	0.297	0.288	0.324	0.303	0.265	0.303

Clustered standard errors at regional (NUTS2) level in parentheses. *** p<0.01, ** p<0.05, * p<0.1

Table D7. Excess mortality and institutional variables

Dependent variable:	(1)	(2)	(3)	(4)	(5)	(6)	(7)
Excess mortality in the first six months of 2020	***Autonomy***	***Government quality***			***Social capital & trust***		
		Regional	*National*	*Both*	*Interaction*	*Trust*	*Bridging*
	OLS	OLS	OLS	OLS	OLS	OLS	OLS
Regional government quality 2017		3.859*		2.141			
		(2.198)		(2.228)			
Regional government quality 2017 - dummy		4.267		1.411			
		(14.331)		(4.736)			
Change in regional government quality 2010-2017		1.634		2.660			
		(2.688)		(2.403)			
Change in regional government quality 2010-2017 – dummy (omitted)		-		-			
Regional autonomy 2016	-0.363***						
	(0.108)						
Regional autonomy 2016 - dummy	1.218						
	(7.560)						
National government effectiveness 2018			1.475	-1.968			
			(1.141)	(4.057)			
Change in national government effectiveness 1998-2018			-13.398***	-13.480***			
			(2.121)	(3.076)			
Frequency of meeting friends (once a week or more) 2016					-0.072		
					(0.074)		
Frequency of meeting friends (once a week or more) 2016 - dummy					-5.340		
					(3.705)		
Generalised trust 2016						3.231	
						(2.371)	
Generalised trust 2016 - dummy						-1.941	
						(3.301)	
Bridging social networks 2016							-5.469***
							(1.834)
Bridging social networks 2016 - dummy							-1.303
							(3.270)
Observations	206	206	206	206	206	206	206
R-squared	0.360	0.375	0.167	0.193	0.355	0.355	0.368
Adjusted R-squared	0.276	0.289	0.159	0.173	0.269	0.269	0.284

Clustered standard errors at regional (NUTS2) level in parentheses.

*** p<0.01, ** p<0.05, * p<0.1

Table D8. Full model

Dependent variable: Excess mortality in the first six months of 2020	(1) OLS	(2) OLS	(3) OLS	(4) OLS	(5) OLS	(6) OLS	(7) OLS	(8) OLS
Population 2019 (ln)	-0.351	0.546	0.901	-0.706	1.255	1.040	0.851	1.123
	(1.237)	(1.205)	(1.217)	(1.914)	(1.242)	(1.257)	(1.205)	(1.166)
GDP per capita 2018 (ln)	6.380	10.471***	5.444*	5.006*	3.010	6.242*	5.984**	3.721
	(4.226)	(3.867)	(2.787)	(2.840)	(2.778)	(3.511)	(2.877)	(2.947)
GDP per capita 2018 (ln) - dummy	79.295*	114.804***	64.238**	59.032**	36.659	73.720*	69.594**	47.919*
	(42.769)	(41.027)	(28.886)	(29.046)	(28.102)	(42.425)	(29.741)	(28.799)
Population density 2018 (ln)	0.286	0.883	-0.805	-0.504	-0.721	-0.994	-0.528	-0.542
	(0.799)	(0.764)	(0.902)	(0.970)	(0.894)	(1.012)	(0.926)	(0.834)
Air passengers 2018 (ln)	-0.181	-0.113	-0.174	-0.145	-0.235	-0.181	-0.132	-0.173
	(0.230)	(0.216)	(0.240)	(0.250)	(0.248)	(0.247)	(0.238)	(0.234)
Accessibility by road 2014 (ln)	6.271***	5.432***	4.243***	3.963***	3.716***	4.192**	4.004***	4.749***
	(1.735)	(1.843)	(1.375)	(1.314)	(1.400)	(2.119)	(1.388)	(1.796)
Accessibility by road 2014 (ln) - dummy	99.978***	85.771***	46.713***	40.879**	44.968**	34.262	42.962**	55.008***
	(26.912)	(26.058)	(17.054)	(16.804)	(17.457)	(36.341)	(17.370)	(20.837)
Share of population over 65 2019		-15.505	-8.501	-11.919	-10.557	-14.811	-2.320	0.170
		(17.992)	(18.384)	(18.388)	(18.793)	(19.284)	(18.556)	(17.728)
Share of adults with higher education 2017		-0.197*	0.121	0.109	0.112	0.125	0.122	0.114
		(0.107)	(0.080)	(0.080)	(0.081)	(0.081)	(0.081)	(0.085)
Average temperature 2019		-1.539***	-0.311	-0.329	-0.082	-0.272	-0.343	-0.493
		(0.453)	(0.326)	(0.304)	(0.343)	(0.348)	(0.353)	(0.421)
Average temperature 2019 - dummy		-29.289***	10.997*	10.876*	15.193***	13.042	10.308*	10.595**
		(9.312)	(5.596)	(5.770)	(5.631)	(8.662)	(5.908)	(5.226)
Average precipitation 2019		-1.550	-0.731	-0.767	-0.611	-0.687	-0.738	-0.606
		(1.108)	(1.121)	(1.097)	(1.121)	(1.085)	(1.129)	(1.163)
Average precipitation 2019 – dummy (omitted)		-	-	-	-	-	-	-
Regional autonomy 2016			-0.274***	-0.234**	-0.283***	-0.293**	-0.250**	-0.160
			(0.101)	(0.104)	(0.103)	(0.136)	(0.109)	(0.110)
Regional autonomy 2016 - dummy			4.943	6.055*	1.013	4.474	5.354	2.224
			(3.289)	(3.392)	(3.445)	(3.245)	(3.278)	(3.151)
Frequency of meeting friends (once a week or more) 2016			0.180***	0.166***	0.143**	0.163***	0.198***	0.111*
			(0.055)	(0.055)	(0.059)	(0.057)	(0.057)	(0.058)
Frequency of meeting friends (once a week or more) 2016 - dummy			6.354*	5.906*	3.723	3.869	7.718**	2.117
			(3.367)	(3.426)	(3.338)	(3.348)	(3.487)	(3.378)
Change in regional government quality 2010-2017			3.606**	3.459*	2.872	3.642**	3.362*	3.103*
			(1.822)	(1.814)	(1.845)	(1.771)	(1.853)	(1.829)

Change in regional government quality 2010-2017 - dummy			-7.127*	-7.861*	-8.433**	-9.533	-6.033	-11.937*
			(3.616)	(4.030)	(3.582)	(9.917)	(4.615)	(6.173)
National government effectiveness 2018			-6.492**	-7.564***	-6.843**	-6.057*	-7.188**	-4.208
			(2.932)	(2.864)	(2.912)	(3.246)	(3.040)	(2.666)
Change in national government effectiveness 1998-2018			-12.227***	-12.082***	-10.512***	-13.597***	-11.674***	-8.834***
			(2.674)	(2.713)	(2.558)	(3.178)	(2.850)	(3.318)
Generalised trust 2016			2.044	2.149	0.619	3.231	2.466	-0.685
			(2.207)	(2.289)	(2.739)	(1.999)	(2.221)	(2.328)
Generalised trust 2016 - dummy			1.514	1.031	2.423	3.016	1.226	2.401
			(3.227)	(3.253)	(3.161)	(3.513)	(3.242)	(3.017)
Bridging social networks 2016			-3.889**	-4.346***	-4.609***	-4.239**	-4.418***	-4.550**
			(1.600)	(1.550)	(1.631)	(1.632)	(1.562)	(1.767)
Bridging social networks 2016 – dummy (omitted)			-	-	-	-	-	-
Total household carbon footprint 2010 (ln)				1.853				
				(1.409)				
Total household carbon footprint 2010 (ln) - dummy				46.076				
				(33.650)				
Average household carbon footprint 2010 (ln)					10.701**			
					(5.026)			
Average household carbon footprint 2010 (ln) - dummy					104.298**			
					(48.218)			
Average household carbon footprint 2010 (ln)						0.620		
						(5.221)		
Average household carbon footprint 2010 (ln) - dummy						12.009		
						(16.302)		
Doctors per capita 2017 (ln)							-3.512	
							(2.315)	
Doctors per capita 2017 (ln) - dummy							19.601	
							(13.142)	
Hospital beds per capita 2017 (ln)								-5.742**
								(2.337)
Hospital beds per capita 2017 (ln) - dummy								33.215***
								(11.197)
Observations	206	203	203	203	203	203	203	203
R-squared	0.516	0.610	0.430	0.439	0.446	0.447	0.435	0.459
Adjusted R-squared	0.439	0.534	0.357	0.360	0.368	0.369	0.355	0.382

Clustered standard errors at regional (NUTS2) level in parentheses.
*** p<0.01, ** p<0.05, * p<0.1

APPENDIX E

Table E1. Year-on-year variation in mortality rates at regional level by regions.

AUSTRIA

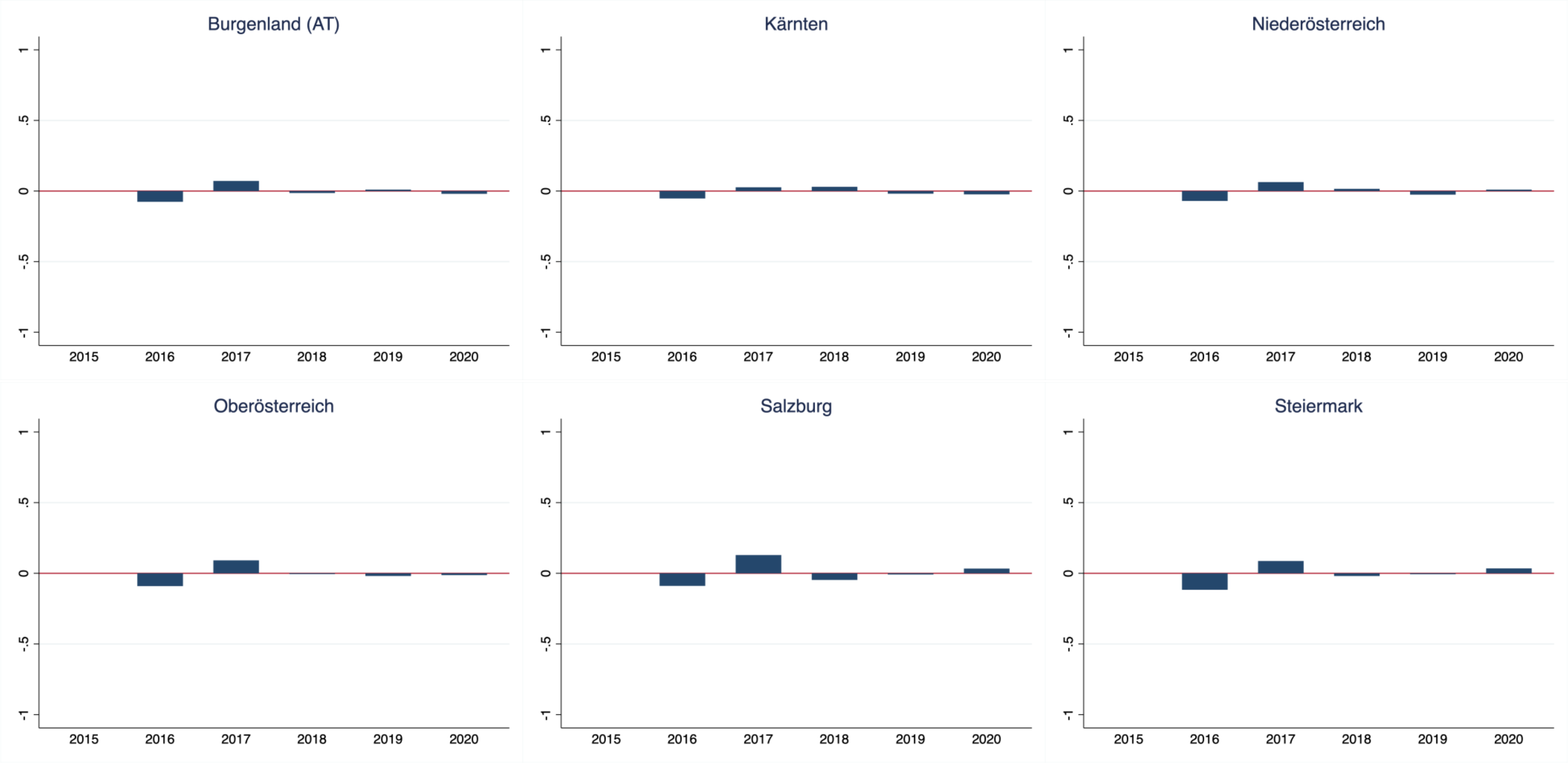

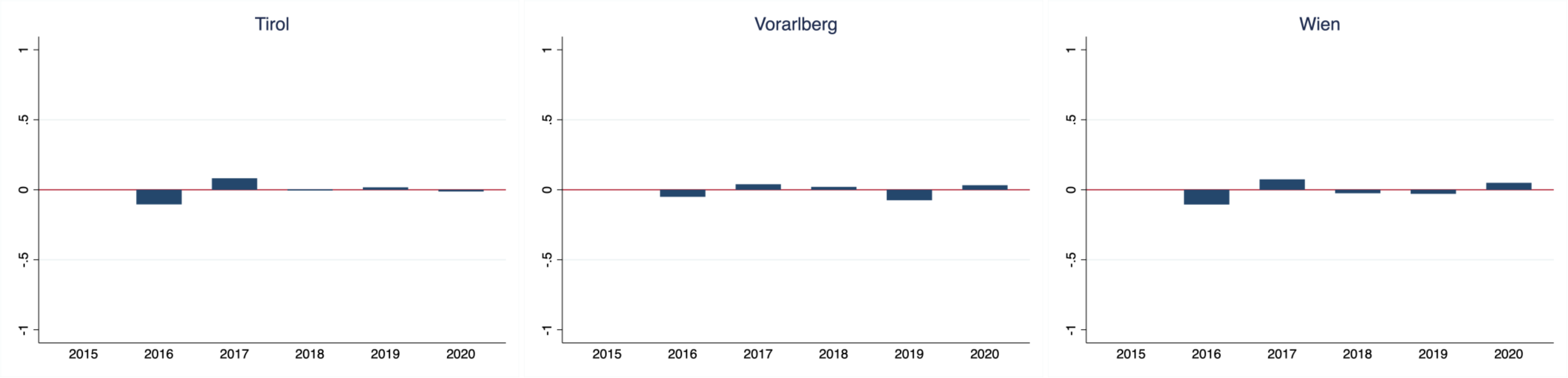
Tirol
Vorarlberg
Wien
1
.5
0
-.5
-1
2015
2016
2017
2018
2019
2020

BELGIUM

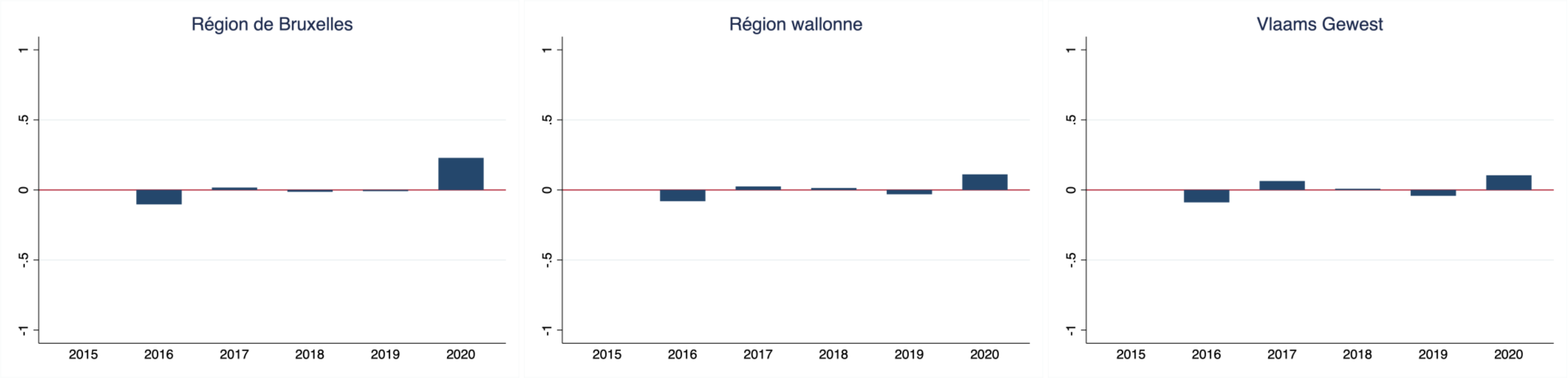
Région de Bruxelles
Région wallonne
Vlaams Gewest
1
.5
0
-.5
-1
2015
2016
2017
2018
2019
2020

BULGARIA

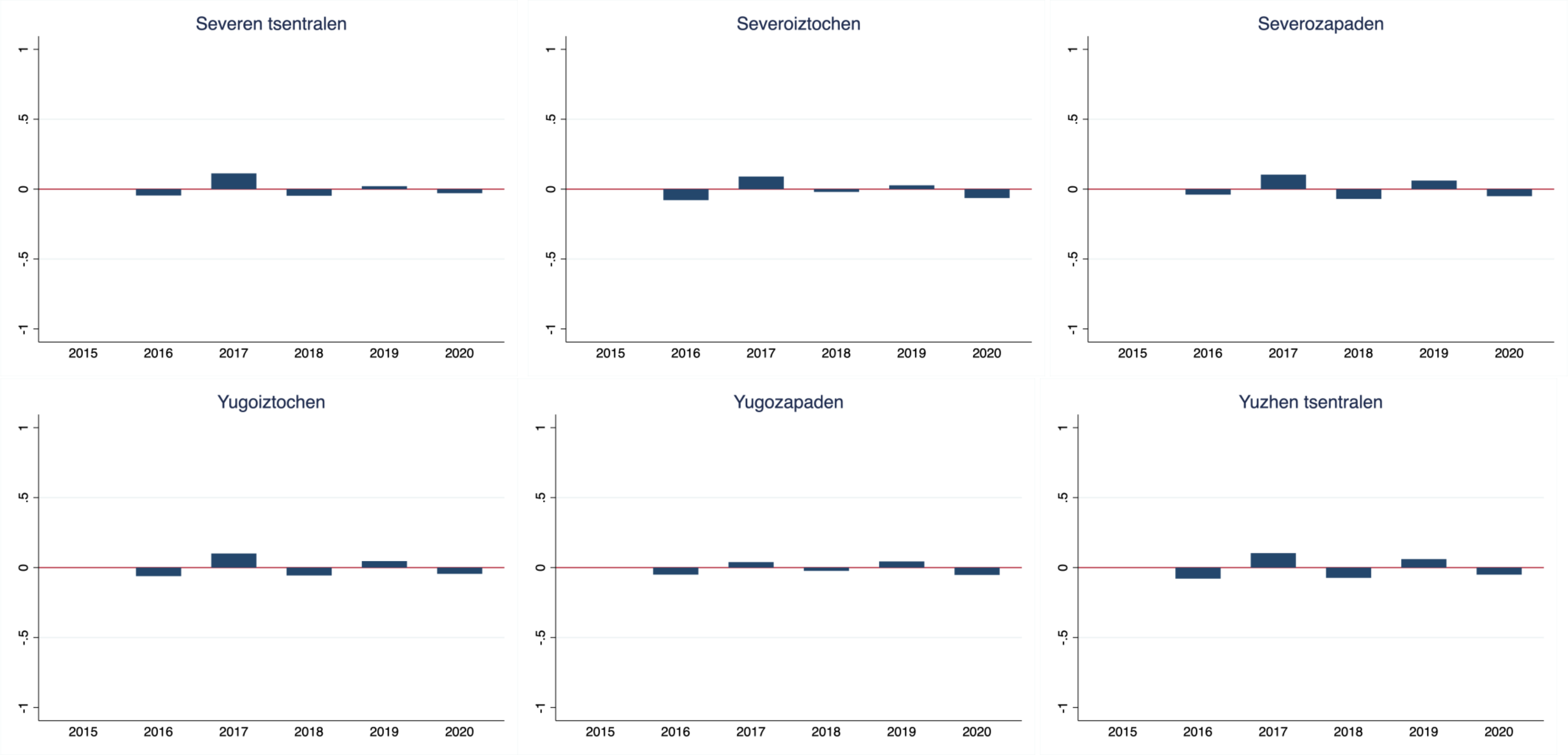
Severen tsentralen
Severoiztochen
Severozapaden
Yugoiztochen
Yugozapaden
Yuzhen tsentralen
1
.5
0
-.5
-1
2015
2016
2017
2018
2019
2020

CZECHIA

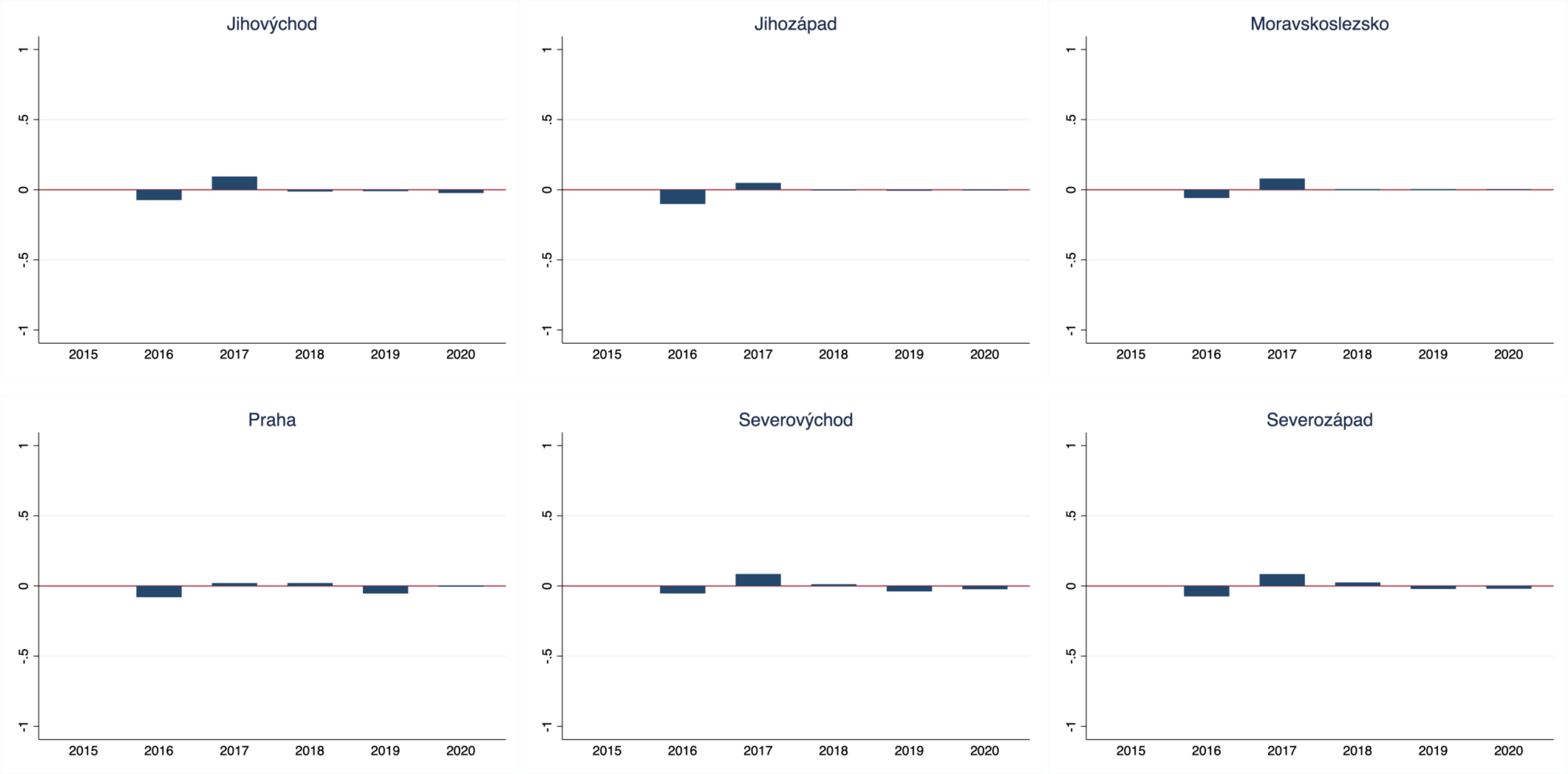
Jihovýchod
Jihozápad
Moravskoslezsko
Praha
Severovýchod
Severozápad
1
.5
0
-.5
-1
2015
2016
2017
2018
2019
2020

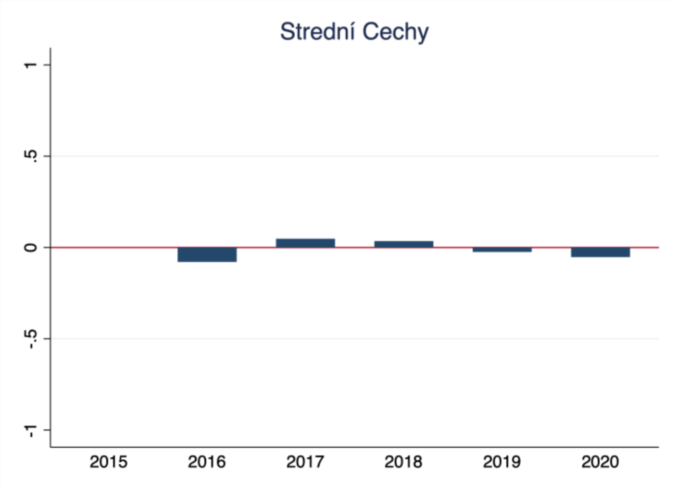
Strední Cechy
1
.5
0
-.5
-1
2015
2016
2017
2018
2019
2020

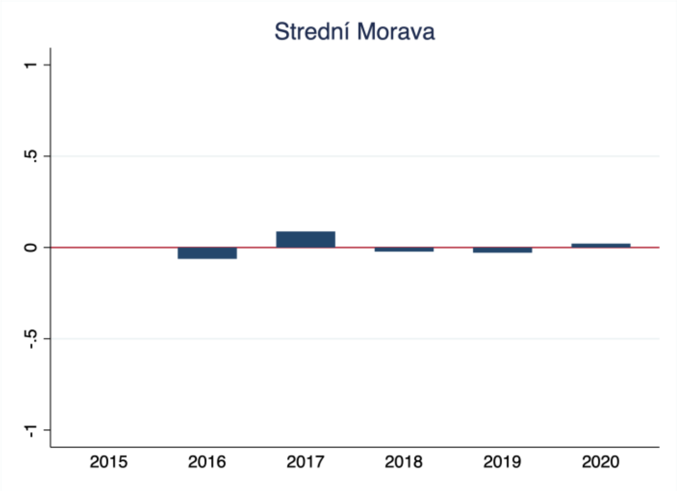
Strední Morava
1
.5
0
-.5
-1
2015
2016
2017
2018
2019
2020

GERMANY

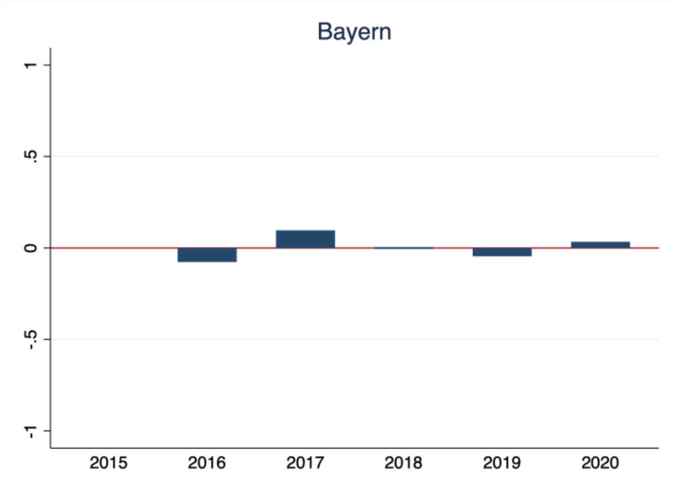
Bayern
1
.5
0
-.5
-1
2015
2016
2017
2018
2019
2020

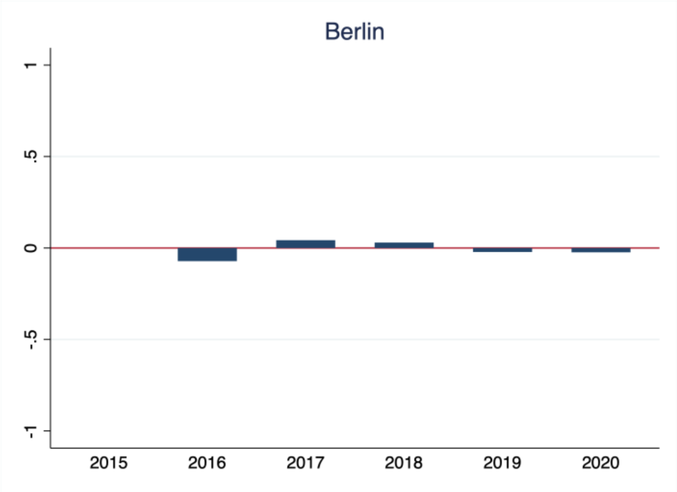
Berlin
1
.5
0
-.5
-1
2015
2016
2017
2018
2019
2020

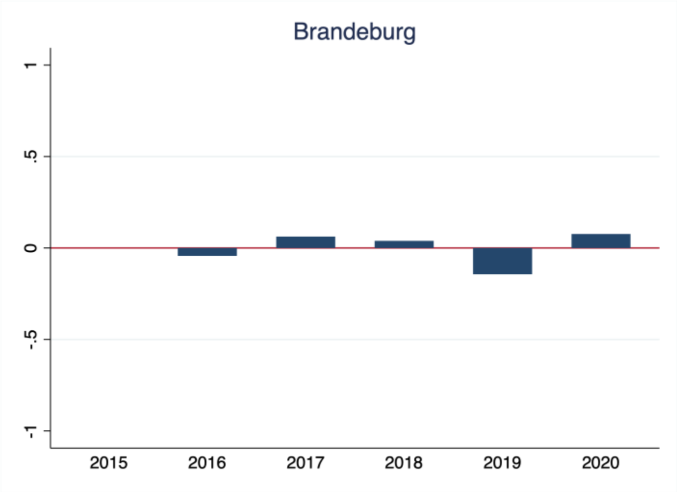
Brandeburg
1
.5
0
-.5
-1
2015
2016
2017
2018
2019
2020

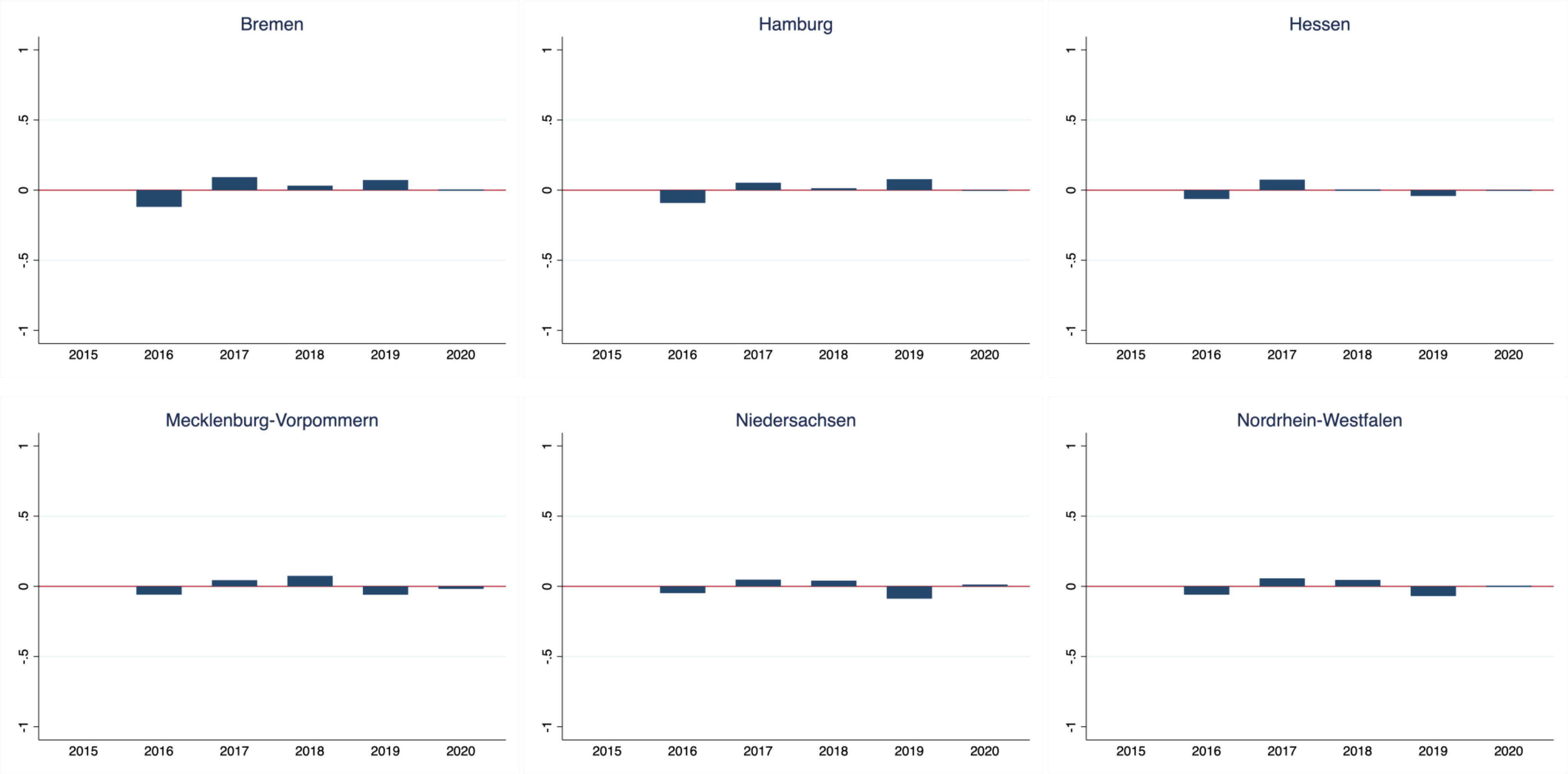
Bremen
Hamburg
Hessen
Mecklenburg-Vorpommern
Niedersachsen
Nordrhein-Westfalen
1
.5
0
-.5
-1
2015
2016
2017
2018
2019
2020

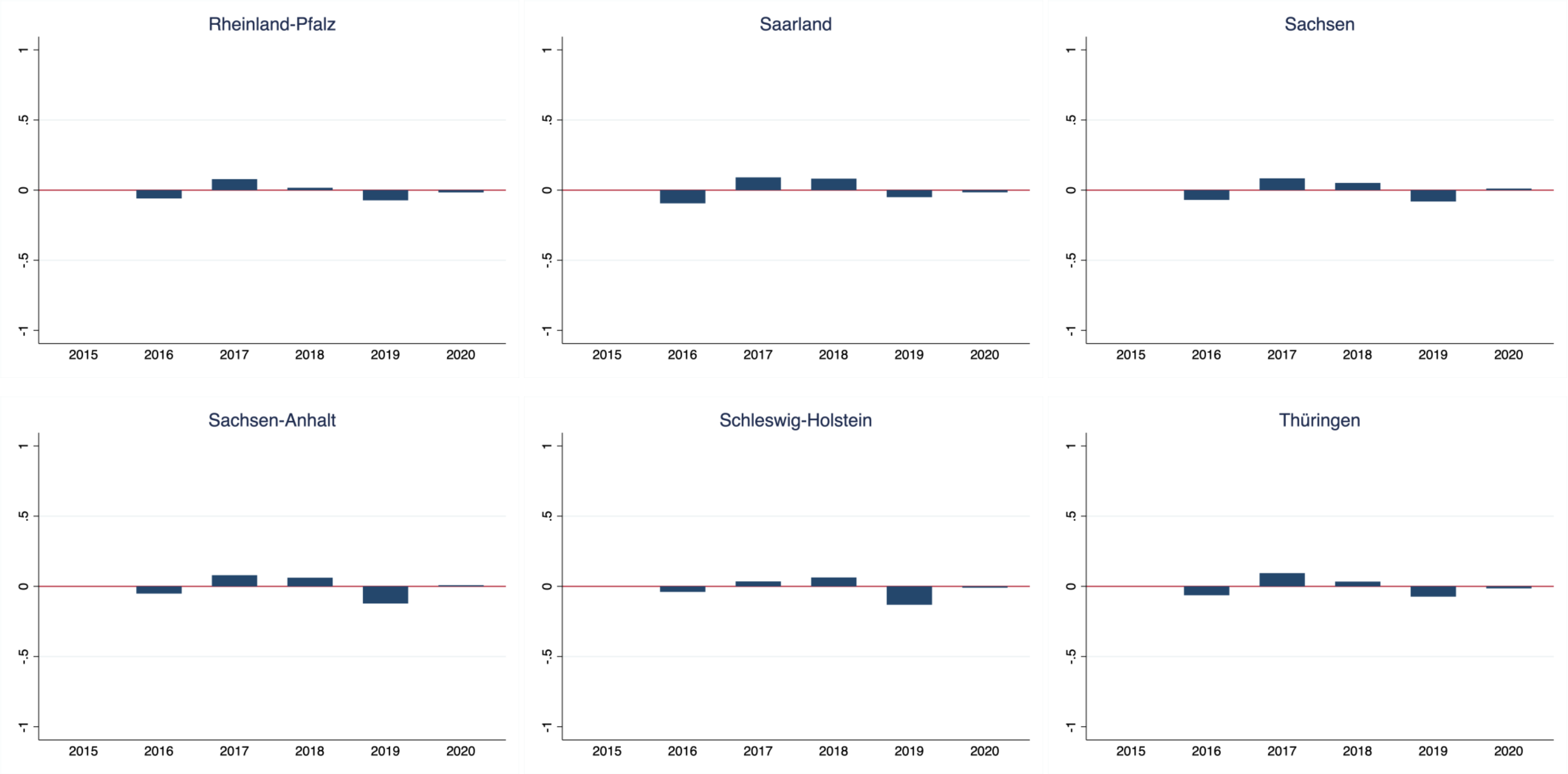
Rheinland-Pfalz
Saarland
Sachsen
Sachsen-Anhalt
Schleswig-Holstein
Thüringen
1
.5
0
-.5
-1
2015
2016
2017
2018
2019
2020

DENMARK

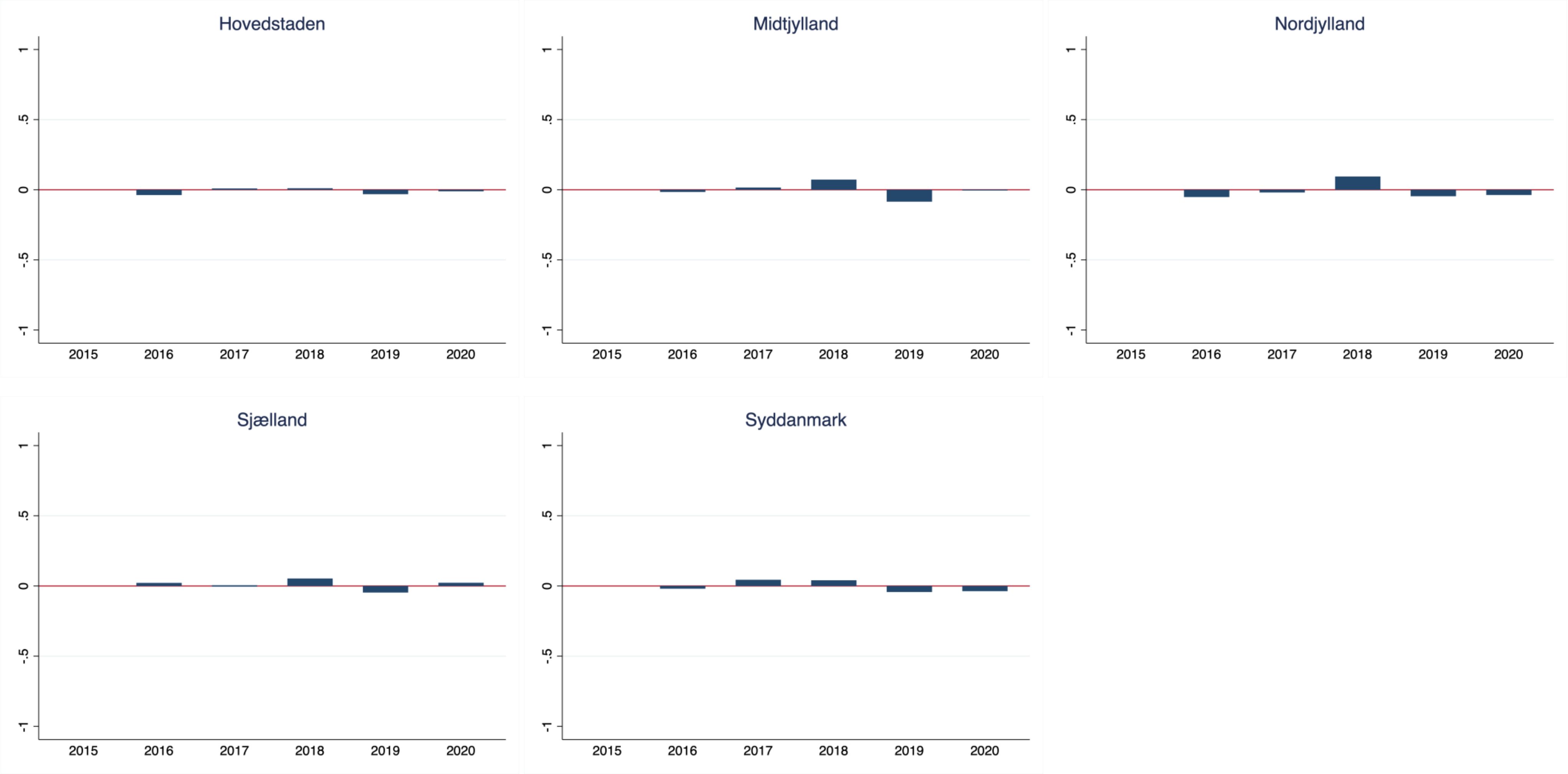
Hovedstaden
Midtjylland
Nordjylland
Sjælland
Syddanmark
1
.5
0
-.5
-1
2015
2016
2017
2018
2019
2020

ESTONIA

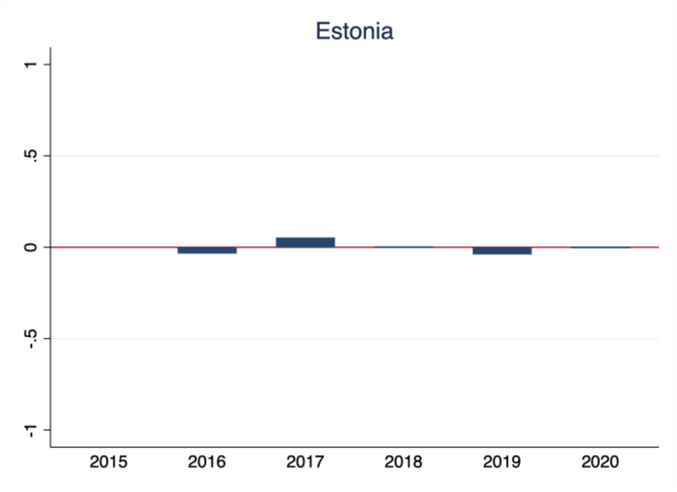

FINLAND

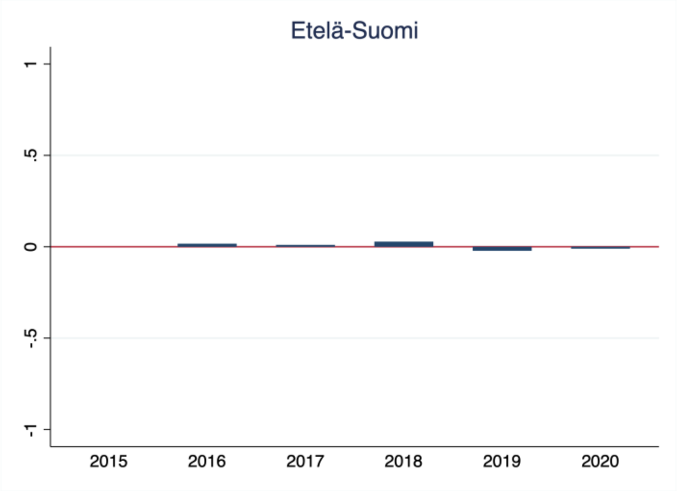

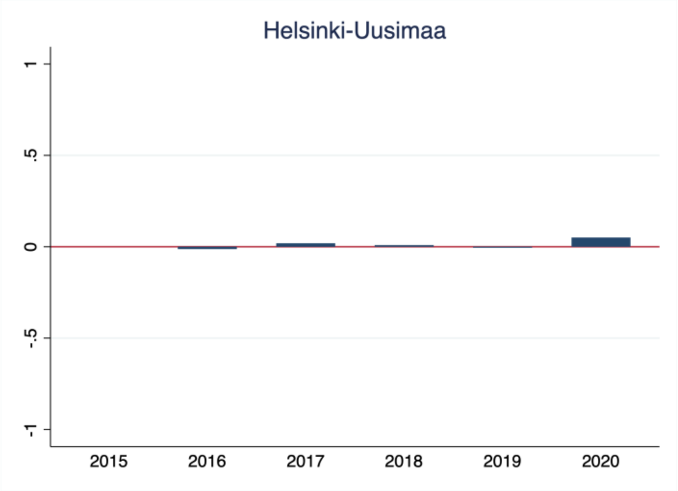

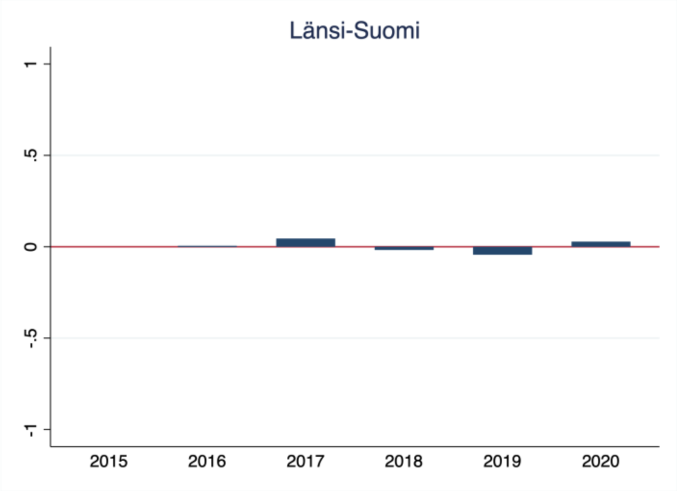

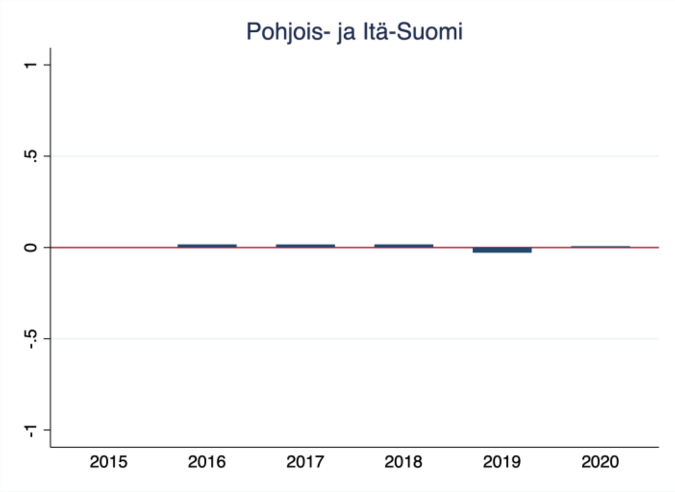
Pohjois- ja Itä-Suomi
1
.5
0
-.5
-1
2015
2016
2017
2018
2019
2020

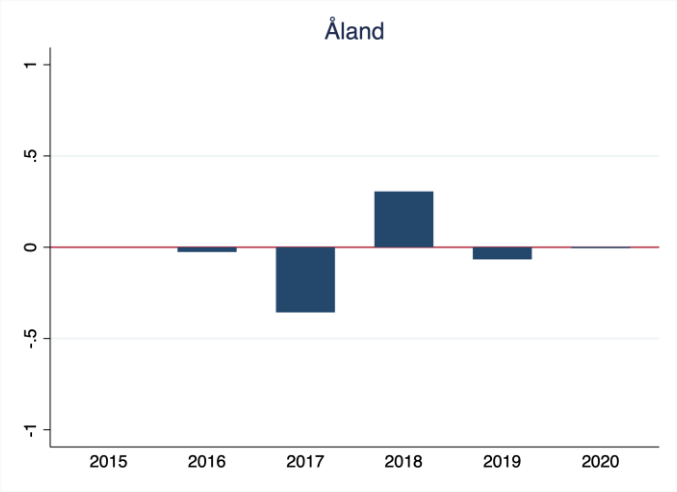
Åland
1
.5
0
-.5
-1
2015
2016
2017
2018
2019
2020

FRANCE

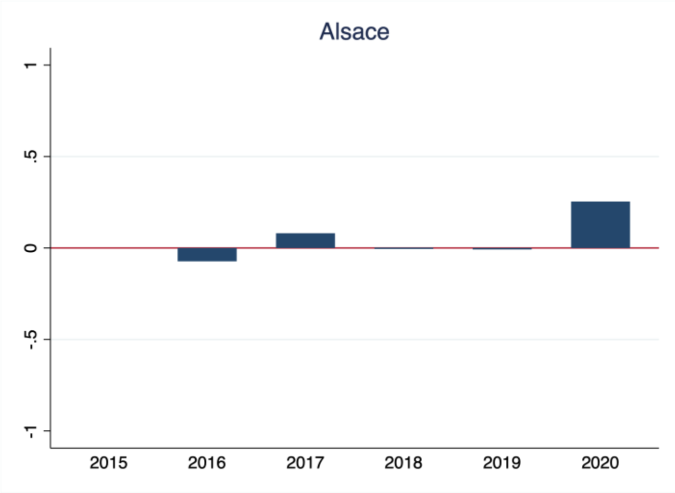
Alsace
1
.5
0
-.5
-1
2015
2016
2017
2018
2019
2020

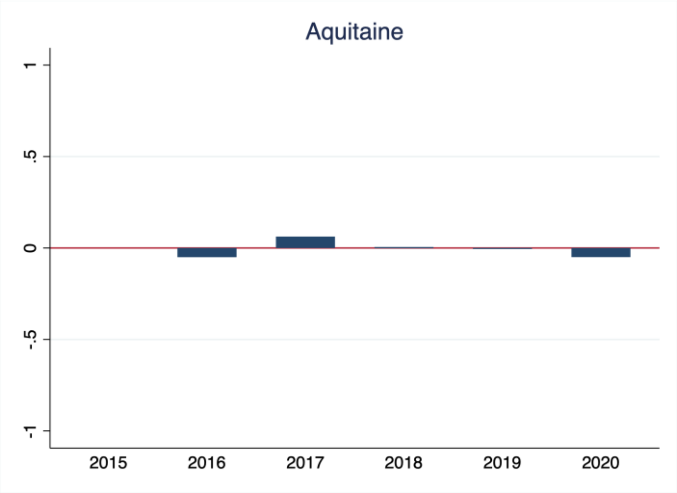
Aquitaine
1
.5
0
-.5
-1
2015
2016
2017
2018
2019
2020

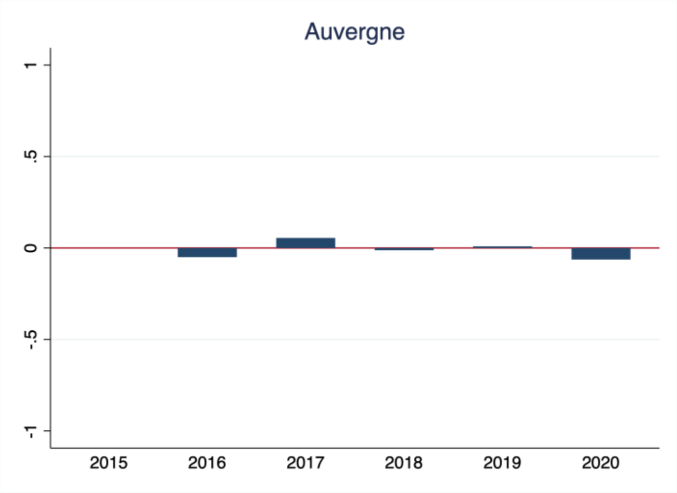
Auvergne
1
.5
0
-.5
-1
2015
2016
2017
2018
2019
2020

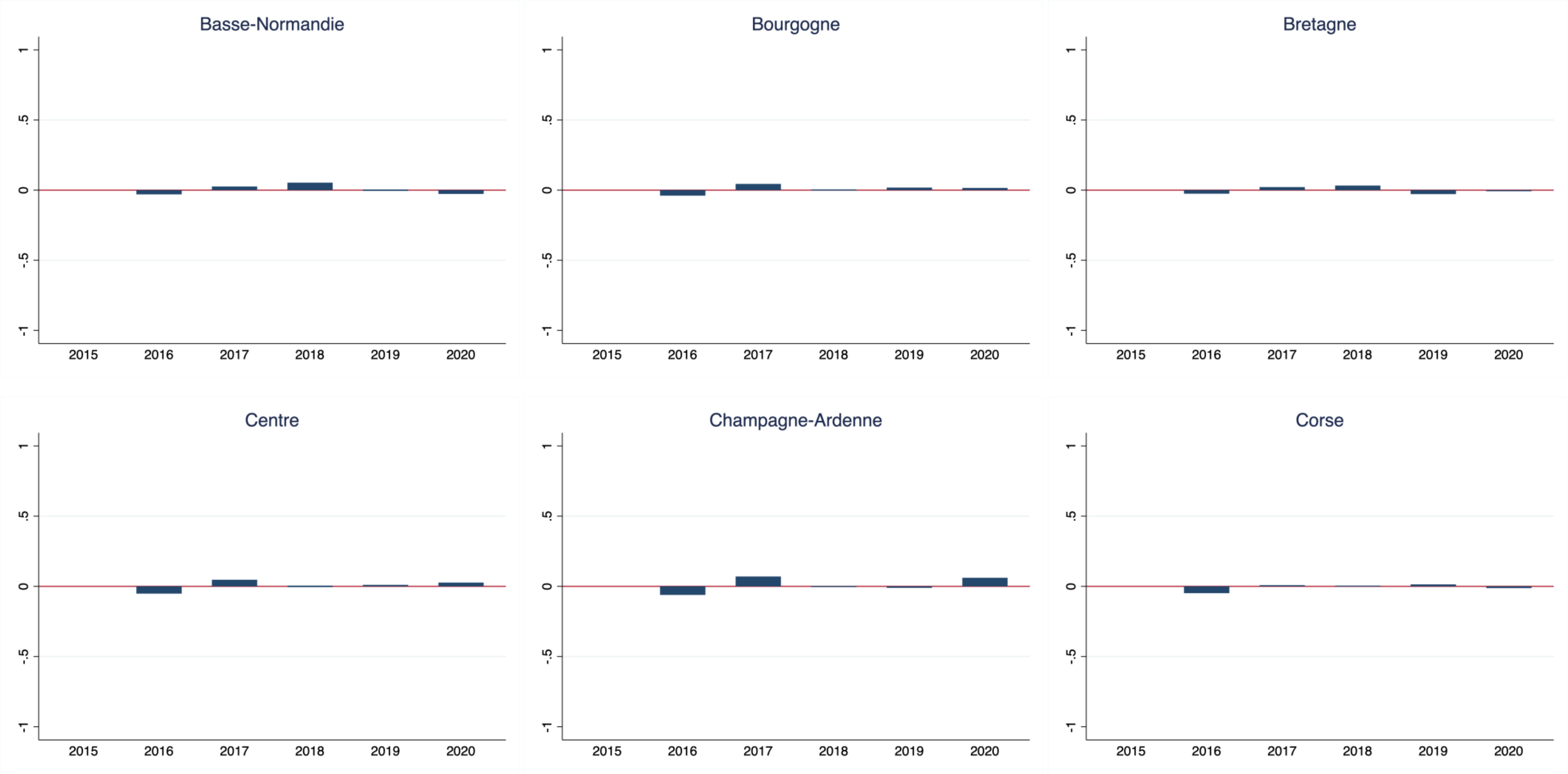
Basse-Normandie
Bourgogne
Bretagne
Centre
Champagne-Ardenne
Corse
-1
-.5
0
.5
1
2015
2016
2017
2018
2019
2020

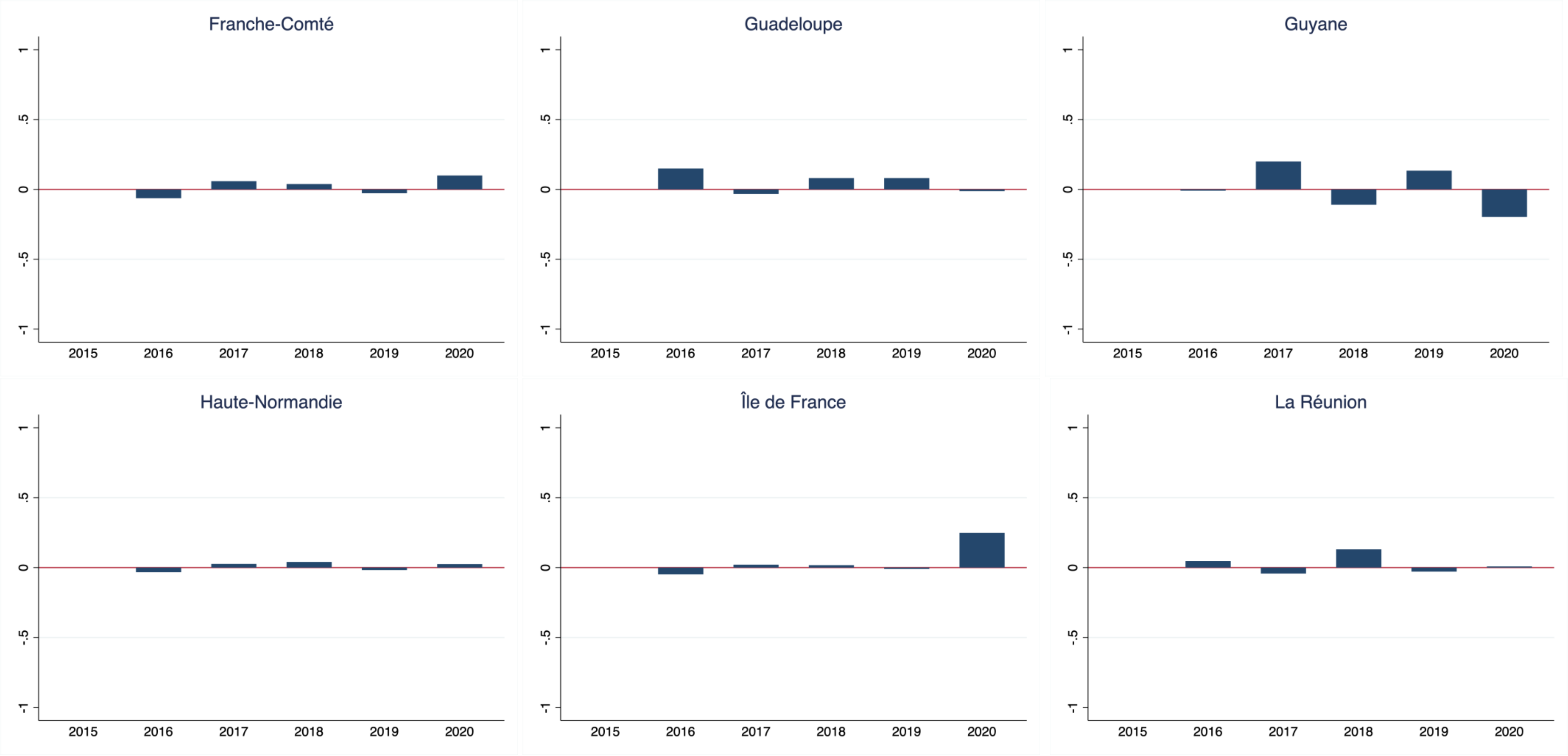
Franche-Comté
Guadeloupe
Guyane
Haute-Normandie
Île de France
La Réunion
1
.5
0
-.5
-1
2015
2016
2017
2018
2019
2020

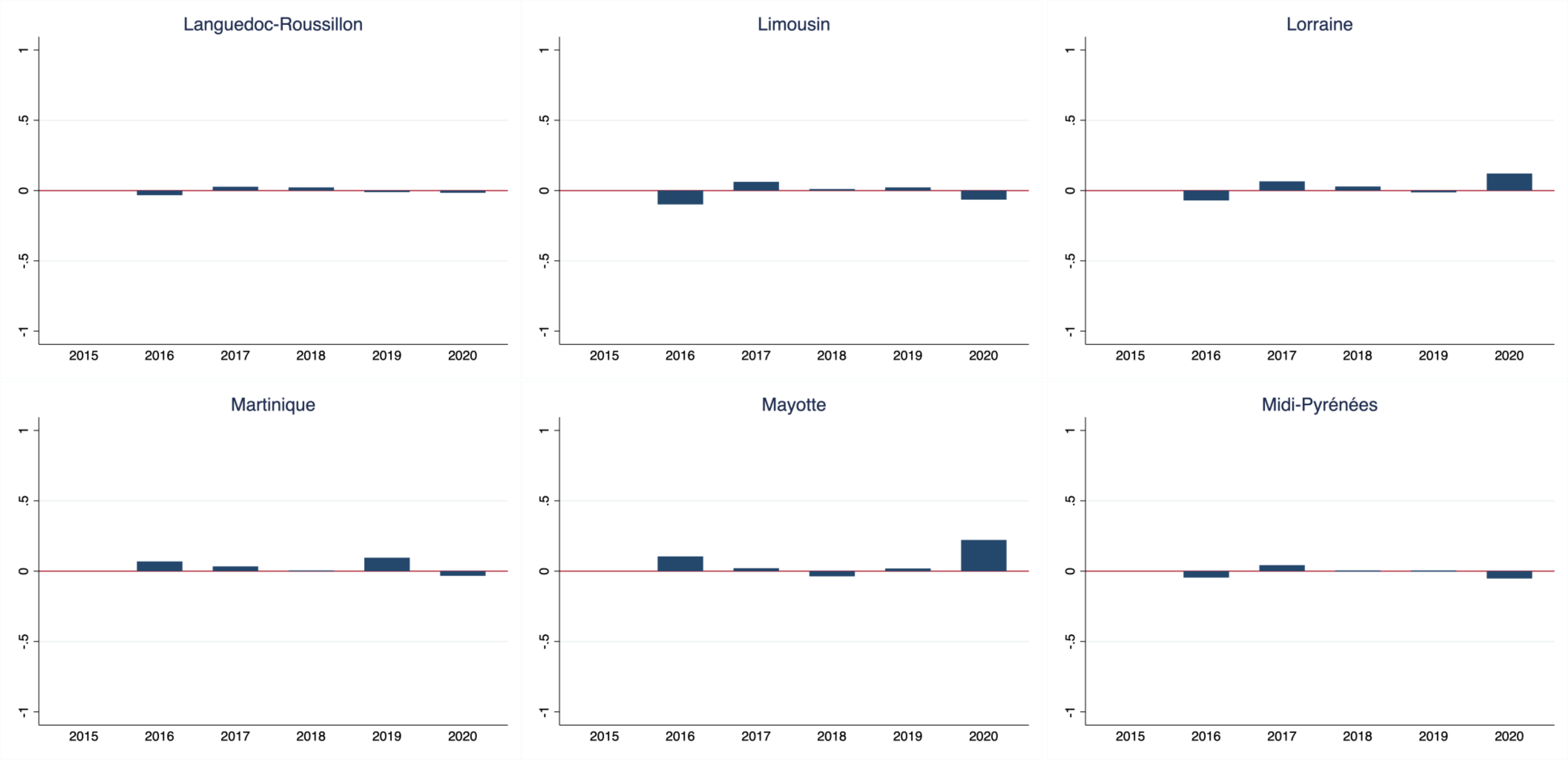
Languedoc-Roussillon
Limousin
Lorraine
Martinique
Mayotte
Midi-Pyrénées
1
.5
0
-.5
-1
2015
2016
2017
2018
2019
2020

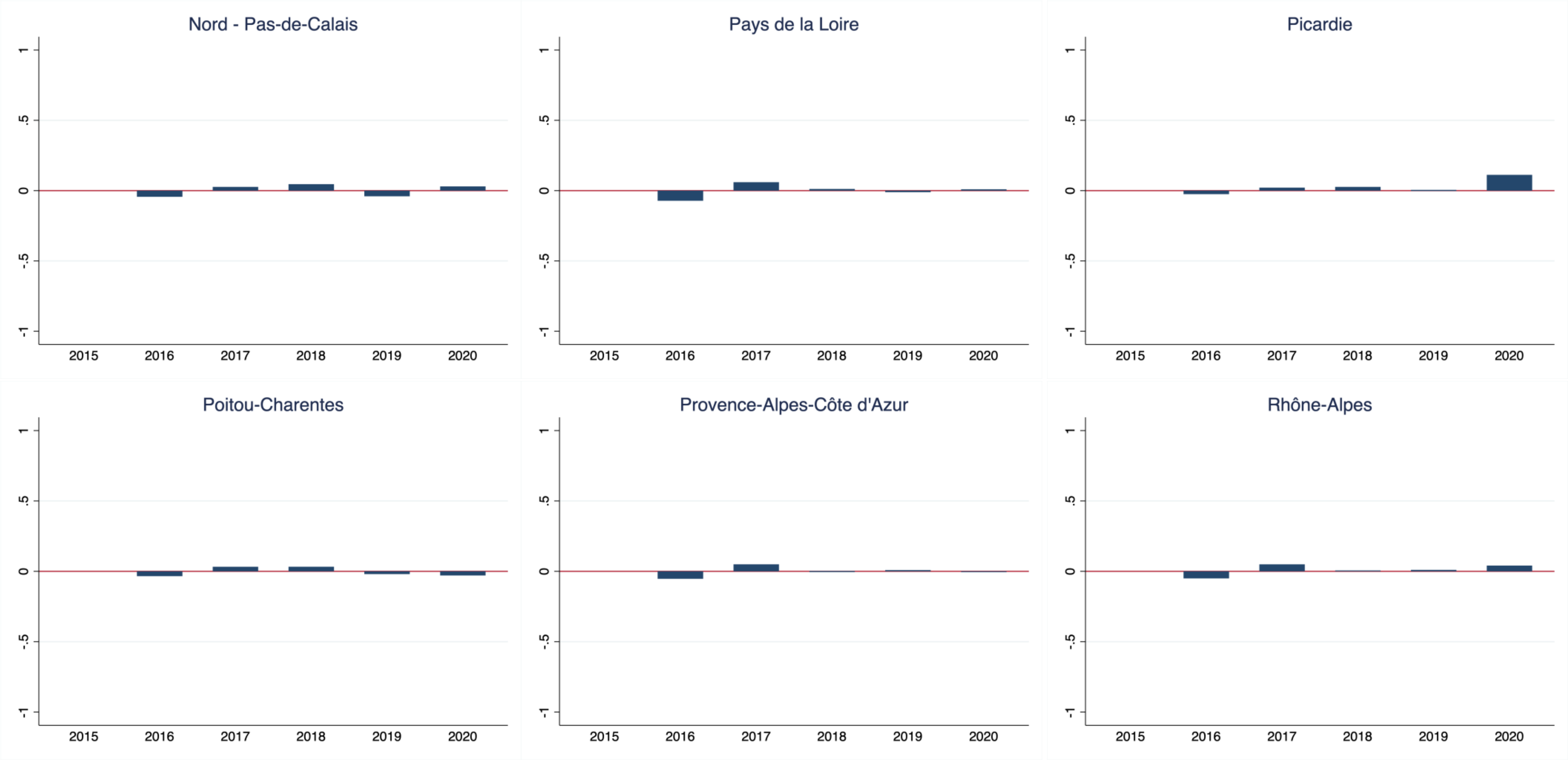
Nord - Pas-de-Calais
Pays de la Loire
Picardie
Poitou-Charentes
Provence-Alpes-Côte d'Azur
Rhône-Alpes
-1
-.5
0
.5
1
2015
2016
2017
2018
2019
2020

HUNGARY

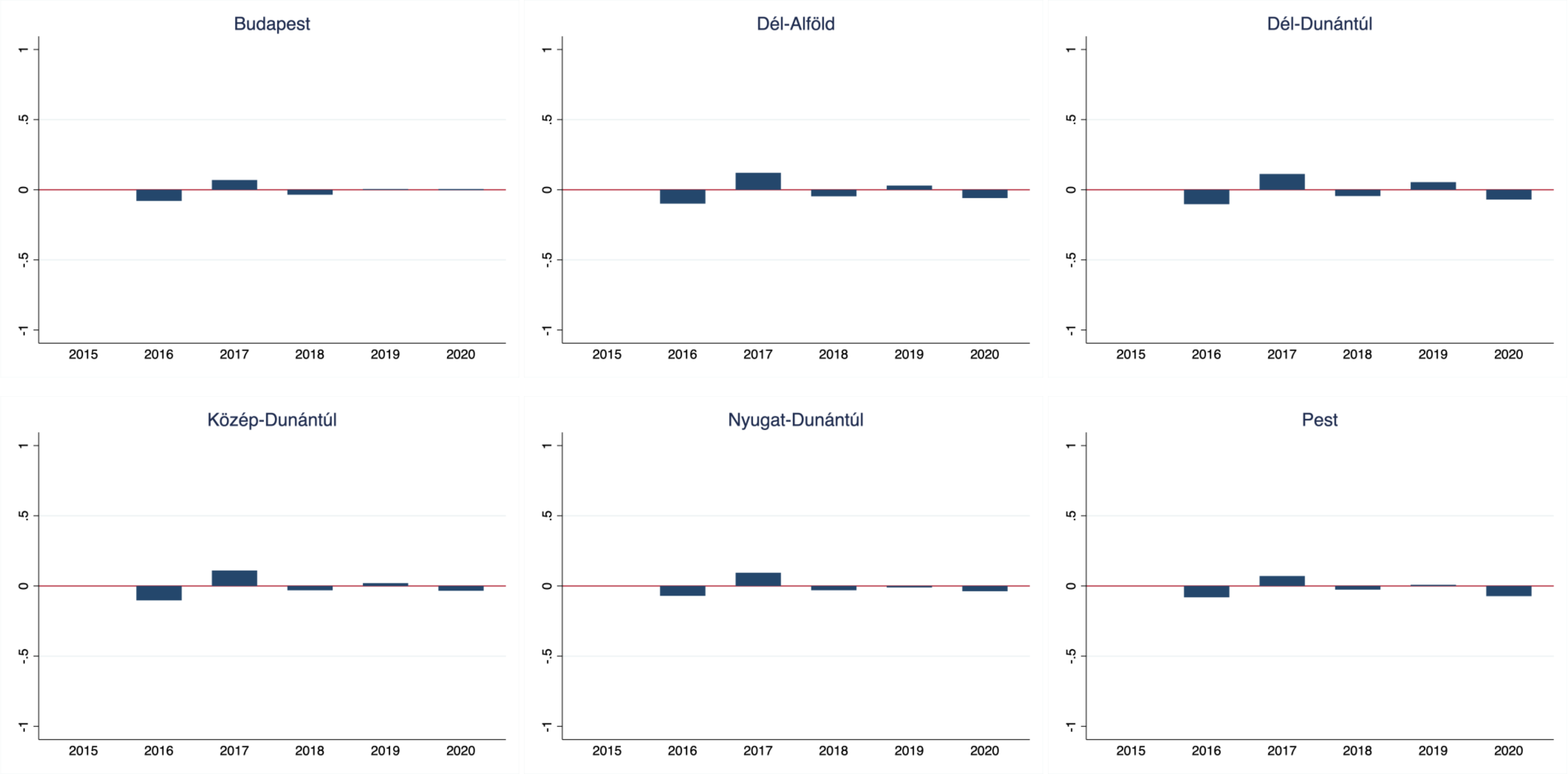

Budapest
Dél-Alföld
Dél-Dunántúl
Közép-Dunántúl
Nyugat-Dunántúl
Pest
1
.5
0
-.5
-1
2015
2016
2017
2018
2019
2020

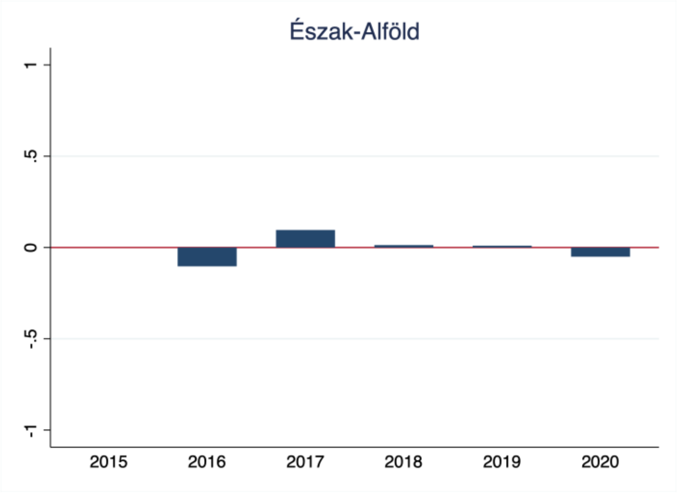
Észak-Alföld
1
.5
0
-.5
-1
2015
2016
2017
2018
2019
2020

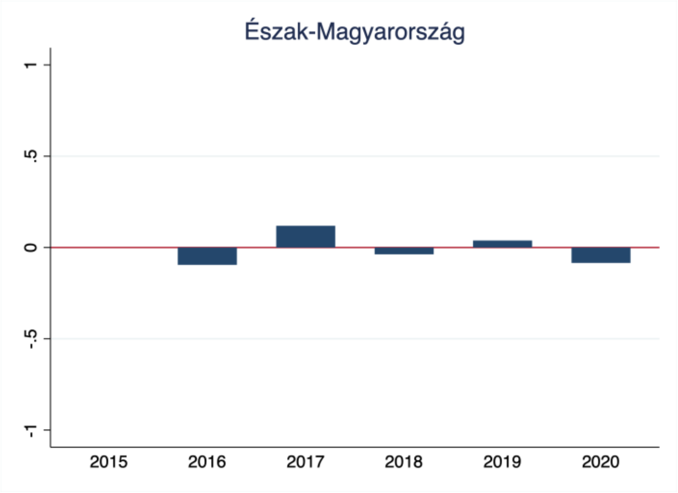
Észak-Magyarország
1
.5
0
-.5
-1
2015
2016
2017
2018
2019
2020

ITALY

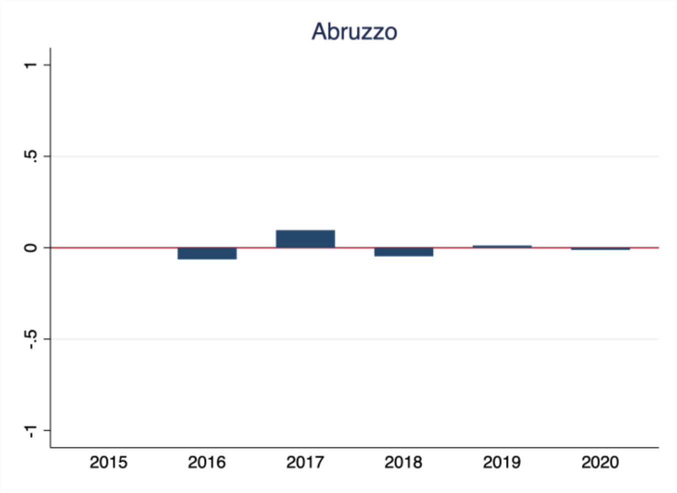
Abruzzo
1
.5
0
-.5
-1
2015
2016
2017
2018
2019
2020

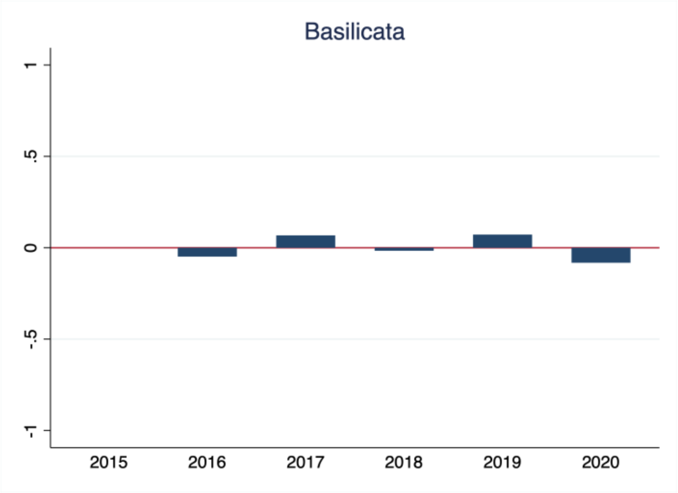
Basilicata
1
.5
0
-.5
-1
2015
2016
2017
2018
2019
2020

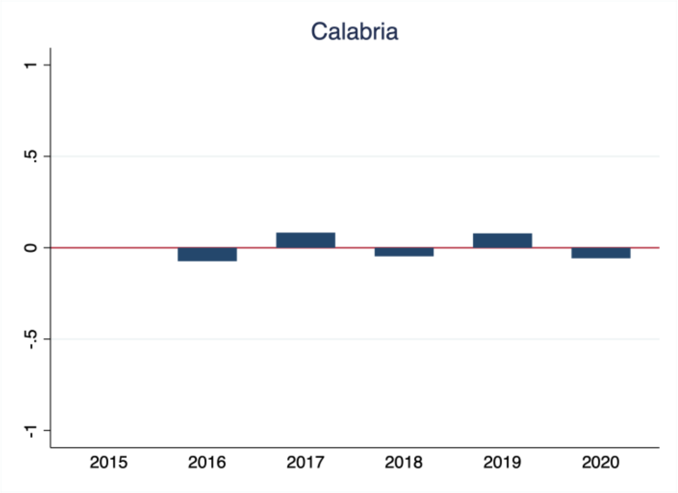
Calabria
1
.5
0
-.5
-1
2015
2016
2017
2018
2019
2020

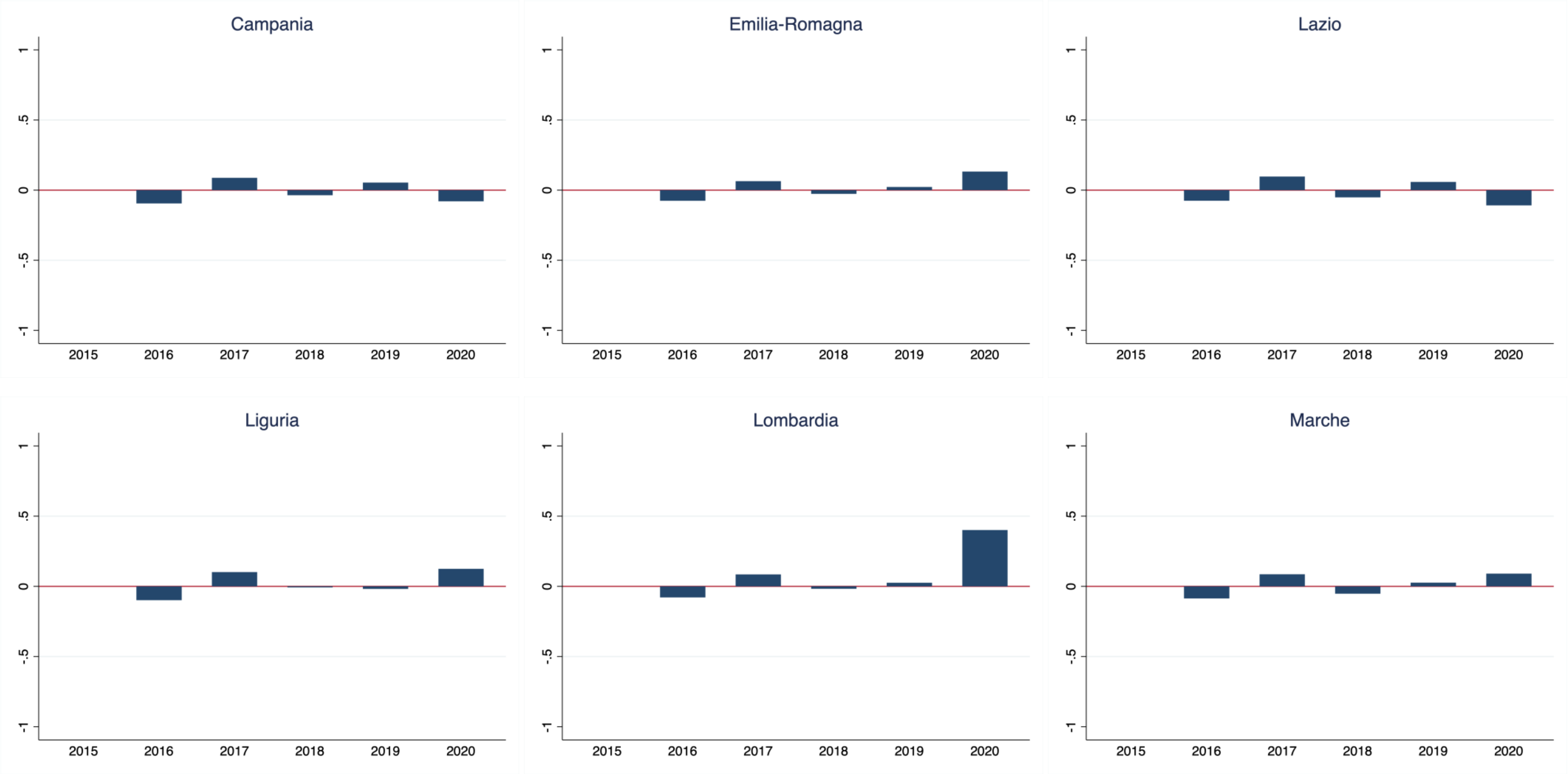
Campania
Emilia-Romagna
Lazio
Liguria
Lombardia
Marche
1
.5
0
-.5
-1
2015
2016
2017
2018
2019
2020

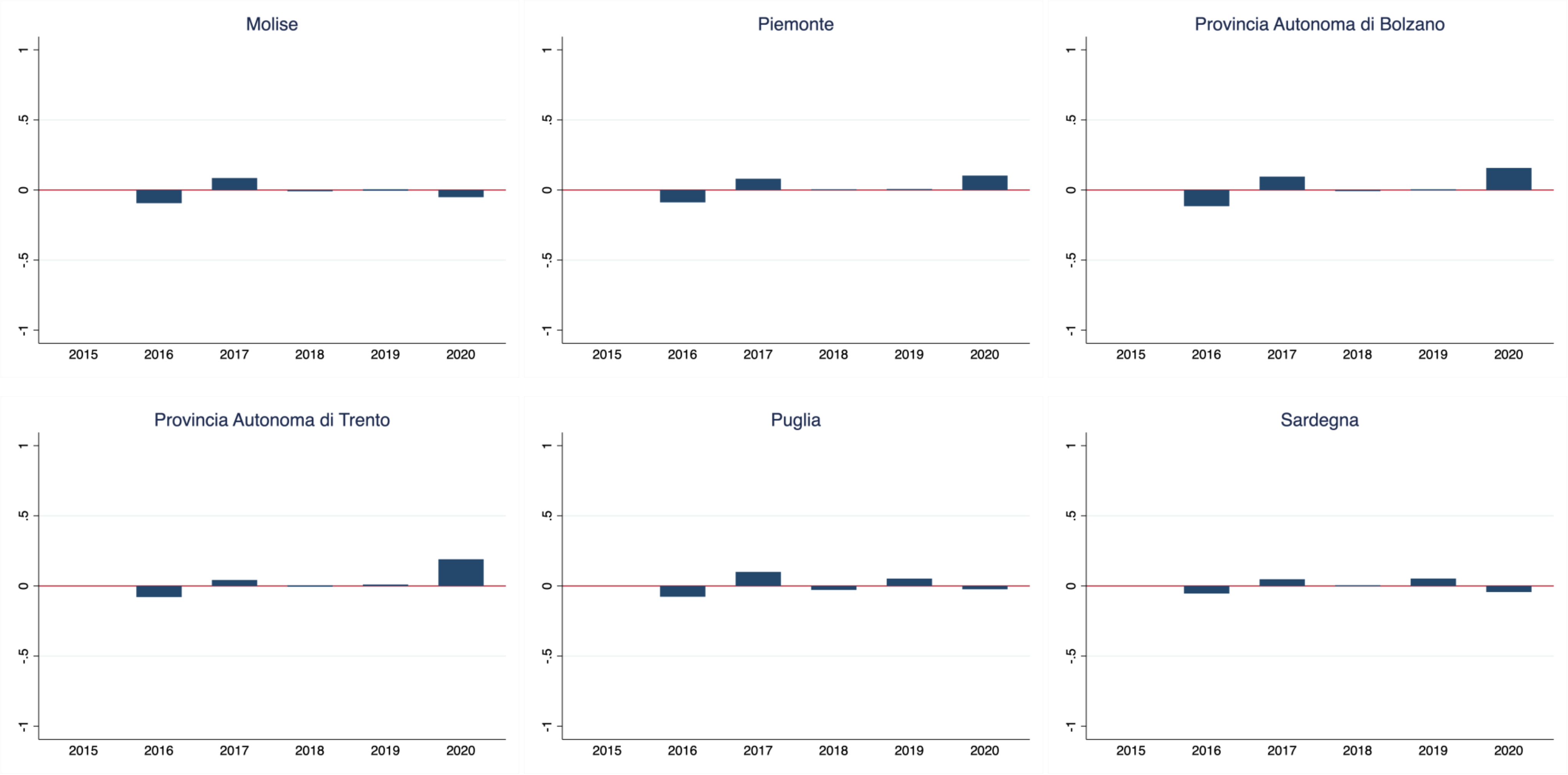
Molise
Piemonte
Provincia Autonoma di Bolzano
Provincia Autonoma di Trento
Puglia
Sardegna
1
.5
0
-.5
-1
2015
2016
2017
2018
2019
2020

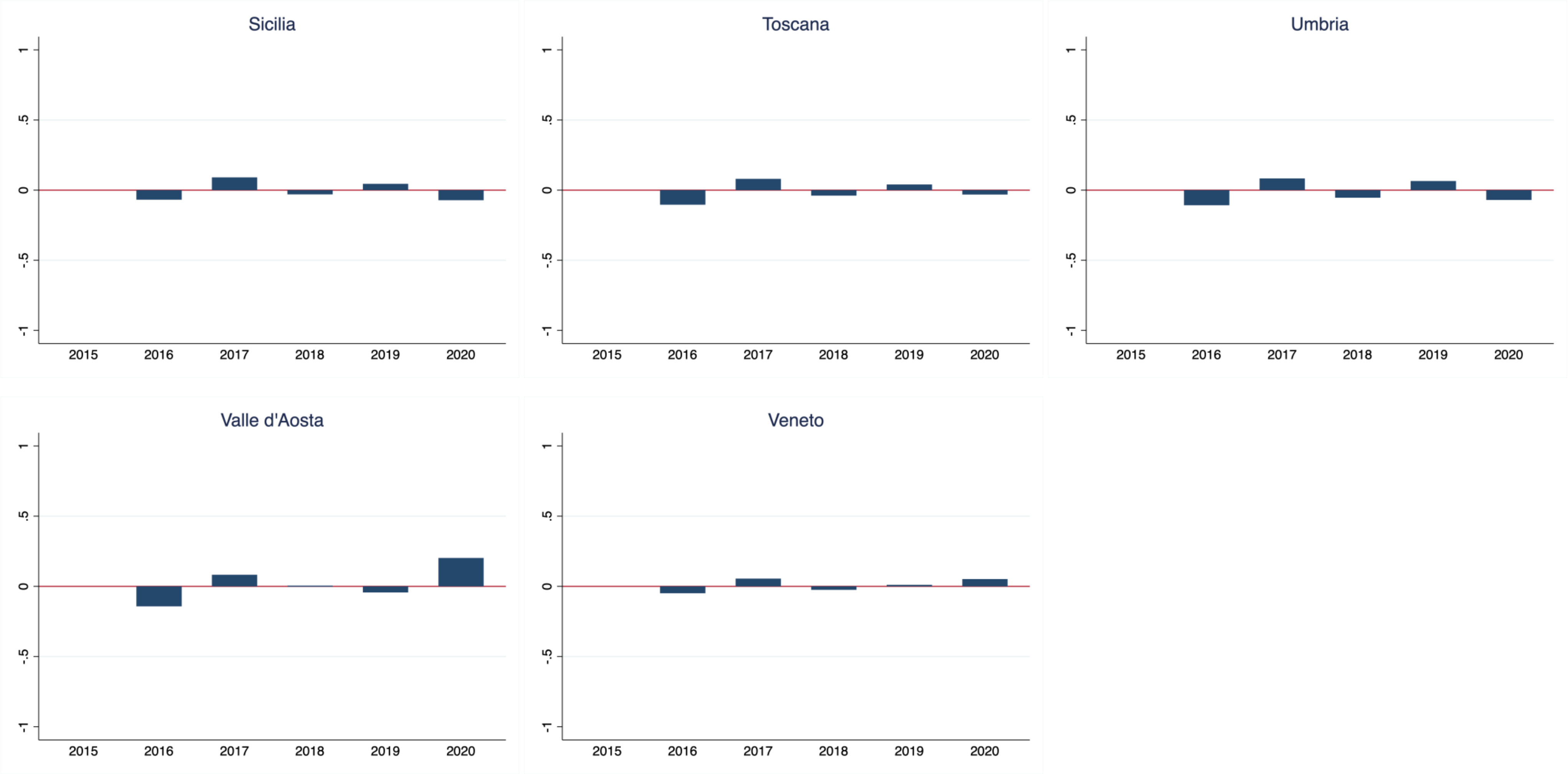
Sicilia
Toscana
Umbria
Valle d'Aosta
Veneto
1
.5
0
-.5
-1
2015
2016
2017
2018
2019
2020

LITHUANIA

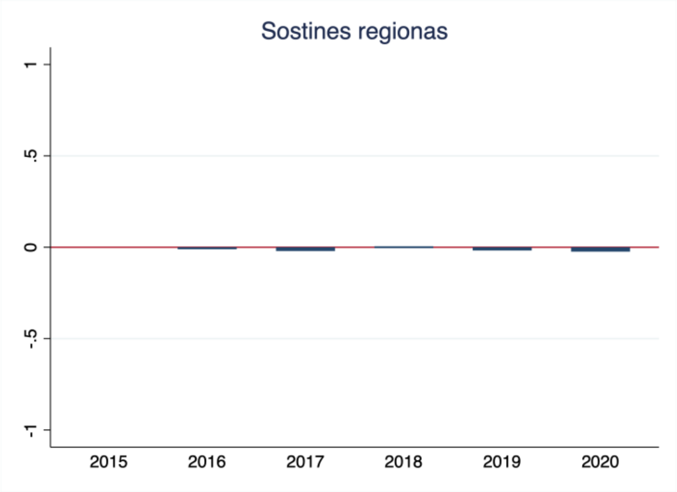

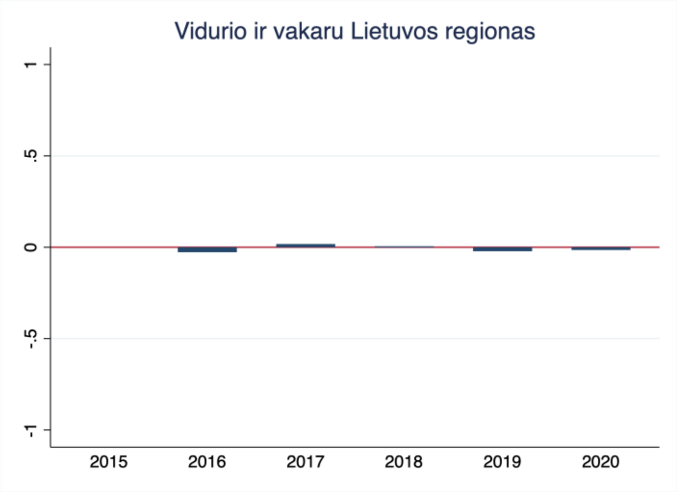

LUXEMBOURG

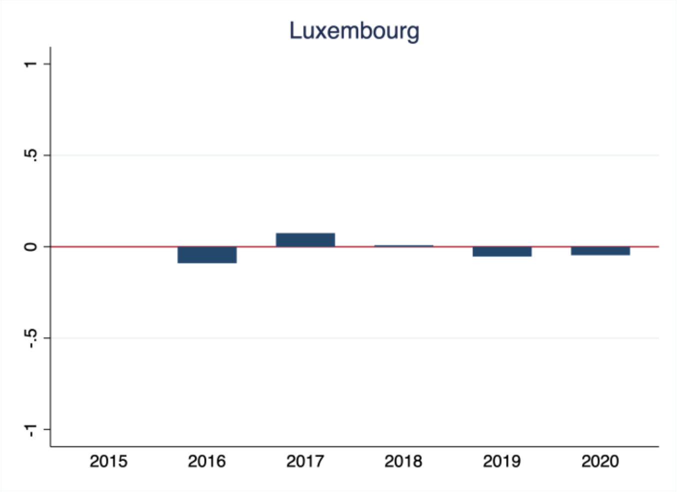

LATVIA

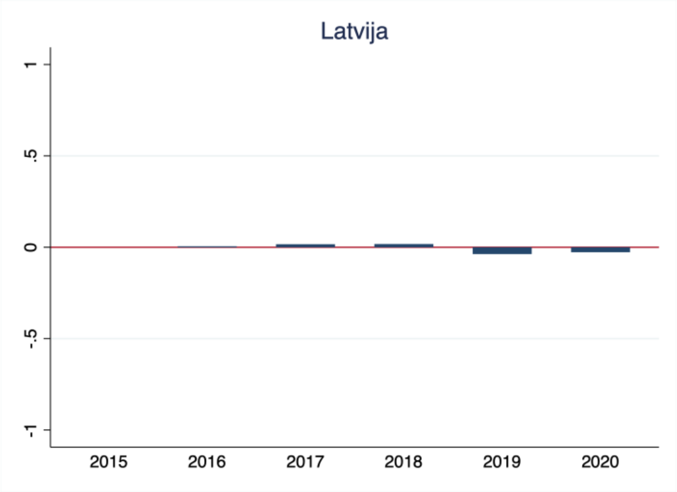

NETHERLANDS

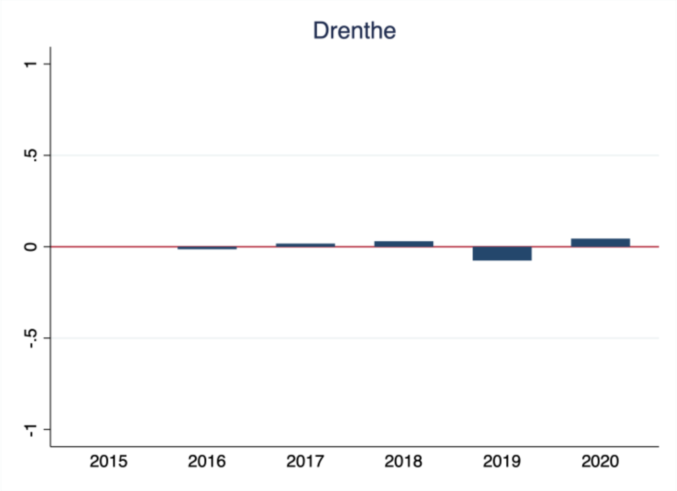

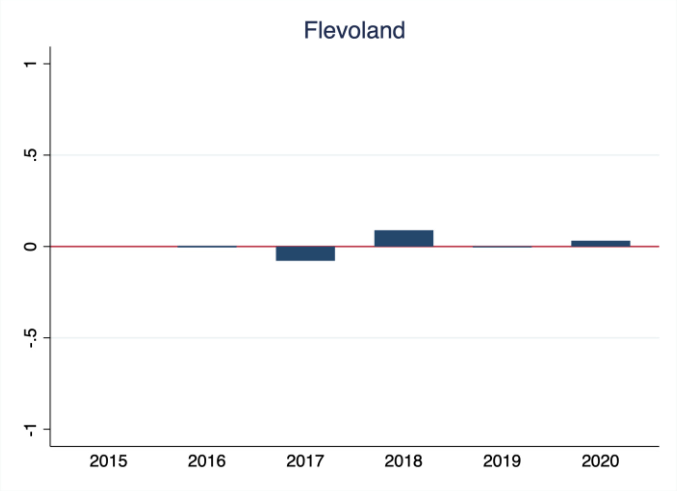

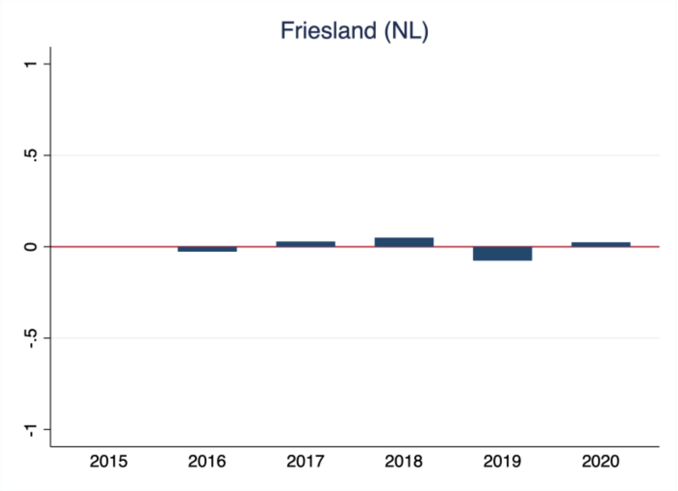

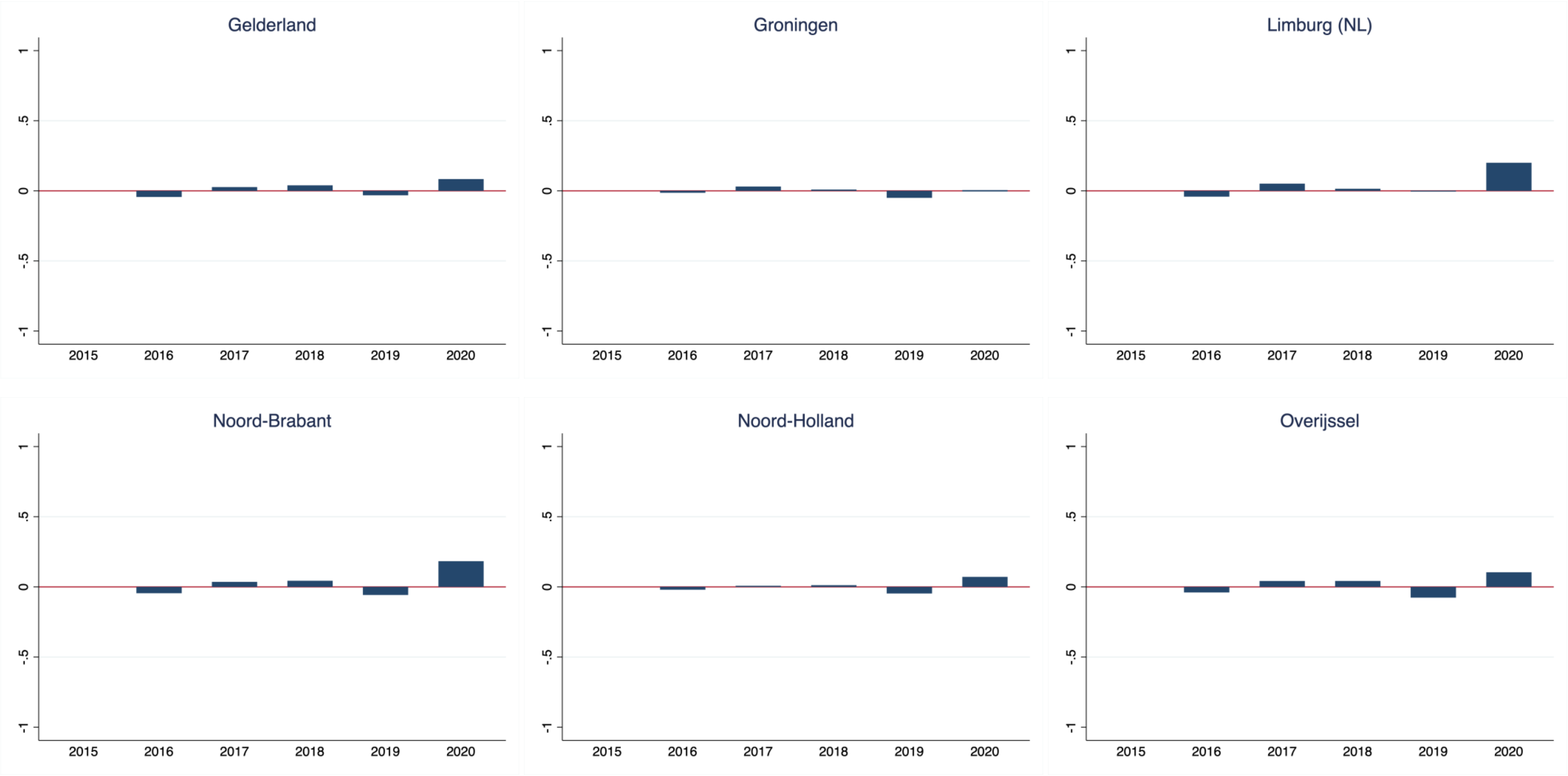
Gelderland
Groningen
Limburg (NL)
Noord-Brabant
Noord-Holland
Overijssel
1
.5
0
-.5
-1
2015
2016
2017
2018
2019
2020

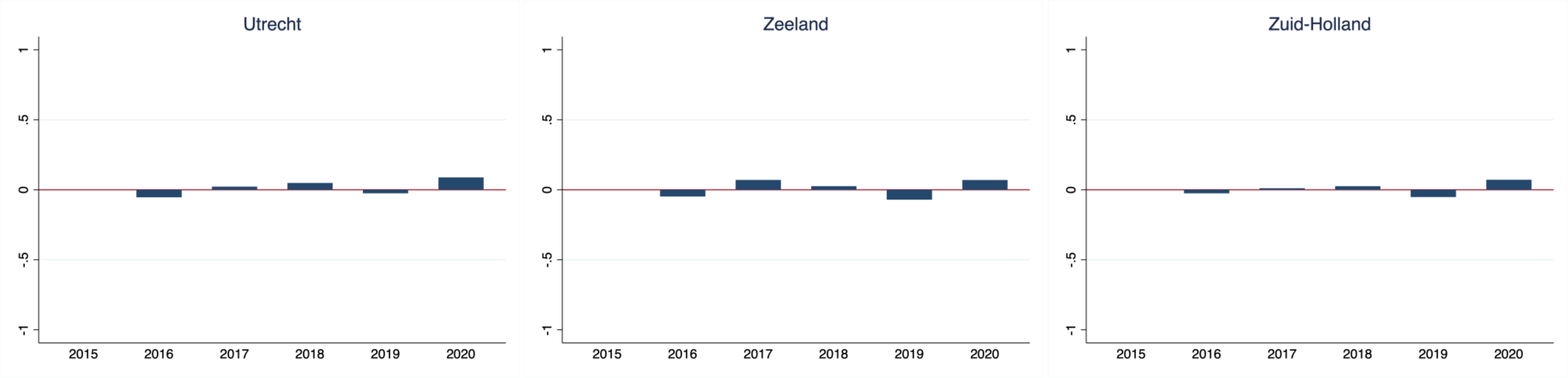
Utrecht
Zeeland
Zuid-Holland
1
.5
0
-.5
-1
2015
2016
2017
2018
2019
2020

NORWAY

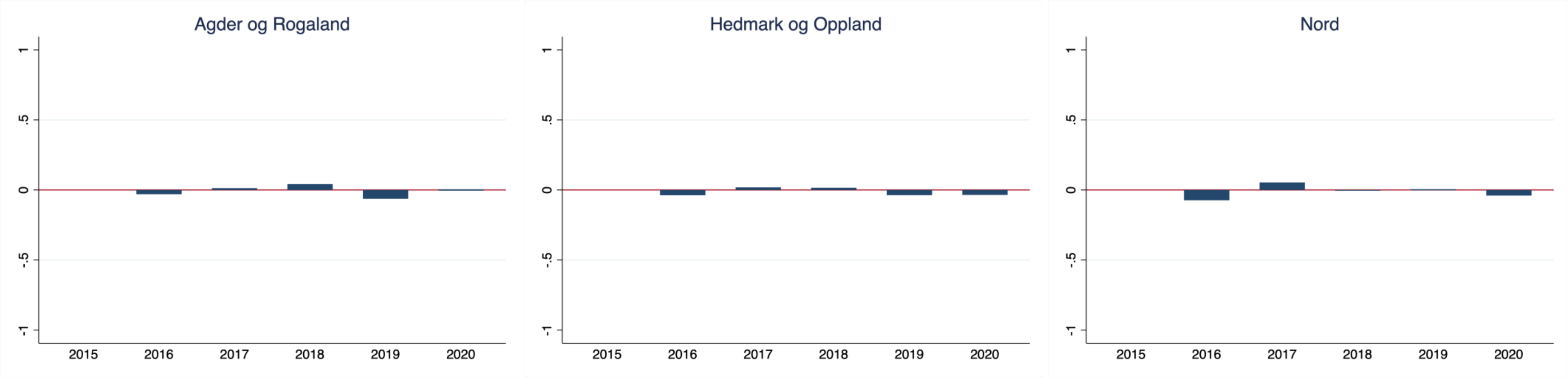
Agder og Rogaland
Hedmark og Oppland
Nord
1
.5
0
-.5
-1
2015
2016
2017
2018
2019
2020

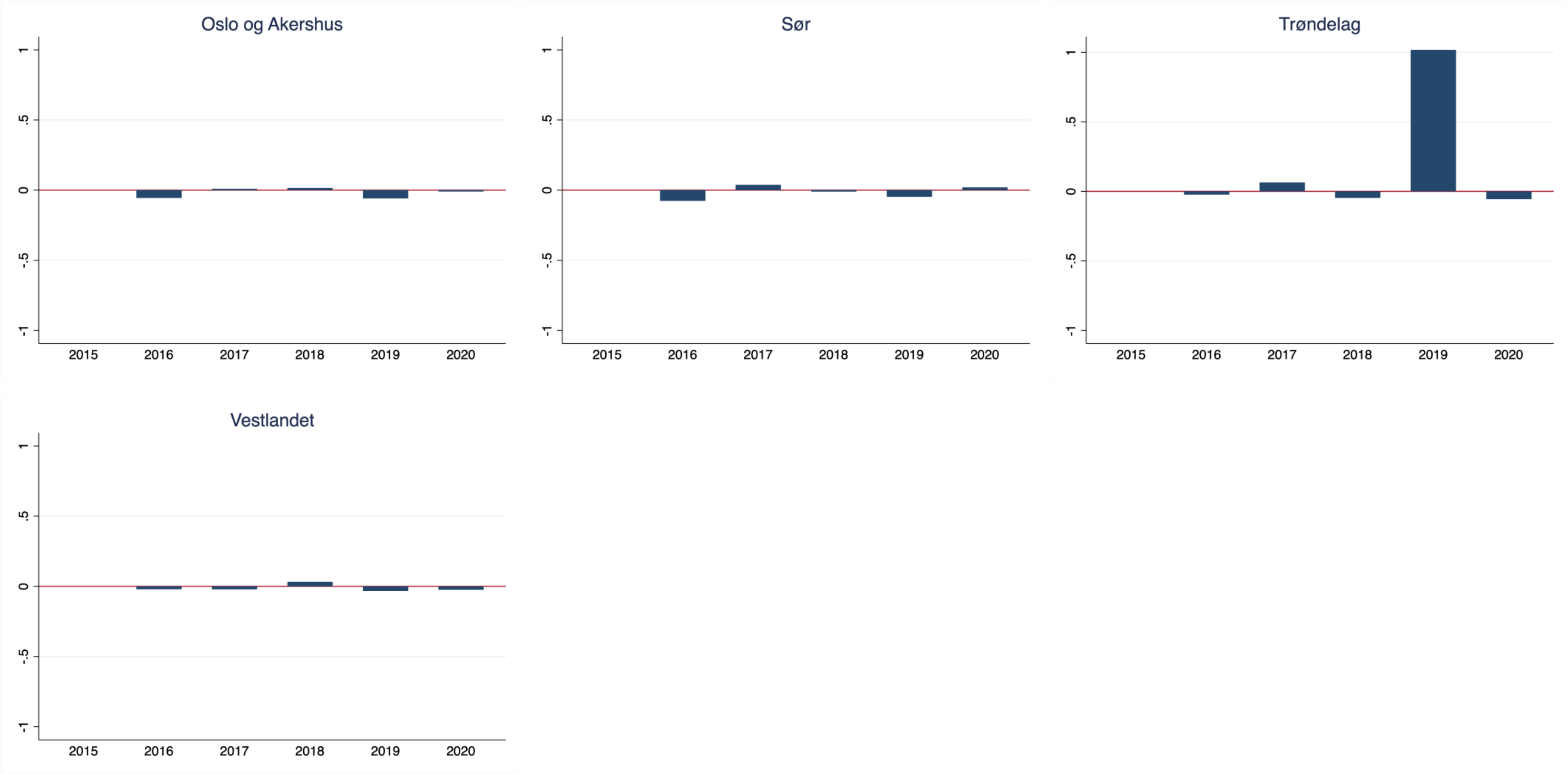
Oslo og Akershus
Sør
Trøndelag
Vestlandet
1
.5
0
-.5
-1
2015
2016
2017
2018
2019
2020

POLAND

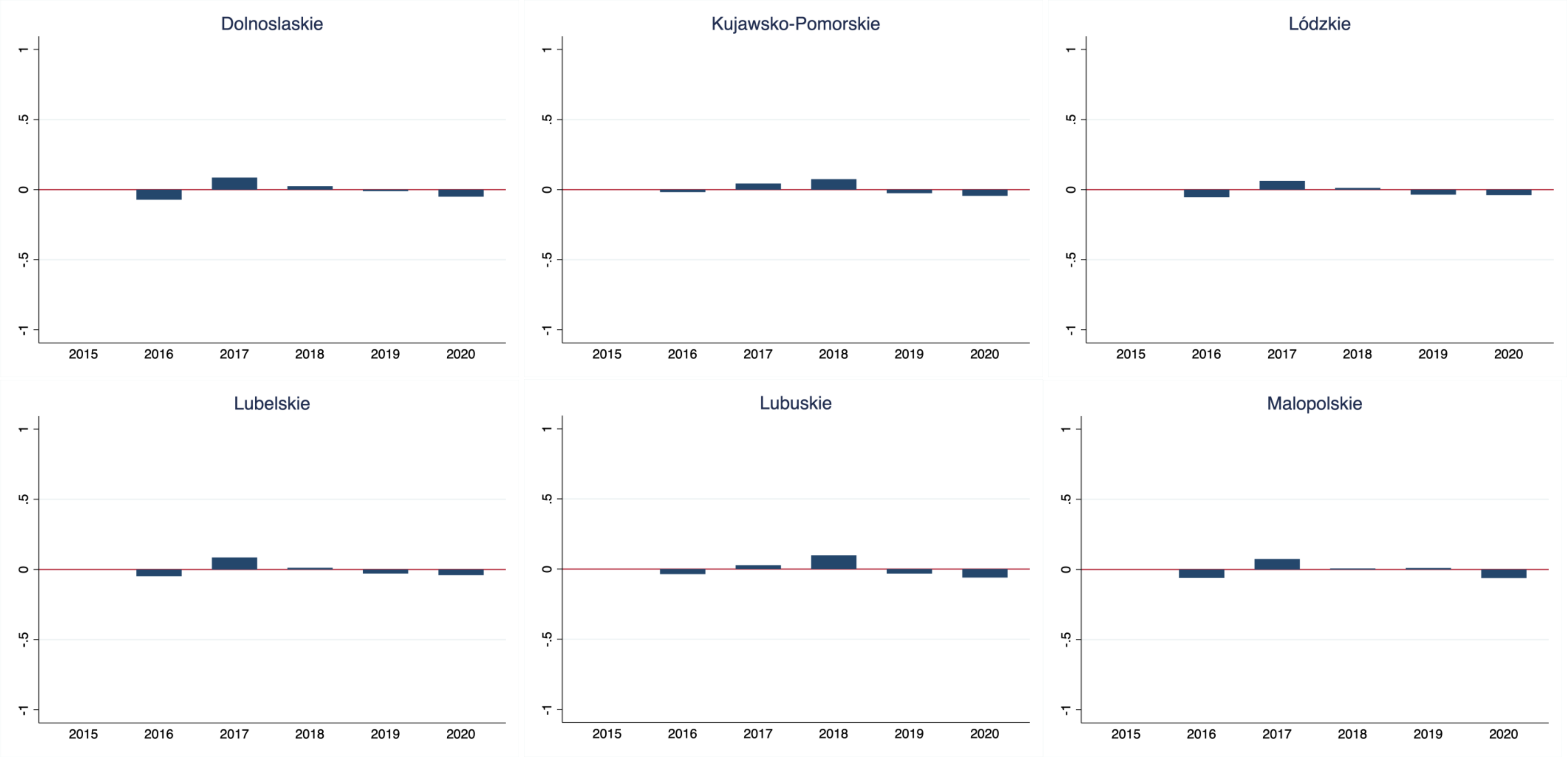
Dolnoslaskie
Kujawsko-Pomorskie
Lódzkie
Lubelskie
Lubuskie
Malopolskie
1
.5
0
-.5
-1
2015
2016
2017
2018
2019
2020

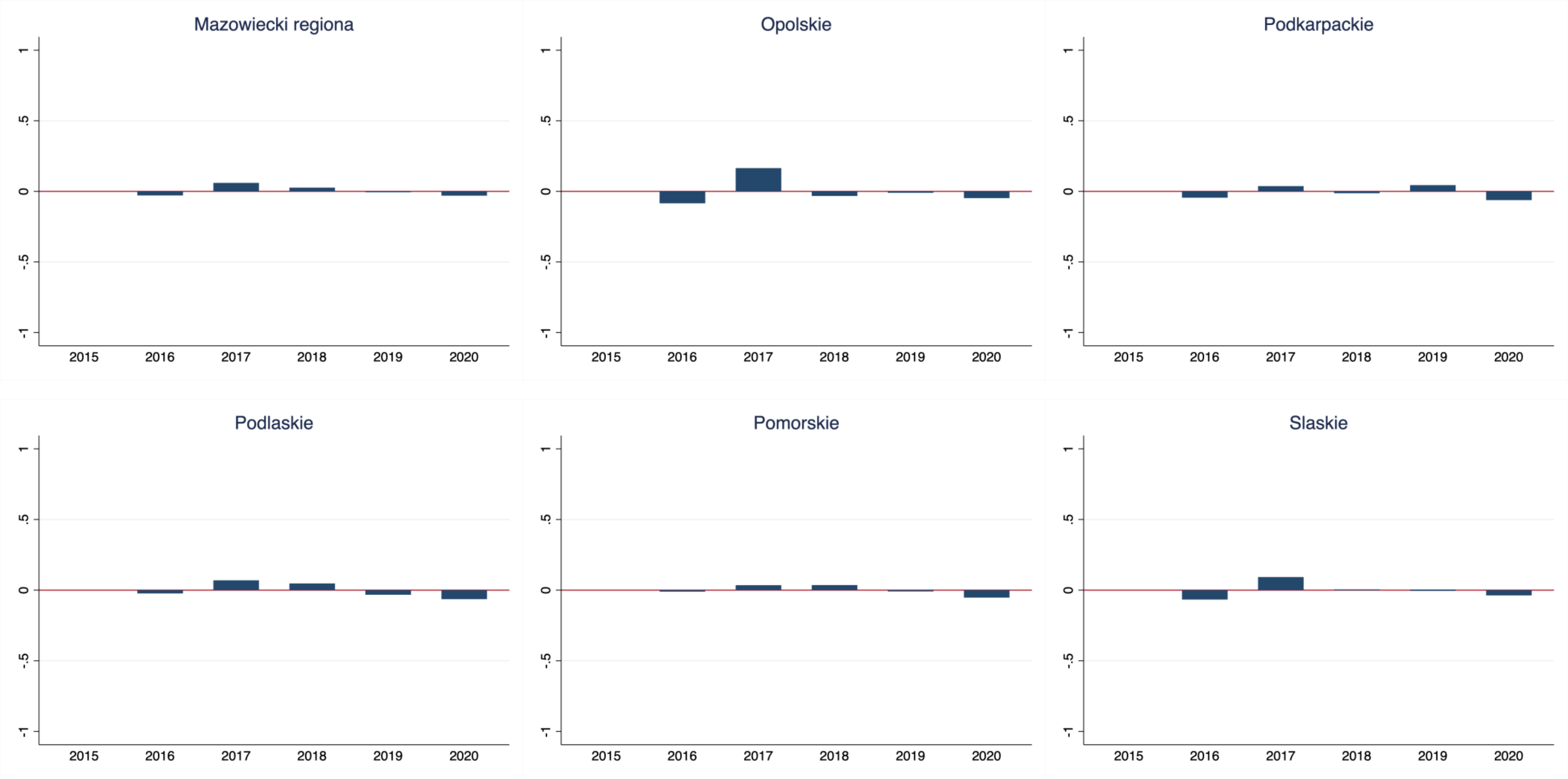
Mazowiecki regiona
Opolskie
Podkarpackie
Podlaskie
Pomorskie
Slaskie
1
.5
0
-.5
-1
2015
2016
2017
2018
2019
2020

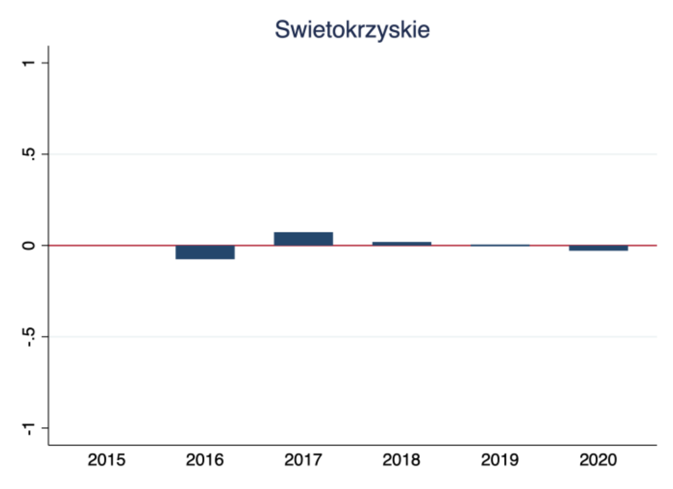
Swietokrzyskie
1
.5
0
-.5
-1
2015
2016
2017
2018
2019
2020

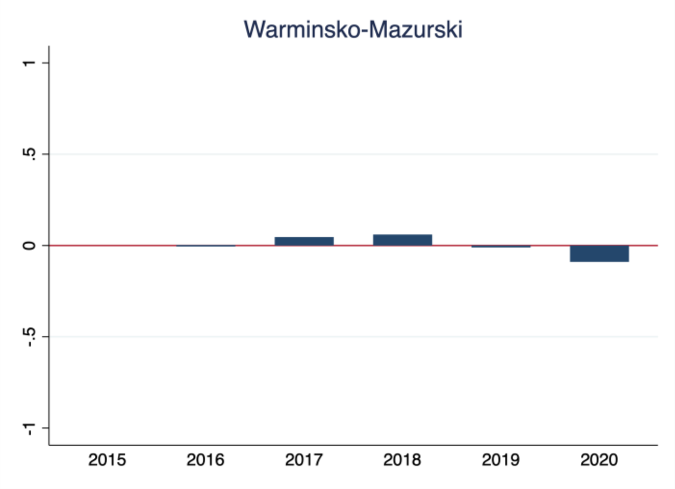
Warminsko-Mazurski
1
.5
0
-.5
-1
2015
2016
2017
2018
2019
2020

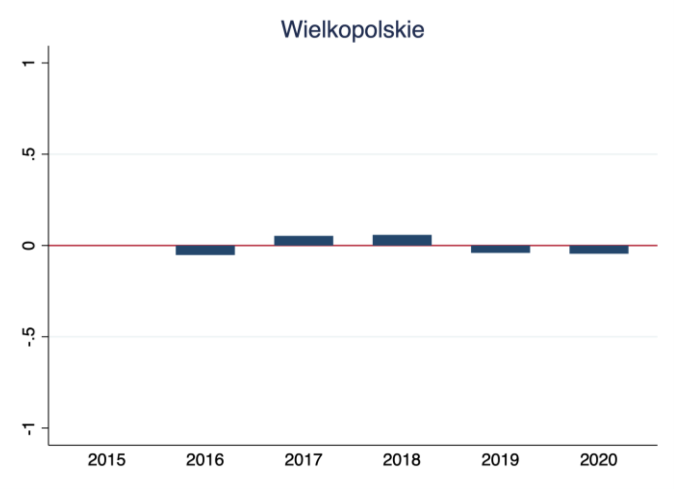
Wielkopolskie
1
.5
0
-.5
-1
2015
2016
2017
2018
2019
2020

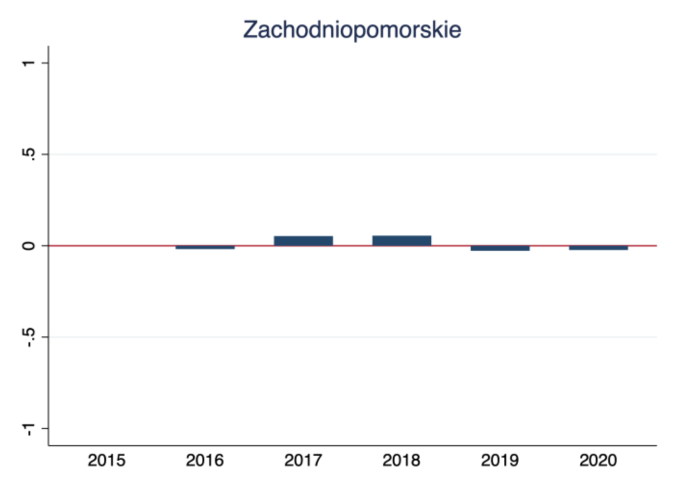
Zachodniopomorskie
1
.5
0
-.5
-1
2015
2016
2017
2018
2019
2020

PORTUGAL

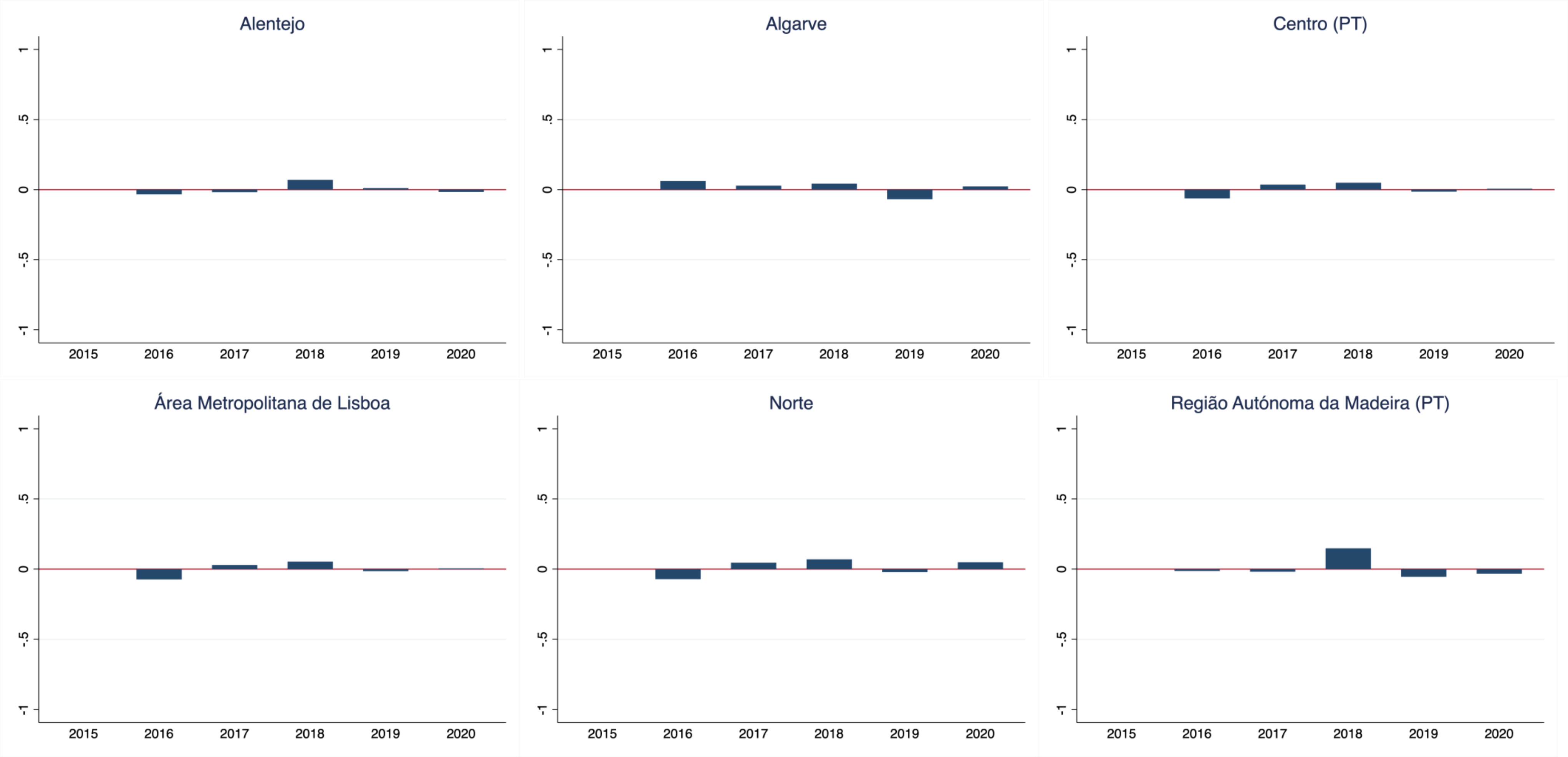

Alentejo
Algarve
Centro (PT)
Área Metropolitana de Lisboa
Norte
Região Autónoma da Madeira (PT)
1
.5
0
-.5
-1
2015
2016
2017
2018
2019
2020

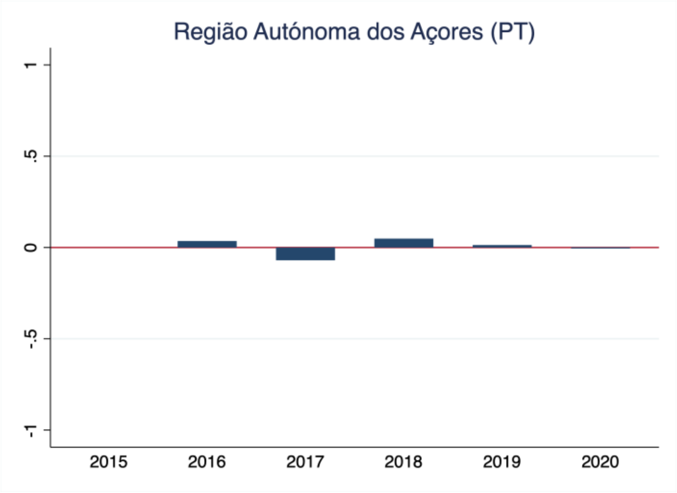

SLOVAK REPUBLIC

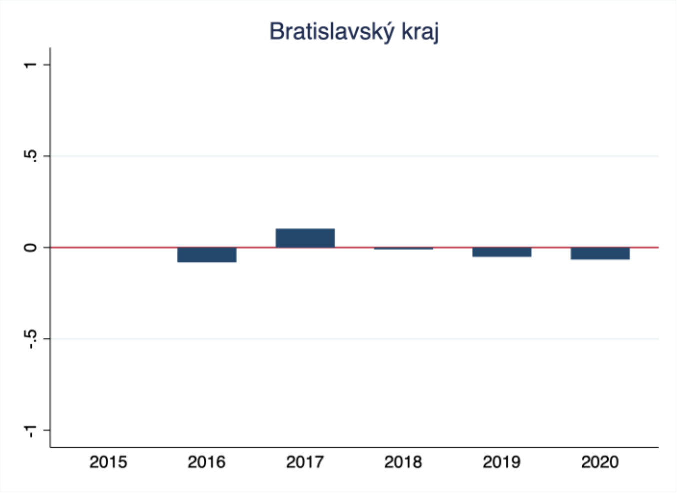

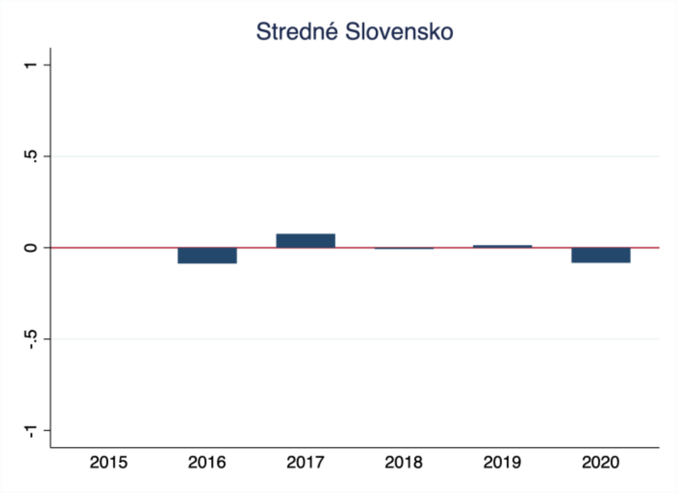

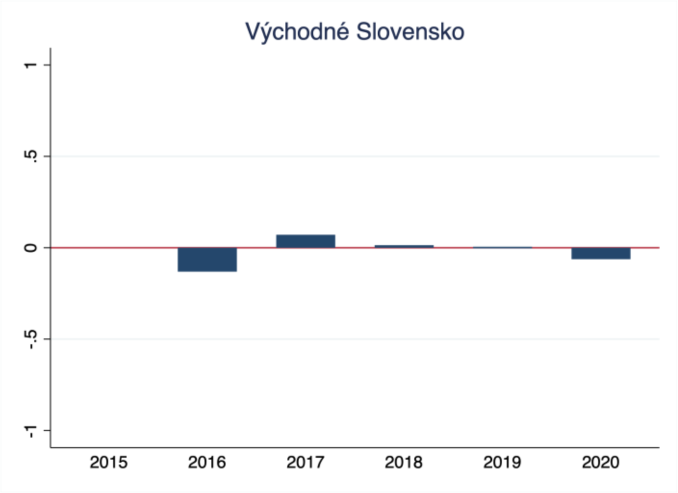

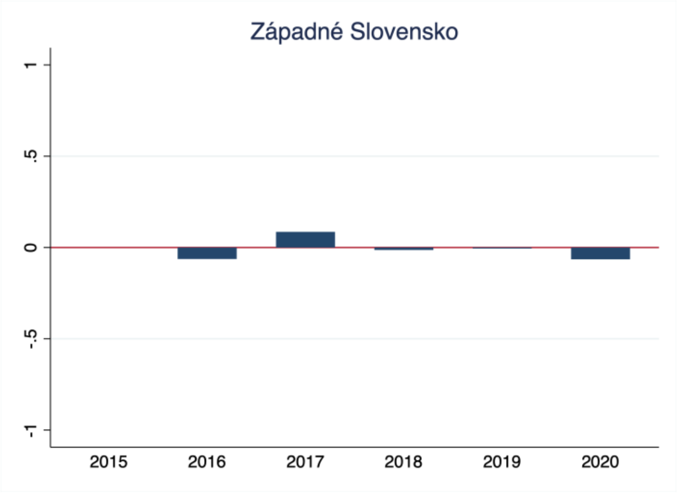
Západné Slovensko
1
.5
0
-.5
-1
2015
2016
2017
2018
2019
2020

SPAIN

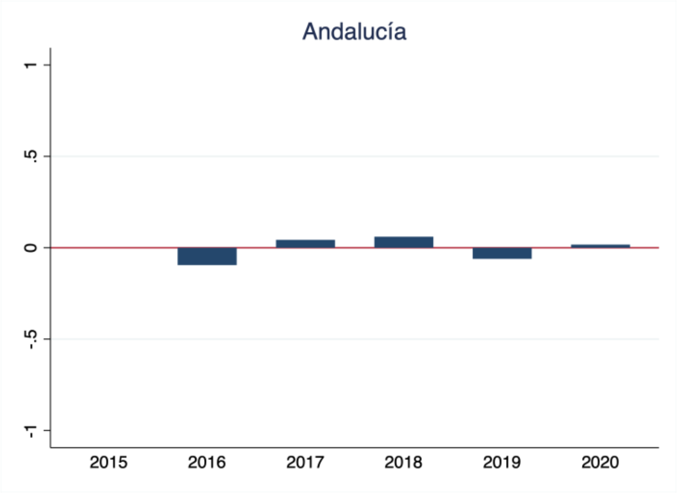
Andalucía
1
.5
0
-.5
-1
2015
2016
2017
2018
2019
2020

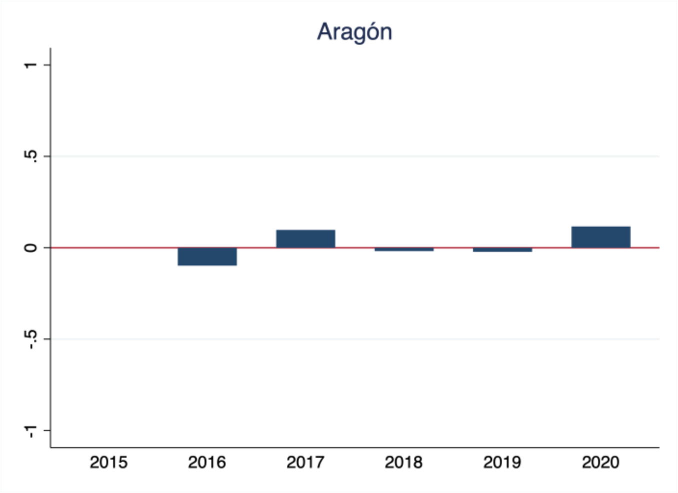
Aragón
1
.5
0
-.5
-1
2015
2016
2017
2018
2019
2020

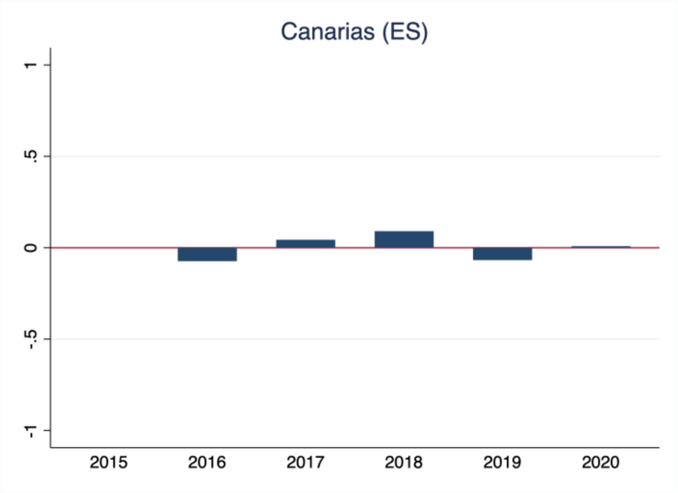
Canarias (ES)
1
.5
0
-.5
-1
2015
2016
2017
2018
2019
2020

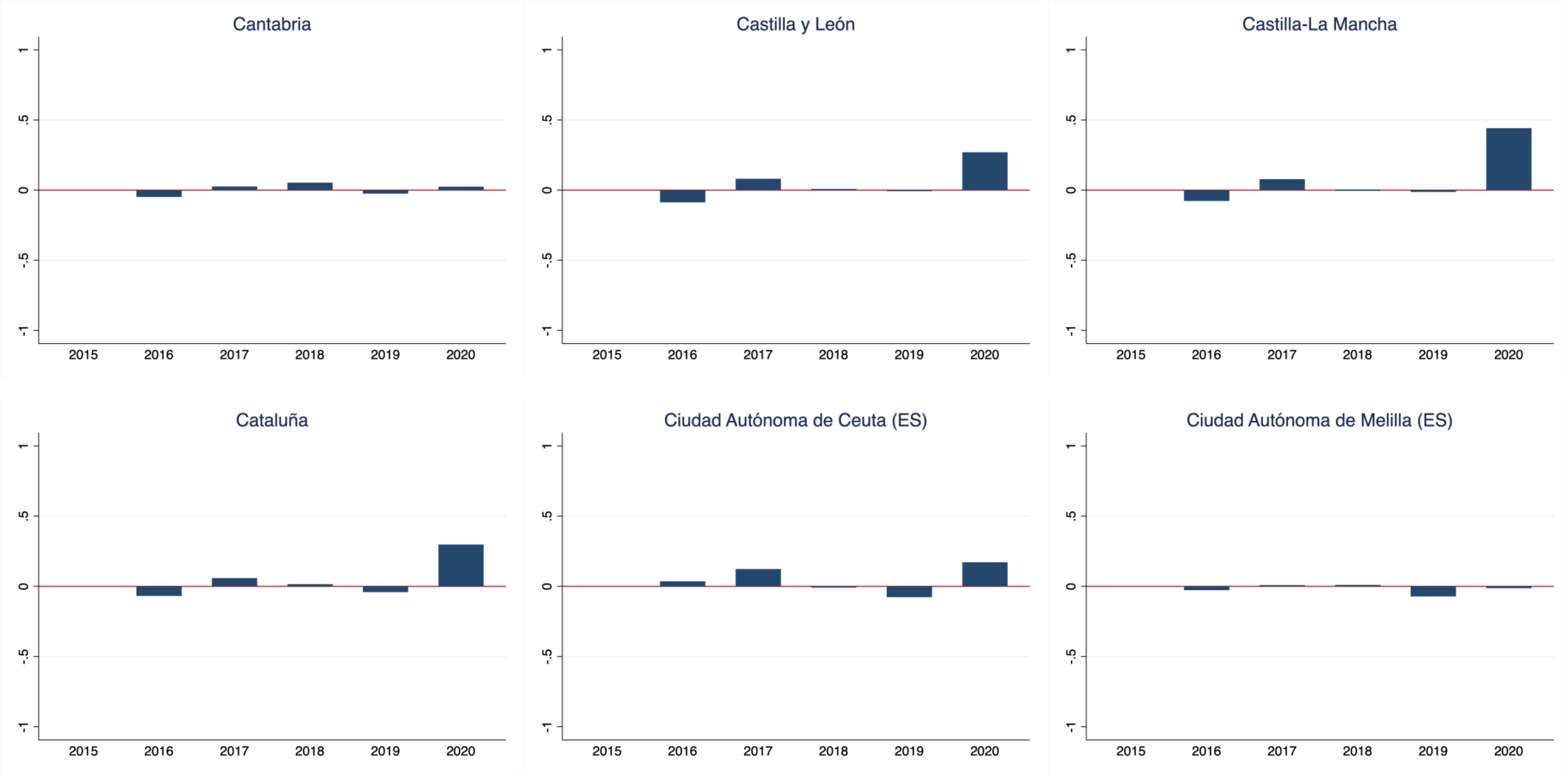

Cantabria
Castilla y León
Castilla-La Mancha
Cataluña
Ciudad Autónoma de Ceuta (ES)
Ciudad Autónoma de Melilla (ES)
1
.5
0
-.5
-1
2015
2016
2017
2018
2019
2020

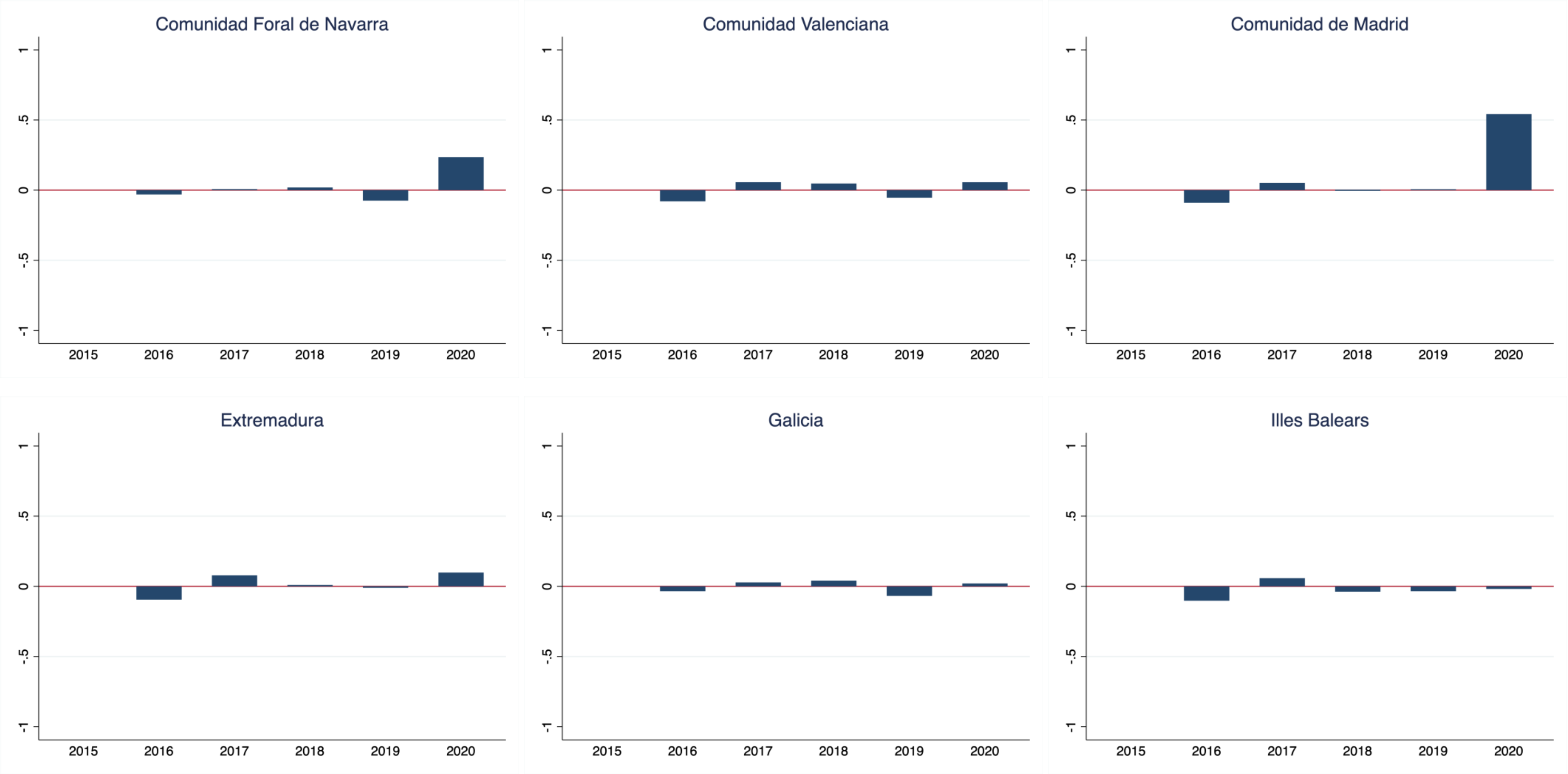

Comunidad Foral de Navarra
Comunidad Valenciana
Comunidad de Madrid
Extremadura
Galicia
Illes Balears
1
.5
0
-.5
-1
2015
2016
2017
2018
2019
2020

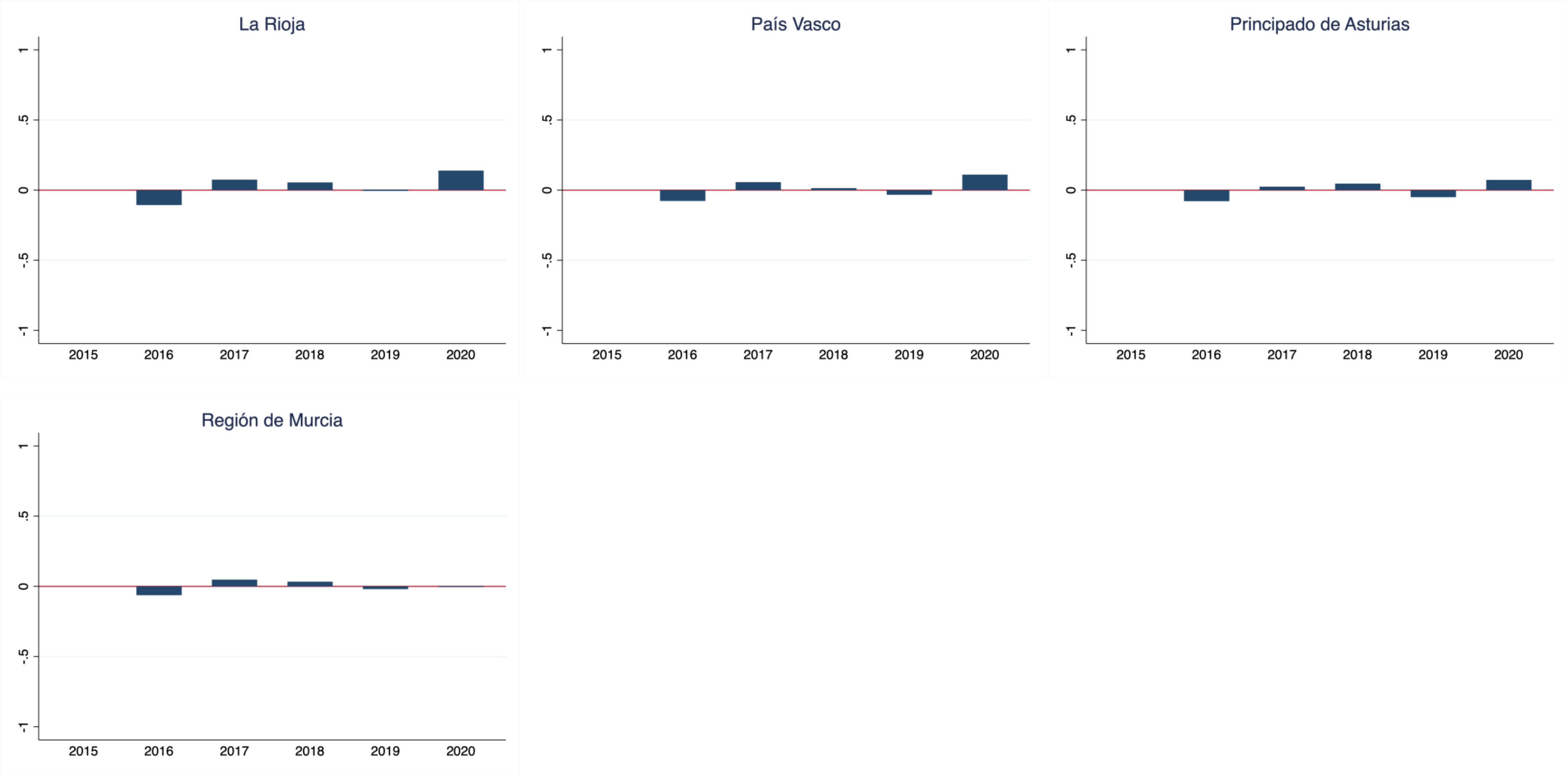
La Rioja
País Vasco
Principado de Asturias
Región de Murcia
1
.5
0
-.5
-1
2015
2016
2017
2018
2019
2020

SWEDEN

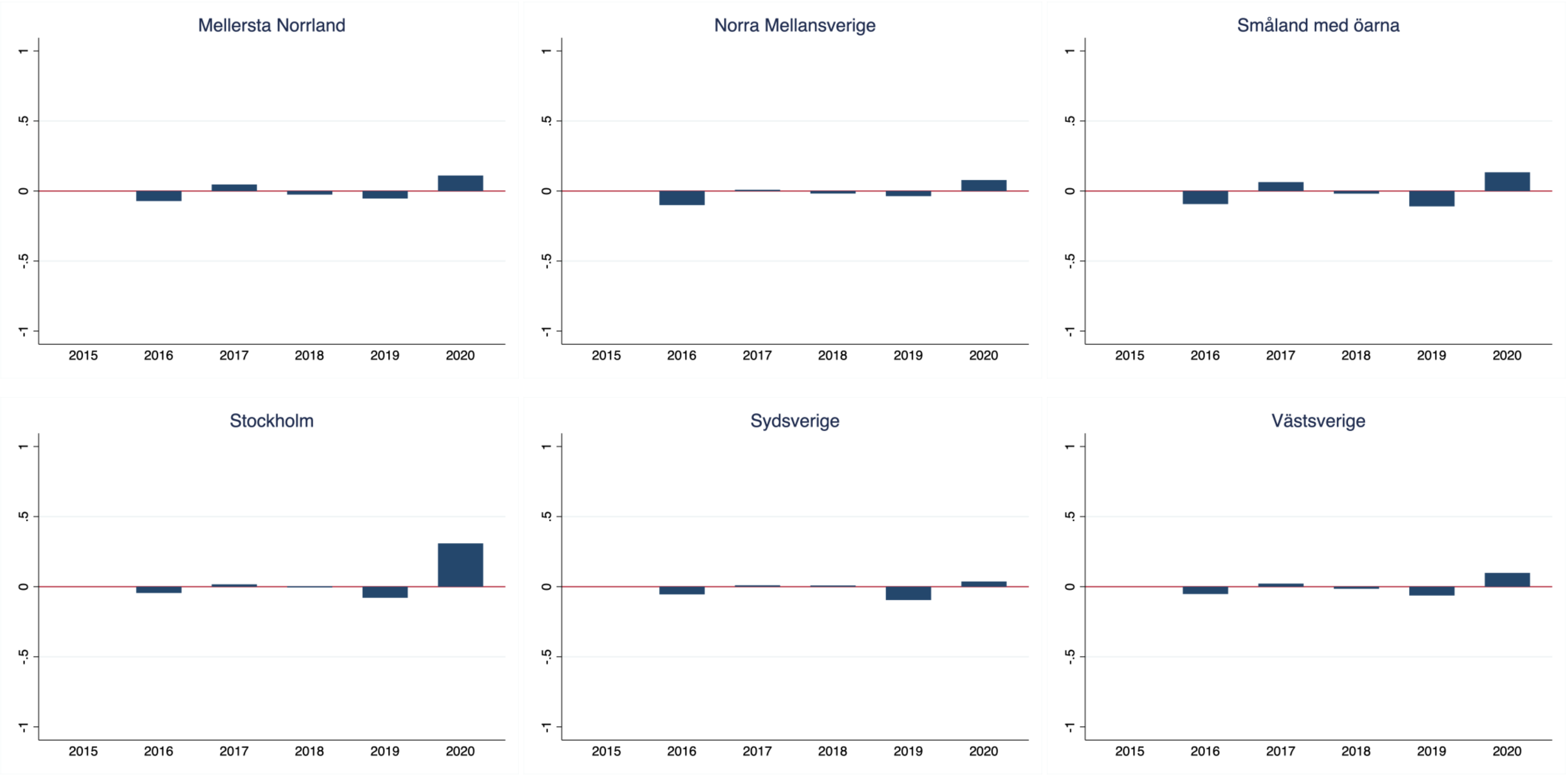
Mellersta Norrland
Norra Mellansverige
Småland med öarna
Stockholm
Sydsverige
Västsverige
1
.5
0
-.5
-1
2015
2016
2017
2018
2019
2020

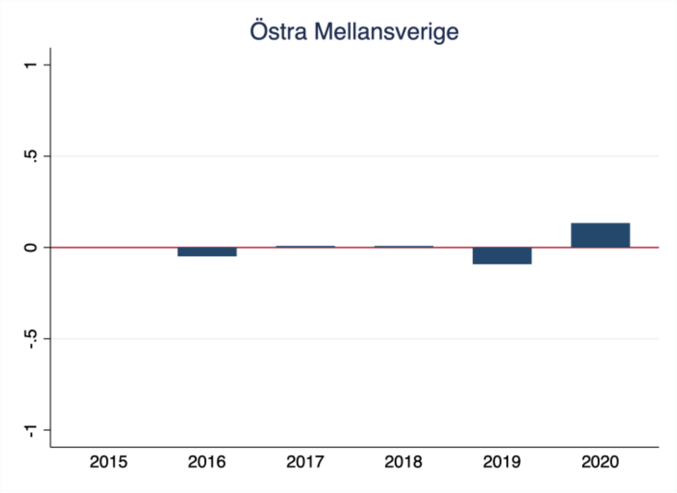

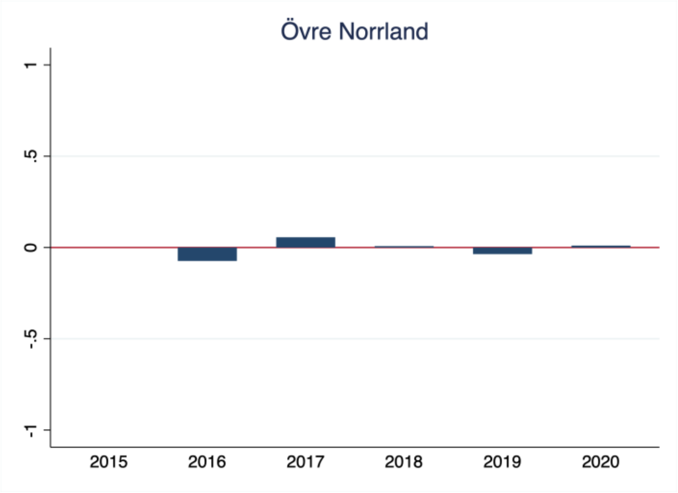

SWITZERLAND

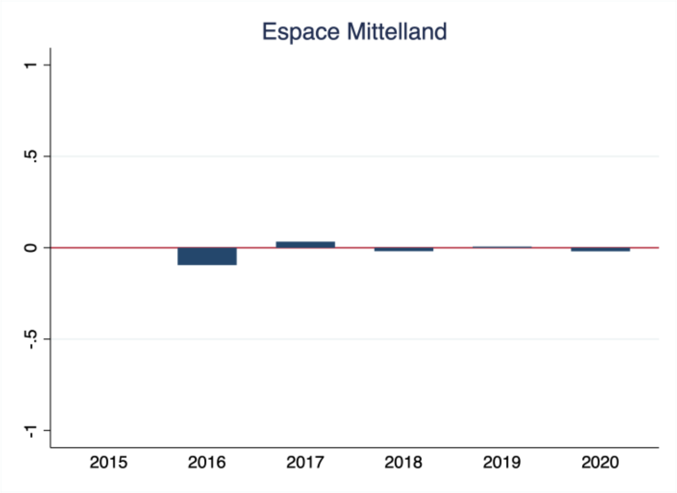

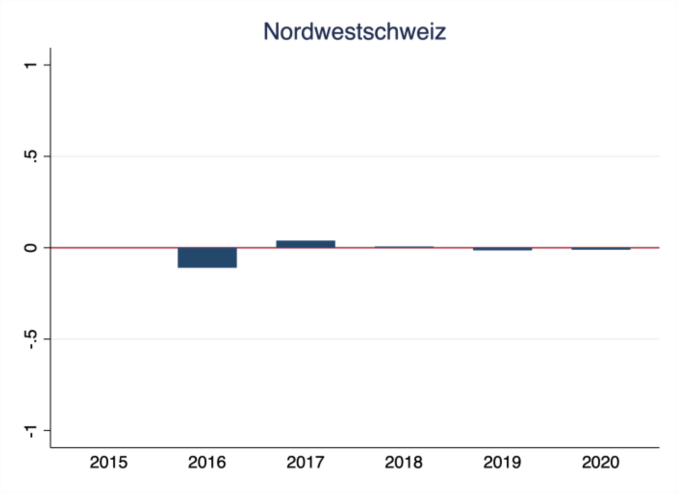

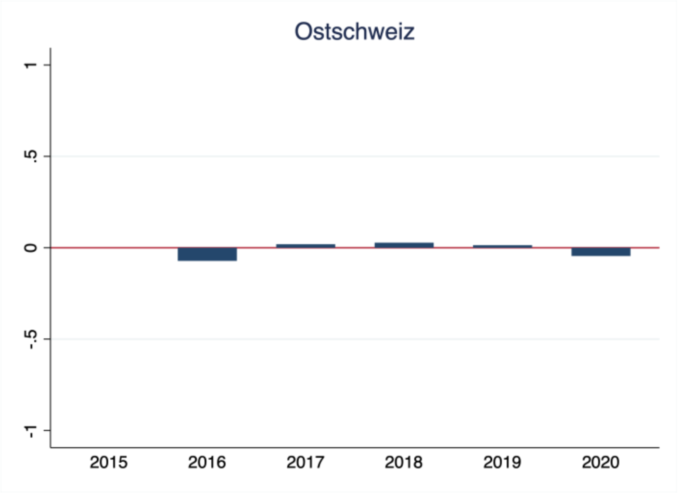

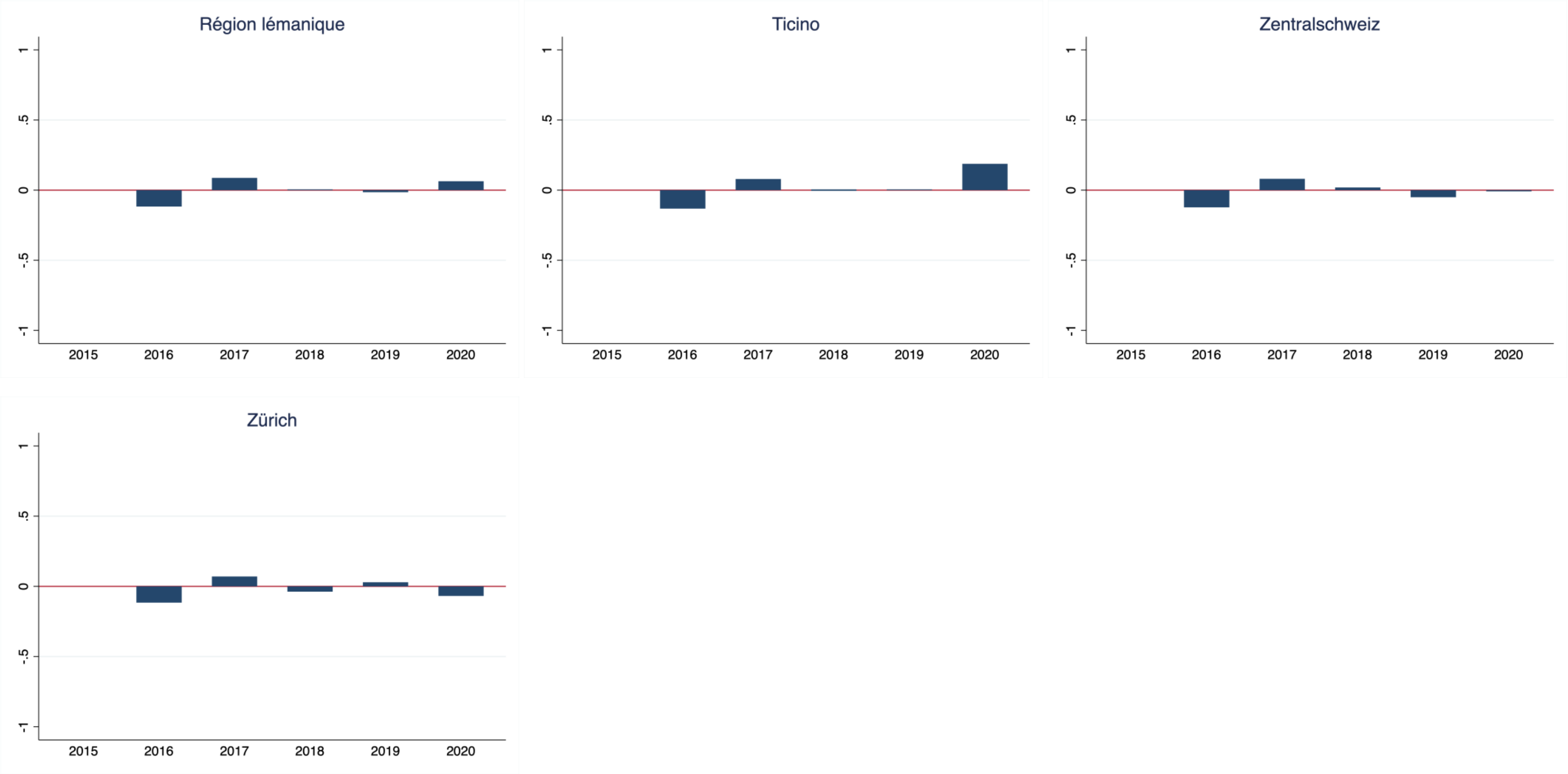
Région lémanique
Ticino
Zentralschweiz
Zürich
1
.5
0
-.5
-1
2015
2016
2017
2018
2019
2020

UNITED KINGDOM

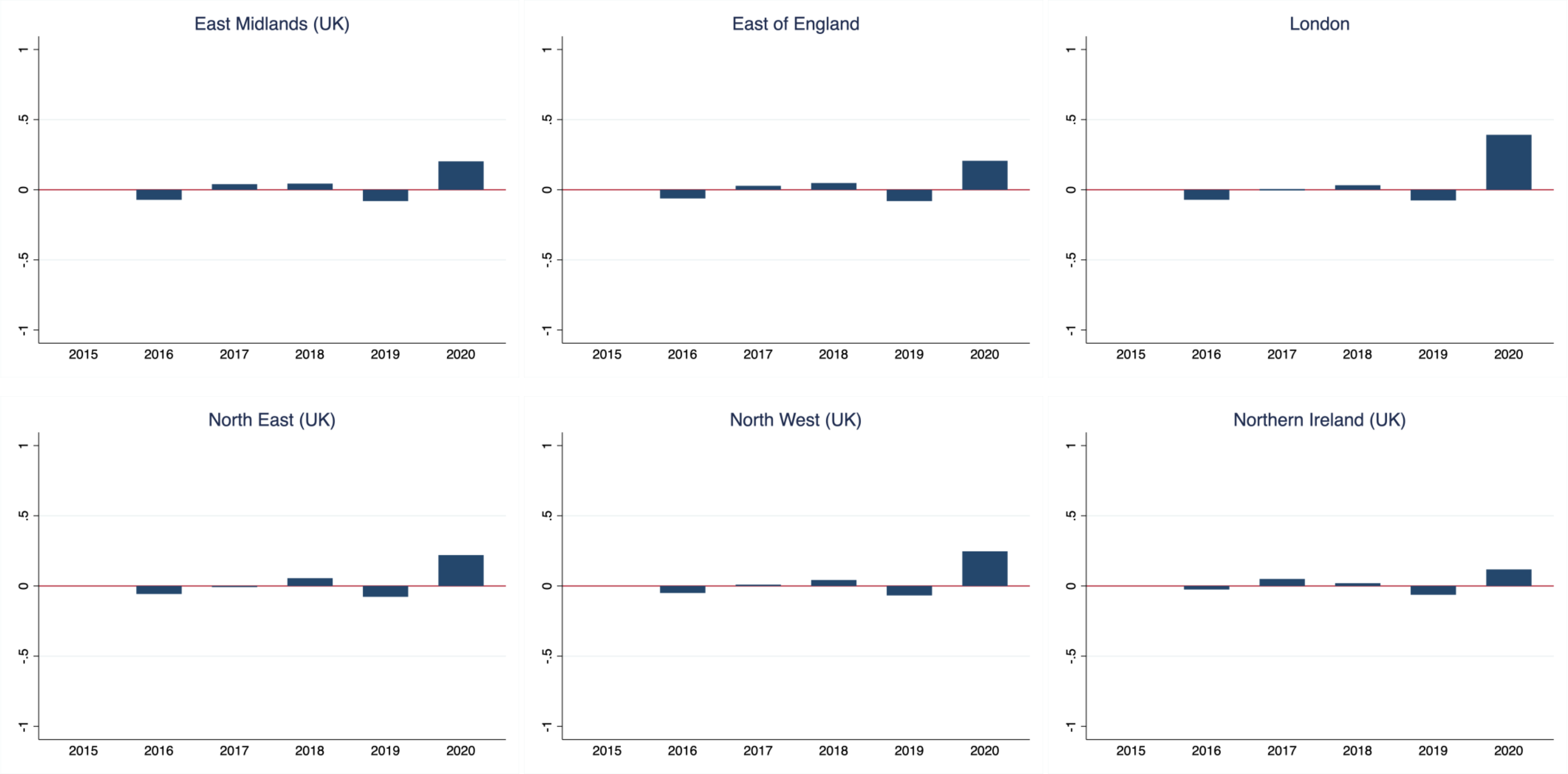

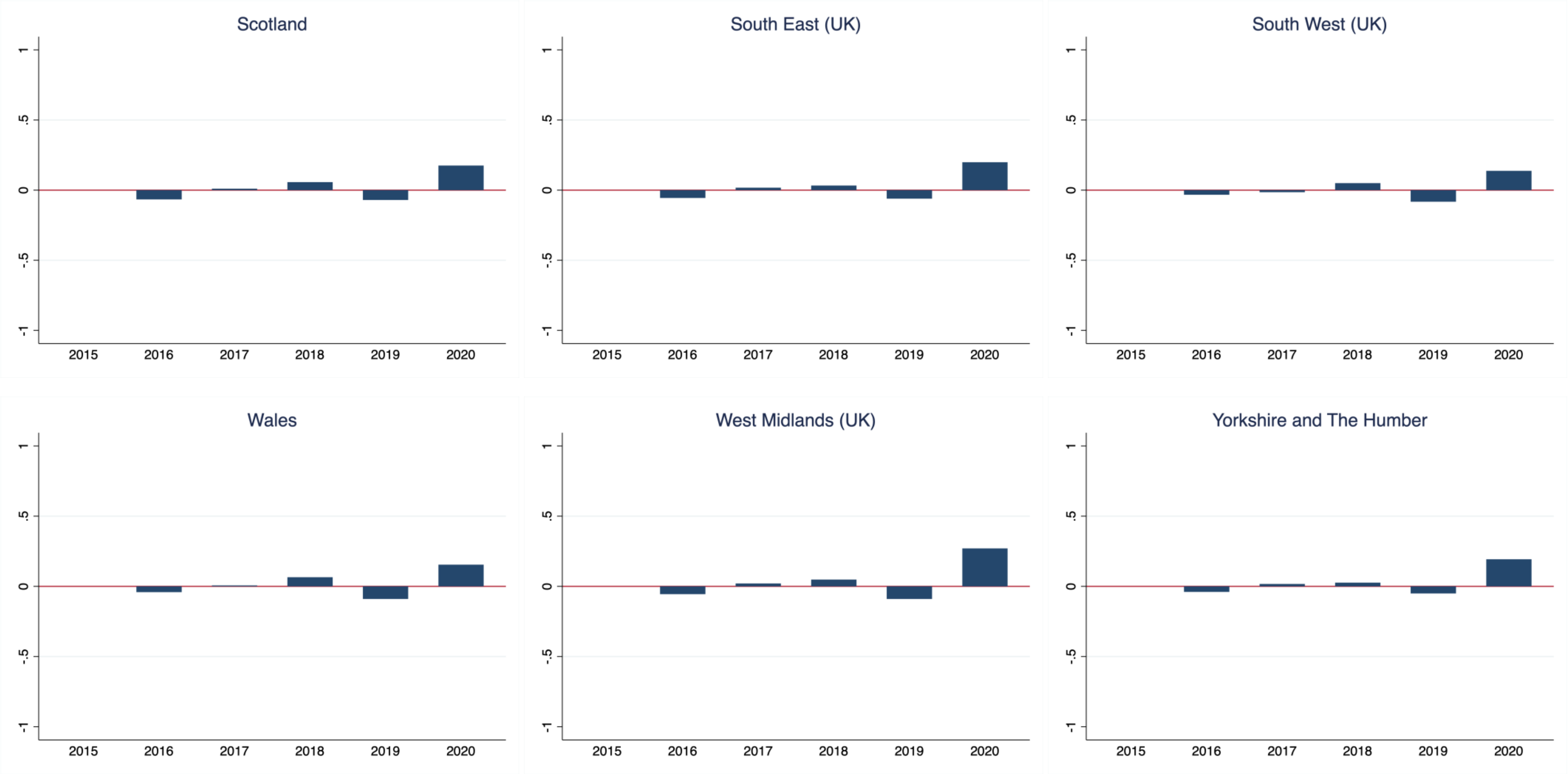
Scotland
South East (UK)
South West (UK)
Wales
West Midlands (UK)
Yorkshire and The Humber
1
.5
0
-.5
-1
2015
2016
2017
2018
2019
2020
